# Synthesis, Absolute Configuration, Antibacterial, and Antifungal Activities of Novel Benzofuryl *β*-Amino Alcohols

**DOI:** 10.3390/ma13184080

**Published:** 2020-09-14

**Authors:** Agnieszka Tafelska-Kaczmarek, Renata Kołodziejska, Marcin Kwit, Bartosz Stasiak, Magdalena Wypij, Patrycja Golińska

**Affiliations:** 1 Department of Organic Chemistry, Faculty of Chemistry, Nicolaus Copernicus University in Toruń, 7 Gagarin Street, 87-100 Toruń, Poland; 2 Department of Medical Biology and Biochemistry, Faculty of Medicine, Nicolaus Copernicus University in Toruń, Collegium Medicum in Bydgoszcz, 24 Karłowicz Street, 85-092 Bydgoszcz, Poland; renatak@cm.umk.pl; 3 Faculty of Chemistry, Adam Mickiewicz University, 8 Uniwersytetu Poznańskiego Street, 61-614 Poznań, Poland; Marcin.Kwit@amu.edu.pl (M.K.); bartek.stasiak@vp.pl (B.S.); 4 Department of Microbiology, Faculty of Biology and Veterinary Sciences, Nicolaus Copernicus University in Toruń, 1 Lwowska Street, 87-100 Toruń, Poland; mwypij@umk.pl (M.W.); golinska@umk.pl (P.G.)

**Keywords:** benzofuran, amino alcohol, asymmetric synthesis, transfer hydrogenation, antifungal activity, antibacterial activity, imidazole, triazole

## Abstract

A series of new benzofuryl *α*-azole ketones was synthesized and reduced by asymmetric transfer hydrogenation (ATH). Novel benzofuryl *β*-amino alcohols bearing an imidazolyl and triazolyl substituents were obtained with excellent enantioselectivity (96–99%). The absolute configuration (*R*) of the products was confirmed by means of electronic circular dichroism (ECD) spectroscopy supported by theoretical calculations. Selected benzofuryl *α*-azole ketones were also successfully asymmetrically bioreduced by fungi of *Saccharomyces cerevisiae* and *Aureobasidium pullulans* species. Racemic and chiral *β*-amino alcohols, as well as benzofuryl *α*-amino and *α*-bromo ketones were evaluated for their antibacterial and antifungal activities. From among the synthesized *β*-amino alcohols, the highest antimicrobial activity was found for (*R*)-1-(3,5-dimethylbenzofuran-2-yl)-2-(1*H*-imidazol-1-yl)ethan-1-ol against *S. aureus* ATCC 25923 (MIC = 64, MBC = 96 μg mL^−1^) and (*R*)-1-(3,5-dimethylbenzofuran-2-yl)-2-(1*H*-1,2,4-triazol-1-yl)ethan-1-ol against yeasts of *M. furfur* DSM 6170 (MIC = MBC = 64 μg mL^−1^). In turn, from among the tested ketones, 1-(benzofuran-2-yl)-2-bromoethanones (1–4) were found to be the most active against *M. furfur* DSM 6170 (MIC = MBC = 1.5 μg mL^−1^) (MIC—minimal inhibitory concentration, MBC—minimal biocidal concentration).

## 1. Introduction

**Figure 1:**
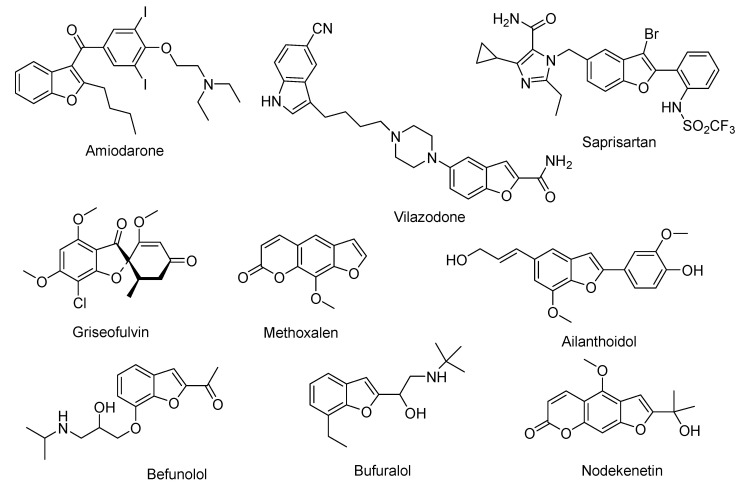
Drugs containing the benzofuran moiety.

Benzofuran is considered as a very important oxygen-containing heterocyclic compound due to its diverse biological profile [1]. Many of the clinically approved medicines are synthetic and naturally occurring substituted benzofuran derivatives which display various biological (antitumor, antiarrhythmic, antidepressant, antihyperglycemic, antimicrobial, antibiotic, antiparasitic) activities. The selected known drugs containing the benzofuran moiety are depicted in Figure 1. The most recognized synthetic drug is amiodarone, a highly effective antiarrhythmic agent with class III activity, used for both, ventricular and supraventricular types of arrhythmia [2]. Saprisartan is used in the treatment of hypertension and heart failure [3] whereas vilazodone is a medication used to treat major depressive disorders [4].

The benzofuran scaffold occurs in a great number of natural compounds having physiological, pharmacological, and toxic properties. For example, griseofulvin—a fermentation product of three species, *Penicillium griseofulvum*, *Penicillium janczewski*, *Penicillium patulum*, is a natural fungistatic antibiotic used for fungal skin, hair, and nail infections [5]. In turn, methoxalen obtained from four species of the *Heracleum* genus in the *Apiaceae* carrot family is a drug used to treat psoriasis, eczema, vitiligo, or cutaneous lymphomas [6]. Another benzofuran derivative, ailanthoidol, isolated from *Zanthoxylum ailanthoidol,* a Chinese herbal medicine, has been reported to possess anticancer, antiviral, immunosuppressive, antioxidant, antifungal, and antifeedant activities [7]. Benzofuryl alcohols as well as *β*-amino alcohols also exhibit various biological properties. Nodekenetin is effective against skin diseases including cutaneous T-cell lymphoma, vitiligo, atopic dermatitis, and psoriasis [8]. Both, befunolol and bufuralol are *β*-adrenergic receptor antagonist, wherein befunolol is used to treat glaucoma [9], while bufuralol is a commonly used marker of hepatic CYP 2D6 activity [10]. Due to their wide scope of pharmacological activity and the ability to modify their structure, benzofuran derivatives are a promising basis for the development of new potentially useful therapeutics.

On the other hand, azoles are an important and well-known class of antifungal drugs [11,12]. Clinically used azole antifungals (miconazole, econazole, and ketoconazole) have recently gained attention as they also show potent activity against some common bacterial strains, especially Gram-positive bacteria, including the methicillin-resistant *Staphylococcus aureus* (MRSA) [13,14,15]. However, the efficient activity of azole-based compounds against Gram-negative bacteria (e.g., *Escherichia coli*, *Pseudomonas aeruginosa, Proteus vulgaris, Shigella sonnei, Salmonella typhi*) has been also recorded [16]. Moreover, a combination of antifungal and antibacterial efficacy in a single drug (dual-acting hits) could be a very powerful tool, especially in some complicated cases [12]. Therefore, efforts to design and screen new azoles or azol-based compounds for their antibacterial effects have been intensified in recent years [12,16,17,18].

**Figure 2:**
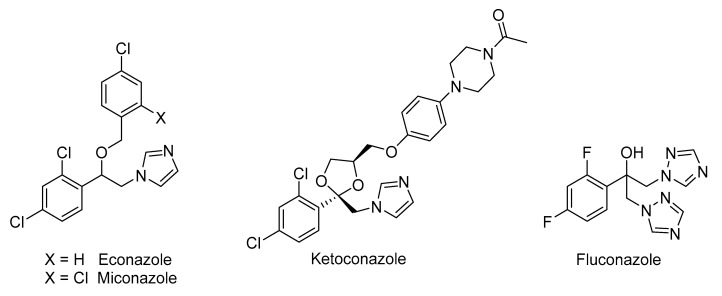
Azole-antifungal drugs.

The main drugs used for the treatment of invasive fungal infections (above-mentioned econazole, miconazole, fluconazole, ketoconazole) contain derivatives of 1-phenylethanol bearing imidazolyl and triazolyl substituents in the alpha position to the alcohol [19,20] (Figure 2).

The main azole drugs possess at least two principal pharmacophoric groups, i.e., the iron coordinating group (the azole ring, able to interact with the heme iron) and the aromatic group (as a hydrophobic moiety adjacent to it) [21]. In the commonly used drugs of this type, the racemic *β*-amino alcohols have found application; however, the pharmaceutical industry is guided by the trend suggesting the application of a single isomer of a chiral compound [22]. Many scientists focus on the synthesis of optically pure compounds and carry out studies of the biological activity of these compounds in comparison with racemic mixtures [21,23]. The Scipione Group research, which showed that the levoraotatory enantiomer of 1-(4-fluorophenyl)-2-(1H-imidazol-1-yl)ethyl biphenyl-4-carboxylate is 500 times more active towards strains of the *Candida* species than the dextrorotatory one, and about 10 times more active than racemate and fluconazole, constitutes an excellent example [21].

The asymmetric transfer hydrogenation (ATH) of the corresponding ketones is an attractive method for the synthesis of chiral *β*-amino alcohols [24,25,26,27,28,29]. This reduction method is versatile, operationally simple (avoids the use of hydrogen gas), and highly stereoselective [30,31,32].

Chiroptical spectroscopy, mostly electronic circular dichroism (ECD) and optical rotation (OR) constitutes a convenient alternative and/or a complementary method to the anomalous X-ray scattering on crystals [33,34,35]. In the latter case, the appropriate crystal(s) for the measurement is required, and the results apply to the “frozen” structures in the solid state. The former group of experimental methods is based on the nondestructive interaction of randomly oriented molecules with linearly or circularly polarized light [33]. As the majority of optically active compounds is conformationally flexible, the measured ECD spectra or optical rotation values are the combination of configurational and conformational effects. This may hinder the interpretation of the results; on the other hand, however, this gives an advantage to the chiroptical methods [36,37]. It is worth mentioning that the most common analytical technique, namely the NMR spectroscopy, might be applied to the determination of the absolute configuration, albeit under special conditions. For molecules having only one chirality element, it is connected with the necessity of either prior derivatization by the chiral derivatizing agent (CDA) or the use of the chiral solvating agent (CSA) that weakly binds to the substrate. In the former case, one enantiomer of the compound under study is derivatized with the two enantiomers of the CDA, and the NMR spectra of the resulting diastereoisomers are compared. This methodology seems to be simple; however, in the case of more complex compounds, the assignment of the absolute configuration is not straightforward due to the overlapping of diagnostic signals and/or small differences of chemical shifts. The applications of the NMR methods for determining the absolute stereochemistry have been recently reviewed [38,39,40,41].

In the previous papers, we studied the asymmetric syntheses of benzofuryl *β*-amino alcohols with primary, secondary, and tertiary amine groups [42,43,44]. Herein, we report the synthesis of new *β*-amino alcohols bearing imidazolyl and triazolyl substituents, determination of the absolute configuration of chiral alcohols, and evaluation of antimicrobial activities of racemic and chiral compounds.

## 2. Materials and Methods

### 2.1. General Information

- Nuclear magnetic resonance (NMR) spectra were recorded on Bruker AMX 300 MHz, Bruker Avance III 400 MHz and Brucker Avance III 700 MHz spectrometers (Ettlingen, Germany). Chemical shifts were reported in δ ppm from tetramethylsilane (TMS) as the internal standard.
- High-performance liquid chromatography (HPLC) analyses were performed on a Shimadzu LC-10AT chromatograph (Shimadzu, Kyoto, Japan).
- Optical rotations were measured on an automatic polarimeter PolAAr 3000 (Optical Activity Ltd., Ramsey, Cambridgeshire, UK) and recorded at 20 °C on a Jasco P-2000 polarimeter (Easton, MD, USA).
- The ECD and UV (ultraviolet) spectra were measured using a Jasco J-810 spectropolarimeter (Easton, MD, USA) at room temperature, in acetonitrile solution, and with the use of a quartz cell of 0.1 cm optical length. The analytes concentration ranged from 1.0 to 2.0 × 10^−4^ mol L^−1^.
- IR (infrared) analyses were recorded on an Alpha FT-IR spectrometer from Bruker (Ettlingen, Germany).
- Melting points were determined in open glass capillaries and are uncorrected.
- Elemental analyses were performer on a Vario MACRO CHN, ELEMENTAR Anaylsensysteme GmbH instrument (Langenselbold, Germany).

### 2.2. Materials

Silica gel 60, Merck 230–400 mesh was used for the preparative column chromatography. The analytical TLC was performed using Merck TLC Silica gel 60 F_254_ 0.2 mm aluminum plates (Merck, Darmstadt, Germany).

1*H*-imidazole, 1*H*-1,2,4-triazole, formic acid, triethylamine, sodium borohydride, glucose, Na_2_HPO_4_, NaH_2_PO_4_, cysteine, allyl alcohol, ethyl chloroacetate, ethyl (9-antryl)glyoxylate (AMA-1) were commercial products. Boni Protect^®^ from Koppert Biological Systems (Vienna, Austria) contains germinating fungal *Aureobasidium pullulans* DSM 14940 and 14941 strains (500 g per 1 kg of the preparation). 1-(Benzofuran-2-yl)-2-bromoethanone (**1**) [45], 2-bromo-1-(7-ethylbenzofuran-2-yl)ethanone (**2**) [46], 2-bromo-1-(3-methylbenzofuran-2-yl)-ethanone (**3**) [47], [((*R*,*R*)-2-Amino-1,2-diphenylethyl)[(4-tolyl)sulfonyl]amido](chloro)pentamethylcyclopentadienylrhodium(I) (RhCl[(*R*,*R*)-TsDPEN](C_5_Me_5_)) [48] were prepared according to the literature. 2-Bromo-1-(3,5-dimethylbenzofuran-2-yl)ethanone (**4**, mp 82–84 °C, ^1^H NMR (700 MHz, CDCl_3_) δ (ppm): 2.50 (s, 3H, CH_3_), 2.63 (s, 3H, CH_3_), 4.53 (s, 2H, CH_2_), 7.35 (dd, 1H, *J* = 8.4 Hz, *J* = 1.4 Hz, CH), 7.42 (d, 1H, *J* = 9.1 Hz, CH), 7.46–7.47 (m, 1H). ^13^C NMR (75 MHz, CDCl_3_) δ (ppm): 9.56 (CH_3_), 21.32 (CH_3_), 32.07 (CH_2_), 111.78 (CH), 121.10 (CH), 127.14 (C), 129.19 (C), 130.55 (CH), 133.22 (C), 146.06 (C), 152.78 (C), 183.83 (CO). IR, neat (cm^−1^): 2989, 2940, 1680, 1572, 1383, 1312, 1290, 1126, 986, 785, 680, 430) was prepared from 1-(3,5-dimethylbenzofuran-2-yl)ethanone and pyridinium tribromide according to the known protocol [46].

### 2.3. General Procedures

#### 2.3.1. Method of α-Amino Ketones **5**–**12** Preparation

Triethylamine (0.81 g, 8.0 mmol) and then, dropwise, a solution of the corresponding benzofuryl *α*-bromo ketone (4.0 mmol) in acetonitrile (12 mL) were added to a solution of 1*H*-imidazole or 1*H*-1,2,4-triazole (8.0 mmol) in acetonitrile (3 mL) (for 1*H*-1,2,4-triazole 5 mL of acetonitrile). The mixture was stirred at room temperature for 24 h. The reaction mixture was concentrated in vacuum, and the residue was suspended in water and extracted with dichloromethane. The organic layer was dried over anhydrous MgSO_4_ and the solvent was evaporated in vacuum. The crude product was purified by column chromatography on silica gel (hexane/ethyl acetate/dichloromethane/methanol, 5:5:3:1.5).

1-(benzofuran-2-yl)-2-(1*H*-imidazol-1-yl)ethanone (**5**)

Yellow solid, 0.25 g, 53% yield, mp 145–148 °C, lit. 152 °C [49]. ^1^H NMR (700 MHz, CDCl_3_) δ (ppm): 5.42 (s, 2H, CH_2_), 7.02 (s, 1H, CH), 7.17 (s, 1H, CH), 7.36 (ddd, 1H, *J* = 8.4 Hz, *J* = 7.0 Hz, *J* = 1.4 Hz, CH), 7.55 (ddd, 1H, *J* = 8.4 Hz, *J* = 7.0 Hz, *J* = 1.4 Hz, CH), 7.60 (dq, 1H, *J* = 8.4 Hz, *J* = 0.7 Hz, CH), 7.63 (s, 1H, CH), 7.70 (s, 1H, CH), 7.75 (dq, 1H, *J* = 7.7 Hz, *J* = 0.7 Hz, CH). ^13^C NMR (175 MHz, CDCl_3_) δ (ppm): 52.07 (CH_2_), 112.08 (CH), 113.76 (CH), 119.87 (CH), 123.29 (CH), 124.07 (CH), 126.33 (C), 128.78 (CH), 129.23 (CH), 137.75 (CH), 149.97 (C), 155.38 (C), 182.79 (CO). (Figure S1 in the Supplementary Materials) IR neat (cm^−1^): 3110, 2919, 1693, 1558, 1510, 1475, 1245, 1156, 1081, 1007, 842, 770, 429. Anal. calcd for C_13_H_10_N_2_O_2_: C, 69.02; H, 4.46; N, 12.38. Found: C, 69.20; H, 4.53; N, 12.31.

1-(7-ethylbenzofuran-2-yl)-2-(1*H*-imidazol-1-yl)ethanone (**6**)

Red solid, 1.04 g, 55% yield, mp 84–87 °C. ^1^H NMR (700 MHz, CDCl_3_) δ (ppm): 1.39 (t, 3H, *J* = 7.7 Hz, CH_3_), 3.00 (q, 2H, *J* = 7.7 Hz, CH_2_), 5.40 (s, 2H, CH_2_), 7.01 (s, 1H, CH), 7.15 (s, 1H, CH), 7.28 (t, 1H, *J* = 7.7 Hz, CH), 7.36 (dq, 1H, *J* = 7.7 Hz, *J* = 0.7 Hz, CH), 7.56 (dd, 1H, *J* = 7.7 Hz, *J* = 0.7 Hz, CH), 7.61 (s, 1H, CH), 7.62 (s, 1H, CH). ^13^C NMR (175 MHz, CDCl_3_) δ (ppm): 13.58 (CH_3_), 22.32 (CH_2_), 52.08 (CH_2_), 114.11 (CH), 119.81 (CH), 120.65 (CH), 124.27 (CH), 126.03 (C), 127.67 (CH), 128.56 (C), 129.29 (CH), 137.75 (CH), 149.76 (C), 154.17 (C), 182.76 (CO). (Figure S2 in the Supplementary Materials) IR neat (cm^−1^): 3115, 2972, 1693, 1550, 1487, 1286, 1149, 1014, 824, 742, 662, 619. Anal. calcd for C_15_H_14_N_2_O_2_: C, 70.85; H, 5.55; N, 11.02. Found: C, 70.69; H, 5.45; N, 11.17.

2-(1*H*-imidazol-1-yl)-1-(3-methylbenzofuran-2-yl)ethanone (**7**)

Yellow solid, 0.49 g, 49% yield, mp 151–153 °C. ^1^H NMR (700 MHz, CDCl_3_) δ (ppm): 2.63 (s, 3H, CH_3_), 5.40 (s, 2H, CH_2_), 7.00 (s, 1H, CH), 7.15 (s, 1H, CH), 7.36 (ddd, 1H, *J* = 8.4 Hz, *J* = 5.6 Hz, *J* = 2.8 Hz, CH), 7.54 (d, 1H, *J* = 0.7 Hz, CH), 7.55 (dd, 1H, *J* = 2.8 Hz, *J* = 0.7 Hz, CH), 7.57 (s, 1H, CH), 7.70 (dt, 1H, *J* = 7.7 Hz, *J* = 0.7 Hz, CH). ^13^C NMR (175 MHz, CDCl_3_) δ (ppm): 9.03 (CH_3_), 52.58 (CH_2_), 111.84 (CH), 119.86 (CH), 121.47 (CH), 123.41 (CH), 127.14 (C), 128.55 (C), 128.81 (CH), 129.20 (CH), 137.83 (CH), 145.47 (C), 153.97 (C), 184.43 (CO). (Figure S3 in the Supplementary Materials) IR neat (cm^−1^): 3112, 2919, 1682, 1573, 1366, 1255, 1129, 980, 819, 743, 658, 427. Anal. calcd for C_14_H_12_N_2_O_2_: C, 69.99; H, 5.03; N, 11.66. Found: C, 70.29; H, 5.25; N, 11.82.

1-(3,5-dimethylbenzofuran-2-yl)-2-(1*H*-imidazol-1-yl)ethanone (**8**)

Yellow solid, 0.33 g, 58% yield, mp 153–155 °C. ^1^H NMR (700 MHz, CDCl_3_) δ (ppm): 2.49 (s, 3H, CH_3_), 2.60 (s, 3H, CH_3_), 5.42 (s, 2H, CH_2_), 7.02 (s, 1H, CH), 7.17 (s, 1H, CH), 7.36 (ddd, 1H, *J* = 8.4 Hz, *J* = 1.4 Hz, *J* = 0.7 Hz, CH), 7.42 (dd, 1H, *J* = 8.4 Hz, *J* = 0.7 Hz, CH), 7.46–7.47 (m, 1H, CH), 7.71 (s, 1H, CH). ^13^C NMR (175 MHz, CDCl_3_) δ (ppm): 9.03 (CH_3_), 20.97 (CH_3_), 52.57 (CH_2_), 111.36 (CH), 119.91 (CH), 120.90 (CH), 126.98 (C), 128.63 (C), 128.95 (C), 130.46 (CH), 133.12 (CH), 137.78 (CH), 145.64 (C), 152.53 (C), 184.28 (CO). (Figure S4 in the Supplementary Materials) IR neat (cm^−1^): 3101, 2916, 2854, 1690, 1574, 1365, 1259, 1128, 977, 817, 658, 428. Anal. calcd for C_15_H_14_N_2_O_2_: C, 70.85; H, 5.55; N, 11.02. Found: C, 70.94; H, 5.73; N, 11.19.

1-(benzofuran-2-yl)-2-(1*H*-1,2,4-triazol-1-yl)ethanone (**9**)

Yellow solid, 0.72 g, 42% yield, mp 169–171 °C, lit. 172 °C [49]. ^1^H NMR (400 MHz, CDCl_3_) δ (ppm): 5.70 (s, 2H, CH_2_), 7.39 (ddd, 1H, *J* = 8.0 Hz, *J* = 7.2 Hz, *J* = 0.8 Hz, CH), 7.57 (ddd, 1H, *J* = 8.4 Hz, *J* = 7.2 Hz, *J* = 1.2 Hz, CH), 7.63 (dq, 1H, *J* = 8.4 Hz, *J* = 0.8 Hz, CH), 7.70 (d, 1H, *J* = 0.8 Hz, CH), 7.78 (dq, 1H, *J* = 8.0 Hz, *J* = 0.8 Hz, CH), 8.06 (s, 1H, CH), 8.39 (s, 1H, CH). ^13^C NMR (100 MHz, CDCl_3_) δ (ppm): 54.92 (CH_2_), 112.54 (CH), 114.56 (CH), 123.72 (CH), 124.51 (CH), 126.71 (C), 129.28 (CH), 144.86 (CH), 150.23 (C), 152.08 (CH), 155.93 (C), 181.91 (CO). (Figure S5 in the Supplementary Materials) IR neat (cm^−1^): 3123, 2922, 1692, 1562, 1353, 1271, 1135, 1020, 840, 755, 674, 433. Anal. calcd for C_12_H_9_N_3_O_2_: C, 63.43; H, 3.99; N, 18.49. Found: C, 63.50; H, 4.08; N, 18.57.

1-(7-ethylbenzofuran-2-yl)-2-(1*H*-1,2,4-triazol-1-yl)ethanone (**10**)

Yellow solid, 0.54 g, 47% yield, mp 115–118 °C. ^1^H NMR (700 MHz, CDCl_3_) δ (ppm): 1.38 (t, 3H, *J* = 7.7 Hz, CH_3_), 2.99 (q, 2H, *J* = 7.7 Hz, CH_2_), 5.69 (s, 2H, CH_2_), 7.29 (t, 1H, *J* = 7.0 Hz, CH), 7.37 (dq, 1H, *J* = 7.0 Hz, *J* = 0.7 Hz, CH), 7.58 (dd, 1H, *J* = 7.7 Hz, *J* = 1.4 Hz, CH), 7.68 (s, 1H, CH), 8.03 (s, 1H, CH), 8.31 (s, 1H, CH). ^13^C NMR (175 MHz, CDCl_3_) δ (ppm): 13.56 (CH_3_), 22.34 (CH_2_), 54.54 (CH_2_), 114.48 (CH), 120.69 (CH), 124.31 (CH), 126.00 (C), 127.80 (CH), 128.63 (C), 144.48 (CH), 149.58 (C), 151.67 (CH), 154.29 (C), 181.48 (CO). (Figure S6 in the Supplementary Materials) IR neat (cm^−1^): 3105, 2965, 1681, 1548, 1269, 1142, 1021, 919, 848, 742, 666, 420. Anal. calcd for C_14_H_13_N_3_O_2_: C, 65.87; H, 5.13; N, 16.46. Found: C, 65.77; H, 5.10; N, 16.47.

1-(3-methylbenzofuran-2-yl)-2-(1*H*-1,2,4-triazol-1-yl)ethanone (**11**)

Yellow solid, 0.48 g, 53% yield, mp 167–170 °C. ^1^H NMR (700 MHz, CDCl_3_) δ (ppm): 2.63 (s, 3H, CH_3_), 5.69 (s, 2H, CH_2_), 7.37 (ddd, 1H, *J* = 7.7 Hz, *J* = 5.6 Hz, *J* = 2.1 Hz, CH), 7.55 (d, 1H, *J* = 0.7 Hz, CH), 7.56 (dd, 1H, *J* = 2.8 Hz, *J* = 0.7 Hz, CH), 7.71 (dt, 1H, *J* = 7.7 Hz, *J* = 1.4 Hz, CH), 8.03 (s, 1H, CH), 8.27 (s, 1H, CH). ^13^C NMR (100 MHz, CDCl_3_) δ (ppm): 9.41 (CH_3_), 55.49 (CH_2_), 112.30 (CH), 121.89 (CH), 123.85 (CH), 127.84 (C), 128.90 (C), 129.31 (CH), 144.93 (C), 145.73 (CH), 152.05 (CH), 154.49 (C), 183.52 (CO). (Figure S7 in the Supplementary Materials) IR neat (cm^−1^): 3136, 2932, 1683, 1574, 1366, 1264, 1131, 1016, 870, 767, 680, 426. Anal. calcd for C_13_H_11_N_3_O_2_: C, 64.72; H, 4.60; N, 17.42. Found: C, 64.79; H, 4.70; N, 17.53.

1-(3,5-dimethylbenzofuran-2-yl)-2-(1*H*-1,2,4-triazol-1-yl)ethanone (**12**)

Yellow solid, 0.96 g, 83% yield, mp 157–159 °C. ^1^H NMR (400 MHz, CDCl_3_) δ (ppm): 2.51 (s, 3H, CH_3_), 2.62 (s, 3H, CH_3_), 5.70 (s, 2H, CH_2_), 7.35–7.46 (m, 2H, 2xCH), 7.48–7.49 (m, 1H, CH), 8.05 (s, 1H, CH), 8.33 (s, 1H, CH). ^13^C NMR (100 MHz, CDCl_3_) δ (ppm): 9.42 (CH_3_), 21.35 (CH_3_), 55.51 (CH_2_), 111.83 (CH), 121.31 (CH), 127.70 (C), 128.99 (C), 130.97 (CH), 133.56 (C), 144.85 (CH), 145.92 (C), 151.78 (CH), 153.07 (C), 183.37 (CO). (Figure S8 in the Supplementary Materials) IR neat (cm^−1^): 3130, 2985, 2930, 1673, 1573, 1308, 1267, 1131, 1017, 826, 708, 635, 427. Anal. calcd for C_14_H_13_N_3_O_2_: C, 65.87; H, 5.13; N, 16.46. Found: C, 65.92; H, 5.16; N, 16.58.

#### 2.3.2. General Procedure for the Asymmetric Transfer Hydrogenation of α-Amino Ketones **5**–**12**

RhCl[(*R*,*R*)-TsDPEN](C_5_Me_5_) (2.2 mg, 0.0035 mmol) and triethylamine (0.15 g, 3.3 mmol) were added to a solution of the corresponding benzofuryl *α*-amino ketone **5**–**12** (0.7 mmol) in dichloromethane (5 mL) at nitrogen atmosphere. Formic acid (0.15 g, 3.3 mmol) was added dropwise over a period of 30 min. and the reaction mixture was stirred at reflux for 24 h (48 h for *α*-amino ketone **9**–**12**). After cooling to room temperature, the saturated solution of NaHCO_3_ (10 mL) was added, and the organic layer was separated, washed with brine, and dried over anhydrous MgSO_4_. The solvent was evaporated in vacuum and the crude product was purified by column chromatography on silica gel (hexane/ethyl acetate/methanol, 5:5:2).

(*R*)-1-(benzofuran-2-yl)-2-(1*H*-imidazol-1-yl)ethan-1-ol (**13**)

Orange solid, 0.12 g, 79% yield, mp 117–120 °C, ${\left[\alpha \right]}_{D}^{24}$ + 84.61 (*c* 1.82, CHCl_3_); 99% ee, determined by HPLC analysis, Lux^®^ 5 μm Cellulose-4, LC column 250 × 4.6 mm, methanol/water 45:55, flow 0.7 mL/min, (*R*) 35.77 min,99.64%, (*S*) 43.76 min, 0.36%. ^1^H NMR (700 MHz, CDCl_3_) δ (ppm): 4.28 (dd, 1H, *J* = 14.0 Hz, *J* = 7.7 Hz, CH_2_), 4.41 (dd, 1H, *J* = 14.0 Hz, *J* = 3.5 Hz, CH_2_), 4.67 (bs, 1H, OH), 5.08 (ddd, 1H, *J* = 7.7 Hz, *J* = 3.5 Hz, *J* = 0.7 Hz, CH), 6.71 (t, 1H, *J* = 0.7 Hz, CH), 6.84 (s, 1H, CH), 6.85 (s, 1H, CH), 7.23 (td, 1H, *J* = 7.0 Hz, *J* = 0.7 Hz, CH), 7.28 (ddd, 1H, *J* = 8.4 Hz, *J* = 7.0 Hz, *J* = 1.4 Hz, CH), 7.47 (dq, 1H, *J* = 8.4 Hz, *J* = 0.7 Hz, CH), 7.48 (s, 1H, CH), 7.53 (dq, 1H, *J* = 7.7 Hz, *J* = 0.7 Hz, CH). ^13^C NMR (100 MHz, CDCl_3_) δ (ppm): 52.22 (CH_2_), 67.79 (CH), 104.05 (CH), 111.20 (CH), 119.77 (CH), 121.26 (CH), 123.04 (CH), 124.44 (CH), 128.00 (C), 128.06 (CH), 137.62 (CH), 154.84 (C), 156.34 (C). (Figure S9 in the Supplementary Materials) IR neat (cm^−1^): 3115, 2851, 1503, 1448, 1248, 1074, 961, 880, 811, 742, 659, 424. Anal. calcd for C_13_H_12_N_2_O_2_: C, 68.41; H, 5.30; N, 12.27. Found: C, 68.47; H, 5.35; N, 12.36.

(*R*)-1-(7-ethylbenzofuran-2-yl)-2-(1*H*-imidazol-1-yl)ethan-1-ol (**14**)

Dark yellow oil, 0.07 g, 75% yield, ${\left[\alpha \right]}_{D}^{25}$ + 66.67 (*c* 1.05, CHCl_3_); 96% ee, determined by HPLC analysis, Daicel Chiralcel OD-H 5 μm, column 250 × 4.6 mm, hexane/isopropanol 80:20, flow 0.8 mL/min, (*R*) 24.08 min, 98.09%, (*S*) 41.00 min, 1.91%. ^1^H NMR (700 MHz, CDCl_3_) δ (ppm): 1.38 (t, 3H, *J* = 7.0 Hz, CH_3_), 2.97 (q, 2H, *J* = 7.0 Hz, CH_2_), 4.33 (dd, 1H, *J* = 14.0 Hz, *J* = 7.7 Hz, CH_2_), 4.44 (dd, 1H, *J* = 14.0 Hz, *J* = 3.5 Hz, CH_2_), 5.12 (ddd, 1H, *J* = 7.7 Hz, *J* = 3.5 Hz, *J* = 0.7 Hz, CH), 5.67 (bs, 1H, OH), 6.73 (d, 1H, *J* = 0.7 Hz, CH), 6.90 (d, 1H, *J* = 3.5 Hz, CH), 7.15 (dd, 1H, *J* = 7.0 Hz, *J* = 0.7 Hz, CH), 7.19 (t, 1H, *J* = 7.7 Hz, CH), 7.39 (dd, 1H, *J* = 7.7 Hz, *J* = 1.4 Hz, CH), 7.56 (s, 1H, CH). ^13^C NMR (175 MHz, CDCl_3_) δ (ppm): 15.19 (CH_3_), 23.81 (CH_2_), 53.20 (CH_2_), 68.77 (CH), 105.29 (CH), 119.73 (CH), 120.86 (CH), 124.18 (CH), 124.64 (CH), 128.63 (C), 128.69 (C), 128.88 (CH), 138.63 (CH), 154.41 (C), 156.94 (C). (Figure S10 in the Supplementary Materials) IR neat (cm^−1^): 3117, 2966, 2873, 1509, 1425, 1285, 1178, 1078, 921, 812, 730, 659. Anal. calcd for C_15_H_16_N_2_O_2_: C, 70.29; H, 6.29; N, 10.93. Found: C, 70.36; H, 6.34; N, 10.99.

(*R*)-2-(1*H*-imidazol-1-yl)-1-(3-methylbenzofuran-2-yl)ethan-1-ol (**15**)

Light yellow solid, 0.07 g, 66% yield, mp 119–121 °C, ${\left[\alpha \right]}_{D}^{25}$ − 5.40 (*c* 1.11, CHCl_3_); 98% ee, determined by HPLC analysis, Lux^®^ 5 μm Cellulose-4, LC column 250 × 4.6 mm, methanol/water 40:60, flow 0.8 mL/min, (*R*) 95.63 min, 99.20%, (*S*) 102.82 min, 0.80%. ^1^H NMR (400 MHz, CDCl_3_) δ (ppm): 2.10 (s, 3H, CH_3_), 4.26 (dd, 1H, *J* = 14.0 Hz, *J* = 5.2 Hz, CH_2_), 4.38 (dd, 1H, *J* = 14.0 Hz, *J* = 7.6 Hz, CH_2_), 5.06 (dd, 1H, *J* = 7.6 Hz, *J* = 5.2 Hz, CH), 5.90 (bs, 1H, OH), 6.82 (s, 1H, CH), 6.83 (s, 1H, CH), 7.24 (td, 1H, *J* = 7.2 Hz, *J* = 1.2 Hz, CH), 7.28–7.31 (m, 1H, CH), 7.32–7.33 (m, 1H, CH), 7.43–7.47 (m, 2H, 2xCH). ^13^C NMR (100 MHz, CDCl_3_) δ (ppm): 7.48 (CH_3_), 51.52 (CH_2_), 65.92 (CH), 110.11 (CH), 113.28 (C), 119.54 (CH), 119.75 (CH), 122.50 (CH), 124.59 (CH), 128.18 (CH), 129.70 (C), 137.44 (CH), 150.36 (C), 153.95 (C). (Figure S11 in the Supplementary Materials) IR neat (cm^−1^): 3122, 2854, 1511, 1455, 1317, 1231, 1181, 1068, 746, 660, 518. Anal. calcd for C_14_H_14_N_2_O_2_: C, 69.41; H, 5.82; N, 11.56. Found: C, 69.55; H, 5.97; N, 11.39.

(*R*)-1-(3,5-dimethylbenzofuran-2-yl)-2-(1*H*-imidazol-1-yl)ethan-1-ol (**16**)

Yellow solid, 0.08 g, 76% yield, mp 87–89 °C, ${\left[\alpha \right]}_{D}^{25}$ − 11.48 (*c* 1.48, CHCl_3_); 98% ee, determined by HPLC analysis, Lux^®^ 5 μm Cellulose-4, LC column 250 × 4.6 mm, methanol/water 45:55, flow 0.8 mL/min, (*R*) 121.34 min, 98.93%, (*S*) 138.93 min, 1.07%. ^1^H NMR (400 MHz, CDCl_3_) δ (ppm): 2.04 (s, 3H, CH_3_), 2.45 (s, 3H, CH_3_), 4.24 (dd, 1H, *J* = 14.4 Hz, *J* = 5.6 Hz, CH_2_), 4.35 (dd, 1H, *J* = 14.4 Hz, *J* = 7.6 Hz, CH_2_), 5.01 (dd, 1H, *J* = 7.6 Hz, *J* = 5.6 Hz, CH), 6.79 (s, 1H, CH), 6.82 (s, 1H, CH), 6.95 (bs, 1H, OH), 7.09 (dd, 1H, *J* = 8.4 Hz, *J* = 1.6 Hz, CH), 7.22 (s, 1H, CH), 7.29 (s, 1H, CH), 7.32 (d, 1H, *J* = 8.4 Hz, CH). ^13^C NMR (100 MHz, CDCl_3_) δ (ppm): 7.47 (CH_3_), 21.33 (CH_3_), 51.52 (CH_2_), 65.96 (CH), 110.61 (CH), 113.13 (C), 119.40 (CH), 119.82 (CH), 125.83 (CH), 128.03 (CH), 129.75 (C), 131.93 (C), 137.39 (CH), 150.30 (C), 152.38 (C). (Figure S12 in the Supplementary Materials) IR neat (cm^−1^): 3127, 2919, 2851, 1513, 1452, 1233, 1172, 1068, 800, 738, 592, 490. Anal. calcd for C_15_H_16_N_2_O_2_: C, 70.29; H, 6.29; N, 10.93. Found: C, 70.39; H, 6.25; N, 11.14.

(*R*)-1-(benzofuran-2-yl)-2-(1*H*-1,2,4-triazol-1-yl)ethan-1-ol (**17**)

Light yellow solid, 0.07 g, 71% yield, mp 57–59 °C, ${\left[\alpha \right]}_{D}^{23}$ + 72.73 (*c* 2.42, CHCl_3_); 96% ee, determined by HPLC analysis, Lux^®^ 5 μm i-Cellulose-5, LC column 250 × 4.6 mm, hexane/isopropanol 90:10, flow 0.7 mL/min, (*R*) 46.60 min, 98.10%, (*S*) 55.59 min, 1.90%. ^1^H NMR (400 MHz, CDCl_3_) δ (ppm): 4.47 (dd, 1H, *J* = 14.0 Hz, *J* = 8.4 Hz, CH_2_), 4.61 (dd, 1H, *J* = 14.0 Hz, *J* = 3.2 Hz, CH_2_), 5.23 (dd, 1H, *J* = 8.4 Hz, *J* = 3.5 Hz, CH), 5.70 (bs, 1H, OH), 6.66 (s, 1H, CH), 7.23 (td, 1H, *J* = 7.6 Hz, *J* = 1.2 Hz, CH), 7.28 (ddd, 1H, *J* = 8.0 Hz, *J* = 7.6 Hz, *J* = 1.2 Hz, CH), 7.45 (dq, 1H, *J* = 8.4 Hz, *J* = 0.8 Hz, CH), 7.54 (ddd, 1H, *J* = 7.6 Hz, *J* = 1.2 Hz, *J* = 0.8 Hz, CH), 7.79 (s, 1H, CH), 8.05 (s, 1H, CH). ^13^C NMR (100 MHz, CDCl_3_) δ (ppm): 54.14 (CH_2_), 66.50 (CH), 104.16 (CH), 111.30 (CH), 121.30 (CH), 123.10 (CH), 124.64 (CH), 127.78 (CH), 144.13 (C), 151.46 (CH), 154.89 (C), 155.62 (C). (Figure S13 in the Supplementary Materials) IR neat (cm^−1^): 3304, 3123, 3047, 2851, 1512, 1450, 1253, 1135, 1042, 872, 736, 675, 522, 499. Anal. calcd for C_12_H_11_N_3_O_2_: C, 62.87; H, 4.84; N, 18.33. Found: C, 63.04; H, 4.86; N, 18.39.

(*R*)-1-(7-ethylbenzofuran-2-yl)-2-(1*H*-1,2,4-triazol-1-yl)ethan-1-ol (**18**)

White solid, 0.08 g, 77% yield, mp 98–100 °C, ${\left[\alpha \right]}_{D}^{25}$ + 70.47 (*c* 2.10, CHCl_3_); 98% ee, determined by HPLC analysis, Lux^®^ 5 μm Amylose-1, LC column 250 × 4.6 mm, hexane/isopropanol 90:10, flow 0.7 mL/min, (*R*) 21.55 min, 99.22%, (*S*) 25.24 min, 0.78%. ^1^H NMR (400 MHz, CDCl_3_) δ (ppm): 1.37 (t, 3H, *J* = 7.6 Hz, CH_3_), 2.34 (bs, 1H, OH), 2.94 (q, 2H, *J* = 7.6 Hz, CH_2_), 4.63 (dd, 1H, *J* = 14.0 Hz, *J* = 7.6 Hz, CH_2_), 4.73 (dd, 1H, *J* = 14.0 Hz, *J* = 2.8 Hz, CH_2_), 5.33 (dd, 1H, *J* = 7.6 Hz, *J* = 2.8 Hz, CH), 6.71 (s, 1H, CH), 7.14 (ddd, 1H, *J* = 7.6 Hz, *J* = 1.2 Hz, *J* = 0.8 Hz, CH), 7.19 (t, 1H, *J* = 7.6 Hz, CH), 7.39 (dd, 1H, *J* = 7.2 Hz, *J* = 1.6 Hz, CH), 8.01 (s, 1H, CH), 8.25 (s, 1H, CH). ^13^C NMR (100 MHz, CDCl_3_) δ (ppm): 14.16 (CH_3_), 22.80 (CH_2_), 53.77 (CH_2_), 67.34 (CH), 104.65 (CH), 118.82 (CH), 123.31 (CH), 123.93 (CH), 127.36 (C), 127.78 (CH), 144.21 (C), 151.91 (CH), 153.52 (C), 154.60 (C). (Figure S14 in the Supplementary Materials) IR neat (cm^−1^): 3122, 2964, 2869, 1512, 1433, 1279, 1131, 1092, 953, 844, 729, 650. Anal. calcd for C_14_H_15_N_3_O_2_: C, 65.36; H, 5.88; N, 16.33. Found: C, 65.24; H, 5.68; N, 16.33.

(*R*)-1-(3-methylbenzofuran-2-yl)-2-(1*H*-1,2,4-triazol-1-yl)ethan-1-ol (**19**)

Light yellow solid, 0.06 g, 57% yield, mp 114–116 °C, ${\left[\alpha \right]}_{D}^{26}$ + 15.91 (*c* 2.64, CHCl_3_); 97% ee, determined by HPLC analysis, Lux^®^ 5 μm i-Cellulose-5, LC column 250 × 4.6 mm, hexane/isopropanol 90:10, flow 0.7 mL/min, (*R*) 50.88 min, 98.72%, (*S*) 59.31 min, 1.28%. ^1^H NMR (400 MHz, CDCl_3_) δ (ppm): 2.19 (s, 3H, CH_3_), 4.06 (bs, 1H, OH), 4.53 (dd, 1H, *J* = 13.6 Hz, *J* = 4.4 Hz, CH_2_), 4.68 (dd, 1H, *J* = 14.0 Hz, *J* = 8.4 Hz, CH_2_), 5.40 (dd, 1H, *J* = 8.4 Hz, *J* = 4.4 Hz, CH), 7.26 (td, 1H, *J* = 7.6 Hz, *J* = 0.8 Hz, CH), 7.32 (ddd, 1H, *J* = 8.4 Hz, *J* = 7.6 Hz, *J* = 1.2 Hz, CH), 7.45 (dt, 1H, *J* = 8.0 Hz, *J* = 0.8 Hz, CH), 7.49 (ddd, 1H, *J* = 7.6 Hz, *J* = 1.6 Hz, *J* = 0.8 Hz, CH), 7.91 (s, 1H, CH), 8.07 (s, 1H, CH). ^13^C NMR (100 MHz, CDCl_3_) δ (ppm): 7.66 (CH_3_), 53.69 (CH_2_), 64.99 (CH), 111.20 (CH), 113.97 (C), 119.74 (CH), 122.69 (CH), 124.98 (CH), 129.43 (C), 144.05 (CH), 148.93 (C), 151.74 (CH), 154.09 (C). (Figure S15 in the Supplementary Materials) IR neat (cm^−1^): 3196, 3131, 2865, 1513, 1457, 1274, 1130, 1041, 875, 748, 652. Anal. calcd for C_13_H_13_N_3_O_2_: C, 64.19; H, 5.39; N, 17.27. Found: C, 64.29; H, 5.46; N, 17.41.

(*R*)-1-(3,5-dimethylbenzofuran-2-yl)-2-(1*H*-1,2,4-triazol-1-yl)ethan-1-ol (**20**)

Yellow solid, 0.05 g, 48% yield, mp 107–110 °C, ${\left[\alpha \right]}_{D}^{26}$ + 4.93 (*c* 2.23, CHCl_3_); 97% ee, determined by HPLC analysis, Daicel Chiralcel OJ 10 μm, column 250 × 4.6 mm, hexane/isopropanol 90:10, flow 0.7 mL/min, (*R*) 50.44 min, 98.81%, (*S*) 56.87 min, 1.19%. ^1^H NMR (400 MHz, CDCl_3_) δ (ppm): 2.19 (s, 3H, CH_3_), 2.46 (s, 3H, CH_3_), 3.32 (bs, 1H, OH), 4.57 (dd, 1H, *J* = 14.0 Hz, *J* = 4.4 Hz, CH_2_), 4.77 (dd, 1H, *J* = 14.0 Hz, *J* = 8.0 Hz, CH_2_), 5.34 (dd, 1H, *J* = 8.0 Hz, *J* = 4.4 Hz, CH), 7.13 (ddd, 1H, *J* = 8.0 Hz, *J* = 1.6 Hz, *J* = 0.4 Hz, CH), 7.27 (t, 1H, *J* = 0.8 Hz, CH), 7.32 (d, 1H, *J* = 8.0 Hz, CH), 8.07 (s, 1H, CH), 8.47 (s, 1H, CH). ^13^C NMR (100 MHz, CDCl_3_) δ (ppm): 7.68 (CH_3_), 21.32 (CH_3_), 53.97 (CH_2_), 65.02 (CH), 110.70 (CH), 113.96 (C), 119.60 (CH), 126.27 (CH), 129.47 (C), 132.22 (C), 143.73 (CH), 148.64 (C), 150.35 (CH), 152.51 (C). (Figure S16 in the Supplementary Materials) IR neat (cm^−1^): 3173, 3115, 2958, 1515, 1459, 1256, 1139, 1061, 872, 797, 674, 496. Anal. calcd for C_14_H_15_N_3_O_2_: C, 65.36; H, 5.88; N, 16.33. Found: C, 65.44; H, 6.00; N, 16.40.

#### 2.3.3. General Procedure for α-Amino Ketones **5**–**12** Reduction by NaBH_4_

Sodium borohydride (0.068 g, 1.8 mmol) was added portion-wise to a solution of the corresponding benzofuryl *α*-amino ketone **5**–**12** (1.0 mmol) in methanol (5 mL) at 0 °C, over the period of 30 min. Next, the reaction mixture was stirred at room temperature for 24 h. The solvent was removed under reduced pressure, and the solid residue was suspended in water and extracted with dichloromethane. The organic layer was dried over anhydrous MgSO_4_ and the solvent was evaporated in vacuum. The crude product was purified by column chromatography on silica gel (hexane/ethyl acetate/methanol, 5:5:2).

1-(benzofuran-2-yl)-2-(1*H*-imidazol-1-yl)ethan-1-ol (**21**)

Orange solid, 0.22 g, 95% yield, mp 93–95 °C, lit. 92 °C [49], lit. 96 °C [50]. ^1^H NMR (400 MHz, CDCl_3_) δ (ppm): 4.32 (dd, 1H, *J* = 14.0 Hz, *J* = 7.2 Hz, CH_2_), 4.40 (bs, 1H, OH), 4.44 (dd, 1H, *J* = 14.0 Hz, *J* = 3.6 Hz, CH_2_), 5.11 (ddd, 1H, *J* = 7.6 Hz, *J* = 3.6 Hz, *J* = 0.8 Hz, CH), 6.73 (t, 1H, *J* = 0.8 Hz, CH), 6.88 (s, 1H, CH), 6.89 (s, 1H, CH), 7.25 (td, 1H, *J* = 7.6 Hz, *J* = 1.2 Hz, CH), 7.31 (ddd, 1H, *J* = 8.4 Hz, *J* = 7.2 Hz, *J* = 1.2 Hz, CH), 7.49 (dq, 1H, *J* = 8.4 Hz, *J* = 1.2 Hz, CH), 7.51 (s, 1H, CH), 7.56 (ddd, 1H, *J* = 7.6 Hz, *J* = 1.6 Hz, *J* = 0.8 Hz, CH). ^13^C NMR (100 MHz, CDCl_3_) δ (ppm): 52.12 (CH_2_), 67.70 (CH), 103.96 (CH), 111.19 (CH), 119.80 (CH), 121.23 (CH), 123.00 (CH), 124.38 (CH), 128.04 (C), 128.23 (CH), 137.59 (CH), 154.84 (C), 156.64 (C). (Figure S17 in the Supplementary Materials) IR neat (cm^−1^): 3108, 2996, 1679, 1451, 1285, 1167, 1079, 955, 805, 749, 643, 505, 428. Anal. calcd for C_13_H_12_N_2_O_2_: C, 68.41; H, 5.30; N, 12.27. Found: C, 68.55; H, 5.48; N, 12.34.

1-(7-ethylbenzofuran-2-yl)-2-(1*H*-imidazol-1-yl)ethan-1-ol (**22**)

Orange solid, 0.23 g, 93% yield, mp 78–81 °C. ^1^H NMR (700 MHz, CDCl_3_) δ (ppm): 1.36 (t, 3H, *J* = 7.0 Hz, CH_3_), 2.94 (q, 2H, *J* = 7.0 Hz, CH_2_), 3.67 (bs, 1H, OH), 4.31 (dd, 1H, *J* = 14.0 Hz, *J* = 7.0 Hz, CH_2_), 4.42 (dd, 1H, *J* = 14.0 Hz, *J* = 3.5 Hz, CH_2_), 5.10 (ddd, 1H, *J* = 7.0 Hz, *J* = 3.5 Hz, *J* = 0.7 Hz, CH), 6.70 (d, 1H, *J* = 0.7 Hz, CH), 6.86 (s, 1H, CH), 6.87 (s, 1H, CH), 7.12 (ddd, 1H, *J* = 7.7 Hz, *J* = 1.4 Hz, *J* = 0.7 Hz, CH), 7.17 (t, 1H, *J* = 7.7 Hz, CH), 7.37 (dd, 1H, *J* = 7.7 Hz, *J* = 1.4 Hz, CH), 7.47 (s, 1H, CH). ^13^C NMR (175 MHz, CDCl_3_) δ (ppm): 13.86 (CH_3_), 22.45 (CH_2_), 51.64 (CH_2_), 67.05 (CH), 103.74 (CH), 118.31 (CH), 119.59 (CH), 122.73 (CH), 123.12 (CH), 127.25 (C), 127.35 (C), 127.60 (CH), 137.17 (CH), 153.00 (C), 156.30 (C). (Figure S18 in the Supplementary Materials) IR neat (cm^−1^): 3111, 2966, 1509, 1425, 1232, 1178, 1078, 920, 809, 743, 659. Anal. calcd for C_15_H_16_N_2_O_2_: C, 70.29; H, 6.29; N, 10.93. Found: C, 70.47; H, 6.43; N, 11.11.

2-(1*H*-imidazol-1-yl)-1-(3-methylbenzofuran-2-yl)ethan-1-ol (**23**)

Brown oil, 0.17 g, 85% yield. ^1^H NMR (700 MHz, CDCl_3_) δ (ppm): 2.08 (s, 3H, CH_3_), 4.23 (dd, 1H, *J* = 14.0 Hz, *J* = 5.6 Hz, CH_2_), 4.35 (dd, 1H, *J* = 14.0 Hz, *J* = 7.7 Hz, CH_2_), 5.03 (dd, 1H, *J* = 7.7 Hz, *J* = 4.9 Hz, CH), 5.58 (bs, 1H, OH), 6.79 (s, 1H, CH), 6.80 (s, 1H, CH), 7.22 (td, 1H, *J* = 7.7 Hz, *J* = 0.7 Hz, CH), 7.27 (dd, 1H, *J* = 6.3 Hz, *J* = 1.4 Hz, CH), 7.29 (dd, 1H, *J* = 7.0 Hz, *J* = 1.4 Hz, CH), 7.42 (dt, 1H, *J* = 8.4 Hz, *J* = 0.7 Hz, CH), 7.44 (ddd, 1H, *J* = 7.7 Hz, *J* = 1.4 Hz, *J* = 0.7 Hz, CH). ^13^C NMR (175 MHz, CDCl_3_) δ (ppm): 7.06 (CH_3_), 51.01 (CH_2_), 65.35 (CH), 110.70 (CH), 112.87 (C), 119.14 (CH), 119.44 (CH), 122.09 (CH), 124.17 (CH), 127.60 (CH), 129.30 (C), 136.97 (CH), 150.08 (C), 153.53 (C). (Figure S19 in the Supplementary Materials) IR neat (cm^−1^): 3081, 2920, 1614, 1509, 1453, 1231, 1079, 922, 818, 729, 659, 522. Anal. calcd for C_14_H_14_N_2_O_2_: C, 69.41; H, 5.82; N, 11.56. Found: C, 69.39; H, 5.80; N, 11.67.

1-(3,5-dimethylbenzofuran-2-yl)-2-(1*H*-imidazol-1-yl)ethan-1-ol (**24**)

Light yellow solid, 0.18 g, 88% yield, mp 123–125 °C. ^1^H NMR (700 MHz, CDCl_3_) δ (ppm): 2.04 (s, 3H, CH_3_), 2.43 (s, 3H, CH_3_), 4.22 (dd, 1H, *J* = 14.0 Hz, *J* = 5.6 Hz, CH_2_), 4.33 (dd, 1H, *J* = 14.0 Hz, *J* = 7.7 Hz, CH_2_), 5.00 (dd, 1H, *J* = 7.7 Hz, *J* = 5.6 Hz, CH), 5.93 (bs, 1H, OH), 6.77 (s, 1H, CH), 6.80 (s, 1H, CH), 7.08 (ddd, 1H, *J* = 8.4 Hz, *J* = 2.1 Hz, *J* = 0.7 Hz, CH), 7.21 (t, 1H, *J* = 0.7 Hz, CH), 7.27 (s, 1H, CH), 7.29 (d, 1H, *J* = 8.4 Hz, CH). ^13^C NMR (100 MHz, CDCl_3_) δ (ppm): 7.56 (CH_3_), 21.33 (CH_3_), 51.46 (CH_2_), 66.34 (CH), 110.66 (CH), 113.45 (C), 119.48 (CH), 119.63 (CH), 126.02 (CH), 128.75 (CH), 129.65 (C), 132.08 (C), 137.59 (CH), 149.73 (C), 152.41 (C). (Figure S20 in the Supplementary Materials) IR neat (cm^−1^): 3120, 2918, 1590, 1510, 1414, 1258, 1050, 968, 809, 736, 626, 425. Anal. calcd for C_15_H_16_N_2_O_2_: C, 70.29; H, 6.29; N, 10.93. Found: C, 70.35; H, 6.36; N, 10.96.

1-(benzofuran-2-yl)-2-(1*H*-1,2,4-triazol-1-yl)ethan-1-ol (**25**)

White solid, 0.18 g, 88% yield, mp 78–80 °C, lit. 73 °C [49]. ^1^H NMR (700 MHz, CDCl_3_) δ (ppm): 4.22 (bs, 1H, OH), 4.56 (dd, 1H, *J* = 14.0 Hz, *J* = 7.7 Hz, CH_2_), 4.65 (dd, 1H, *J* = 14.0 Hz, *J* = 3.5 Hz, CH_2_), 5.27 (dd, 1H, *J* = 7.7 Hz, *J* = 3.5 Hz, CH), 6.68 (t, 1H, *J* = 0.7 Hz, CH), 7.23 (ddd, 1H, *J* = 7.7 Hz, *J* = 7.0 Hz, *J* = 0.7 Hz, CH), 7.29 (ddd, 1H, *J* = 8.4 Hz, *J* = 7.0 Hz, *J* = 1.4 Hz, CH), 7.46 (dq, 1H, *J* = 8.4 Hz, *J* = 1.4 Hz, CH), 7.54 (ddd, 1H, *J* = 7.7 Hz, *J* = 1.4 Hz, *J* = 0.7 Hz, CH), 7.89 (s, 1H, CH), 8.06 (s, 1H, CH). ^13^C NMR (175 MHz, CDCl_3_) δ (ppm): 53.55 (CH_2_), 66.33 (CH), 103.84 (CH), 110.89 (CH), 120.91 (CH), 122.72 (CH), 124.26 (CH), 127.36 (CH), 143.70 (C), 151.28 (CH), 154.50 (C), 155.04 (C). (Figure S21 in the Supplementary Materials) IR neat (cm^−1^): 3208, 3106, 2943, 2869, 1515, 1453, 1246, 1141, 1079, 937, 676, 428. Anal. calcd for C_12_H_11_N_3_O_2_: C, 62.87; H, 4.84; N, 18.33. Found: C, 62.96; H, 4.92; N, 18.48.

1-(7-ethylbenzofuran-2-yl)-2-(1*H*-1,2,4-triazol-1-yl)ethan-1-ol (**26**)

White solid, 0.18 g, 91% yield, mp 88–90 °C. ^1^H NMR (700 MHz, CDCl_3_) δ (ppm): 1.34 (t, 3H, *J* = 7.7 Hz, CH_3_), 2.92 (q, 2H, *J* = 7.7 Hz, CH_2_), 3.31 (bs, 1H, OH), 4.58 (dd, 1H, *J* = 14.0 Hz, *J* = 7.7 Hz, CH_2_), 4.68 (dd, 1H, *J* = 14.0 Hz, *J* = 2.8 Hz, CH_2_), 5.29 (ddd, 1H, *J* = 7.7 Hz, *J* = 3.5 Hz, *J* = 0.7 Hz, CH), 6.69 (d, 1H, *J* = 0.7 Hz, CH), 7.12 (ddd, 1H, *J* = 7.0 Hz, *J* = 1.4 Hz, *J* = 0.7 Hz, CH), 7.16 (t, 1H, *J* = 7.7 Hz, CH), 7.37 (dd, 1H, *J* = 7.7 Hz, *J* = 1.4 Hz, CH), 7.95 (s, 1H, CH), 8.17 (s, 1H, CH). ^13^C NMR (100 MHz, CDCl_3_) δ (ppm): 14.14 (CH_3_), 22.78 (CH_2_), 54.05 (CH_2_), 66.92 (CH), 104.46 (CH), 118.78 (CH), 123.28 (CH), 123.85 (CH), 127.40 (C), 127.78 (CH), 144.10 (C), 151.71 (CH), 153.51 (C), 155.03 (C). (Figure S22 in the Supplementary Materials) IR neat (cm^−1^): 3120, 2964, 2890, 1513, 1433, 1278, 1131, 1090, 953, 740, 649, 545. Anal. calcd for C_14_H_15_N_3_O_2_: C, 65.36; H, 5.88; N, 16.33. Found: C, 65.50; H, 5.81; N, 16.42.

1-(3-methylbenzofuran-2-yl)-2-(1*H*-1,2,4-triazol-1-yl)ethan-1-ol (**27**)

White solid, 0.15 g, 87% yield, mp 115–118 °C. ^1^H NMR (700 MHz, CDCl_3_) δ (ppm): 2.17 (s, 3H, CH_3_), 3.73 (bs, 1H, OH), 4.51 (dd, 1H, *J* = 14.0 Hz, *J* = 4.2 Hz, CH_2_), 4.68 (dd, 1H, *J* = 14.0 Hz, *J* = 8.4 Hz, CH_2_), 5.32 (dd, 1H, *J* = 8.4 Hz, *J* = 4.2 Hz, CH), 7.23–7.25 (m, 1H, CH), 7.30 (td, 1H, *J* = 8.4 Hz, *J* = 1.4 Hz, CH), 7.43 (dd, 1H, *J* = 7.7 Hz, *J* = 1.4 Hz, CH), 7.47 (dd, 1H, *J* = 7.7 Hz, *J* = 0.7 Hz, CH), 7.90 (s, 1H, CH), 8.04 (s, 1H, CH). ^13^C NMR (175 MHz, CDCl_3_) δ (ppm): 7.20 (CH_3_), 53.28 (CH_2_), 64.19 (CH), 110.77 (CH), 113.37 (C), 119.31 (CH), 122.26 (CH), 124.52 (CH), 129.02 (C), 143.65 (CH), 148.84 (C), 151.17 (CH), 153.65 (C). (Figure S23 in the Supplementary Materials) IR neat (cm^−1^): 3136, 2868, 1513, 1457, 1276, 1135, 1086, 970, 880, 748, 652, 530. Anal. calcd for C_13_H_13_N_3_O_2_: C, 64.19; H, 5.39; N, 17.27. Found: C, 64.35; H, 5.52; N, 17.29.

1-(3,5-dimethylbenzofuran-2-yl)-2-(1*H*-1,2,4-triazol-1-yl)ethan-1-ol (**28**)

White solid, 0.12 g, 64% yield, mp 113–114 °C. ^1^H NMR (400 MHz, CDCl_3_) δ (ppm): 2.13 (s, 3H, CH_3_), 2.45 (s, 3H, CH_3_), 4.37 (bs, 1H, OH), 4.49 (dd, 1H, *J* = 14.0 Hz, *J* = 4.8 Hz, CH_2_), 4.65 (dd, 1H, *J* = 14.0 Hz, *J* = 8.0 Hz, CH_2_), 5.29 (dd, 1H, *J* = 8.0 Hz, *J* = 4.8 Hz, CH), 7.12 (dd, 1H, *J* = 4.8 Hz, *J* = 1.6 Hz, CH), 7.25 (t, 1H, *J* = 0.8 Hz, CH), 7.31 (d, 1H, *J* = 8.4 Hz, CH), 7.87 (s, 1H, CH), 8.00 (s, 1H, CH). ^13^C NMR (100 MHz, CDCl_3_) δ (ppm): 7.56 (CH_3_), 21.32 (CH_3_), 53.66 (CH_2_), 64.56 (CH), 110.66 (CH), 113.50 (C), 119.54 (CH), 126.12 (CH), 129.49 (C), 132.10 (C), 144.04 (CH), 149.39 (C), 151.53 (CH), 152.46 (C). (Figure S24 in the Supplementary Materials) IR neat (cm^−1^): 3179, 3100, 2877, 1521, 1474, 1254, 1144, 1066, 889, 797, 575, 502. Anal. calcd for C_14_H_15_N_3_O_2_: C, 65.36; H, 5.88; N, 16.33. Found: C, 65.57; H, 5.97; N, 16.41.

#### 2.3.4. General Procedure for the Asymmetric Reduction of α-Amino Ketones **6**, **9** and **12** by Saccharomyces Cerevisiae

A suspension of baker’s yeast (1 g) in 14.5 mL potassium phosphate buffer (pH = 7.0) and 0.07 g (3.8 × 10^−4^ mol) glucose was put into a 40 mL Falcon tube. The resulting mixture was stirred on a rotary shaker at 350 revolutions per minute (RPM) at 30 °C. After the fermentation step (0.5 h), a solution of ketone (5 × 10^−4^ mol in 0.5 mL EtOH) was added. The reaction progress was monitored by TLC. After 24 h, the yeast cells were removed by filtration using a Celite filter. The yeast cells were washed with 2 × 25 mL water. The filtrate was saturated with NaCl and then extracted with 5 × 20 mL portions of ethyl acetate. The organic layer was dried over anhydrous MgSO_4_, and the solvent was evaporated in vacuum. The crude product was purified by TLC (hexane/ethyl acetate/methanol, 5:5:2). The NMR spectra of the products **14**, **17**, and **20** and the HPLC analysis conditions were the same as described in Section 2.3.2.

#### 2.3.5. General Procedure for the Asymmetric Reduction of α-Amino Ketones **6**, **9** and **12** by Aureobasidium Pullulans without Additives

The biotransformation reaction was preceded with half an hour pre-incubation on a rotary shaker at 350 revolutions per minute (RPM) at 24 or 30 °C. The pre-incubation mixture composition was as follows: 0.07 g glucose (3.8 × 10^−4^ mol), 1 g Boni Protect, 14.5 mL phosphate buffer (pH = 7.0). After pre-incubation, 5 × 10^−4^ moles of the substrate dissolved in 0.5 mL of ethanol were added to the mixture. After the reaction was completed, the mixture was filtered. The filtrate and the solid phase were then extracted with ethyl acetate (4 × 25 mL) and the organic layer was evaporated. The crude product was purified by TLC (hexane/ethyl acetate/methanol, 5:5:2). The NMR spectra of the products **14**, **17**, and **20** and the HPLC analysis conditions were the same as described in Section 2.3.2.

#### 2.3.6. General Procedure for the Asymmetric Reduction of α-Amino Ketones **6**, **9** and **12** by Aureobasidium Pullulans with Additives

In the biotransformation reaction, 0.07 g glucose (3.8 × 10^−4^ mol) was added to a suspension of 1 g Boni Protect in 14.5 mL phosphate buffer (pH = 7.0), and the resulting suspension was stirred on a rotary shaker at 350 RPM at 24, 27 or 30 °C. An additive compound (1.25 × 10^−5^ mol ethyl (9-antryl)glyoxylate (AMA-1)/allyl alcohol/ethyl chloroacetate/cysteine) and a substrate (5×10^−4^ mol in 0.5 mL of ethanol) were then added and stirring was continued at the same temperature. After the reaction was completed, the mixture was filtered. The filtrate and the solid phase were then extracted with ethyl acetate (4 × 25 mL), and the organic layer was evaporated. The crude product was purified by TLC (hexane/ethyl acetate/methanol, 5:5:2). The NMR spectra of the products **14**, **17**, and **20** and the HPLC analysis conditions were the same as described in Section 2.3.2.

### 2.4. Antibacterial and Antifungal Studies of Racemic and Chiral Benzofuryl Derivatives

Susceptibility tests of *β*-amino alcohols **13**–**28** and ketones **1**–**12** were performed against Gram-negative (*Escherichia coli* ATCC 8739 and *Escherichia coli* ATCC 25922) and Gram-positive (*Staphylococcus aureus* ATCC 6538 and *Staphylococcus aureus* ATCC 25923) bacteria, and against yeast *viz*., *Candida albicans* ATCC 10231 and *Malassezia furfur* DSM 6170. Microbial strains were obtained from American Type Culture Collection (ATCC; Manassas, Virginia, USA) or Leibniz Institute DSMZ-German Collection of Microorganisms and Cell Cultures GmbH (Braunschweig, Germany), respectively. The assays were performed in 96-well plates using the standard fold broth microdilution method of the benzofuryl derivatives in trypticase soy broth (TSB; Becton Dickinson and Company, Franklin Lakes, NJ, USA) for bacteria or Sabouraud Dextrose Broth (SDB; Becton Dickinson and Company, Franklin Lakes, NJ, USA) for yeast, following Clinical and Laboratory Standards Institute (CLSI) guidelines [51]. The SDB medium supplemented with olive oil (2.5 mL L^−1^) was used to enhance the growth of *M. furfur*. The final concentration, 5 × 10^5^ c.f.u per mL of bacteria or yeast, was maintained in each well of the multi-well plate. The tested concentration range of the benzofuryl derivatives was from 0.016 to 512 μg mL^−1^. Both positive (broth mixed with microbial inoculum) and negative (sterile non-inoculated broth) controls were also maintained. The inoculated plates were incubated at 37 °C for 24 h (the bacteria and *C. albicans*) or for 48 h (*M. furfur*). The MIC value was defined as the lowest concentration of the benzofuryl derivative which inhibited the growth of bacteria and yeast (estimated visually). The minimum biocidal (bactericidal, fungicidal) concentrations (MBCs) of the benzofuryl derivatives were also determined. After incubation, 100 μL of each test sample was aseptically spread onto the agar medium (TSA for bacteria, SDA for *C. albicans*, SDA with olive oil for *M. furfur*) on Petri plates. The plates were incubated at 37 °C for 24 or 48 h, respectively, and microbial growth was observed. MBC was defined as the lowest concentration of the benzofuryl derivative that inhibited the growth of >99.9% microbial cells. All the assays were performed in triplicate.

## 3. Results and Discussion

### 3.1. Synthesis and Reduction of Novel Benzofuryl α-Azole Ketones

**Scheme 1:**
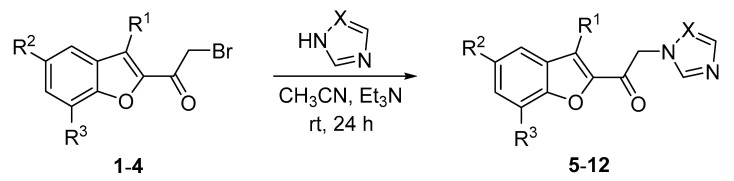
Synthesis of benzofuryl *α*-azole ketones **5**–**12**.

The first step of this study involved the synthesis of eight differently substituted benzofuryl *α*-amino ketones **5**–**12**, the azole derivatives. The compounds were obtained by the corresponding 1-(benzofuran-2-yl)-2-bromoethanones **1**–**4** and 1*H*-imidazole or 1*H*-1,2,3-triazole condensation according to the known procedure [49]. The reactions were carried out in acetonitrile, in the presence of triethylamine, at room temperature, for 24 h, and the products **5**–**12** were separated in moderate to good yields (42–83%) (Scheme 1).

**Scheme 2:**
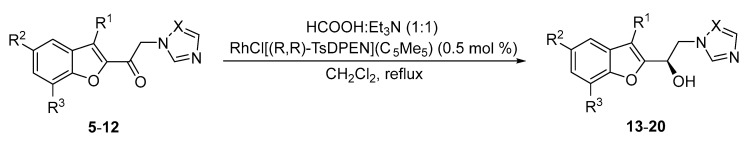
Asymmetric transfer hydrogenation of benzofuryl *α*-azole ketones **5**–**12**.

In our previously published research, we have proved that the asymmetric transfer hydrogenation (ATH) is an excellent, highly enantioselective method for the reduction of various benzofuryl ketones [42,43,44,46]. Moreover, this method has also been successfully used for the reduction of 2-(1*H*-imidazol-1-yl)-1-phenylethanones [26,27,28,52]. Therefore, we have decided to use the asymmetric transfer hydrogenation for the reduction of new benzofuryl *α*-azole ketones **5**−**12**. When our standard conditions for the ATH (a chiral Rh(III)/TsDPEN complex as the catalyst, the formic acid/triethylamine azeotrope (5:2), ethyl acetate as the solvent, at room temperature) were applied; no desired products were obtained. Thus, after conditions optimization, the reduction was carried out with HCOOH as a hydrogen resource in the presence of Et_3_N in the 1:1 ratio, catalyzed by RhCl[(*R*,*R*)-TsDPEN](C_5_Me_5_), in dichloromethane, at reflux for 24 h (Scheme 2). However, longer reaction time (48 h) was required for the triazole **9**–**12** derivatives hydrogenation. The reactions progress was monitored by the TLC analysis.

The corresponding optically active *β*-amino alcohols **13**–**20** were obtained in good yields (48–79%) and excellent enantioselectivities (96–99%), as determined by the HPLC analysis on the chiral columns. The imidazole derivatives **5**–**8** were reduced with selectivity slightly higher than the triazole ketone **9**–**12**. The highest enantioselectivity (99%) was achieved in the reduction of 1-(benzofuran-2-yl)-2-(1*H*-imidazol-1-yl)ethanone (**5**). To the best of our knowledge, no chiral benzofuryl *β*-amino alcohols bearing the azole rings have been reported in the literature.

**Scheme 3:**
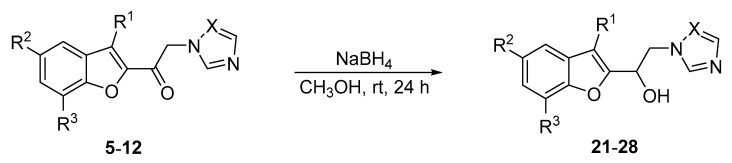
Reduction of benzofuryl *α*-azole ketones **5–12** by NaBH_4_.

The appropriate racemic *β*-amino alcohols **21**–**28** were also prepared by ketones reduction using NaBH_4_ under standard conditions (Scheme 3) [49]. The reduction reactions were carried out in methanol, at room temperature, for 24 h, and the products were separated in high yields (64–95%). These compounds were necessary as standards for the ee determination.

Due to the fact that, as an asymmetric reduction method, bioreduction is also in our field of interest [53,54,55], we decided to check the possibilities of the benzofuryl *α*-amino ketones reduction with the fungi of *Saccharomyces cerevisiae* and *Aureobasidium pullulans* species. Reduction with the use of baker’s yeast (*Saccharomyces cerevisiae*) is the best known and commonly used method of the microbiological chiral reduction [56]. In turn, the Boni Protect^®^ antimycotic agent containing living cells of *Aureobasidium pullulans* showed unusual catalytic abilities in the enantioselective reduction of various carbonyl compounds [53,55]. Three *α*-azole ketones, 1-(7-ethylbenzofuran-2-yl)-2-(1*H*-imidazol-1-yl)ethanone (**6**), 1-(benzofuran-2-yl)-2-(1*H*-1,2,4-triazol-1-yl)ethanone (**9**), and 1-(3,5-dimethylbenzofuran-2-yl)-2-(1*H*-1,2,4-triazol-1-yl)ethanone (**12**) were selected for the microbiological reduction with the aforementioned reagents and the results are summarized in Table 1.

The optimization of reaction conditions was carried out for each of the ketones. The microbiological reduction was carried out in an aqueous medium using a phosphate buffer solution of pH = 7 in the presence of glucose as the energy source. The bioreduction with the use of *A. pullulans* was performed without and with additional substances (ethyl (9-antryl)glyoxylate (AMA-1), allyl alcohol, ethyl chloroacetate, cysteine) to improve enantioselectivity, i.e., additives which inhibit oxidoreductases (present in *A. pullulans*) with a specific stereopreference [53,55]. The reduction of *α*-imidazole ketone **6** catalyzed by *A. pullulans* in the presence of cysteine gave the best results. The *β*-amino alcohol **14** was formed in 79% yield and with 99% ee of the *S* configuration, while the reduction without the additive gave the product with the lowest enantioselectivity (59%). In contrast, bio-reduction by baker’s yeast allowed for the formation of the (*R*)-product **14** in 89% yield and with 86% ee. In the case of *α*-triazole ketones **9** and **12**, the highest efficiency and enantioselectivity were observed in the case of reduction catalyzed by baker’s yeast. The corresponding (*R*)-alcohols **17** and **20** were obtained with 89 and 99% ee, respectively. The reduction of ketone **9** in the presence of *A. pullulans* afforded to (*S*)-product **17**. With no additive, the alcohol **17** was obtained only with 10% ee, whereas the use of cysteine allowed the formation of **17** with 50% ee. In turn, the bioreduction of ketone **12** catalyzed by *A. pullulans* led to the alcohol **20** of the *R* configuration. In this case, the use of additional substances resulted in a decrease in enantioselectivity (32–38%) and poor yield (13–33%). On the other hand, the reduction of **12** without the additive gave the (*R*)-product **20** with 51–% enantiomeric excess and 65–% yield. Based on these results, it can be concluded that the microbiological reduction, besides the transfer hydrogenation, is also a convenient method for the asymmetric reduction of benzofuryl *α*-azole ketones. However, the choice of bioreduction conditions depends on a ketone structure.

When the two methods of asymmetric ketone reduction discussed above are compared, i.e., transfer hydrogenation (ATH) and bioreduction, their advantages as well as disadvantages can be mentioned. For example, bioreduction is an ecological (reaction in water, with a small addition of organic solvents) and economical (cheap microorganisms, their high availability) method. Unfortunately, the fact that the reduction conditions must be selected separately for each substrate, which in the case of a series of compounds, extends the research time, is thus its biggest disadvantage. On the other hand, transfer hydrogenation is a method that uses costly catalysts containing metal atoms (in this case: rhodium), but once optimized, the reaction conditions allow for a rapid and simple reduction of many compounds of a given type.

### 3.2. Determination of the Absolute Configuration of Chiral Benzofuryl β-Amino Alcohols

Within the last decades, lots of correlation rules binding the sign of the Cotton effect (CE, the main quality of ECD spectrum) or the sign of optical rotation with the structure of an analyte were proposed [33]. We, as well as other authors, demonstrated that most of the traditional empirical correlation rules are inadequate and often incorrect and may lead to wrong conclusions [57,58,59]. Solving the Rosenfeld equation of optically active electron transitions, with the use of modern theoretical methods, mostly the DFT-based one is an alternative to the empirical rules [60,61,62,63,64,65].

**Figure 3:**
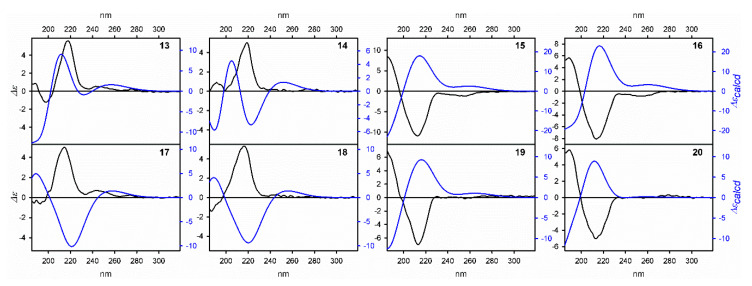
ECD spectra of **13**–**20**, experimental, measured in acetonitrile (black lines) and calculated for (*S*)-**13**-(*S*)-**20** at the IEFPCM/TD-ωB97-XD/6-311++G(2d,2p) level, *ΔΔG*-based and Boltzmann averaged (blue lines). The calculated spectra were wavelength corrected to match the UV maxima.

Therefore, to shed light on the stereochemistry of the products **13**–**20**, we have performed some experimental and theoretical studies. These included, on the one hand, experimental measurements of the ECD spectra and optical rotations, and theoretical simulations of the ECD curves and ORs values, respectively, on the other hand. Since the compounds under study were non-soluble in non-polar hydrocarbon solvents, the ECD spectra were thus measured in acetonitrile (Figure 3). The ORs values were obtained for chloroform solutions of respective alcohols, and at four wavelengths, namely: 589, 578, 546 and 436 nm.

The theoretical approach to the absolute configuration determination was based on a well-established protocol which was successfully applied by us and others to solve stereochemical problems [66]. The detailed theoretical procedure was described in the Supplementary Materials section and will only be briefly summarized here.

After the systematic conformational search at the molecular mechanics level, all the conformers of the assumed *S* absolute configuration at the stereogenic center were pre-optimized in parallel with the use of a B3LYP hybrid functional, small basis set 6–31G(d) and the IEFPCM solvent model, respectively, for chloroform and acetonitrile [67,68,69,70,71]. After the duplicates were removed, all the pre-optimized structures were fully optimized at the IEFPCM/B3LYP/6-311++G(d,p) level, again independently for the chloroform and acetonitrile solvent models. The frequency calculations showing that the structures thus obtained were real minimum energy conformers were confirmed. The Gibbs free energy values were used to obtain the Boltzmann population of the conformers. Only the conformers ranging in the free energy values from 0 to 2 kcal mol^−1^ were taken for further considerations. The detailed results of these calculations were juxtaposed in Tables S1–S16 (see the Supplementary Materials). The ECD spectra were calculated with the use of three different hybrid functionals, namely CAM-B3LYP [72], M06-2X [73], and *ω*B97-XD [74] (see Figures S25–S72 in the Supplementary Materials), all in the conjunction with the enhanced 6-311++G(2d,2p) basis set and the IEFPCM solvent model of acetonitrile. Finally, the calculated ECD spectra were Boltzmann averaged and compared with the experimental ones. Since there are no significant differences between the TD-DFT calculations results, in this work, we discussed the results obtained with the use of the IEFPCM/TD-*ω*B97-XD/6-311++G(2d,2p) method. In a similar way, the ORs calculations were carried out. However, in such calculations, we limited the scope of our work to the IEFPCM/B3LYP/*aug*-cc-pVTZ method only. The ORs values calculated for each individual low-energy conformer of **13**–**20** have been juxtaposed in Tables S17–S24 in the Supplementary Materials.

Despite apparent simplicity, the compounds under study are characterized by complex conformational dynamics. In the simplest case out of **13**, there are four torsional angles, whose change led to observing 21 thermally accessible conformers. The *α* angle defines the spatial relationship between the oxygen atoms (*α* = O–C–C*–O), the *β* angle describes the conformation of the hydroxyl group (*β* = (O)C–C*–O–H). The *γ* angle, defined here as *γ* = (O)C–C*–C–N, indicates the mutual orientation of the both aromatic groups, whereas the *δ* angle describes the orientation of the imidazole or triazole moiety with respect to the hydroxyl group.

In general, a large number of low-energy species participating in equilibrium makes none of them dominant. In the case of the above-mentioned alcohol **13**, the proportion of the *ΔΔG*-based lowest energy conformer No 29 in the population is 16%, whereas the second low-energy conformer No 53 is only slightly less populated (15%). With an exception of the conformer No 14 whose population in equilibrium is 11%, the Boltzmann population of any other conformer does not exceed 10%.

**Figure 4:**
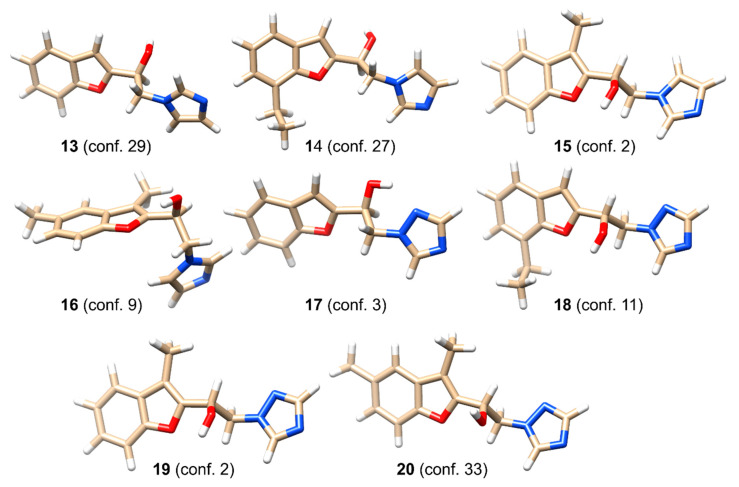
Structures of the *ΔΔG*-based lowest energy conformers of (*S*)-**13**-(*S*)-**20**, calculated at the IEFPCM/B3LYP/6-311++G(d,p) level.

The number of thermally accessible conformers increases when the ethyl group is attached to the benzofuran skeleton. In the extreme case of **14**, the total number of thermally accessible conformers reached the value of 36, and the most abundant conformer No 27 has 12% equilibrium share. The number of conformers is associated with an additional degree of freedom which is rotation around the C-C bond in the ethyl group. The opposite effect, a reduction of the number of thermally accessible conformers is visible for the compounds having the methyl group the in C3 position of benzofuran and/or the triazole ring constitutes a part of the molecule. Unexpectedly, conformation of the lowest energy conformers of **13**−**20** (shown in Figure 4) is not determined by possible hydrogen bonding interactions. To be precise, the formation of the O–H···O or O–H···N hydrogen bonds is one of the factors that may affect conformation, but not the most important one. In majority of cases, the aryl groups are *antiperiplanarly*-oriented, which is associated with the extended conformation of the *γ* angles. The lowest energy conformer of the alcohol **16**, where the *γ* angle adapts the *gauche* conformation, is an exception. The *anti*-orientation of the oxygen atoms is preferred unless steric repulsion involving the methyl group attached to the C3 position of the benzofuran ring causes change of the *α* angle conformation from *anti* to (-)-*synclinal*.

In the case of the lowest energy conformers of **15**, **16**, **18** and **19**, the formation of the OH···O hydrogen bonding is possible. However, the OH···O distances that range from 2.611 to 2.695 Å indicate the weakness of this hydrogen bonding. The six-membered O–H···N hydrogen bonding was found in the lowest energy conformer No 3 of **17**. In this particular case, the O–H···N distance is 2.019 Å which suggests greater strength of this hydrogen bond if compared to the one previously discussed. It is worth noting that the change of environment from polar acetonitrile to less polar chloroform did not alter significantly the conformational preferences of the compounds under study. This suggests that the conformation of the alcohols **13**–**20** is controlled mostly by the sterical interactions and to a lesser extent by repulsive or attractive electrostatic interactions between O, N and H atoms.

In the next stage of the analysis, the calculations of the ECD spectra and the OR values have been performed, and thus, the theoretical results obtained were compared with the experimental data. The comparison of the experimental and the *ΔΔG*-based and Boltzmann averaged ECD spectra is shown in Figure 3, whereas the experimental and calculated ORs values have been juxtaposed in Table 2. One should keep in mind that all the calculations have been carried out for alcohols having the absolute *S* configuration at the stereogenic center.

The UV spectra of 13−20 (shown in Supplementary Information) are characterized by two pronounced absorption bands. The low-energy absorption bands appear at around 245 nm (ε ≈ 10000) and are associated with very weak ECD bands (|Δε| ranging from 0.3 to 3). The more intense UV bands are observed at around 205 nm (ε ≈ 30000) and are associated with much more intense CEs appearing in the same spectral region (|Δε| ranging from 5 to 10). The third CEs are seen at a higher energy region (ca. 190 nm) on the slope of the main UV band. It should be noted that there are no direct correlations of the observed CEs with the stereochemistry of the given compound. Assuming the same absolute configuration at the stereogenic center in each of the compounds, we can draw contradictory conclusions. Thus, the application of the DFT calculations may be useful in those ambiguous cases.

At first glance, it appears that the calculated ECD spectra almost perfectly mirrored the experimental ones, with the exception of **13**. In this particular case, a good overall agreement is visible between the experimental and the theoretical spectrum. Especially, the theoretical results reflect well the low-energy region, hardly noticeable in some cases. Therefore, from the experimental and theoretical ECD data, we may conclude that the alcohols obtained through the ATH reaction are characterized by the absolute *R* configuration at the stereogenic center, with the exception of **13**. However, the reason for this discrepancy is currently not obvious to us.

Even the cursory reading of the data from Table 2 allowed observing similar dependencies. Noticeably, the presence of the methyl group within the benzofuran skeleton significantly decreases the measured OR values.

However, more attention should be paid to more demanding cases, namely **19** and **20**. The OR values of **19** and **20** found experimentally were very small, regardless of the wavelength. Thus, in these cases, the reliability of the stereochemical assignments which were based solely on an optical rotation is questionable. Fortunately, the second method, the ECD, functions well in these cases and unequivocally confirms the *R* configuration at the stereogenic center in the real compounds.

### 3.3. Antibacterial and Antifungal Activities of Racemic and Chiral Benzofuryl Derivatives

The in vitro antibacterial and antifungal activities of all the prepared benzofuran derivatives were evaluated against four bacterial isolates, namely *E. coli* ATCC 25922, *E. coli* ATCC 8739, *S. aureus* ATCC 25923, *S. aureus* ATCC 6538, and two fungal strains, *viz. C. albicans* ATCC 10231 and *M. furfur* DSM 6170. The obtained data, expressed as the mean of the minimal inhibitory concentration (MIC) and the minimal biocidal concentration (MBC) values are reported in Table 3 and Table 4. The MIC and MBC values were determined using the standard broth dilution technique [51]. In addition, commercially used antimicrobial compounds were included into study for comparative analyses (Table 5).

Generally, susceptibility of bacteria and fungi was highest to *α*-bromo ketones **1**–**4**, followed by the *β*-amino alcohols **13**–**28** and the *α*-amino ketones **5**–**12**. From among the tested bacteria, higher sensitivity was found in the Gram-positive (*S. aureus*) rather than the Gram-negative (*E. coli*) ones, while among yeast, in *M. furfur* rather than *C. albicans*. As can be seen from Table 3, among the tested amino alcohols, (*R*)-**16** showed the highest antimicrobial activity against *S. aureus* ATCC 25923 (MIC = 64, MBC = 96 μg mL^−1^) and (*R*)-**20**—against yeasts of *M. furfur* DSM 6170 (MIC = MBC = 64 μg mL^−1^). Interestingly, the yeast of *M. furfur* DSM 6170 was found to be susceptible to most of the studied amino alcohols (MIC = 64–256 μg mL^−1^), apart from (*R*)-**14**. From among the racemic *β*-amino alcohols, the compound **26** exhibited the highest activity against *M. furfur* (MIC = MBC = 128 μg mL^−1^). The MICs evaluated for these amino alcohols against *M. furfur* were equal to the MBC values (Table 3). The fungicidal activity of the new synthesized chiral alcohol with the triazole ring, i.e., (*R*)-**20**, is similar to that found in a commercially available antifungal agent, fluconazole, (MBC = 96 μg mL^−1^, Table 5) which is also a triazole derivative. The antifungal activity of most of the remaining amino alcohols possessing imidazole and triazole groups clearly showed activity higher than those of standard antifungal agents of amphotericin B and ketoconazole against the both yeast strains of *M. furfur* and *C. albicans* (Table 5). Interestingly, the use of the both antifungal agents mentioned above in the treatment of fungal diseases by the oral administration is limited due to their well-known side effects and toxicity [75,76]. Overall, (*R*)-**14** was not active in the tested concentration range (0.016–512 μg mL^−1^) whereas the *β*-amino alcohol **23** was not tested due to lack of solubility in water, and precipitation in low ethanol or DMSO (dimethyl sulfoxide) concentration required to be non-toxic to microbial cells (Table 3).

From among the tested ketones, the highest antimicrobial activity was found for 1-(benzofuran-2-yl)-2-bromoethanones (**1**–**4**), notably against *M. furfur* (MIC = MBC = 1.5 μg mL^−1^) and the strains of *S. aureus* (MIC = 16–256 μg mL^−1^) and α-amino ketone **12** against all the studied microorganisms (MIC = 128–512 and MBC = 256–512 μg mL^−1^). The susceptibility of Gram-negative bacteria and yeast of the *C. albicans* type to these ketones was lower (MIC and MBC from 64 to >512 μg mL^−1^). In turn, the α-amino ketone **8** showed activity against *M. furfur* (MIC = 128 and MBC = 256 μg mL^−1^). The antibacterial and antifungal activities of the remaining tested ketones (*α*-amino ketones, imidazole **5**–**7** and triazole **9**–**11** derivatives) were much lower (Table 4).

## 4. Conclusions

In conclusion, herein, we have reported the synthesis of new benzofuryl *β*-amino alcohols bearing imidazolyl and triazolyl substituents. The chiral *β*-amino alcohols were successfully prepared (96–99% ee) by the asymmetric transfer hydrogenation of the *α*-azole ketones catalyzed by RhCl[(*R*,*R*)-TsDPEN](C_5_Me_5_). With the application of the experimental and theoretical ECD data, the absolute configuration of the products was established as *R*. It has been shown that also microbiological reduction is a highly efficient and enantioselective method for the synthesis of benzofuryl *β*-amino alcohols, but its effectiveness strongly depends on the ketone structure and bio-reduction conditions. All the compounds prepared in this project were tested for their antibacterial and antifungal properties. From among chiral *β*-amino alcohols, the 3,5-dimethylbenzofuryl alcohols with imidazole and triazole rings exhibited good activity against bacteria of the *S. aureus* ATCC 25923 and yeast of *M. furfur* DSM 6170 types. 1-(Benzofuran-2-yl)-2-bromoethanone, alike benzofuryl *α*-bromo ketones possessing methyl and ethyl groups at 3, 5 and 7 positions, displayed an excellent antifungal activity against *M. furfur*.

## Figures and Tables

**Table:** 

No.	R^1^	R^2^	R^3^	No.	R^1^	R^2^	R^3^	X	Yield (%)
**1**	H	H	H	**5**	H	H	H	CH	53
**2**	H	H	Et	**6**	H	H	Et	CH	55
**3**	Me	H	H	**7**	Me	H	H	CH	49
**4**	Me	Me	H	**8**	Me	Me	H	CH	58
				**9**	H	H	H	N	42
				**10**	H	H	Et	N	47
				**11**	Me	H	H	N	53
				**12**	Me	Me	H	N	83

**Table:** 

No.	R^1^	R^2^	R^3^	X	Time (h)	No.	Yield (%)	ee (%)
**5**	H	H	H	CH	24	**13**	79	99
**6**	H	H	Et	CH	24	**14**	75	96
**7**	Me	H	H	CH	24	**15**	66	98
**8**	Me	Me	H	CH	24	**16**	76	98
**9**	H	H	H	N	48	**17**	71	96
**10**	H	H	Et	N	48	**18**	77	98
**11**	Me	H	H	N	48	**19**	57	97
**12**	Me	Me	H	N	48	**20**	48	97

**Table:** 

No.	R^1^	R^2^	R^3^	X	No.	Yield (%)
**5**	H	H	H	CH	**21**	95
**6**	H	H	Et	CH	**22**	93
**7**	Me	H	H	CH	**23**	85
**8**	Me	Me	H	CH	**24**	88
**9**	H	H	H	N	**25**	88
**10**	H	H	Et	N	**26**	91
**11**	Me	H	H	N	**27**	87
**12**	Me	Me	H	N	**28**	64

**Table 1:** The efficiency and enantioselectivity of ketones **6**, **9** and **12** bioreduction to alcohols **14**, **17** and **20**.

No.	Conditions	Inhibitor 1.25 × 10^−5^ mol	Yield (%)	ee (%)/Conf.
**14**	*Aureobasidium Pullulans* Phosphate Buffer pH 7, 30 °C, 1 h	-	76	59 (*S*)
	*Aureobasidium Pullulans* Phosphate Buffer pH 7, 30 °C, 2 h	AMA-1	78	73 (*S*)
	*Aureobasidium Pullulans* Phosphate Buffer pH 7, 24 °C, 1 h	allyl alcohol	79	82 (*S*)
	*Aureobasidium Pullulans* Phosphate Buffer pH 7, 24 °C, 1 h	cysteine	79	99 (*S*)
	*Saccharomyces Cerevisiae* Phosphate Buffer pH 7, 30 °C, 24 h	-	89	86 (*R*)
**17**	*Aureobasidium Pullulans* Phosphate Buffer pH 7, 24 °C, 2 h	-	88	10 (*S*)
	*Aureobasidium Pullulans* Phosphate Buffer pH 7, 24 °C, 2 h	AMA-1	80	16 (*S*)
	*Aureobasidium Pullulans* Phosphate Buffer pH 7, 24 °C, 2 h	allyl alcohol	80	28 (*S*)
	*Aureobasidium Pullulans* Phosphate Buffer pH 7, 24 °C, 2 h	cysteine	80	50 (*S*)
	*Saccharomyces Cerevisiae* Phosphate Buffer pH 7, 30 °C, 24 h	-	90	89 (*R*)
**20**	*Aureobasidium Pullulans* Phosphate Buffer pH 7, 24 °C, 98 h	-	65	51 (*R*)
	*Aureobasidium Pullulans* Phosphate Buffer pH 7, 30 °C, 2 h	AMA-1	14	38 (*R*)
	*Aureobasidium Pullulans* Phosphate Buffer pH 7, 30 °C, 2 h	ethyl chloroacetate	13	38 (*R*)
	*Aureobasidium Pullulans* Phosphate Buffer pH 7, 27 °C, 24 h	cysteine	33	32 (*R*)
	*Saccharomyces Cerevisiae* Phosphate Buffer pH 7, 30 °C, 24 h	-	95	99 (*R*)

**Table 2:** Optical rotations—both measured in chloroform and calculated at the IEFPCM/B3LYP/*aug*-cc-pVTZ level and *ΔΔG*-based Boltzmann averaged—for **13**–**20**.

No.		Optical Rotations (*λ*) ^a^			
		589 nm	578 nm	546 nm	436 nm
**13**	exp.	+79	+84	+95	+159
	calcd.	+18	+20	+23	+54
**14**	exp.	+55	+71	+72	n.a.
	calcd.	−21	−22	−24	−35
**15**	exp.	−19	−18	−26	−64
	calcd.	+108	+114	+134	+268
**16**	exp.	−20	−22	−26	−65
	calcd.	+76	+79	+93	+185
**17**	exp.	+68	+73	+88	+148
	calcd.	−91	−95	−109	−197
**18**	exp.	+74	+78	+94	+153
	calcd.	−41	−43	−49	−84
**19**	exp.	+0.2	+4	−2	−12
	calcd.	+28	+29	+35	+80
**20**	exp.	−3	+1	+0.5	−14
	calcd.	+9	+10	+22	+33

^a^ The concentration of samples equals to 0.25 g 100 mL^−1^.

**Table 3:** MIC and MBC (μg mL^−1^) of benzofuryl *β*-amino alcohols against selected bacteria and yeasts.

No.	*Escherichia Coli* ATCC 25922		*Escherichia Coli* ATCC 8739		*Staphylococcus Aureus* ATCC 25923		*Staphylococcus Aureus* ATCC 6538		*Candida Albicans* ATCC 10231		*Malassezia Furfur* DSM 6170	
	MIC	MBC	MIC	MBC	MIC	MBC	MIC	MBC	MIC	MBC	MIC	MBC
**13**	512	512	512	>512	>512	>512	>512	>512	512	>512	256	256
**14**	>512	>512	>512	>512	>512	>512	>512	>512	>512	>512	>512	>512
**15**	512	512	512	>512	512	>512	>512	>512	512	512	256	256
**16**	512	512	512	512	64	96	>512	>512	512	512	256	256
**17**	512	>512	512	>512	>512	>512	512	>512	512	>512	256	256
**18**	512	>512	512	>512	>512	>512	>512	>512	512	>512	256	256
**19**	512	>512	512	>512	>512	>512	>512	>512	512	512	256	256
**20**	512	>512	512	>512	>512	>512	>512	>512	512	512	64	64
**21**	512	>512	512	512	>512	>512	>512	>512	512	>512	256	256
**22**	512	512	512	>512	512	512	512	512	512	512	256	256
**23** ^a^	-	-	-	-	-	-	-	-	-	-	-	-
**24**	512	512	512	>512	512	>512	>512	>512	512	512	256	256
**25**	512	>512	512	>512	>512	>512	>512	>512	512	512	256	256
**26**	512	>512	512	>512	>512	>512	>512	>512	512	>512	128	128
**27**	512	>512	512	>512	>512	>512	>512	>512	512	512	256	256
**28**	512	>512	512	>512	>512	>512	>512	>512	512	512	256	256

^a^ compound not tested due to lack of solubility in water, and precipitation in low ethanol or DMSO (dimethyl sulfoxide) concentration required to be not toxic to microbial cells.

**Table 4:** MIC and MBC (μg mL−1) of benzofuryl α-bromo and α-amino ketones against selected bacteria and yeasts.

No.	*Escherichia Coli* ATCC 25922		*Escherichia Coli* ATCC 8739		*Staphylococcus Aureus* ATCC 25923		*Staphylococcus Aureus* ATCC 6538		*Candida Albicans* ATCC 10231		*Malassezia Furfur* DSM 6170	
	MIC	MBC	MIC	MBC	MIC	MBC	MIC	MBC	MIC	MBC	MIC	MBC
**1**	64	64	64	64	32	256	64	64	512	512	1.5	1.5
**2**	128	256	256	256	256	256	48	48	256	256	1.5	1.5
**3**	256	256	512	512	48	64	64	96	>512	>512	1.5	1.5
**4**	>512	>512	>512	>512	16	16	16	64	>512	>512	1.5	1.5
**5**	512	>512	>512	>512	>512	>512	>512	>512	512	>512	512	>512
**6**	>512	>512	>512	>512	512	>512	512	512	512	512	512	512
**7**	>512	>512	>512	>512	>512	>512	>512	>512	512	>512	512	512
**8**	>512	>512	>512	>512	>512	>512	>512	>512	>512	>512	128	256
**9**	>512	>512	>512	>512	>512	>512	>512	>512	>512	>512	512	>512
**10**	>512	>512	>512	>512	>512	>512	>512	>512	>512	>512	512	>512
**11**	>512	>512	>512	>512	>512	>512	>512	>512	>512	>512	>512	>512
**12**	512	512	256	512	256	512	128	256	512	512	256	256

**Table 5:** MIC and MBC (μg mL^−1^) of standard antimicrobial agents estimated against bacteria and fungi using CLSI method [51].

**Tested Microorganism**	**AMP**		**K**		**TE**	
	MIC	MBC	MIC	MBC	MIC	MBC
***Escherichia Coli*** ATCC 8739	>512	>512	512	>512	64	256
***Escherichia Coli*** ATCC 25922	4	4	32	32	1	256
***Staphylococcus Aureus*** ATCC 6538	1	64	8	16	4	256
***Staphylococcus Aureus*** ATCC 25923	0.5	32	64	128	0.5	512
	**AMB**		**FLU**		**KCA**	
	MIC	MBC	MIC	MBC	MIC	MBC
***Candida Albicans*** ATCC 10231	0.25	0.5	>512	>512	>512	>512
***Malassezia Furfur*** DSM 6170	>512	>512	4	96	>512	>512

Key: AMP—ampicillin, K—kanamycin, TE—tetracycline, AMB—amphotericin B, FLU—fluconazole, KCA—ketoconazole.

## Supplementary Materials

The following are available online at https://www.mdpi.com/1996-1944/13/18/4080/s1, Figure S1: (a) ^1^H NMR and (b) ^13^C NMR spectra of 1-(benzofuran-2-yl)-2-(1*H*-imidazol-1-yl)ethanone (**5**), Figure S2: (a) ^1^H NMR and (b) ^13^C NMR spectra of 1-(7-ethylbenzofuran-2-yl)-2-(1*H*-imidazol-1-yl)ethanone (**6**), Figure S3: (a) ^1^H NMR and (b) ^13^C NMR spectra of 2-(1*H*-imidazol-1-yl)-1-(3-methylbenzofuran-2-yl)ethanone (**7**), Figure S4: (a) ^1^H NMR and (b) ^13^C NMR spectra of 1-(3,5-dimethylbenzofuran-2-yl)-2-(1*H*-imidazol-1-yl)ethanone (**8**), Figure S5: (a) ^1^H NMR and (b) ^13^C NMR spectra of 1-(benzofuran-2-yl)-2-(1*H*-1,2,4-triazol-1-yl)ethanone (**9**), Figure S6: (a) ^1^H NMR and (b) ^13^C NMR spectra of 1-(7-ethylbenzofuran-2-yl)-2-(1*H*-1,2,4-triazol-1-yl)ethanone (**10**), Figure S7: (a) ^1^H NMR and (b) ^13^C NMR spectra of 1-(3-methylbenzofuran-2-yl)-2-(1*H*-1,2,4-triazol-1-yl)ethanone (**11**), Figure S8: (a) ^1^H NMR and (b) ^13^C NMR spectra of 1-(3,5-dimethylbenzofuran-2-yl)-2-(1*H*-1,2,4-triazol-1-yl)ethanone (**12**), Figure S9: (a) ^1^H NMR and (b) ^13^C NMR spectra of (*R*)-1-(benzofuran-2-yl)-2-(1*H*-imidazol-1-yl)ethan-1-ol (**13**), Figure S10: (a) ^1^H NMR and (b) ^13^C NMR spectra of (*R*)-1-(7-ethylbenzofuran-2-yl)-2-(1*H*-imidazol-1-yl)ethan-1-ol (**14**), Figure S11: (a) ^1^H NMR and (b) ^13^C NMR spectra of (*R*)-2-(1*H*-imidazol-1-yl)-1-(3-methylbenzofuran-2-yl)ethan-1-ol (**15**), Figure S12: (a) ^1^H NMR and (b) ^13^C NMR spectra of (*R*)-1-(3,5-dimethylbenzofuran-2-yl)-2-(1*H*-imidazol-1-yl)ethan-1-ol (**16**), Figure S13: (a) ^1^H NMR and (b) ^13^C NMR spectra of (*R*)-1-(benzofuran-2-yl)-2-(1*H*-1,2,4-triazol-1-yl)ethan-1-ol (**17**), Figure S14: (a) ^1^H NMR and (b) ^13^C NMR spectra of (*R*)-1-(7-ethylbenzofuran-2-yl)-2-(1*H*-1,2,4-triazol-1-yl)ethan-1-ol (**18**), Figure S15: (a) ^1^H NMR and (b) ^13^C NMR spectra of (*R*)-1-(3-methylbenzofuran-2-yl)-2-(1*H*-1,2,4-triazol-1-yl)ethan-1-ol (**19**), Figure S16: (a) ^1^H NMR and (b) ^13^C NMR spectra of (*R*)-1-(3,5-dimethylbenzofuran-2-yl)-2-(1H-1,2,4-triazol-1-yl)ethan-1-ol (**20**), Figure S17: (a) ^1^H NMR and (b) ^13^C NMR spectra of 1-(benzofuran-2-yl)-2-(1*H*-imidazol-1-yl)ethan-1-ol (**21**), Figure S18: (a) ^1^H NMR and (b) ^13^C NMR spectra of 1-(7-ethylbenzofuran-2-yl)-2-(1*H*-imidazol-1-yl)ethan-1-ol (**22**), Figure S19: (a) ^1^H NMR and (b) ^13^C NMR spectra of 2-(1*H*-imidazol-1-yl)-1-(3-methylbenzofuran-2-yl)ethan-1-ol (**23**), Figure S20: (a) ^1^H NMR and (b) ^13^C NMR spectra of 1-(3,5-dimethylbenzofuran-2-yl)-2-(1*H*-imidazol-1-yl)ethan-1-ol (**24**), Figure S21: (a) ^1^H NMR and (b) ^13^C NMR spectra of 1-(benzofuran-2-yl)-2-(1*H*-1,2,4-triazol-1-yl)ethan-1-ol (**25**), Figure S22: (a) ^1^H NMR and (b) ^13^C NMR spectra of 1-(7-ethylbenzofuran-2-yl)-2-(1*H*-1,2,4-triazol-1-yl)ethan-1-ol (**26**), Figure S23: (a) ^1^H NMR and (b) ^13^C NMR spectra of 1-(3-methylbenzofuran-2-yl)-2-(1*H*-1,2,4-triazol-1-yl)ethan-1-ol (**27**), Figure S24: (a) ^1^H NMR and (b) ^13^C NMR spectra of 1-(3,5-dimethylbenzofuran-2-yl)-2-(1*H*-1,2,4-triazol-1-yl)ethan-1-ol (**28**), Tables S1, S3, S5, S7, S9, S11, S13 and S15: Total (E, in Hartree) and relative energies (*ΔE*, *ΔΔG*, in kcal mol^−1^), percentage populations (Pop.) and number of imaginary frequencies calculated at the IEFPCM(MeCN)/B3LYP/6-311++G(d,p) level for low-energy conformers of **13**–**20**, Tables S2, S4, S6, S8, S10, S12, S14 and S16: Total (E, in Hartree) and relative energies (*ΔE*, *ΔΔG*, in kcal mol^−1^), percentage populations (Pop.) and number of imaginary frequencies calculated at the IEFPCM(CHCl_3_)/B3LYP/6-311++G(d,p) level for low-energy conformers of **13**–**20**. Table S17–S24. Calculated at the IEFPCM(CHCl_3_)/B3LYP/*aug*-cc-pVTZ level optical rotations for alcohols **13**–**20**. Figure S25-S72. UV and ECD spectra of alcohols **13**–**20**.
