# Supplementary material for: Synthesis, Absolute Configuration, Antibacterial, and Antifungal Activities of Novel Benzofuryl β-Amino Alcohols
Source: Materials (Basel). 2020 Sep 14;13(18):4080. doi: 10.3390/ma13184080 (PMC7560283; doi:10.3390/ma13184080)
Supplement: Supplementary file 1 [file materials-13-04080-s001.pdf]

materials

Supplementary Materials

Synthesis, Absolute Configuration, Antibacterial and Antifungal Activities of Novel Benzofuryl β-Amino Alcohols

1. NMR Spectra

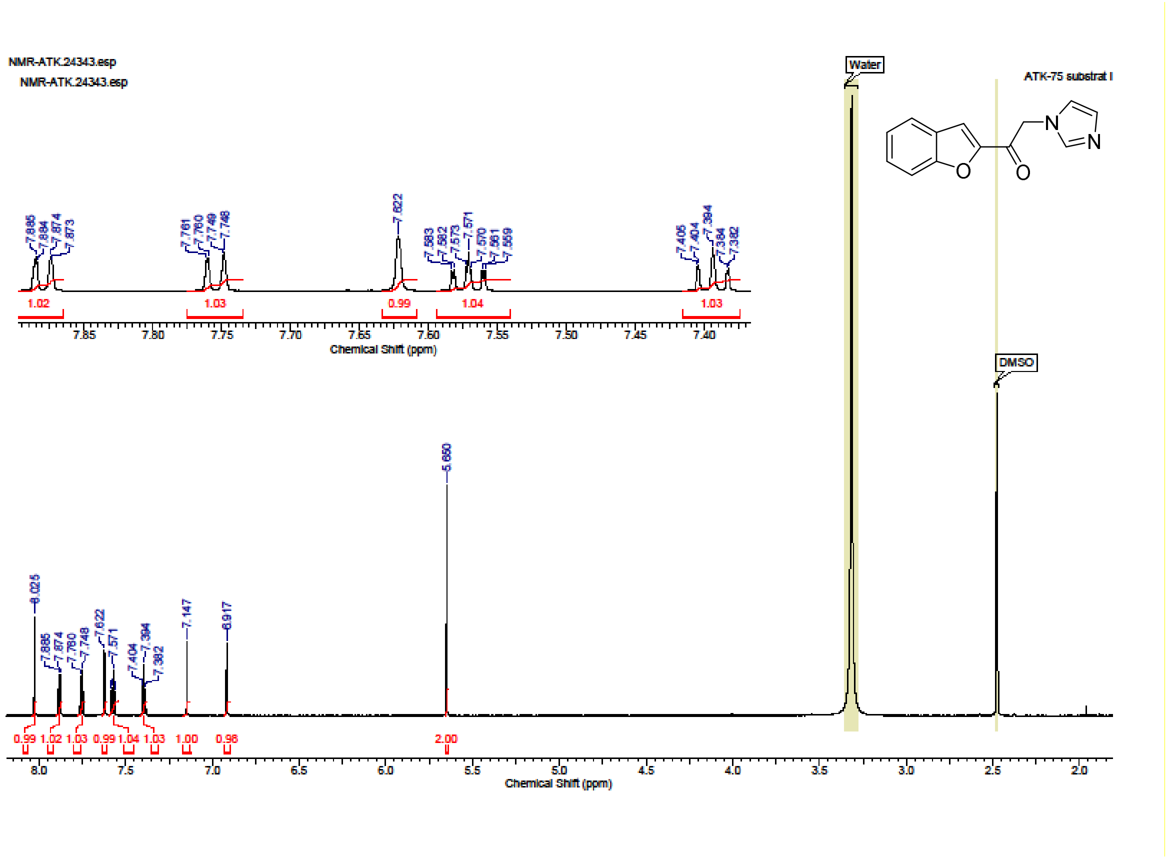

(a)

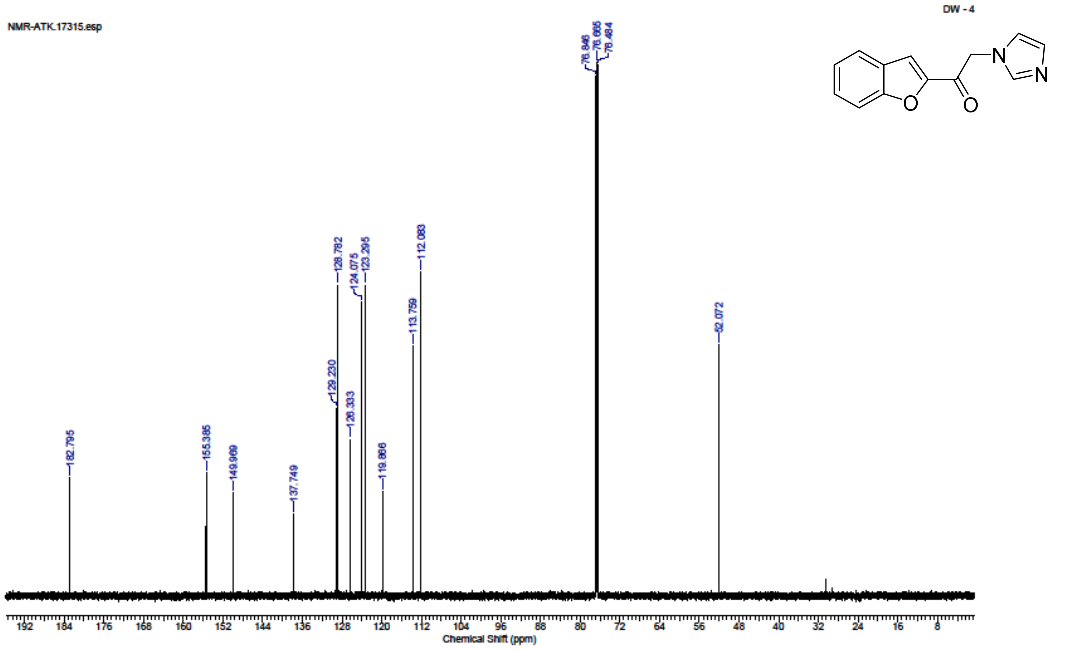

(**b**)

Figure 1. (**a**) ^{1}H NMR and (**b**) ^{13}C NMR spectra of 1-(benzofuran-2-yl)-2-(1*H*-imidazol-1-yl)ethanone (**5**).

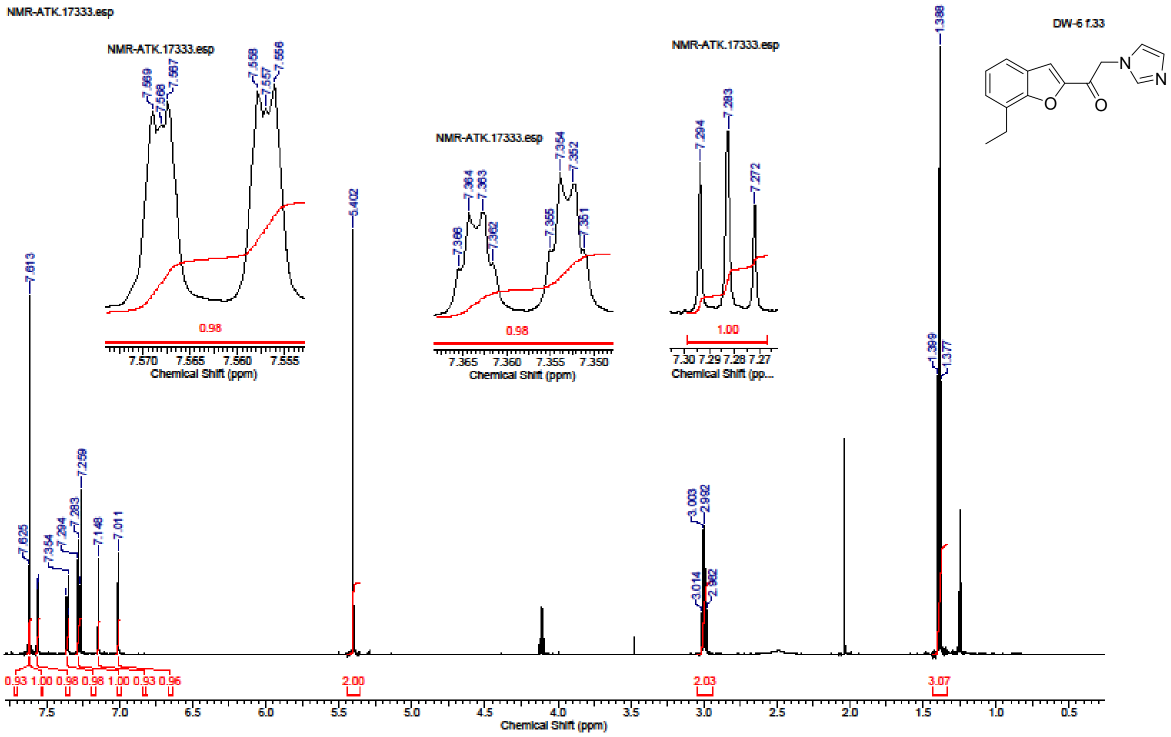

(**a**)

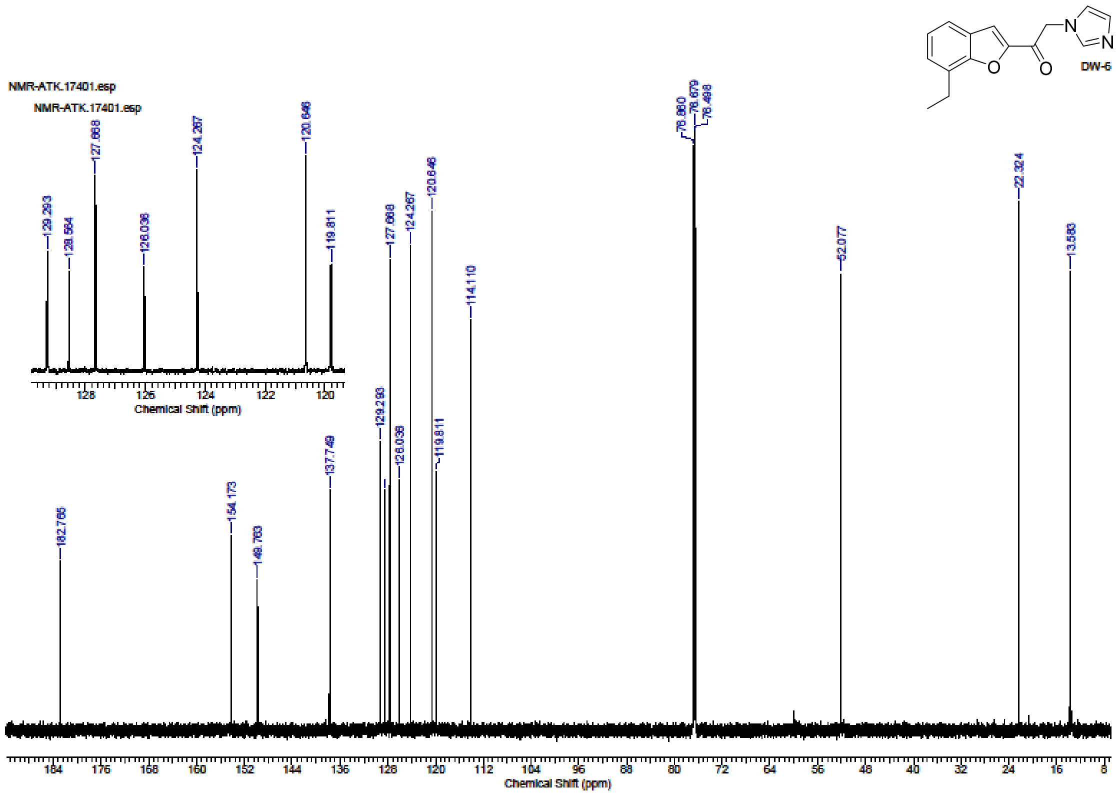

(**b**)

Figure S2. (**a**) ^{1}H NMR and (**b**) ^{13}C NMR spectra of 1-(7-ethylbenzofuran-2-yl)-2-(1*H*-imidazol-1-yl)ethanone (**6**).

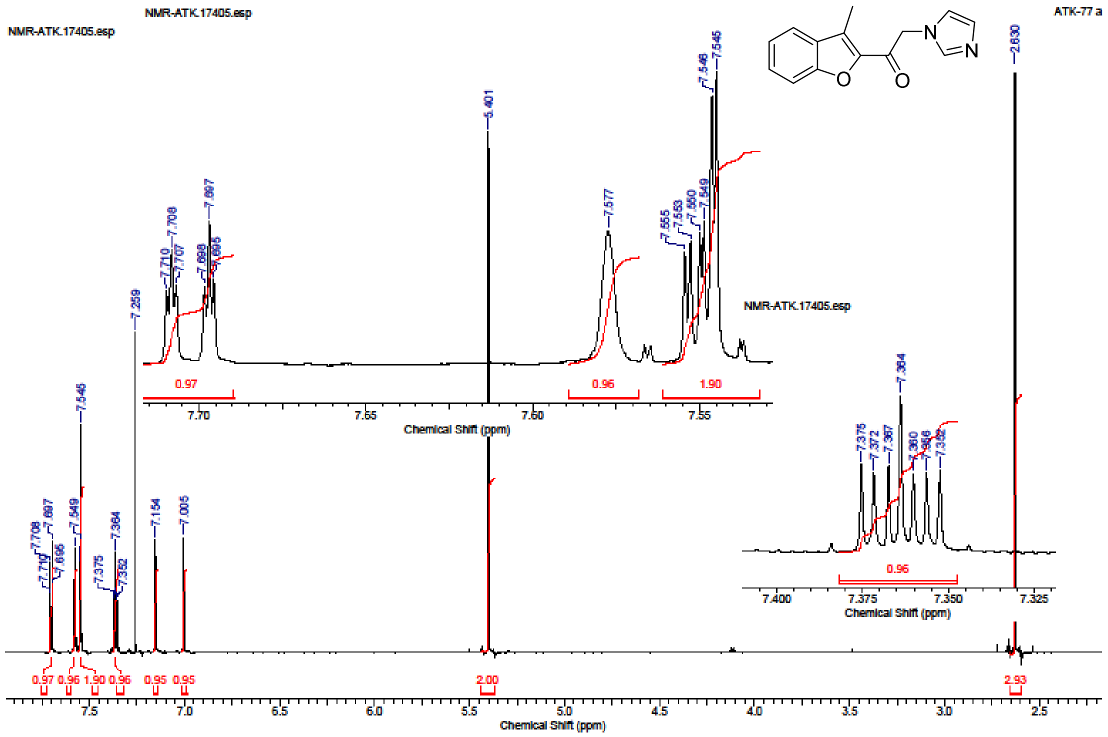

(**a**)

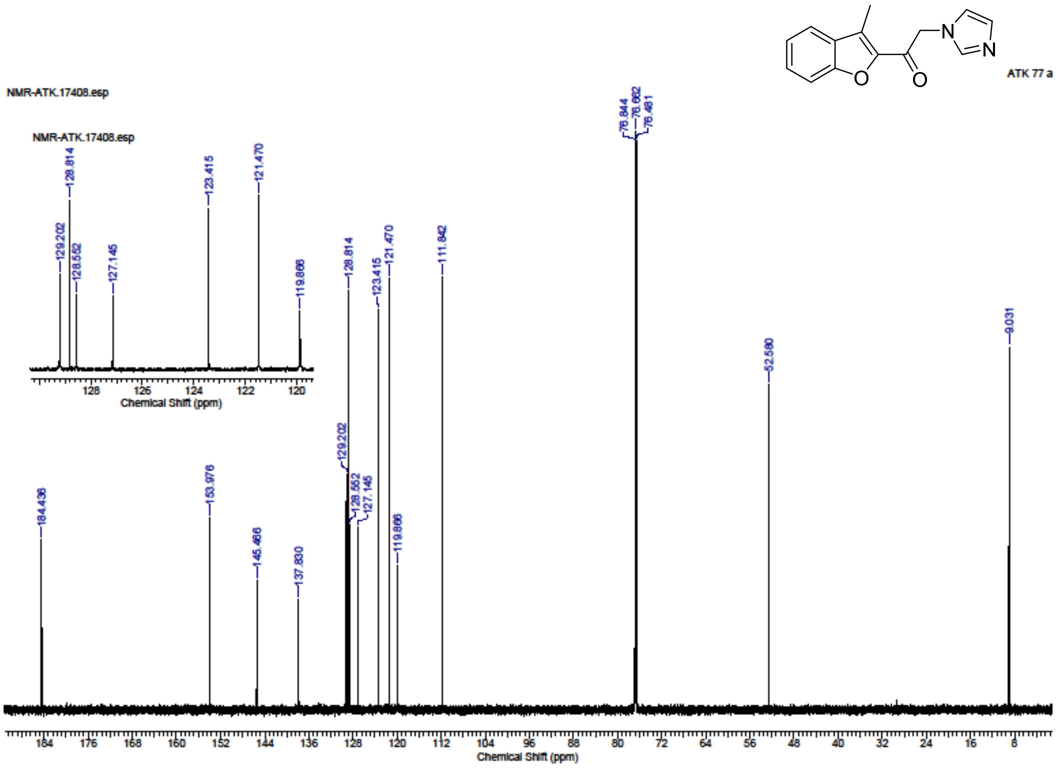

(**b**)

Figure 3. (**a**) ¹H NMR and (**b**) ¹³C NMR spectra of 2-(1*H*-imidazol-1-yl)-1-(3-methylbenzofuran-2-yl)ethanone (**7**).

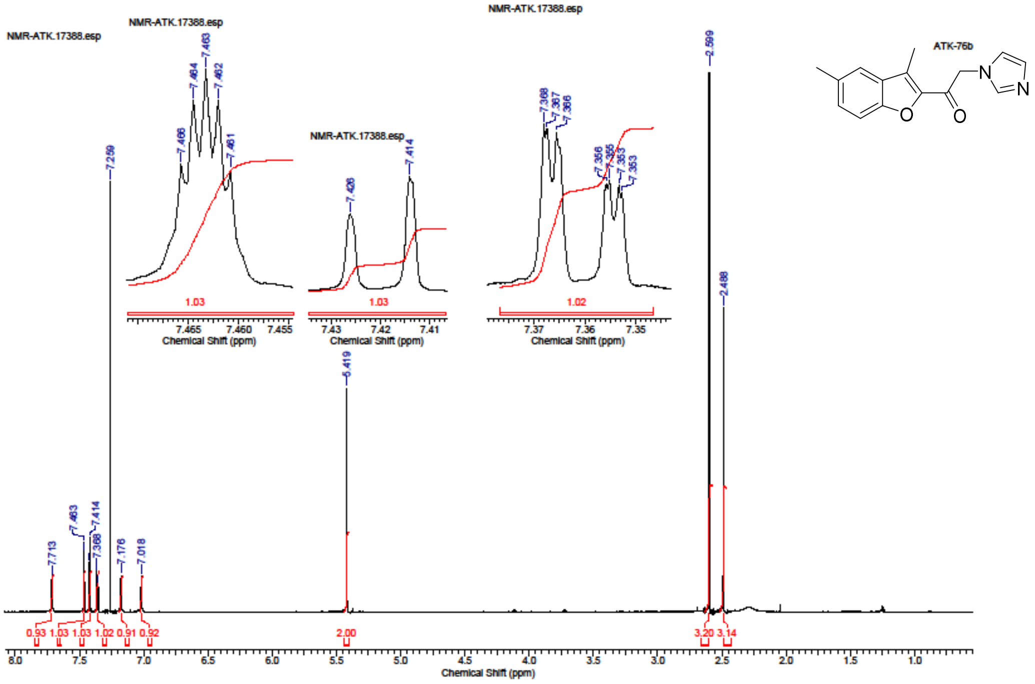

(**a**)

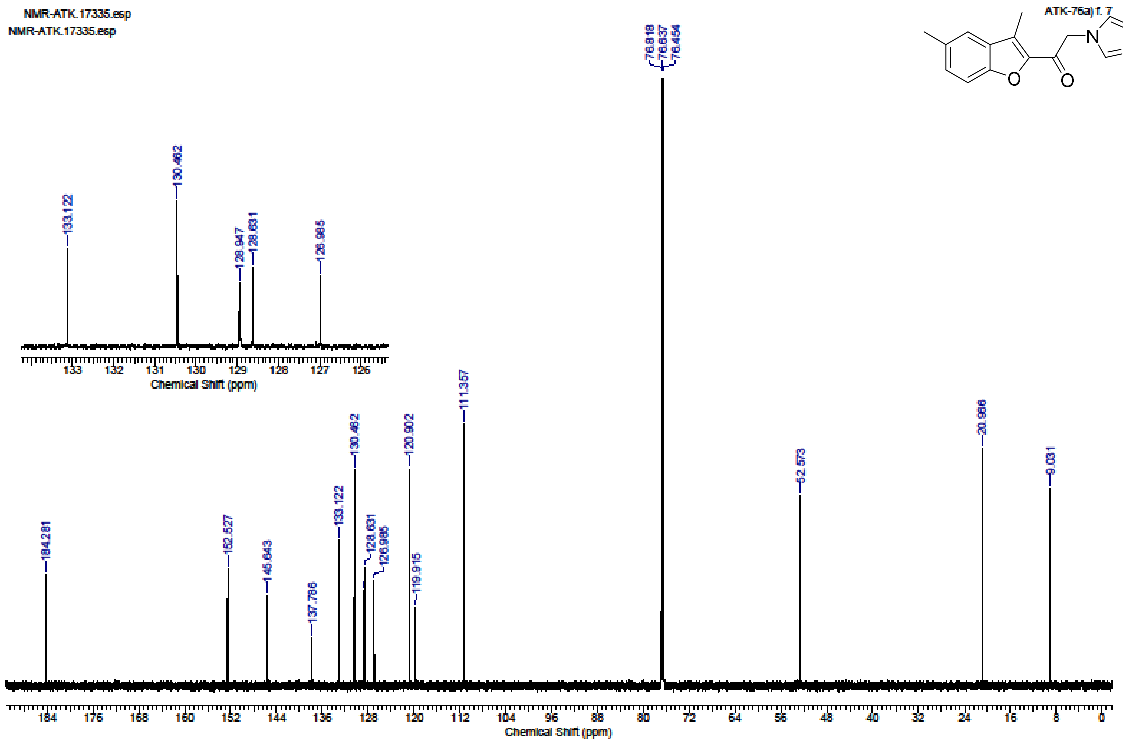

(**b**)

Figure 4. (a) ^{1}H NMR and (**b**) ^{13}C NMR spectra of 1-(3,5-dimethylbenzofuran-2-yl)-2-(1*H*-imidazol-1-yl)ethanone (**8**).

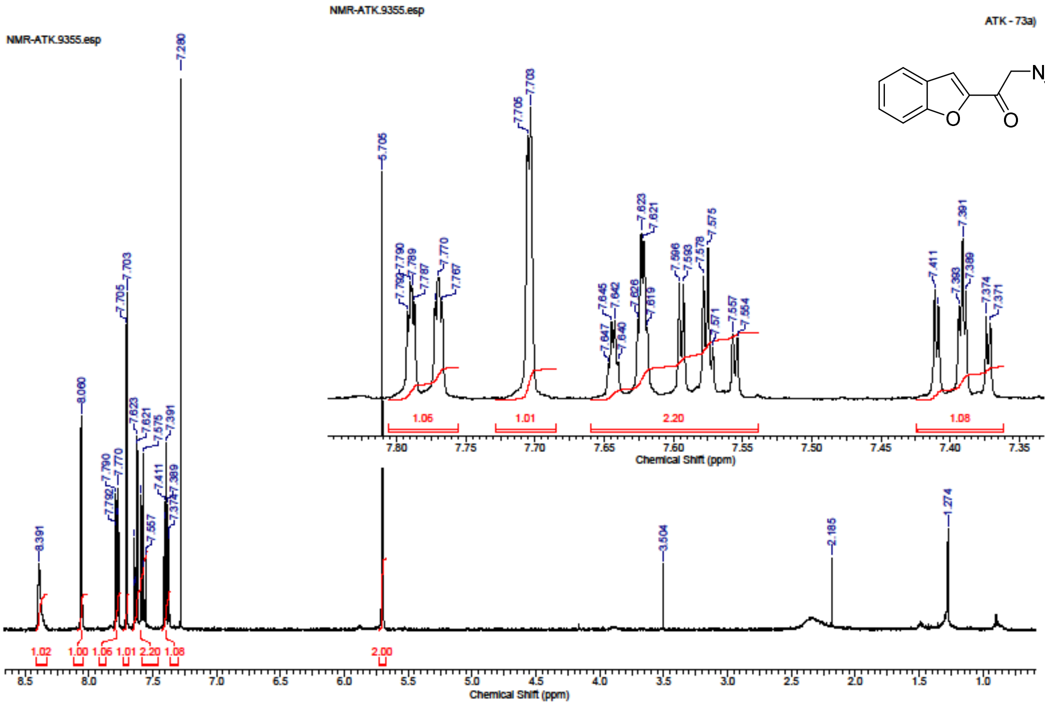

(**a**)

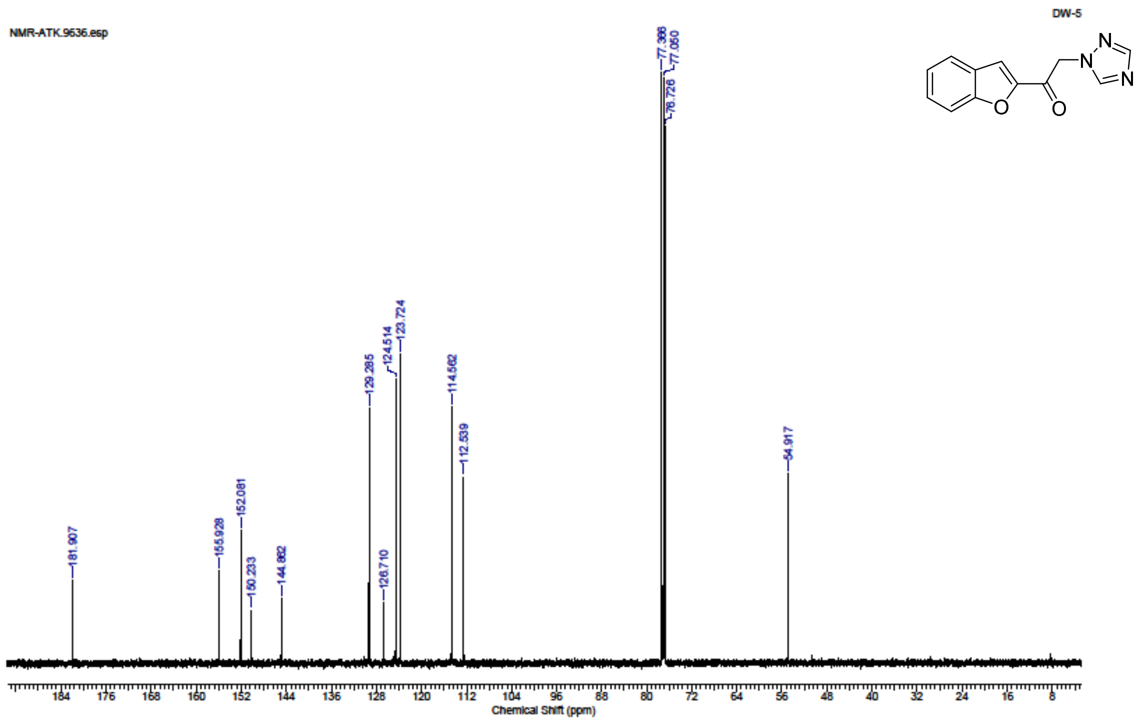

(**b**)

Figure 5. (**a**) ^{1}H NMR and (**b**) ^{13}C NMR spectra of 1-(benzofuran-2-yl)-2-(1*H*-1,2,4-triazol-1-yl)ethanone (**9**).

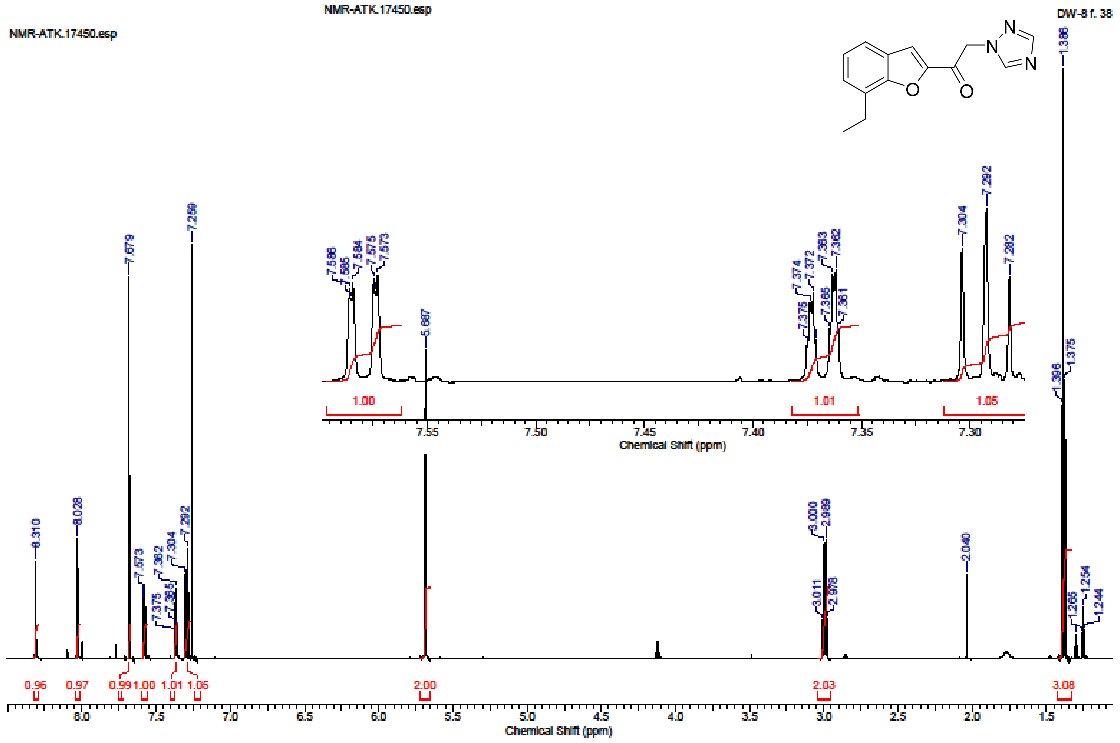

(**a**)

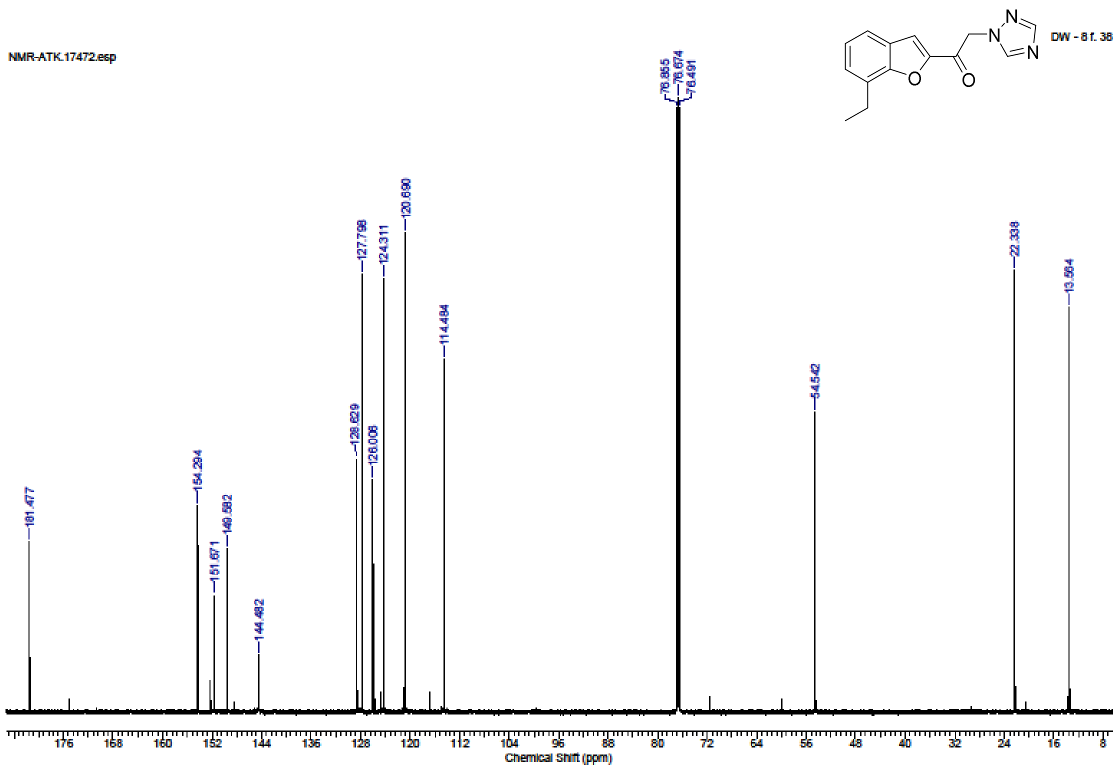

(**b**)

Figure 6. (**a**) ^{1}H NMR and (**b**) ^{13}C NMR spectra of 1-(7-ethylbenzofuran-2-yl)-2-(1*H*-1,2,4-triazol-1-yl)ethanone (**10**).

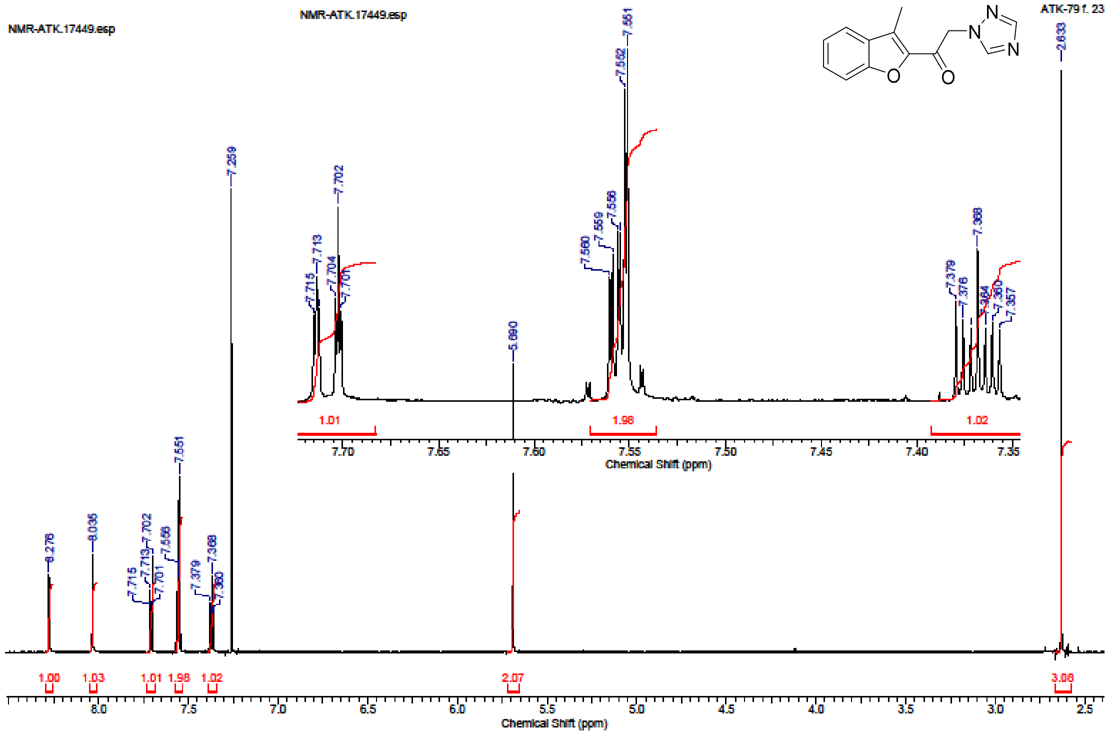

(**a**)

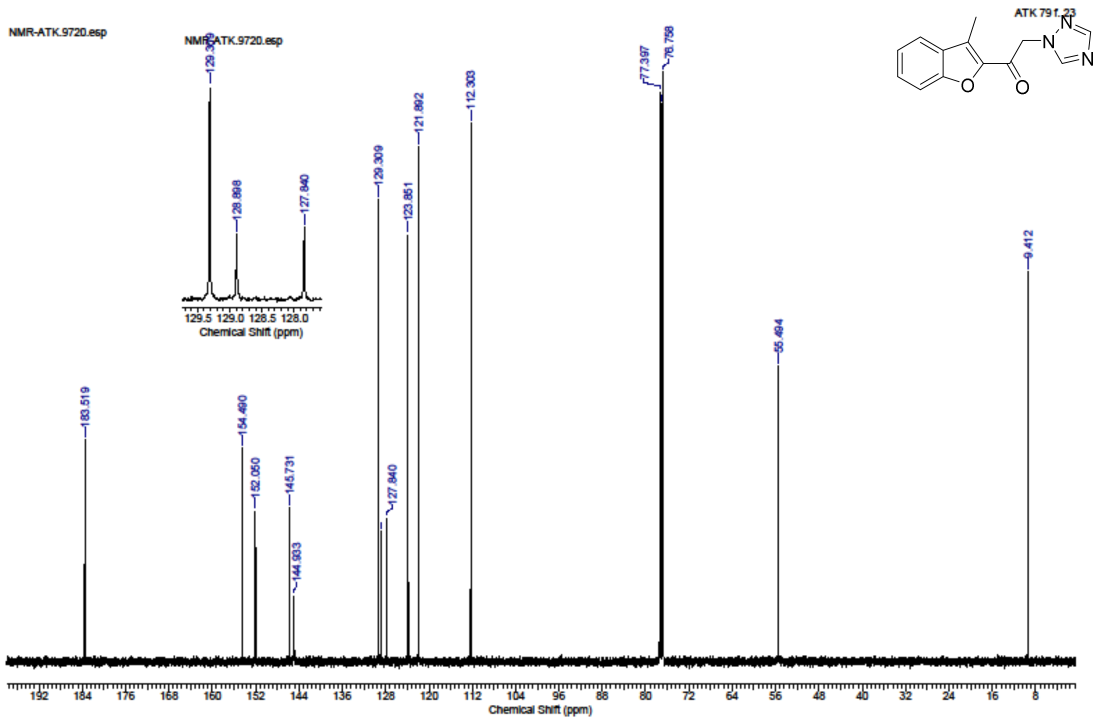

(**b**)

Figure 7. (**a**) ^{1}H NMR and (**b**) ^{13}C NMR spectra of 1-(3-methylbenzofuran-2-yl)-2-(1*H*-1,2,4-triazol-1-yl)ethanone (**11**).

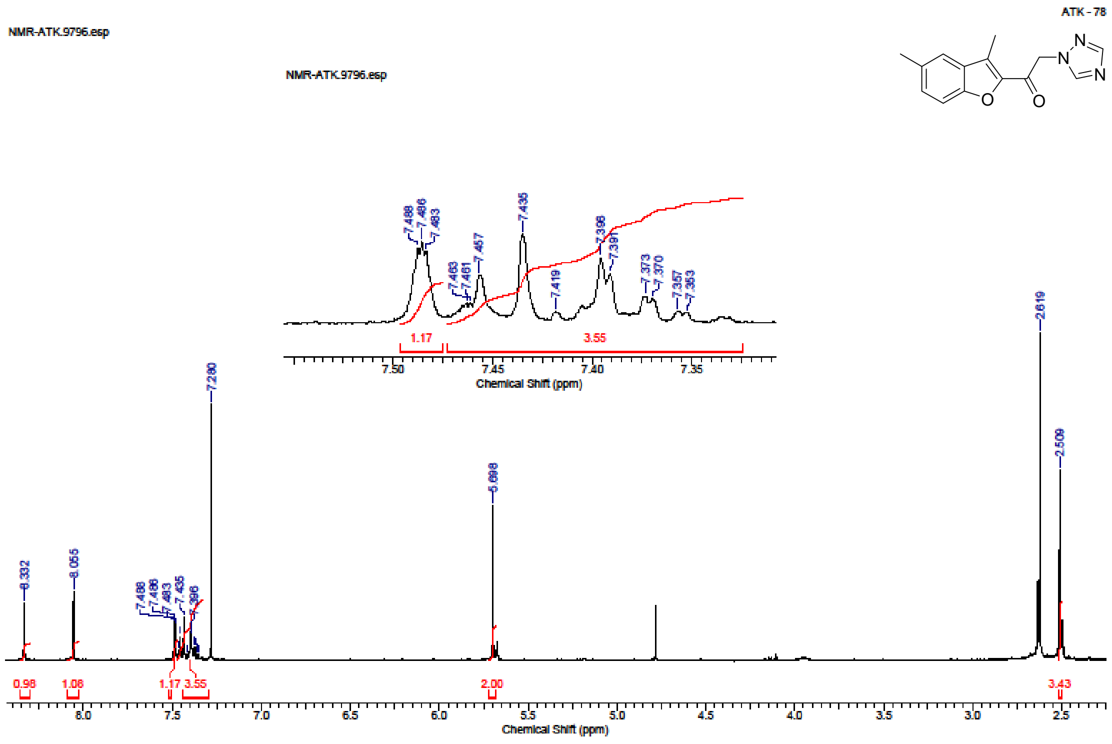

(**a**)

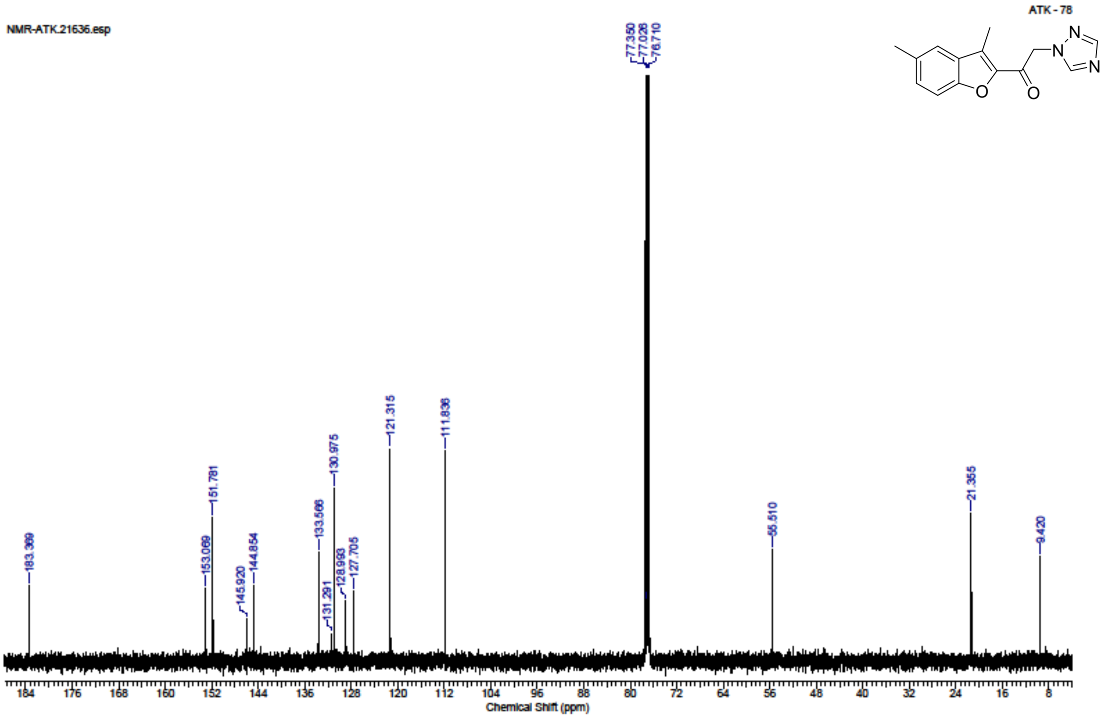

(**b**)

Figure S8. (**a**) ^{1}H NMR and (**b**) ^{13}C NMR spectra of 1-(3,5-dimethylbenzofuran-2-yl)-2-(1*H*-1,2,4-triazol-1-yl)ethanone (**12**).

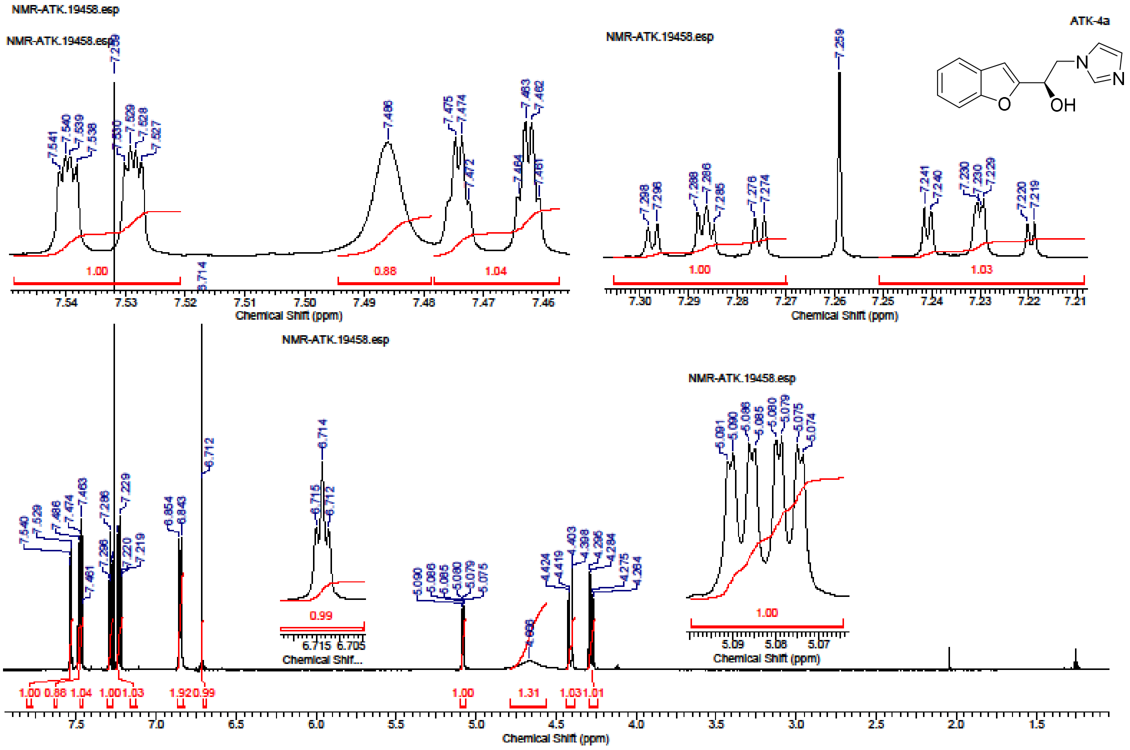

(**a**)

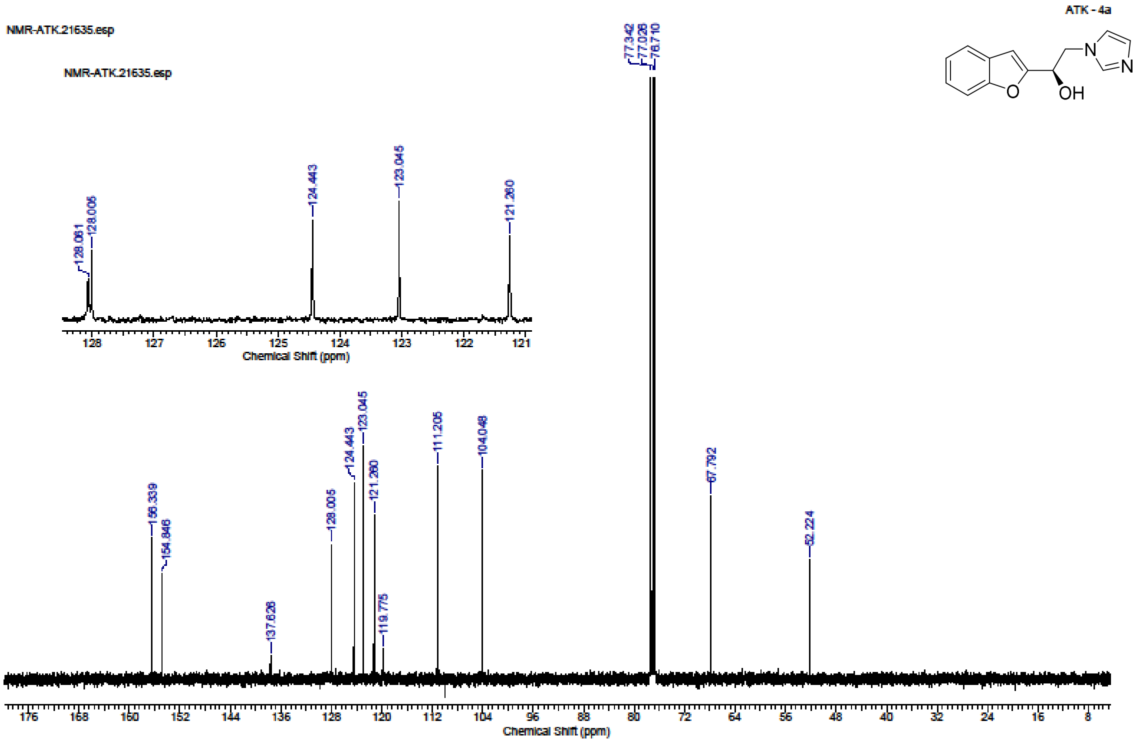

(**b**)

Figure S9. (**a**) ^{1}H NMR and (**b**) ^{13}C NMR spectra of (*R*)-1-(benzofuran-2-yl)-2-(1*H*-imidazol-1-yl)ethan-1-ol (**13**).

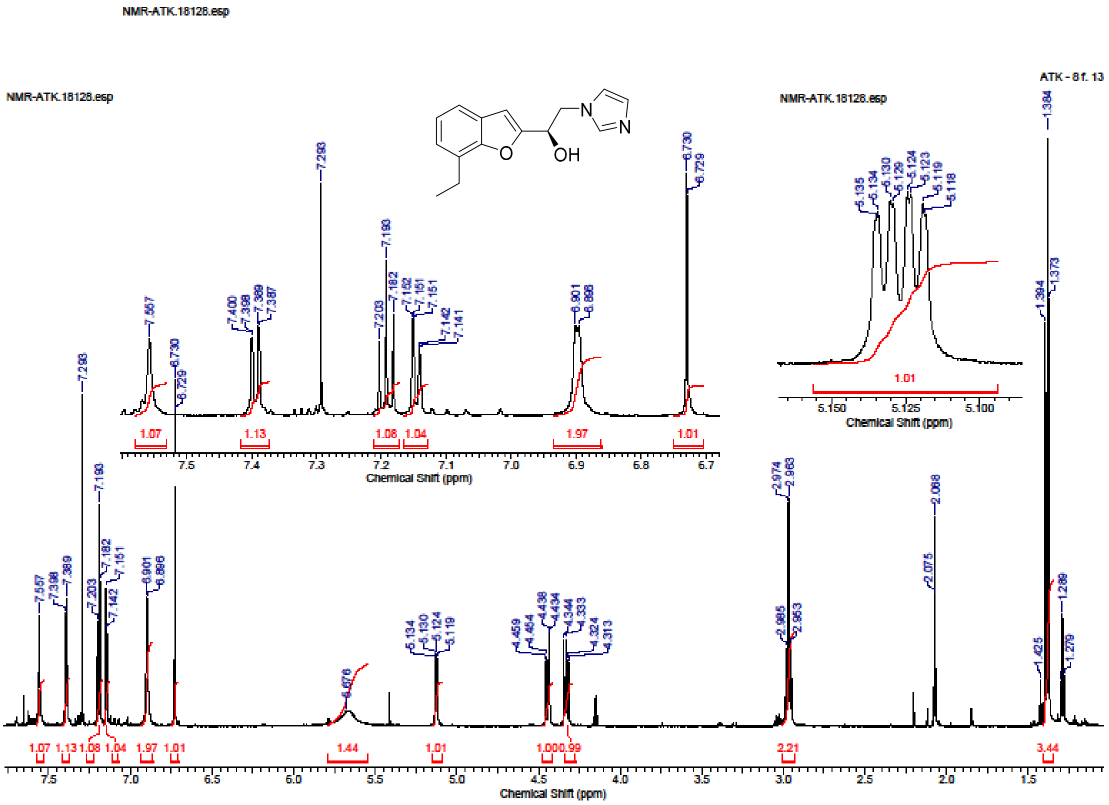

(**a**)

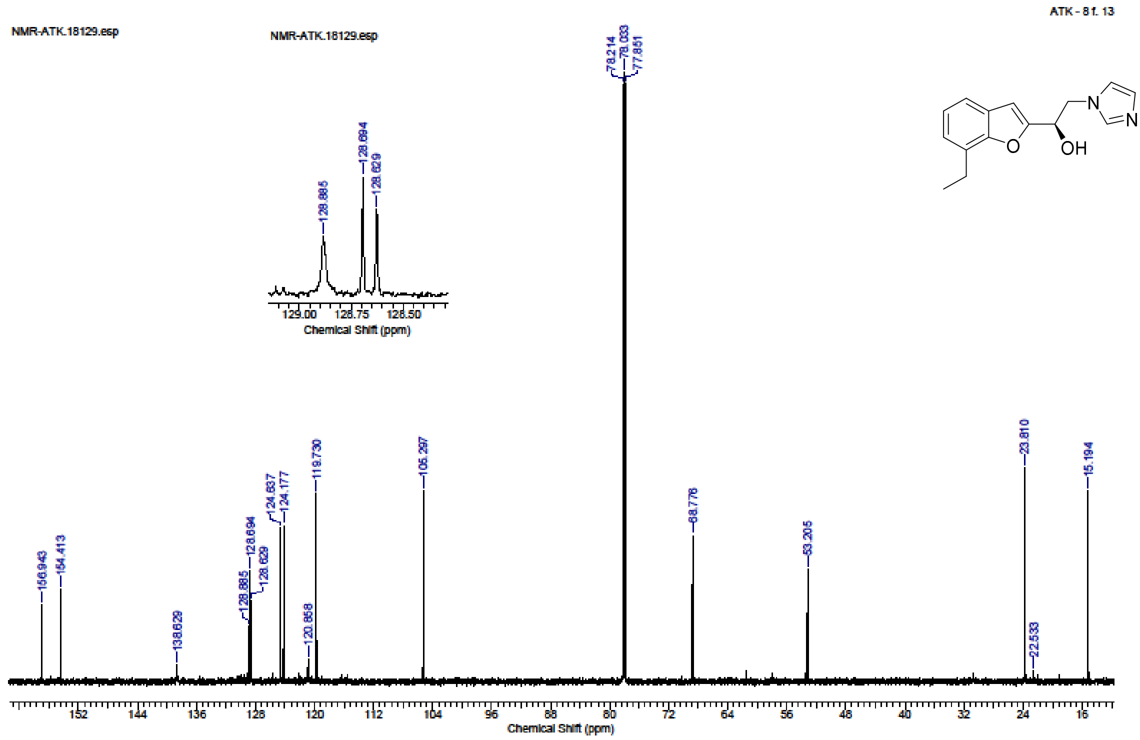

(**b**)

Figure S10. (**a**) ^{1}H NMR and (**b**) ^{13}C NMR spectra of (*R*)-1-(7-ethylbenzofuran-2-yl)-2-(1*H*-imidazol-1-yl)ethan-1-ol (**14**).

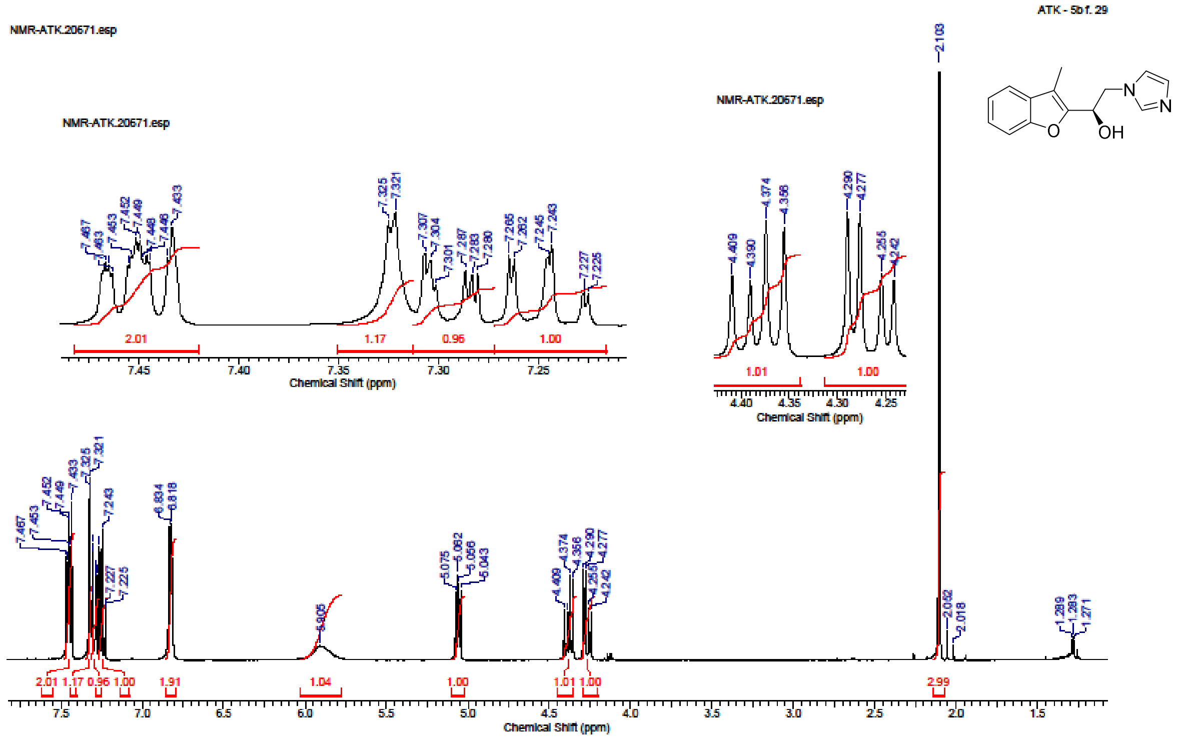

(**a**)

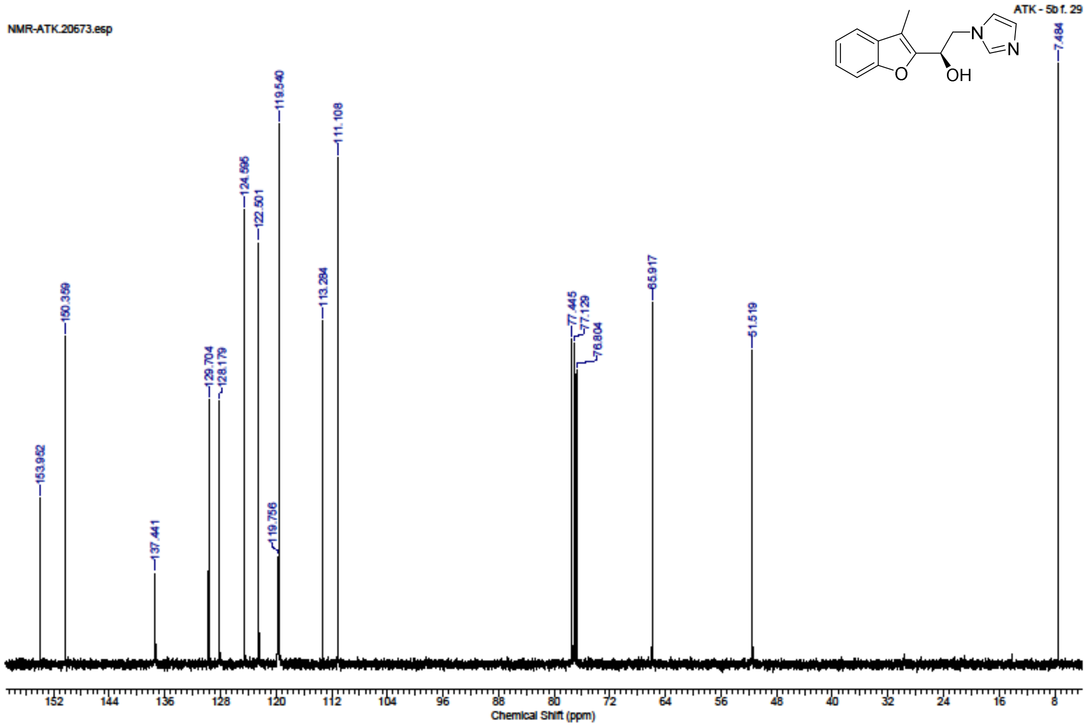

(**b**)

Figure S11. (**a**) ^{1}H NMR and (**b**) ^{13}C NMR spectra of (*R*)-2-(1*H*-imidazol-1-yl)-1-(3-methylbenzofuran-2-yl)ethan-1-ol (**15**).

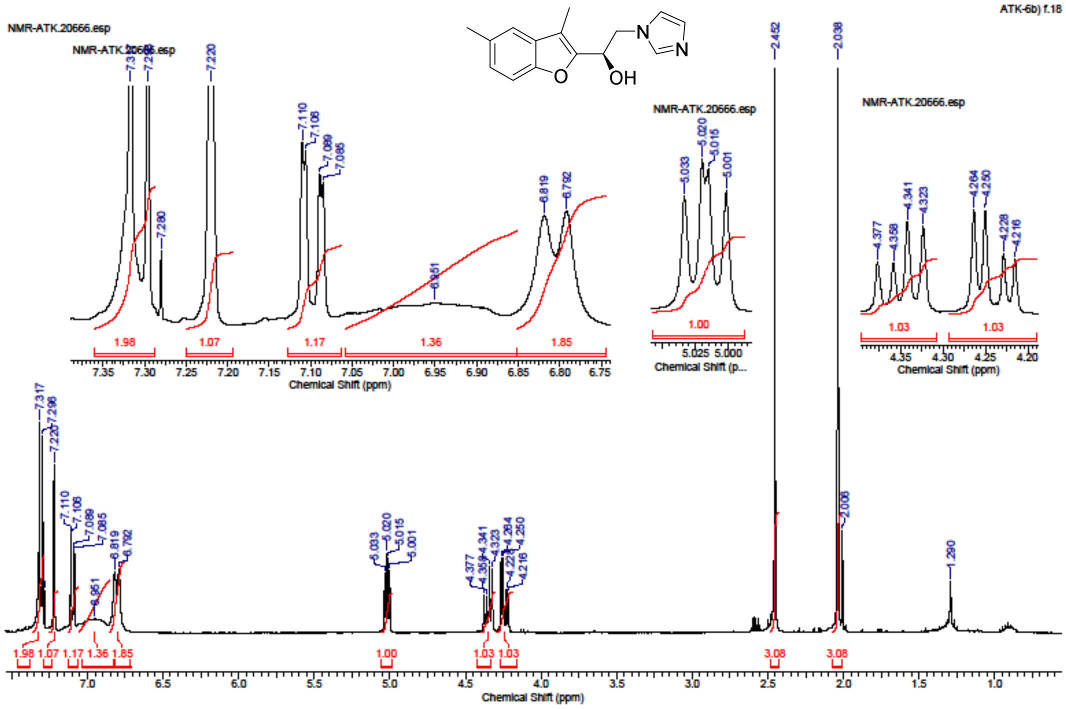

(**a**)

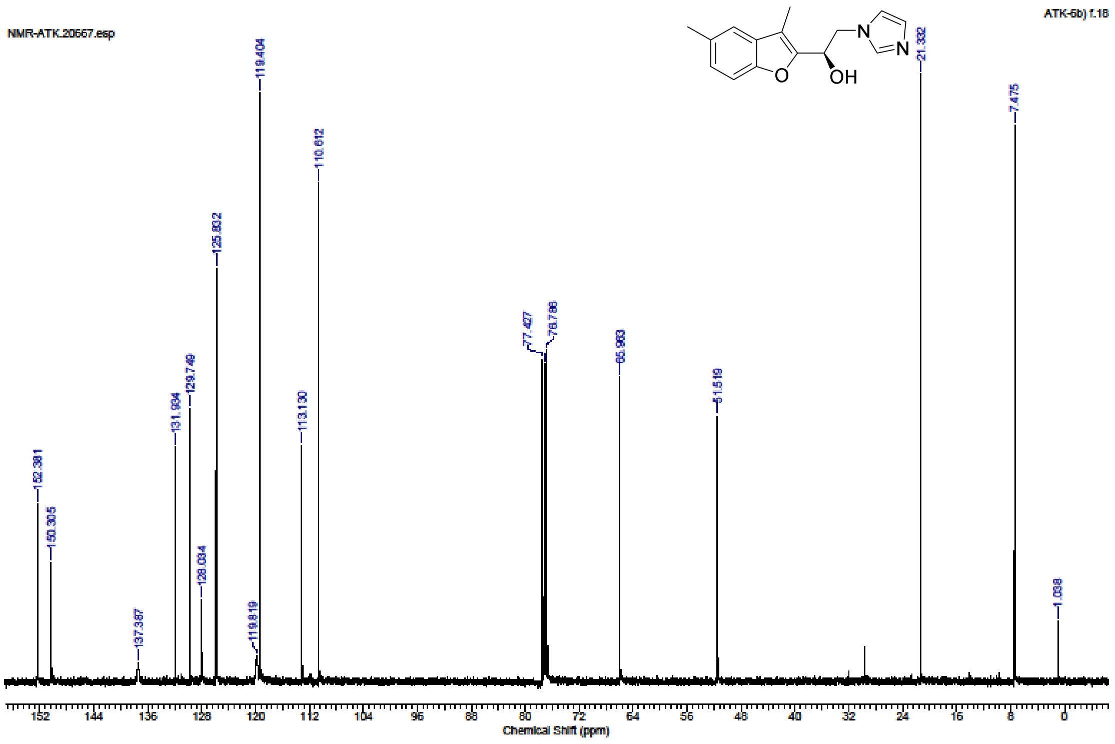

(**b**)

Figure 12. (**a**) ^{1}H NMR and (**b**) ^{13}C NMR spectra of (*R*)-1-(3,5-dimethylbenzofuran-2-yl)-2-(1*H*-imidazol-1-yl)ethan-1-ol (**16**).

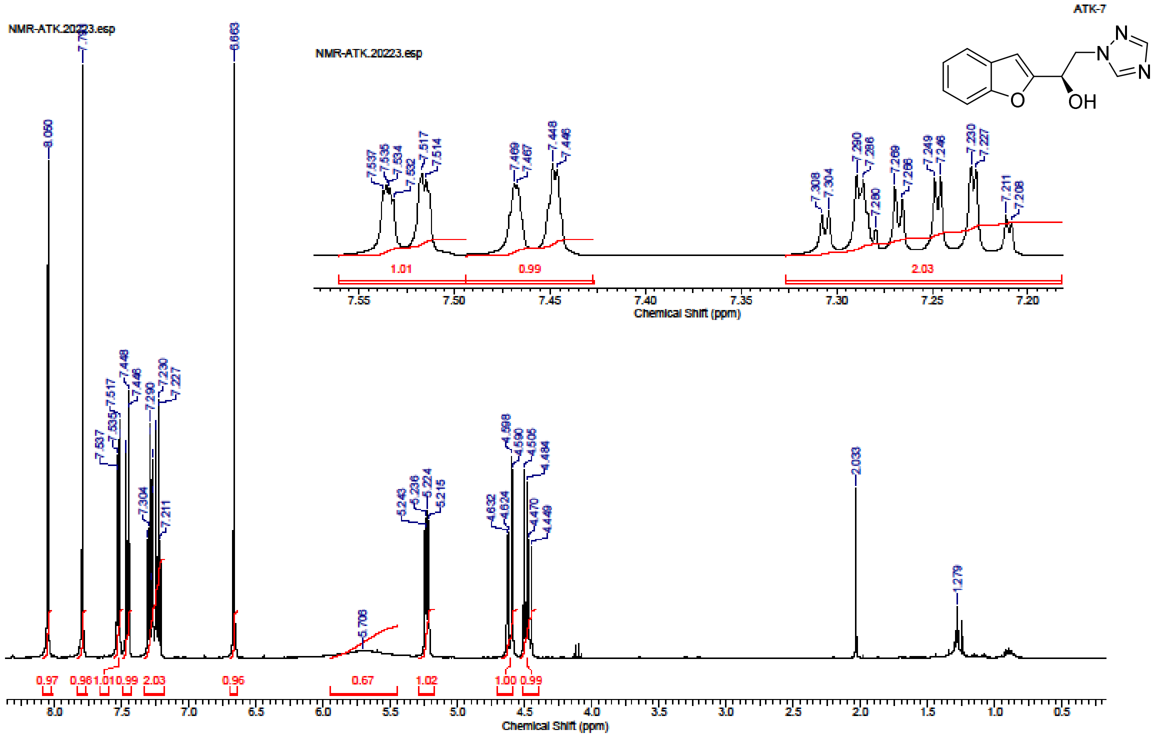

(**a**)

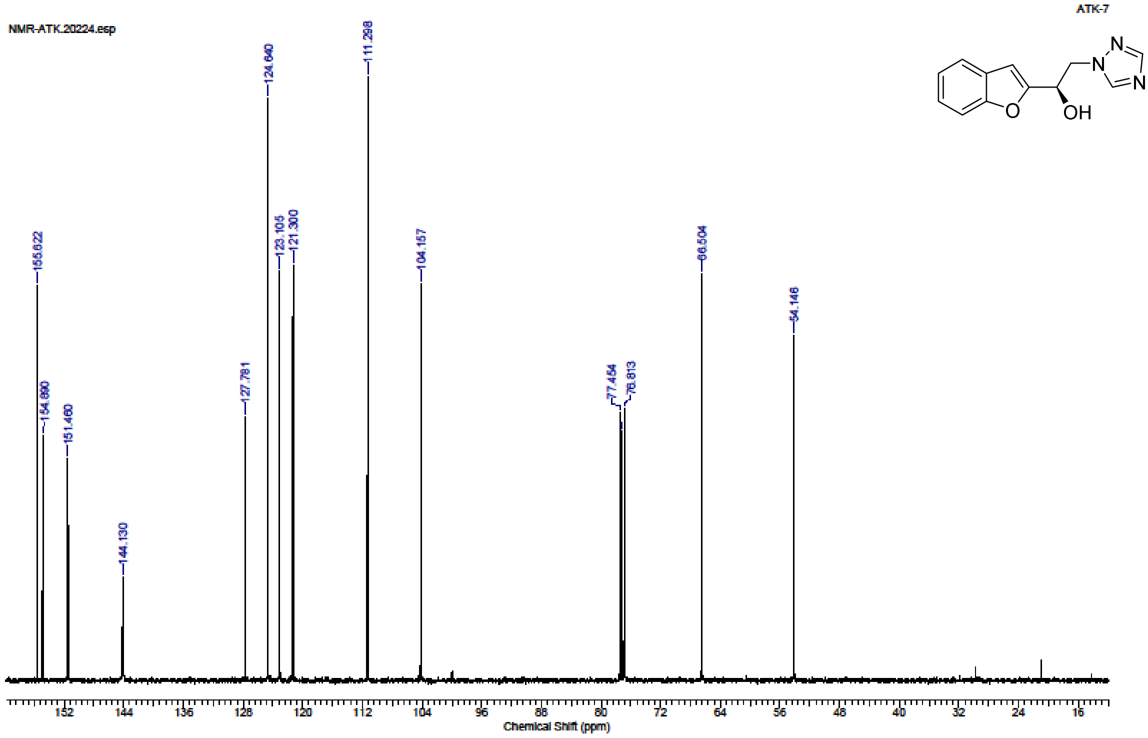

(**b**)

Figure 13. (**a**) ^{1}H NMR and (**b**) ^{13}C NMR spectra of (*R*)-1-(benzofuran-2-yl)-2-(1*H*-1,2,4-triazol-1-yl)ethan-1-ol (**17**).

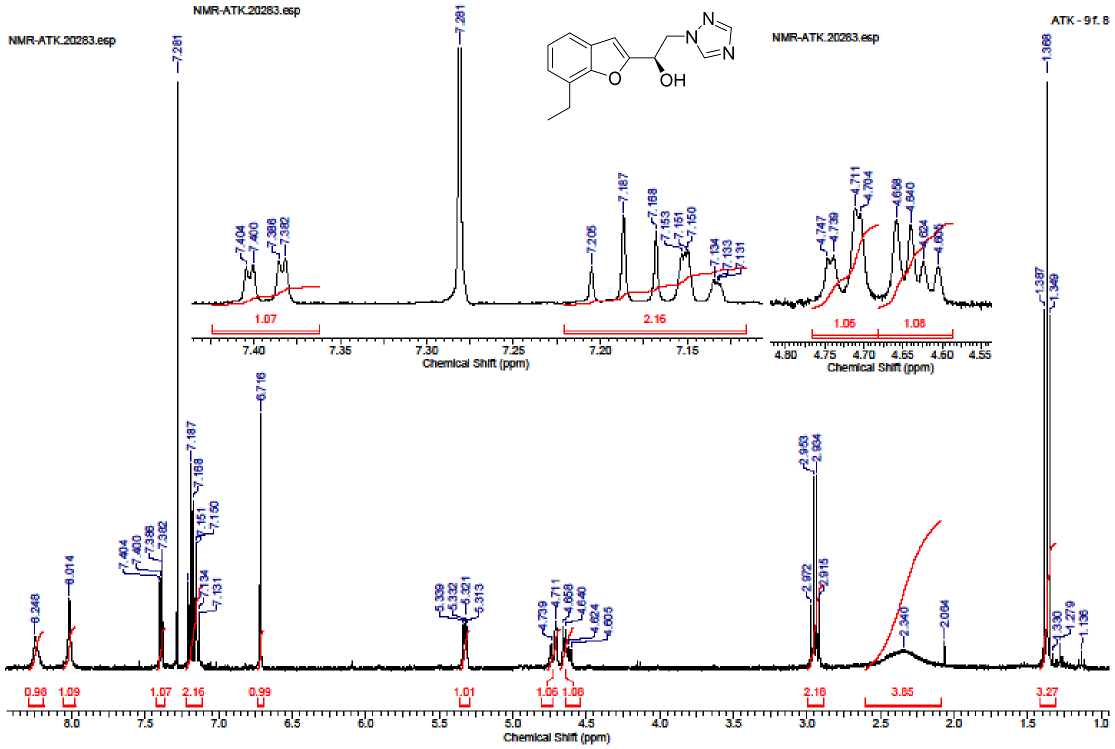

(**a**)

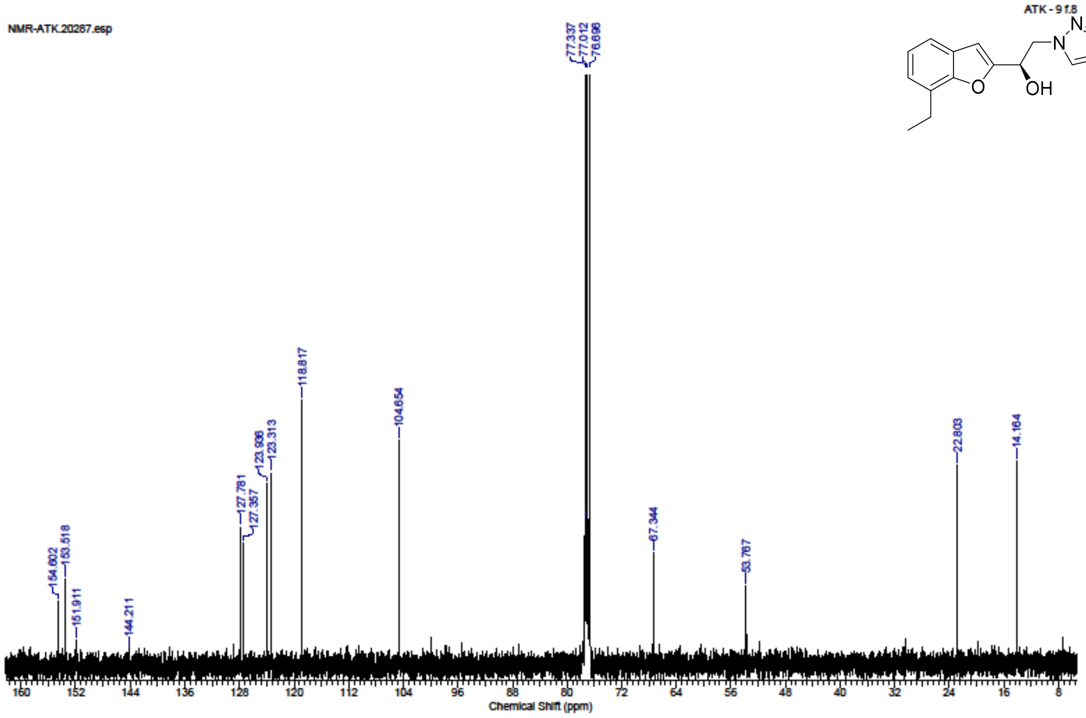

(**b**)

Figure 14. (**a**) ¹H NMR and (**b**) ¹³C NMR spectra of (*R*)-1-(7-ethylbenzofuran-2-yl)-2-(1*H*-1,2,4-triazol-1-yl)ethan-1-ol (**18**).

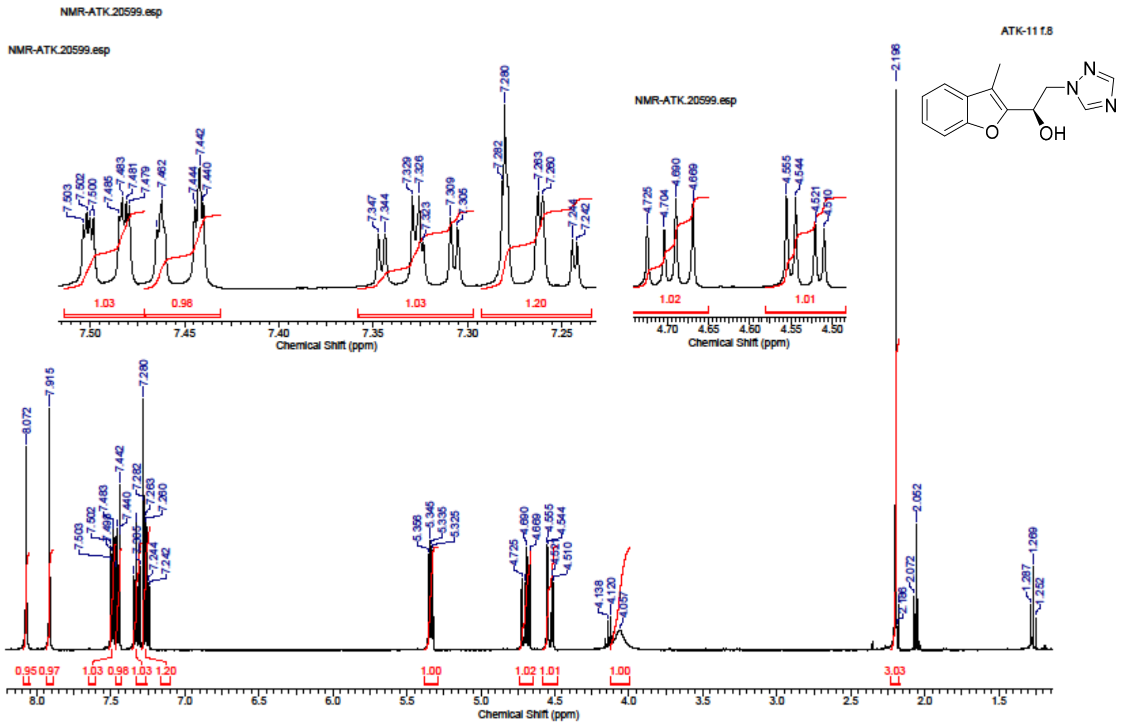

(**a**)

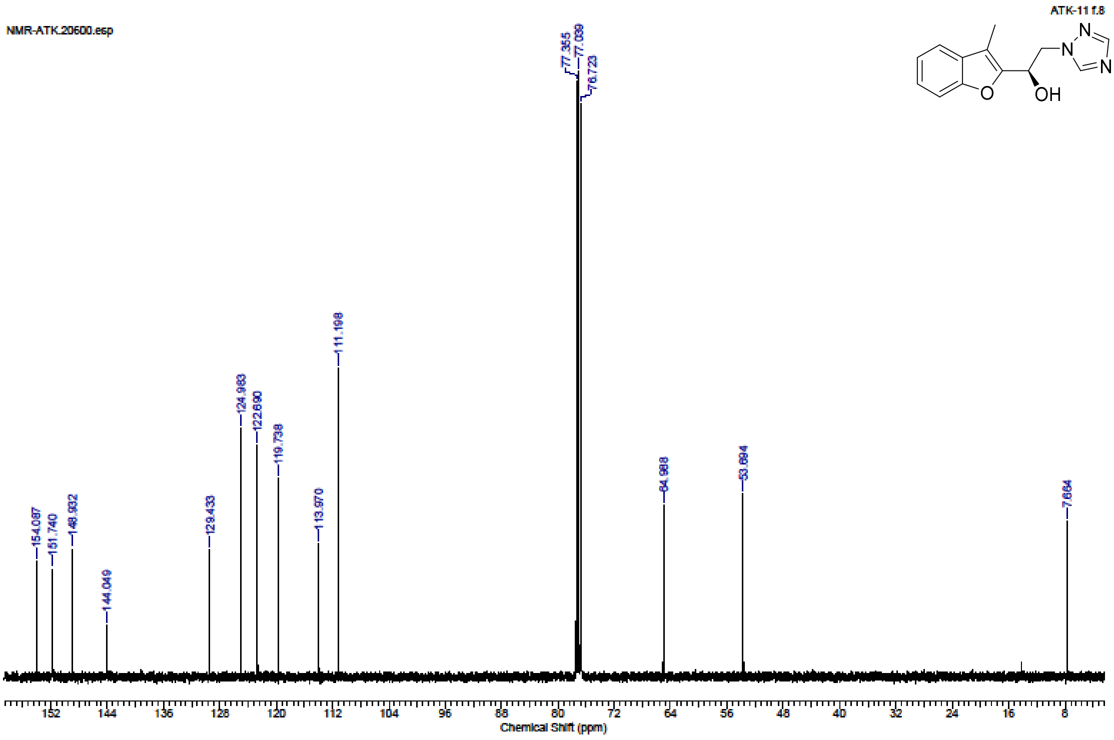

(**b**)

Figure 15. (**a**) ^{1}H NMR and (**b**) ^{13}C NMR spectra of (*R*)-1-(3-methylbenzofuran-2-yl)-2-(1*H*-1,2,4-triazol-1-yl)ethan-1-ol (**19**).

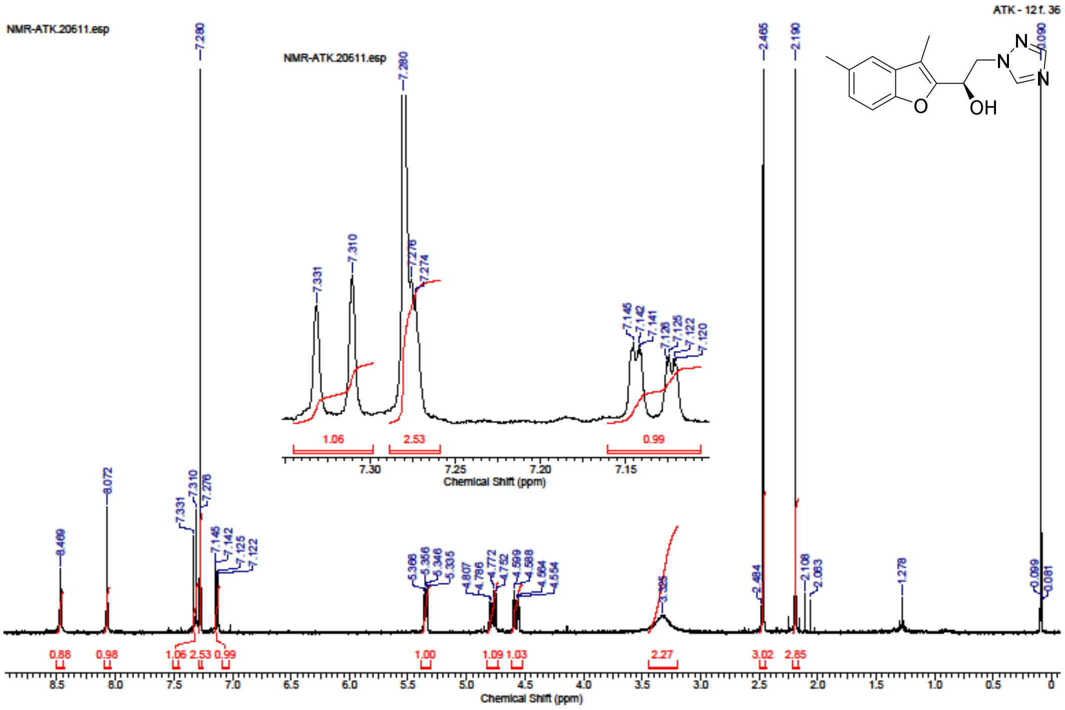

(**a**)

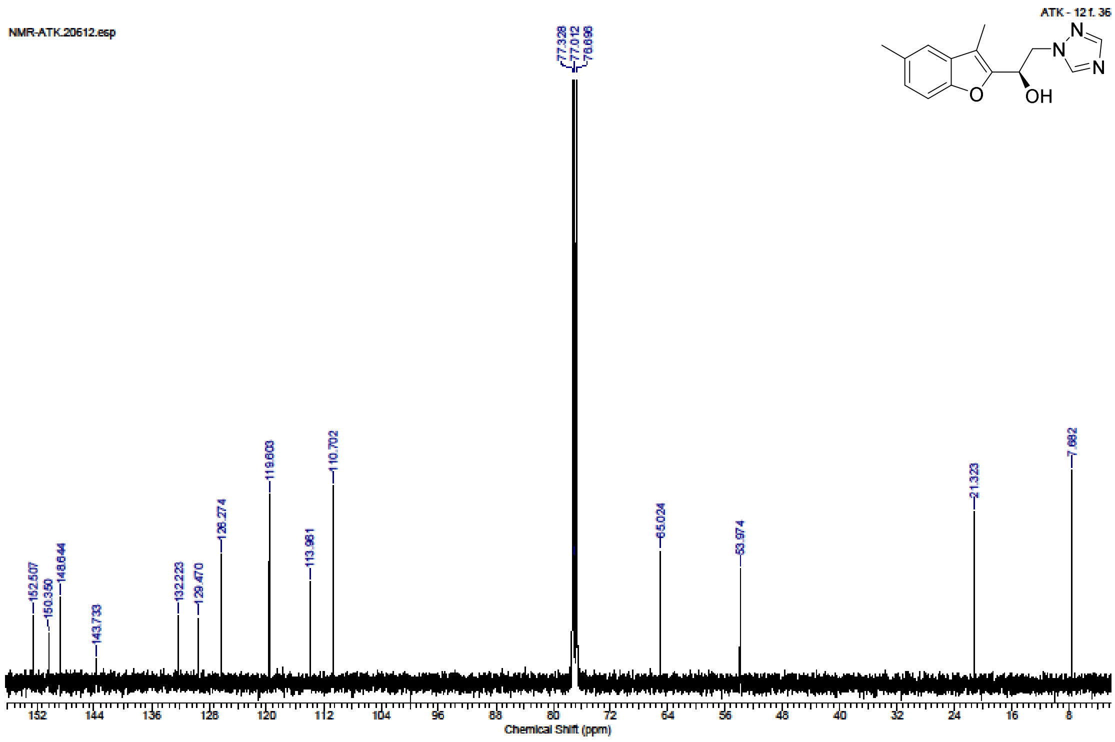

(**b**)

Figure 16. (**a**) ^{1}H NMR and (**b**) ^{13}C NMR spectra of (*R*)-1-(3,5-dimethylbenzofuran-2-yl)-2-(1H-1,2,4-triazol-1-yl)ethan-1-ol (**20**).

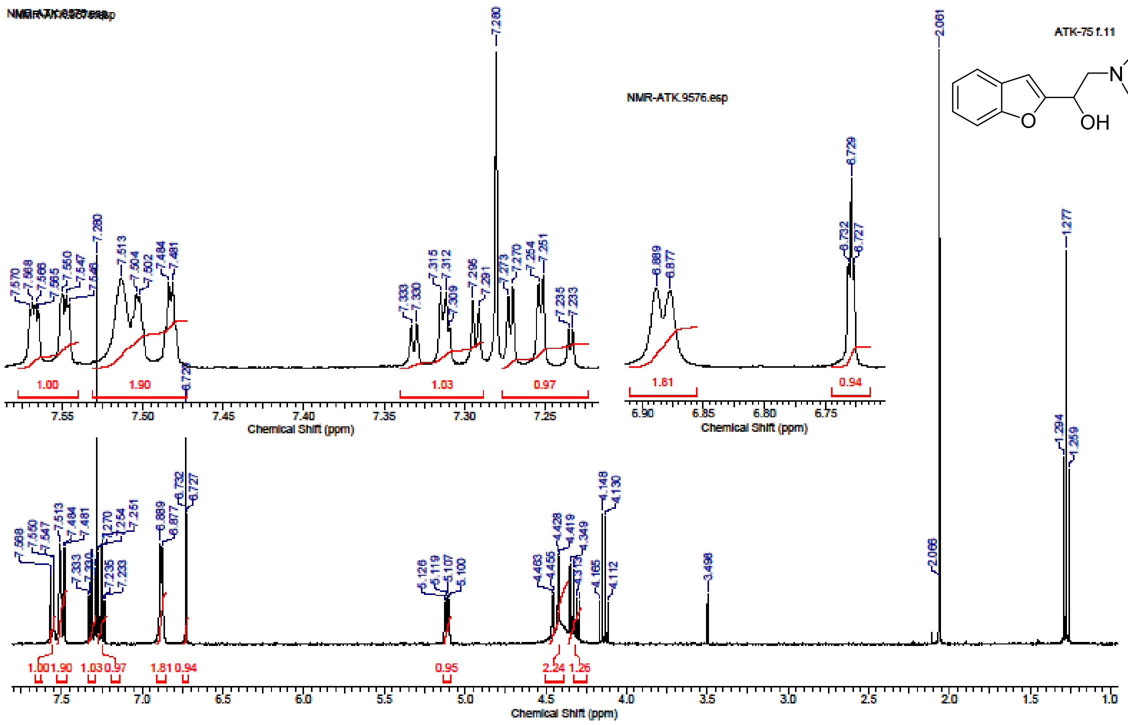

NMR-ATK.9576.esp
ATK-75 f.11
OH
O
N
N
1.00
1.90
1.03
0.97
1.81
0.94
0.95
2.24
1.26
Chemical Shift (ppm)
7.280
2.061
1.277
6.729
6.732
6.727
6.889
6.877
5.126
5.119
5.107
5.100
4.463
4.455
4.428
4.419
4.349
4.319
4.165
4.148
4.130
4.112
3.498
2.068
1.294
1.259

(**a**)

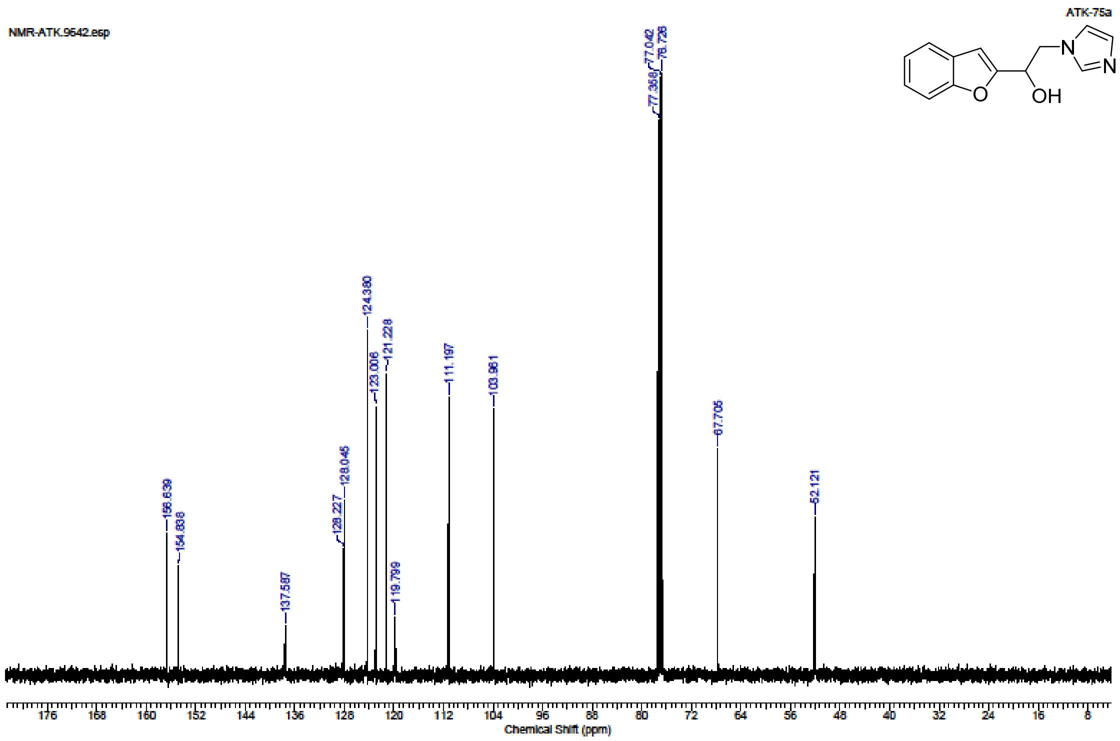

NMR-ATK.9642.esp
ATK-75a
OH
O
N
N
77.042
77.358
76.726
155.639
154.838
137.587
128.227
128.045
124.380
123.006
121.228
119.799
111.197
103.961
67.705
52.121
Chemical Shift (ppm)

(**b**)

Figure 17. (**a**) ^{1}H NMR and (**b**) ^{13}C NMR spectra of 1-(benzofuran-2-yl)-2-(1*H*-imidazol-1-yl)ethan-1-ol (**21**).

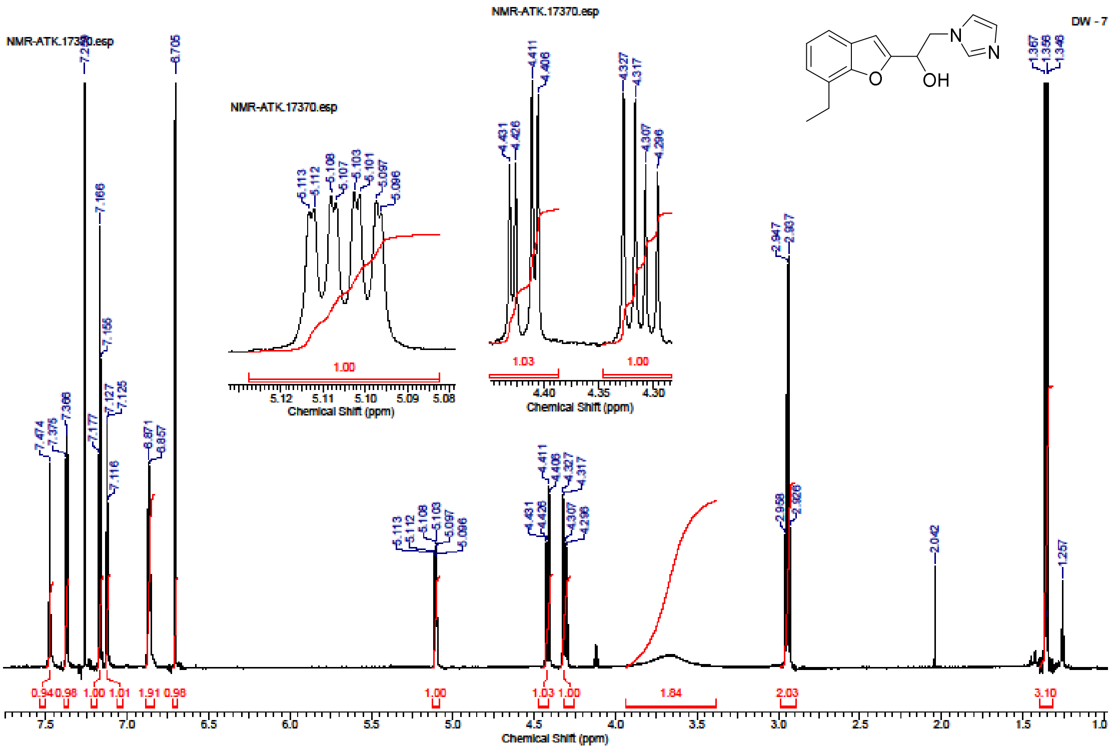

(**a**)

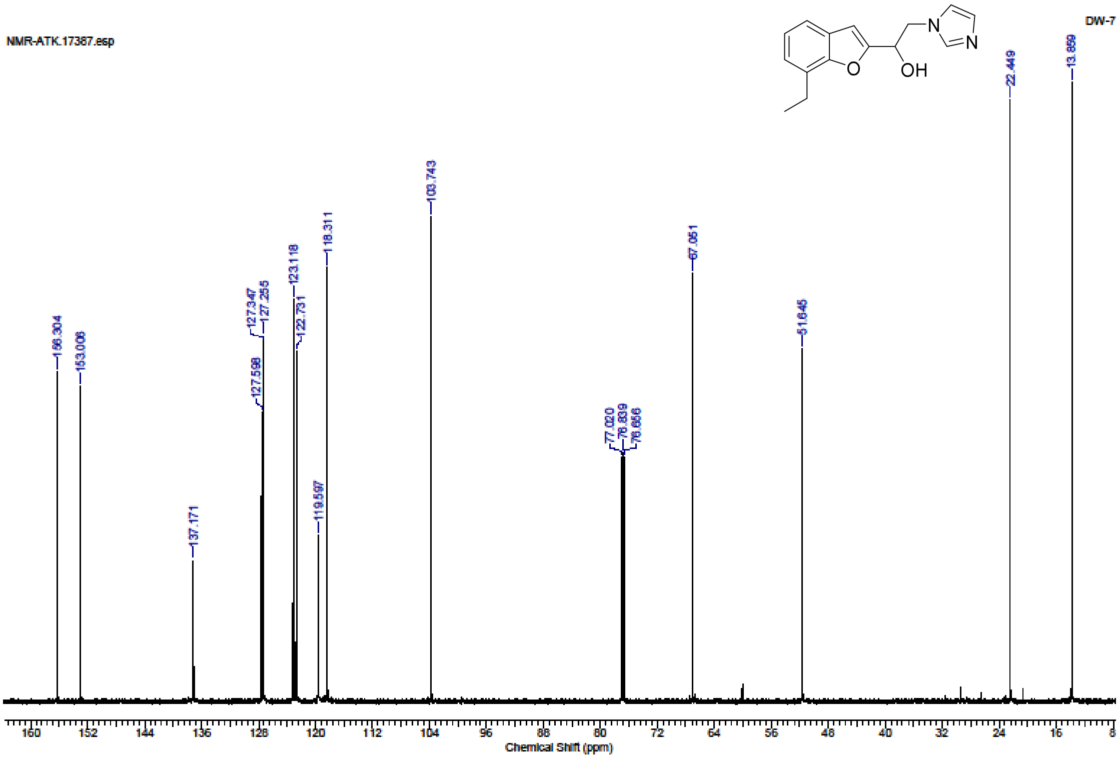

(**b**)

Figure 18. (**a**) ^{1}H NMR and (**b**) ^{13}C NMR spectra of 1-(7-ethylbenzofuran-2-yl)-2-(1*H*-imidazol-1-yl)ethan-1-ol (**22**).

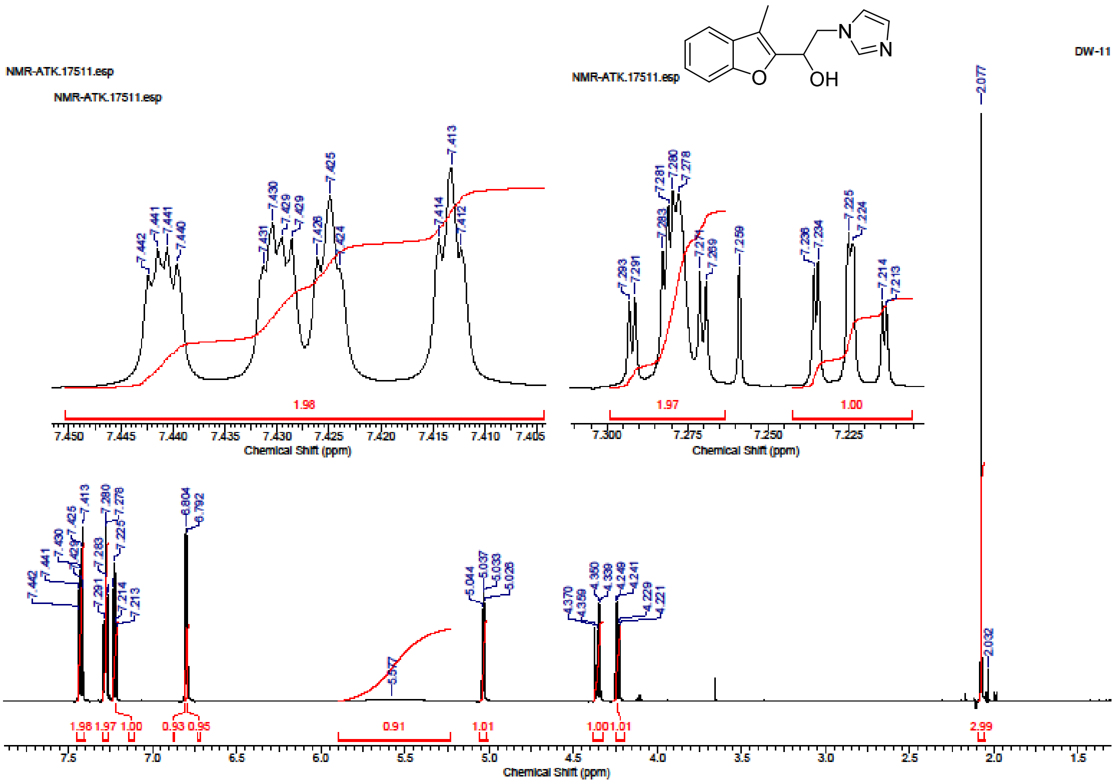

.

(**a**)

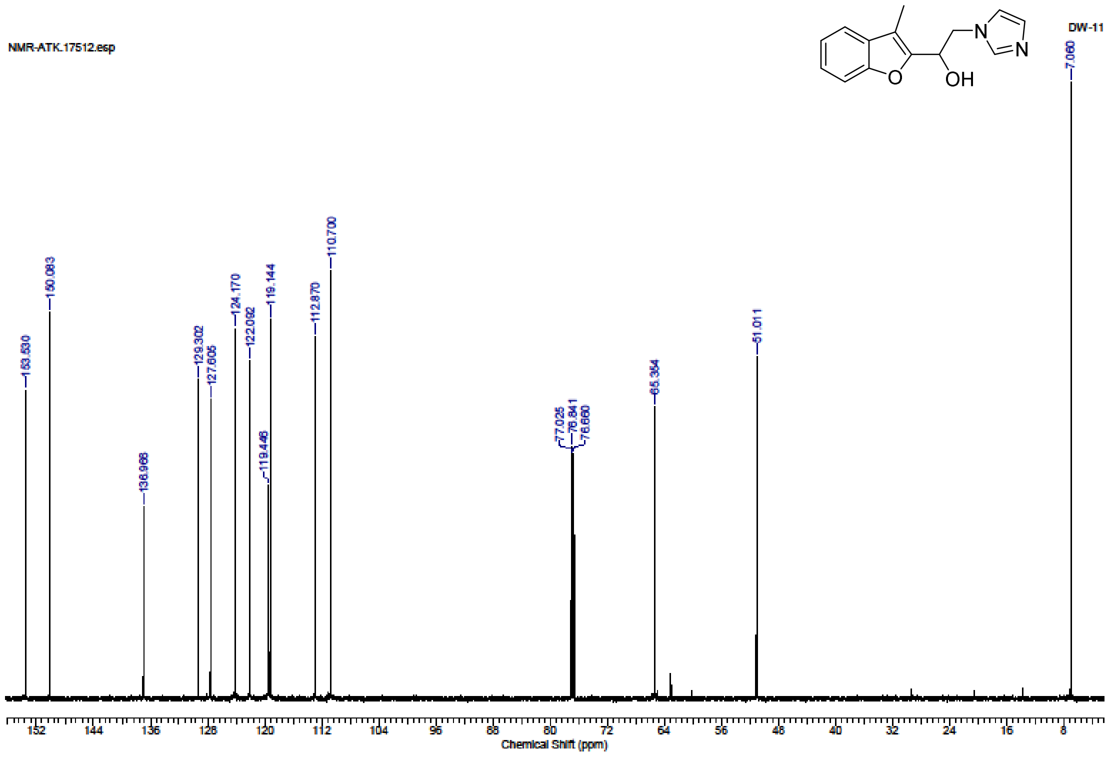

(**b**)

Figure S19. (**a**) ^{1}H NMR and (**b**) ^{13}C NMR spectra of 2-(1*H*-imidazol-1-yl)-1-(3-methylbenzofuran-2-yl)ethan-1-ol (**23**).

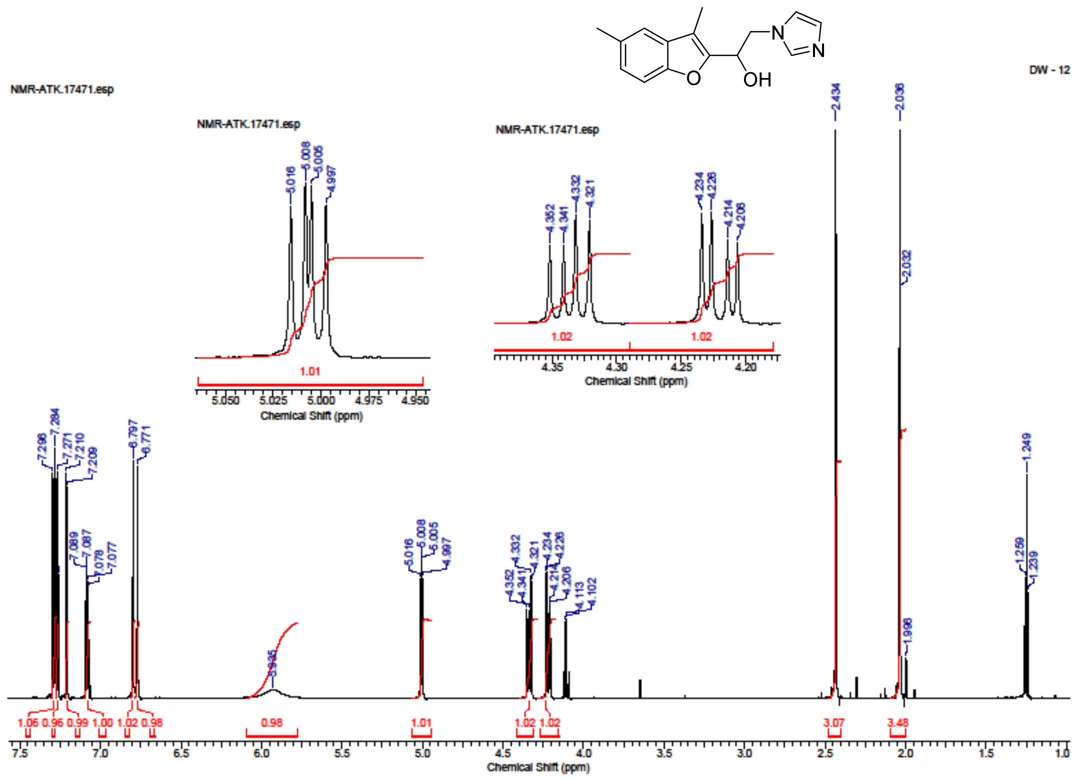

(**a**)

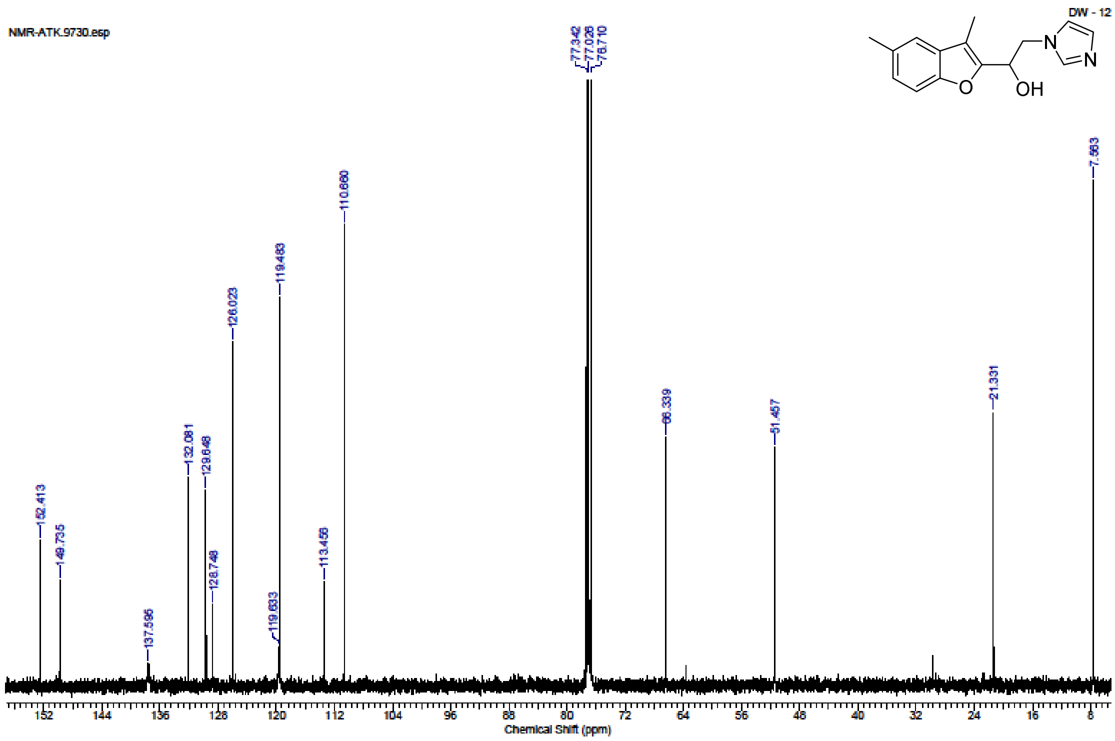

(**b**)

Figure 20. (a) ^{1}H NMR and (b) ^{13}C NMR spectra of 1-(3,5-dimethylbenzofuran-2-yl)-2-(1*H*-imidazol-1-yl)ethan-1-ol (24).

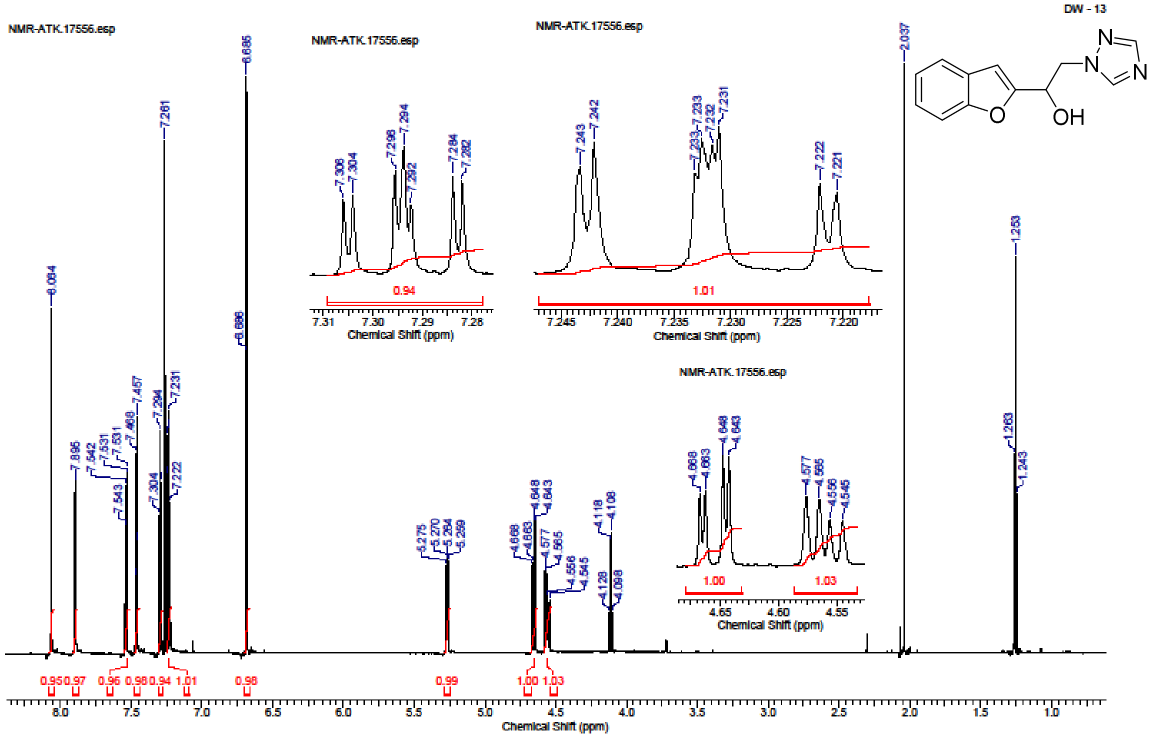
NMR-ATK.17556.esp
DW - 13
OH
Chemical Shift (ppm)

(**a**)

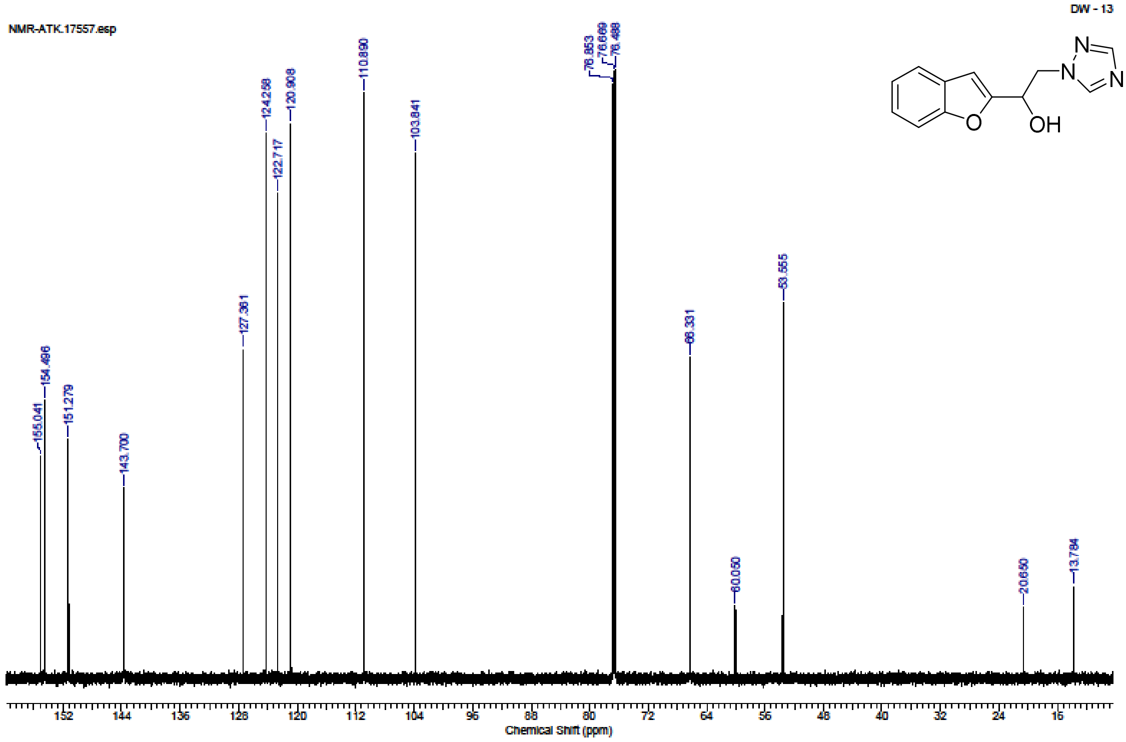
NMR-ATK.17557.esp
DW - 13
OH
Chemical Shift (ppm)

(**b**)

Figure 21. (**a**) ^{1}H NMR and (**b**) ^{13}C NMR spectra of 1-(benzofuran-2-yl)-2-(1*H*-1,2,4-triazol-1-yl)ethan-1-ol (**25**).

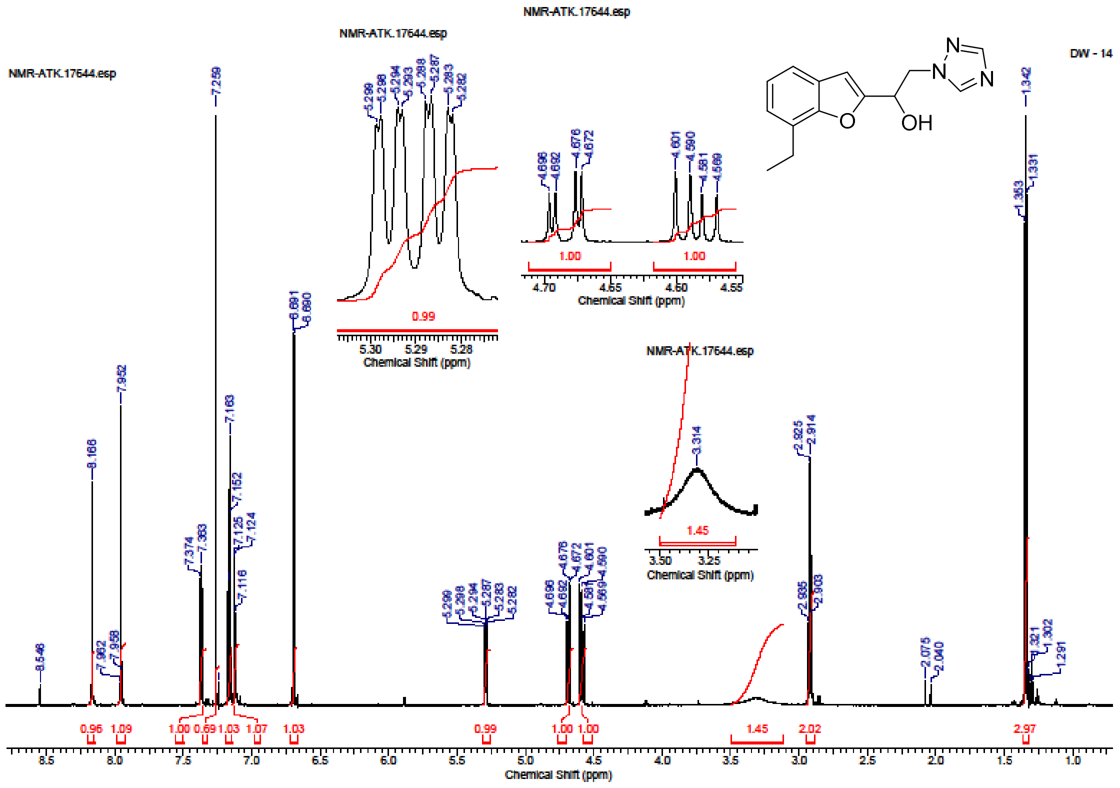

(**a**)

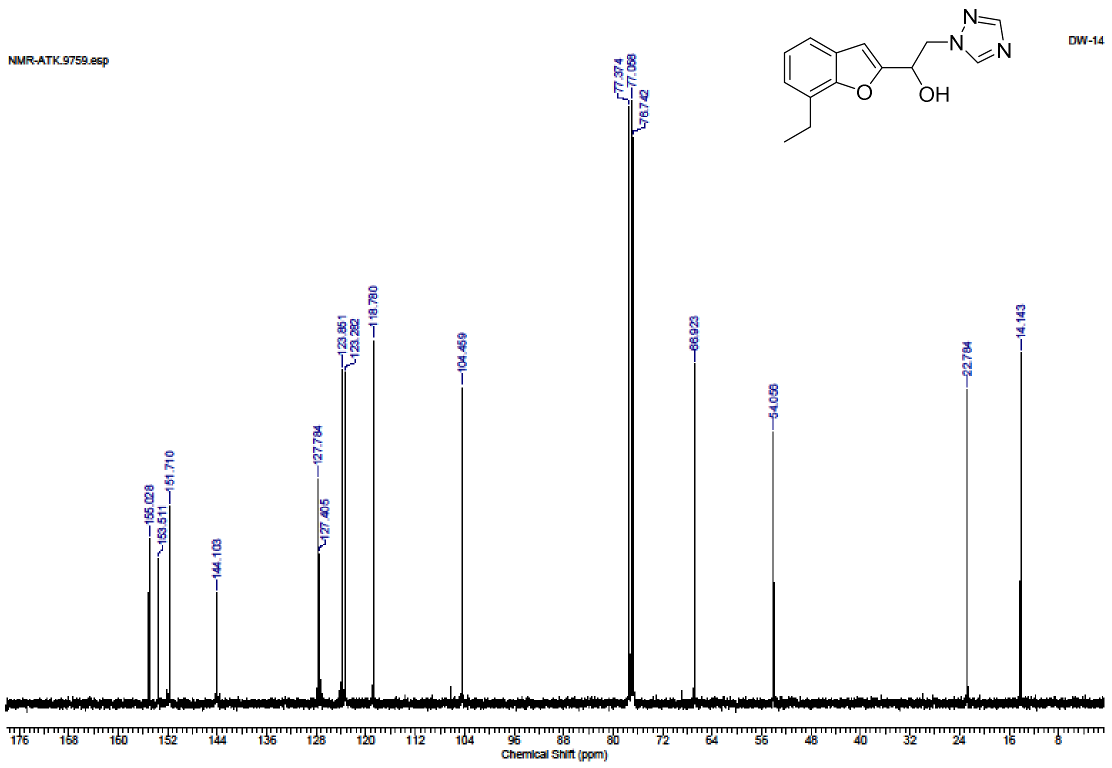

(**b**)

Figure 22. (**a**) ¹H NMR and (**b**) ¹³C NMR spectra of 1-(7-ethylbenzofuran-2-yl)-2-(1*H*-1,2,4-triazol-1-yl)ethan-1-ol (**26**).

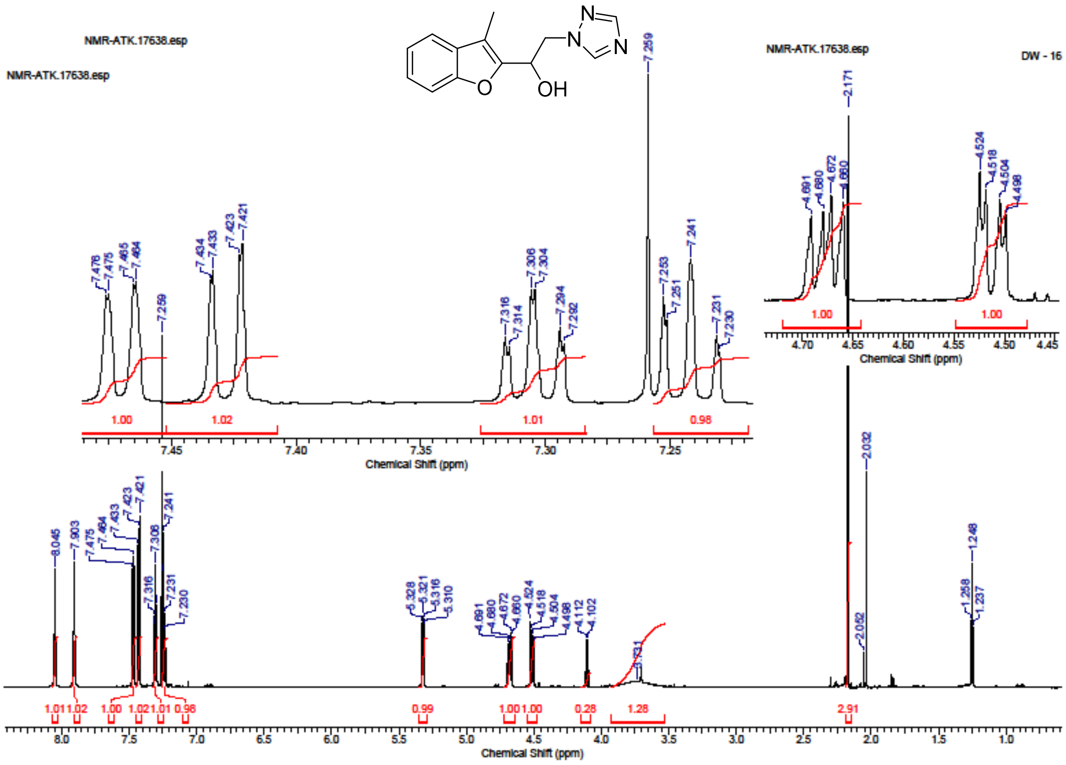

(**a**)

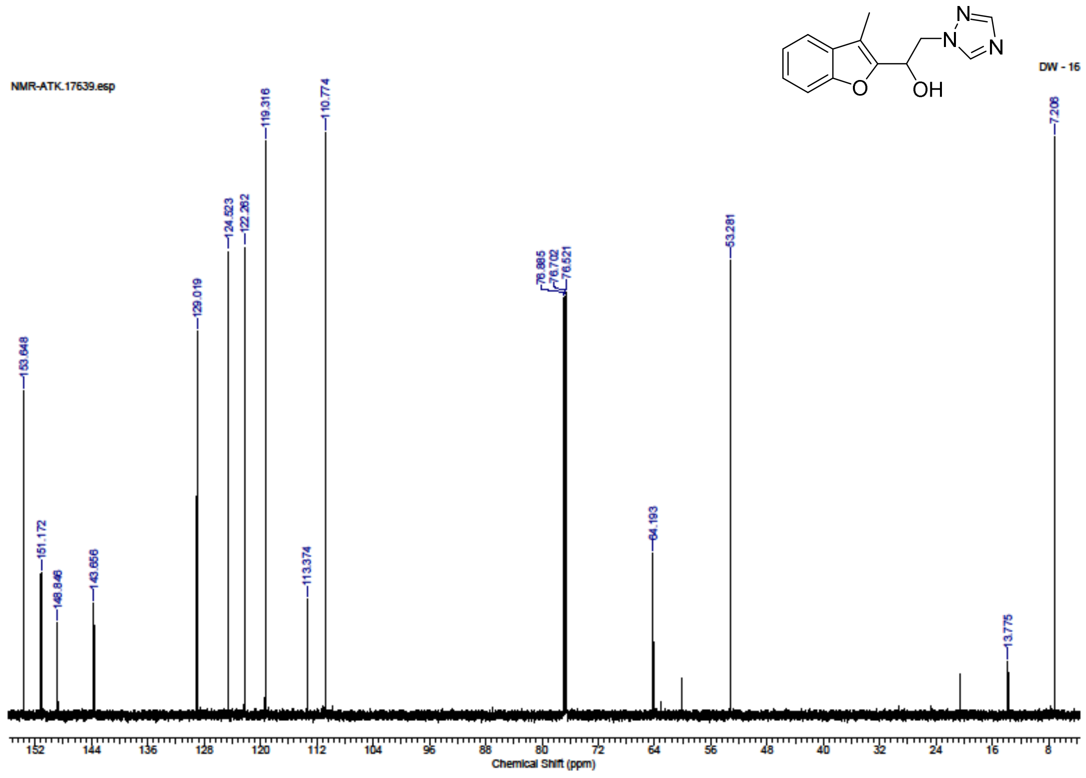

(**b**)

Figure S23. (**a**) ^{1}H NMR and (**b**) ^{13}C NMR spectra of 1-(3-methylbenzofuran-2-yl)-2-(1*H*-1,2,4-triazol-1-yl)ethan-1-ol (**27**).

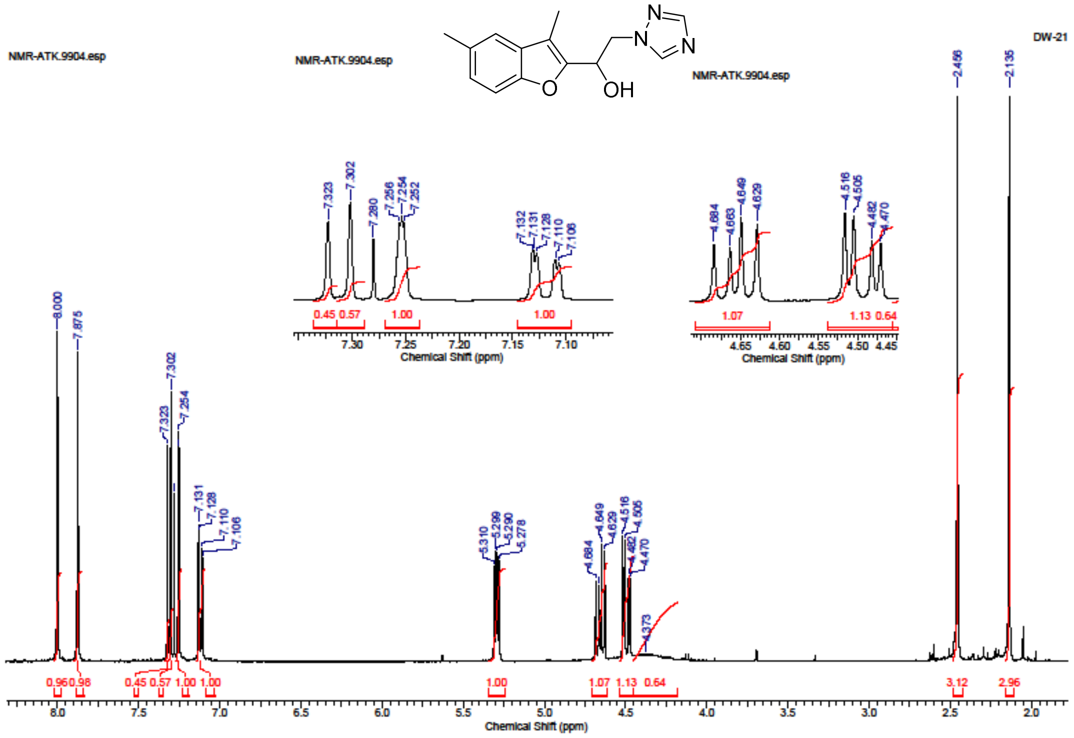

(**a**)

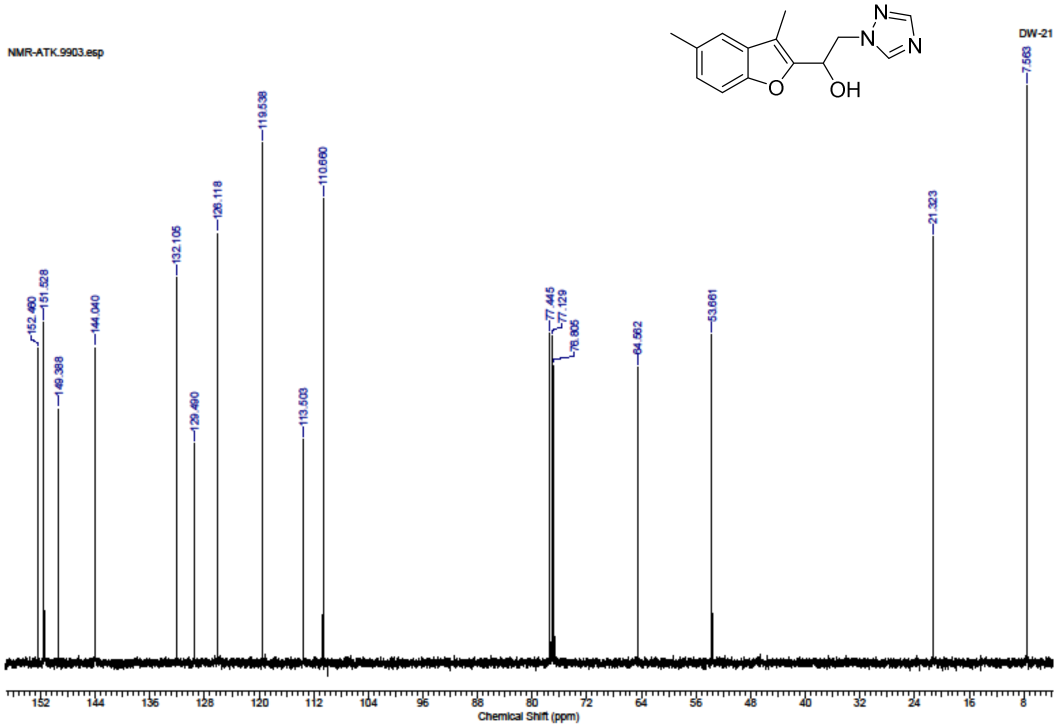

(**b**)

Figure 24. (**a**) ^{1}H NMR and (**b**) ^{13}C NMR spectra of 1-(3,5-dimethylbenzofuran-2-yl)-2-(1*H*-1,2,4-triazol-1-yl)ethan-1-ol (**28**).

2. Calculations Details

The theoretical approach that has been used in this work is common to all studied structures and includes (i) conformational search at molecular mechanics level (MM3); (ii) pre-optimization at the B3LYP/6-31G(d) level to reduce the number of thermally accessible conformers; (iii) parallel re-optimization of conformers found at low-DFT level at B3LYP/6-311++G(d,p) level and with the use of the IEFPCM solvent model, respectively, for acetonitrile and chloroform, followed by frequency calculations to confirm stability of received structures; (iv) calculations on relative energies (ΔE_{DFT} and $\Delta\Delta G_{DFT}$) using Boltzmann distribution at T = 298.15 K; (v) rotatory strengths calculations at the TD-DFT/6-311++G(d,p) level for all stable conformers of relative energies ranging from 0.0 to 2.0 kcal mol^{-1}; (vi) optical rotation calculations at the IEFPCM($CHCl_3$)/B3LYP/*aug*-cc-pVTZ level for all stable conformers of relative energies ranging from 0.0 to 2.0 kcal mol^{-1}.

Preliminary conformer distribution search was performed by the Scigress package [1] using the MM3 molecular mechanics force field. All possible conformers were analyzed using the systematic search methodology. Minimum energy conformers of relative steric energies (ΔE_{SE}) up to 10 kcal mol^{-1} found by molecular mechanics were further fully optimized at the B3LYP/6-31G(d) level as implemented in the Gaussian09 package [2], which significantly reduced the number of conformers. Higher accuracy calculations were performed at the IEFPCM/B3LYP/6-311++G(d,p) level [3].

The conformers obtained at the DFT/6-311++G(d,p) level were the real minima (no imaginary frequencies have been found). Total and free energy values have been calculated and used to obtain the Boltzmann population of conformers at 298.15 K. Only the results for conformers that differ from the most stable one by less than 2 kcal mol^{-1} have been taken into account for further calculations, following a generally accepted protocol [4].

The TD-DFT/6-311++G(2d,2p) calculations of ECD were performed for all structures re-optimized at higher levels of theory. We used three different density functionals for calculations of rotatory strengths, namely CAM-B3LYP [5], M06-2X [6] and ωB97-XD [7] functional. Rotatory

strengths were calculated using both length and velocity representations. In the present study, the differences between the length and velocity representations of the calculated values of rotatory strengths were quite small, and for this reason only the velocity representations were further used. The CD spectra were simulated by overlapping Gaussian functions for each transition according to the procedure previously described and using half band width 0.4 eV [8]. It should be noted, that there are not substantial differences between ECD spectra calculated with these three functionals for the same molecule, therefore we discussed here only results obtained with the use of ωB97-XD functional.

The cartesian coordinates and structures of individual conformers of **13–20** calculated at the various levels of theory are available on request (300 pages) from the authors.

Table S1. Total (E, in Hartree) and relative energies (*ΔE*, *ΔΔG*, in kcal mol^{-1}), percentage populations (Pop.) and number of imaginary frequencies calculated at the IEFPCM(MeCN)/B3LYP/6-311++G(d,p) level for low-energy conformers of **13**.

Conformer [a]	E	*ΔE*	Pop.	*ΔΔG*	Pop.	#ImFreq
1	−762.756385	1.14	2.93	1.61	1.09	0
3	−762.757133	0.67	6.48	0.63	5.81	0
4	−762.756378	1.14	2.91	1.45	1.46	0
6	−762.756791	0.89	4.51	0.53	6.81	0
14	−762.757456	0.47	9.12	0.27	10.66	0
16	−762.756425	1.11	3.06	1.09	2.64	0
18	−762.757051	0.72	5.94	0.69	5.18	0
20	−762.75602	1.37	1.99	1.47	1.4	0
29	−762.757585	0.39	10.45	0.00	16.73	0
32	−762.756444	1.10	3.12	1.14	2.45	0
33	−762.755814	1.50	1.6	1.33	1.77	0
34	−762.757362	0.53	8.26	0.62	5.89	0
35	−762.755439	1.73	1.08	1.66	1.01	0
39	−762.755962	1.41	1.87	1.17	2.33	0
43	−762.755724	1.55	1.45	1.73	0.9	0
44	−762.755417	1.75	1.05	1.74	0.88	0
46	−762.756699	0.94	4.09	1.23	2.11	0
51	−762.756741	0.92	4.27	0.59	6.14	0
52	−762.756414	1.12	3.02	0.51	7.08	0
53	−762.758202	0.00	20.09	0.05	15.45	0
60	−762.756313	1.18	2.72	1.2	2.21	0

[a] conformers are numbered according to their appearance during conformational search.

Table S2. Total (E, in Hartree) and relative energies (*ΔE*, *ΔΔG*, in kcal mol^{-1}), percentage populations (Pop.) and number of imaginary frequencies calculated at the IEFPCM($CHCl_3$)/B3LYP/6−311++G(d,p) level for low-energy conformers of **13**.

Conformer [a]	E	*ΔE*	Pop.	*ΔΔG*	Pop.	#ImFreq
1	−762.752203	0.56	4.53	1.18	1.94	0
3	−762.752527	0.36	6.39	0.58	5.32	0
4	−762.752303	0.50	5.04	1.15	2.03	0
6	−762.752317	0.49	5.11	0.80	3.69	0
14	−762.752261	0.52	4.82	0.68	4.51	0
16	−762.751182	1.20	1.54	1.34	1.47	0
18	−762.752022	0.67	3.74	0.80	3.66	0
20	−762.750943	1.35	1.19	1.81	0.66	0
26	−762.74997	1.96	0.42	2.50	0	0
29	−762.753095	0.00	11.66	0.00	14.23	0

32	−762.750624	1.55	0.85	1.37	1.41	0
33	−762.751114	1.24	1.43	1.27	1.68	0
35	−762.750874	1.39	1.11	1.57	1.01	0
39	−762.750318	1.74	0.61	1.89	0.58	0
40	−762.753024	0.04	10.81	0.27	8.98	0
43	−762.751167	1.21	1.51	1.57	1.01	0
44	−762.750897	1.38	1.13	1.53	1.07	0
46	−762.752089	0.63	4.02	1.18	1.95	0
51	−762.752571	0.33	6.69	0.15	11.11	0
52	−762.752289	0.51	4.96	0.28	8.82	0
53	−762.753087	0.01	11.56	0.03	13.44	0
54	−762.752863	0.15	9.12	0.25	9.37	0
60	−762.751302	1.13	1.74	1.15	2.06	0

[a] conformers are numbered according to their appearance during conformational search.

Table S3. Total (E, in Hartree) and relative energies (*ΔE*, *ΔΔG*, in kcal mol^{-1}), percentage populations (Pop.) and number of imaginary frequencies calculated at the IEFPCM(MeCN)/B3LYP/6-311++G(d,p) level for low-energy conformers of **14**.

Conformer [a]	E	*ΔE*	Pop.	*ΔΔG*	Pop.	#ImFreq
1	−841.4092792	0.71	2.86	1.40	1.15	0
2	−841.4093205	0.68	2.99	0.74	3.49	0
3	−841.408859	0.97	1.83	0.73	3.59	0
4	−841.4090019	0.88	2.13	1.28	1.41	0
5	−841.4097618	0.41	4.77	0.56	4.71	0
6	−841.4097633	0.40	4.78	0.66	4.02	0
7	−841.4086642	1.09	1.49	1.02	2.19	0
8	−841.4087367	1.05	1.61	1.00	2.24	0
9	−841.4077823	1.65	0.59	0.96	2.4	0
10	−841.4096896	0.45	4.42	0.71	3.66	0
11	−841.4096811	0.46	4.38	0.79	3.2	0
12	−841.4074238	1.87	0.4	1.98	0.43	0
14	−841.409473	0.59	3.52	0.70	3.75	0
15	−841.4079711	1.53	0.72	1.70	0.69	0
16	−841.4095086	0.56	3.65	0.88	2.76	0
17	−841.4076186	1.75	0.49	1.97	0.44	0
18	−841.4076186	1.75	0.49	2.26	0	0
19	−841.4074977	1.83	0.43	2.70	0	0
20	−841.4088512	0.98	1.82	0.87	2.81	0
21	−841.4089465	0.92	2.01	1.19	1.63	0
22	−841.4088784	0.96	1.87	0.77	3.32	0
23	−841.4088876	0.95	1.89	0.97	2.36	0
24	−841.4083488	1.29	1.07	2.15	0	0
25	−841.4104077	0.00	9.47	0.17	9.15	0
26	−841.4079246	1.56	0.68	2.16	0	0
27	−841.4103746	0.02	9.14	0.00	12.22	0
28	−841.4092667	0.72	2.83	1.12	1.83	0
29	−841.4092517	0.73	2.78	1.20	1.6	0
30	−841.4085481	1.17	1.32	0.72	3.62	0
31	−841.4083761	1.27	1.1	2.13	0	0
32	−841.4085795	1.15	1.36	1.15	1.76	0
33	−841.4085	1.18	1.29	1.64	0.76	0
34	−841.4085521	1.16	1.33	1.75	0.64	0

35	−841.4101	0.22	6.56	0.39	6.36	0
36	−841.4101	0.21	6.65	0.53	4.99	0
37	−841.4078	1.65	0.59	1.64	0.76	0
38	−841.4079702	1.53	0.72	1.07	2	0
39	−841.4083705	1.28	1.09	1.56	0.88	0
40	−841.4084726	1.21	1.22	1.29	1.38	0
41	−841.4080105	1.50	0.75	1.39	1.17	0
42	−841.4081858	1.39	0.9	1.73	0.65	0

[a] conformers are numbered according to their appearance during conformational search.

Table S4. Total (E, in Hartree) and relative energies (*ΔE*, *ΔΔG*, in kcal mol^{-1}), percentage populations (Pop.) and number of imaginary frequencies calculated at the IEFPCM($CHCl_3$)/B3LYP/6-311++G(d,p) level for low-energy conformers of **14**.

Conformer [a]	E	*ΔE*	Pop.	*ΔΔG*	Pop.	#ImFreq
1	−841.404568	0.53	2.73	0.75	2.18	0
2	−841.4046253	0.50	2.9	0.25	5.1	0
3	−841.4043308	0.68	2.12	0.94	1.59	0
4	−841.404419	0.63	2.33	0.68	2.44	0
5	−841.4052385	0.11	5.56	0.05	7.13	0
6	−841.4052739	0.09	5.77	0.24	5.17	0
7	−841.404065	0.85	1.6	1.25	0.94	0
8	−841.4041139	0.82	1.69	0.78	2.09	0
9	−841.4032474	1.36	0.67	1.41	0.72	0
10	−841.4044759	0.59	2.48	0.68	2.45	0
11	−841.4044657	0.6	2.45	0.69	2.4	0
12	−841.4030072	1.51	0.52	1.22	0.98	0
13	−841.4028025	1.64	0.42	1.77	0.39	0
14	−841.405157	0.16	5.1	0.00	7.74	0
15	−841.4033826	1.28	0.78	1.36	0.78	0
16	−841.4052241	0.12	5.47	0.45	3.61	0
17	−841.4031676	1.41	0.62	1.56	0.56	0
18	−841.4032388	1.37	0.67	1.90	0.31	0
19	−841.4031231	1.44	0.59	2.43	0	0
20	−841.4042695	0.72	1.99	0.86	1.81	0
21	−841.4042805	0.71	2.01	0.46	3.56	0
22	−841.4046642	0.47	3.02	0.35	4.3	0
23	−841.4047078	0.44	3.17	0.59	2.84	0
24	−841.4040851	0.83	1.64	1.25	0.94	0
25	−841.405415	0.00	6.7	0.30	4.69	0
26	−841.4035105	1.20	0.89	1.90	0.31	0
27	−841.4054154	0.00	6.7	0.07	6.85	0
28	−841.4042273	0.75	1.9	0.47	3.53	0
29	−841.4042325	0.74	1.91	1.10	1.21	0
30	−841.4044084	0.63	2.31	0.43	3.74	0
31	−841.4042349	0.74	1.92	1.53	0.58	0
32	−841.4044565	0.60	2.43	0.82	1.95	0
33	−841.4043187	0.69	2.1	1.28	0.89	0
34	−841.4044428	0.61	2.39	1.11	1.19	0
35	−841.4053426	0.05	6.21	0.16	5.94	0
36	−841.405375	0.03	6.42	0.09	6.69	0
37	−841.4022158	2.01	0	1.69	0.45	0
38	−841.402473	1.85	0.3	2.15	0	0

39	−841.4026	1.75	0.35	1.56	0.55	0
40	−841.4028	1.61	0.44	1.81	0.36	0
41	−841.4025	1.81	0.32	1.62	0.5	0
42	−841.4027	1.68	0.4	1.59	0.53	0

[a] conformers are numbered according to their appearance during conformational search.

Table S5. Total (E, in Hartree) and relative energies (*ΔE*, *ΔΔG*, in kcal mol^{-1}), percentage populations (Pop.) and number of imaginary frequencies calculated at the IEFPCM(MeCN)/B3LYP/6-311++G(d,p) level for low-energy conformers of **15**.

**Conformer** [a]	**E**	***ΔE***	**Pop.**	***ΔΔG***	**Pop.**	**#ImFreq**
2	−802.086052	0.26	18.16	0.00	25.67	0
11	−802.085808	0.41	14.02	0.56	10.01	0
18	−802.086014	0.28	17.44	0.40	13.1	0
21	−802.08646	0.00	27.99	0.00	25.81	0
25	−802.084802	1.04	4.83	0.82	6.5	0
26	−802.08374	1.71	1.57	1.93	1	0
27	−802.084488	1.24	3.46	0.56	9.97	0
28	−802.083416	1.91	1.11	2.11	0	0
34	−802.083513	1.85	1.23	2.22	0	0
39	−802.08361	1.79	1.36	2.02	0	0
44	−802.083657	1.76	1.44	1.49	2.09	0
45	−802.083325	1.97	1.01	1.72	1.4	0
46	−802.084535	1.21	3.64	1.48	2.11	0
49	−802.084269	1.38	2.74	1.42	2.35	0

[a] conformers are numbered according to their appearance during conformational search.

Table S6. Total (E, in Hartree) and relative energies (*ΔE*, *ΔΔG*, in kcal mol^{-1}), percentage populations (Pop.) and number of imaginary frequencies calculated at the IEFPCM($CHCl_3$)/B3LYP/6-311++G(d,p) level for low-energy conformers of **15**.

**Conformer** [a]	**E**	***ΔE***	**Pop.**	***ΔΔG***	**Pop.**	**#ImFreq**
2	−802.081609	0.00	22.5	0.07	21.58	0
4	−802.078456	1.98	0.8	2.87	0	0
11	−802.081502	0.07	20.09	0.49	10.72	0
18	−802.081024	0.37	12.1	0.14	19.35	0
21	−802.081282	0.21	15.9	0.00	24.35	0
25	−802.080332	0.80	5.82	0.58	9.15	0
26	−802.079039	1.61	1.48	2.16	0	0
27	−802.080233	0.86	5.23	0.78	6.5	0
28	−802.078857	1.73	1.22	2.33	0	0
34	−802.079279	1.46	1.9	2.17	0	0
39	−802.079406	1.38	2.18	2.12	0	0
44	−802.079418	1.37	2.21	1.36	2.46	0
45	−802.079112	1.57	1.6	1.53	1.85	0
46	−802.079953	1.04	3.89	1.49	1.97	0
49	−802.079736	1.18	3.09	1.46	2.08	0

[a] conformers are numbered according to their appearance during conformational search.

Table S7. Total (E, in Hartree) and relative energies (*ΔE*, *ΔΔG*, in kcal mol^{-1}), percentage populations (Pop.) and number of imaginary frequencies calculated at the IEFPCM(MeCN)/B3LYP/6-311++G(d,p) level for low-energy conformers of **16**.

Conformer [a]	E	ΔE	Pop.	ΔΔG	Pop.	#ImFreq
2	−841.412875	0.33	11.61	0.47	11.19	0
9	−841.412566	0.52	8.36	0.00	24.76	0
15	−841.413289	0.07	17.99	0.41	12.38	0
16	−841.412838	0.35	11.16	0.60	9.05	0
22	−841.411558	1.16	2.87	1.36	2.51	0
23	−841.410499	1.82	0.94	1.82	1.14	0
25	−841.411259	1.34	2.09	1.27	2.89	0
35	−841.410267	1.97	0.73	2.17	0	0
38	−841.41037	1.90	0.82	2.46	0	0
39	−841.411393	1.26	2.41	1.64	1.56	0
40	−841.410446	1.85	0.88	1.86	1.06	0
43	−841.411125	1.43	1.82	1.70	1.41	0
46	−841.413293	0.07	18.06	0.50	10.64	0
48	−841.413401	0.00	20.26	0.09	21.41	0

[a] conformers are numbered according to their appearance during conformational search.

Table S8. Total (E, in Hartree) and relative energies (*ΔE*, *ΔΔG*, in kcal mol^{-1}), percentage populations (Pop.) and number of imaginary frequencies calculated at the IEFPCM($CHCl_3$)/B3LYP/6-311++G(d,p) level for low-energy conformers of **16**.

Conformer [a]	E	ΔE	Pop.	ΔΔG	Pop.	#ImFreq
2	−841.40849	0.08	15.59	0.36	11.26	0
9	−841.408313	0.19	12.92	0.23	13.96	0
15	−841.408156	0.29	10.94	0.29	12.69	0
16	−841.407944	0.42	8.74	0.43	10.07	0
22	−841.407118	0.94	3.64	0.84	4.99	0
23	−841.405844	1.74	0.94	1.96	0.75	0
24	−841.405643	1.87	0.76	1.95	0.77	0
25	−841.407019	1.00	3.28	0.60	7.54	0
35	−841.406071	1.60	1.2	1.96	0.75	0
38	−841.406193	1.52	1.37	2.18	0	0
39	−841.406801	1.14	2.6	1.42	1.9	0
40	−841.406217	1.50	1.4	1.49	1.68	0
43	−841.406597	1.27	2.1	1.56	1.5	0
46	−841.408557	0.04	16.74	0.35	11.44	0
48	−841.408615	0.00	17.79	0.00	20.71	0

[a] conformers are numbered according to their appearance during conformational search.

Table S9. Total (E, in Hartree) and relative energies (*ΔE*, *ΔΔG*, in kcal mol^{-1}), percentage populations (Pop.) and number of imaginary frequencies calculated at the IEFPCM(MeCN)/B3LYP/6-311++G(d,p) level for low-energy conformers of **17**.

Conformer [a]	E	ΔE	Pop.	ΔΔG	Pop.	#ImFreq
1	−778.7983727	1.23	4.67	0.90	5.12	0
2	−778.8003257	0.00	37	0.46	10.73	0
3	−778.8002247	0.06	33.25	0.00	23.36	0
4	−778.7989546	0.86	8.65	0.06	21.1	0
5	−778.7989768	0.85	8.86	0.27	14.81	0
6	−778.7969536	2.12	0	1.35	2.41	0
8	−778.7977453	1.62	2.4	1.20	3.05	0
10	−778.7973149	1.89	1.52	1.43	2.08	0
12	−778.7955512	3	0	1.95	0.87	0

13	−778.7975471	1.74	1.95	1.11	3.57	0
14	−778.7970899	2.03	0	0.96	4.6	0
15	−778.7974147	1.83	1.69	0.74	6.66	0
16	−778.7965866	2.35	0	1.58	1.63	0

[a] conformers are numbered according to their appearance during conformational search.

Table S10. Total (E, in Hartree) and relative energies (*ΔE*, *ΔΔG*, in kcal mol^{-1}), percentage populations (Pop.) and number of imaginary frequencies calculated at the IEFPCM($CHCl_3$)/B3LYP/6-311++G(d,p) level for low-energy conformers of **17**.

Conformer [a]	E	*ΔE*	Pop.	*ΔΔG*	Pop.	#ImFreq
1	−778.794189	1.48	3.54	1.18	5.11	0
2	−778.796516	0.02	41.66	0.42	18.45	0
3	−778.796549	0.00	43.15	0.00	37.29	0
4	−778.794863	1.06	7.23	0.15	28.79	0
5	−778.794397	1.35	4.41	0.76	10.36	0

[a] conformers are numbered according to their appearance during conformational search.

Table S11. Total (E, in Hartree) and relative energies (*ΔE*, *ΔΔG*, in kcal mol^{-1}), percentage populations (Pop.) and number of imaginary frequencies calculated at the IEFPCM(MeCN)/B3LYP/6-311++G(d,p) level for low-energy conformers of **18**.

Conformer [a]	E	*ΔE*	Pop.	*ΔΔG*	Pop.	#ImFreq
1	−857.4523288	0.07	14.22	0.46	5.35	0
2	−857.4504841	1.23	2.01	1.16	1.65	0
3	−857.4505381	1.19	2.13	0.57	4.46	0
4	−857.4524365	0.00	15.94	0.52	4.85	0
5	−857.4510023	0.90	3.49	0.73	3.38	0
6	−857.4523622	0.05	14.73	0.39	5.99	0
7	−857.4523523	0.05	14.58	0.04	10.98	0
8	−857.451149	0.81	4.07	0.15	8.97	0
9	−857.4512116	0.77	4.35	0.45	5.43	0
10	−857.4489901	2.16	0	1.49	0.95	0
11	−857.4511598	0.8	4.12	0	11.65	0
12	−857.451166	0.8	4.15	0.43	5.59	0
13	−857.4491445	2.07	0	1.52	0.9	0
16	−857.4497658	1.68	0.94	1.13	1.72	0
17	−857.4498376	1.63	1.01	0.97	2.27	0
20	−857.44943	1.89	0.66	0.97	2.25	0
21	−857.4495427	1.82	0.74	1.43	1.04	0
22	−857.4471969	3.29	0	1.95	0.43	0
27	−857.451166	0.80	4.15	0.43	5.59	0
28	−857.4496868	1.73	0.86	1.50	0.92	0
29	−857.4497668	1.68	0.94	1.20	1.54	0
30	−857.4493103	1.96	0.58	1.70	0.66	0
31	−857.4492873	1.98	0.57	1.58	0.81	0
32	−857.4496886	1.72	0.87	1.52	0.89	0
33	−857.4496396	1.76	0.82	1.01	2.13	0
35	−857.4486711	2.36	0	1.72	0.63	0

[a] conformers are numbered according to their appearance during conformational search.

Table S12. Total (E, in Hartree) and relative energies (ΔE, $\Delta\Delta G$, in kcal mol^{-1}), percentage populations (Pop.) and number of imaginary frequencies calculated at the IEFPCM($CHCl_3$)/B3LYP/6-311++G(d,p) level for low-energy conformers of **18**.

Conformer [a]	E	ΔE	Pop.	$\Delta\Delta G$	Pop.	#ImFreq
2	−857.446183	1.55	1.93	1.49	1.4	0
3	−857.446291	1.48	2.16	0.65	5.74	0
4	−857.448572	0.05	24.24	0.46	7.9	0
5	−857.446742	1.20	3.48	0.74	4.96	0
6	−857.448617	0.02	25.41	0.15	13.5	0
7	−857.448652	0.00	26.38	0.00	18.31	0
8	−857.44711	0.97	5.15	0.15	13.62	0
9	−857.446612	1.28	3.04	0.67	5.26	0
10	−857.44486	2.38	0	1.39	1.66	0
11	−857.446566	1.31	2.89	0.34	9.97	0
12	−857.447142	0.95	5.33	0.43	8.4	0
13	−857.445012	2.28	0	1.49	1.39	0
16	−857.445436	2.02	0	1.68	1.01	0
17	−857.445394	2.04	0	1.24	2.14	0
20	−857.444685	2.49	0	1.36	1.75	0
21	−857.444737	2.46	0	1.91	0.68	0
28	−857.444845	2.39	0	2.06	0	0
29	−857.445005	2.29	0	1.69	1	0
32	−857.444827	2.4	0	1.95	0.64	0
33	−857.444786	2.43	0	1.33	1.83	0

[a] conformers are numbered according to their appearance during conformational search.

Table S13. Total (E, in Hartree) and relative energies (ΔE, $\Delta\Delta G$, in kcal mol^{-1}), percentage populations (Pop.) and number of imaginary frequencies calculated at the IEFPCM(MeCN)/B3LYP/6-311++G(d,p) level for low-energy conformers of **19**.

Conformer [a]	E	ΔE	Pop.	$\Delta\Delta G$	Pop.	#ImFreq
1	−818.127403	0.39	15.74	0.78	9.6	0
2	−818.128022	0.00	30.34	0.00	36.08	0
3	−818.126227	1.13	4.53	0.94	7.35	0
4	−818.126339	1.06	5.1	2.02	0	0
5	−818.127208	0.51	12.81	1.33	3.81	0
6	−818.124881	1.97	1.09	2.02	0	0
7	−818.126291	1.09	4.85	1.19	4.86	0
8	−818.125617	1.51	2.37	1.83	1.63	0
12	−818.127129	0.56	11.78	0.30	21.65	0
13	−818.126468	0.98	5.84	0.77	9.83	0
16	−818.126421	1.00	5.56	1.15	5.18	0

[a] conformers are numbered according to their appearance during conformational search.

Table S14. Total (E, in Hartree) and relative energies (ΔE, $\Delta\Delta G$, in kcal mol^{-1}), percentage populations (Pop.) and number of imaginary frequencies calculated at the IEFPCM($CHCl_3$)/B3LYP/6-311++G(d,p) level for low-energy conformers of **19**.

Conformer [a]	E	ΔE	Pop.	$\Delta\Delta G$	Pop.	#ImFreq
1	−818.123396	0.10	19.24	0.94	9.41	0
2	−818.123552	0.00	22.71	0.00	46.19	0
3	−818.122139	0.89	5.08	1.19	6.23	0

4	−818.122782	0.48	10.04	1.65	2.86	0
5	−818.123502	0.03	21.53	1.13	6.81	0
6	−818.120586	1.86	0.98	2.35	0	0
7	−818.12162	1.21	2.93	1.37	4.57	0
8	−818.120865	1.69	1.32	2.30	0	0
12	−818.122776	0.49	9.97	0.71	13.98	0
13	−818.121781	1.11	3.47	1.06	7.74	0
16	−818.121556	1.25	2.74	1.80	2.21	0

[a] conformers are numbered according to their appearance during conformational search.

Table S15. Total (E, in Hartree) and relative energies (*ΔE*, *ΔΔG*, in kcal mol^{-1}), percentage populations (Pop.) and number of imaginary frequencies calculated at the IEFPCM(MeCN)/B3LYP/6-311++G(d,p) level for low-energy conformers of **20**.

Conformer [a]	E	*ΔE*	Pop.	*ΔΔG*	Pop.	#ImFreq
1	−857.4530357	1.48	2.45	1.68	2.17	0
4	−857.4548673	0.33	17.04	0.49	16.4	0
5	−857.4542218	0.74	8.6	1.00	6.93	0
17	−857.4529805	1.51	2.31	1.25	4.52	0
20	−857.4531357	1.42	2.72	1.53	2.79	0
24	−857.4532572	1.34	3.09	1.60	2.49	0
26	−857.4540028	0.87	6.82	1.32	4.01	0
33	−857.4553935	0.00	29.76	0.00	37.2	0
38	−857.454734	0.41	14.79	0.65	12.36	0
41	−857.4545692	0.52	12.42	0.71	11.13	0

[a] conformers are numbered according to their appearance during conformational search.

Table S16. Total (E, in Hartree) and relative energies (*ΔE*, *ΔΔG*, in kcal mol^{-1}), percentage populations (Pop.) and number of imaginary frequencies calculated at the IEFPCM($CHCl_3$)/B3LYP/6-311++G(d,p) level for low-energy conformers of **20**.

Conformer [a]	E	*ΔE*	Pop.	*ΔΔG*	Pop.	#ImFreq
1	−857.4495131	0.98	1.99	5.2	1.43	0
3	−857.4504411	0.40	0.34	13.91	23.01	0
5	−857.4502584	0.51	0.99	11.46	7.7	0
17	−857.4489162	1.36	1.23	2.76	5.14	0
20	−857.4484538	1.65	1.92	1.69	1.6	0
24	−857.4483962	1.68	1.86	1.59	1.77	0
26	−857.4502805	0.50	1.25	11.73	4.94	0
33	−857.4510777	0.00	0.00	27.3	40.82	0
38	−857.4502134	0.54	1.66	10.93	2.45	0
41	−857.4504082	0.42	0.77	13.43	11.13	0

[a] conformers are numbered according to their appearance during conformational search.

Table S17. Calculated at the IEFPCM($CHCl_3$)/B3LYP/*aug*-cc-pVTZ level optical rotations for alcohol **13**.

Conformer [a]	589 nm	578 nm	546 nm	436 nm
1	−473	−496	−573	−1065
3	455	477	551	1027
4	−430	−462	−535	−1011
6	445	468	543	1039
14	−20	−20	−19	2

16	−1	−1	−1	2
18	66	70	83	181
20	137	144	167	325
26	206	216	245	411
29	−140	−146	−169	−312
32	−4	−3	−1	23
33	111	116	130	209
35	132	138	159	288
39	95	100	116	217
40	−57	−59	−68	−116
43	−136	−142	−162	−274
44	−154	−161	−186	−342
46	−535	−562	−653	−1258
51	−78	−82	−95	−181
52	21	23	28	66
53	−30	−31	−33	−24
54	419	440	507	943
60	−9	−9	−8	5

[a] conformers are numbered according to their appearance during conformational search.

Table 18. Calculated at the IEFPCM($CHCl_3$)/B3LYP/*aug*-cc-pVTZ level optical rotations for alcohol **14.**

Conformer [a]	589 nm	578 nm	546 nm	436 nm
1	367	386	446	839
2	595	624	720	1323
3	355	373	433	840
4	594	624	721	1346
5	−33	−35	−41	−81
6	−308	−322	−371	−673
7	−496	−521	−606	−1164
8	−680	−714	−826	−1553
9	100	104	117	183
10	−135	−141	−159	−255
11	148	156	182	355
12	121	126	145	256
13	−151	−158	−181	−326
14	50	52	60	109
15	−153	−161	−187	−356
16	−240	−251	−288	−513
17	−129	−135	−155	−276
18	98	102	117	198
19	−149	−156	−177	−299
20	−464	−487	−563	−1057
21	−677	−710	−818	−1507
22	7	8	8	9
23	−258	−271	−311	−560
24	−433	−455	−526	−985
25	−152	−158	−179	−292
26	101	106	123	229
27	133	140	163	321
28	−48	−50	−55	−68
29	218	229	266	504

30	142	149	172	323
31	−394	−414	−481	−922
32	−140	−147	−167	−283
33	−638	−669	−772	−1416
34	−623	−653	−755	−1400
35	−69	−72	−81	−122
36	187	196	227	431
37	−380	−399	−460	−850
38	−576	−604	−695	−1262
39	367	385	445	835
40	599	628	724	1325
41	371	390	453	869
42	600	629	726	1349

[a] conformers are numbered according to their appearance during conformational search.

Table S19. Calculated at the IEFPCM($CHCl_3$)/B3LYP/*aug*-cc-pVTZ level optical rotations for alcohol **15.**

Conformer [a]	589 nm	578 nm	546 nm	436 nm
2	400	420	486	912
4	−393	−417	−479	−915
11	383	403	468	898
18	54	57	68	149
21	−13	−13	−12	10
25	−131	−137	−157	−282
26	191	200	228	406
27	−66	−69	−79	−139
28	201	211	243	453
34	−388	−408	−474	−910
39	−396	−415	−480	−904
44	−93	−97	−112	−201
45	−32	−34	−38	−64
46	−165	−172	−196	−337
49	−176	−185	−213	−389

[a] conformers are numbered according to their appearance during conformational search.

Table S20. Calculated at the IEFPCM($CHCl_3$)/B3LYP/*aug*-cc-pVTZ level optical rotations for alcohol **16.**

Conformer [a]	589 nm	578 nm	546 nm	436 nm
2	372	390	451	851
9	350	368	428	826
15	−22	−23	−23	−14
16	52	55	66	143
22	−116	−121	−139	−246
23	220	231	264	471
24	238	248	288	535
25	−48	−50	−57	−97
35	−350	−368	−428	−826
38	−360	−378	−438	−827
39	−199	−208	−238	−417
40	−79	−83	−95	−168
43	−204	−214	−247	−454

46	329	345	398	738
48	−186	−195	−226	−421

[a] conformers are numbered according to their appearance during conformational search.

Table S21. Calculated at the IEFPCM($CHCl_3$)/B3LYP/*aug*-cc-pVTZ level optical rotations for alcohol **17.**

Conformer[a]	589 nm	578 nm	546 nm	436 nm
1	436	458	529	985
2	−403	−423	−489	−911
3	55	58	68	137
4	−187	−196	−226	−422
5	−55	−57	−63	−90

[a] conformers are numbered according to their appearance during conformational search.

Table S22. Calculated at the IEFPCM($CHCl_3$)/B3LYP/*aug*-cc-pVTZ level optical rotations for alcohol **18**.

Conformer [a]	589 nm	578 nm	546 nm	436 nm
2	354	372	470	811
3	594	623	718	1322
4	−596	−625	−721	−1323
5	306	322	372	700
6	134	140	162	301
7	−163	−170	−194	−334
8	−54	−57	−68	−140
9	−185	−193	−220	−371
10	97	101	113	177
11	103	109	127	249
12	−330	−346	−398	−733
13	−145	−153	−178	−338
16	−472	−496	−575	−1096
17	−692	−726	−839	−1561
20	371	390	453	872
21	598	627	725	1357
29	587	616	710	1301
32	−155	−161	−183	−304
33	124	130	151	289

[a] conformers are numbered according to their appearance during conformational search.

Table S23. Calculated at the IEFPCM($CHCl_3$)/B3LYP/*aug*-cc-pVTZ level optical rotations for alcohol **19**.

Conformer [a]	589 nm	578 nm	546 nm	436 nm
1	370	388	449	839
2	−41	−43	−47	−66
3	−168	−176	−203	−376
4	−330	−346	−401	−753
5	11	12	14	34
6	173	181	207	367
7	388	408	474	911
8	−211	−222	−256	−474
12	−101	−106	−121	−208

13	353	371	428	793
16	−2	−1	0.5	20

[a] conformers are numbered according to their appearance during conformational search.

Table S24. Calculated at the IEFPCM($CHCl_3$)/B3LYP/*aug*-cc-pVTZ level optical rotations for alcohol **20**.

**Conformer** [a]	**589 nm**	**578 nm**	**546 nm**	**436 nm**
1	−275	−289	334	−632
3	−43	−45	−50	−73
5	343	361	417	783
17	−152	−159	−184	−339
20	363	382	443	858
24	−2	−2	−1	18
26	35	37	43	88
33	−77	−81	−92	−162
38	−145	−152	−175	−325
41	288	302	348	643

[a] conformers are numbered according to their appearance during conformational search.

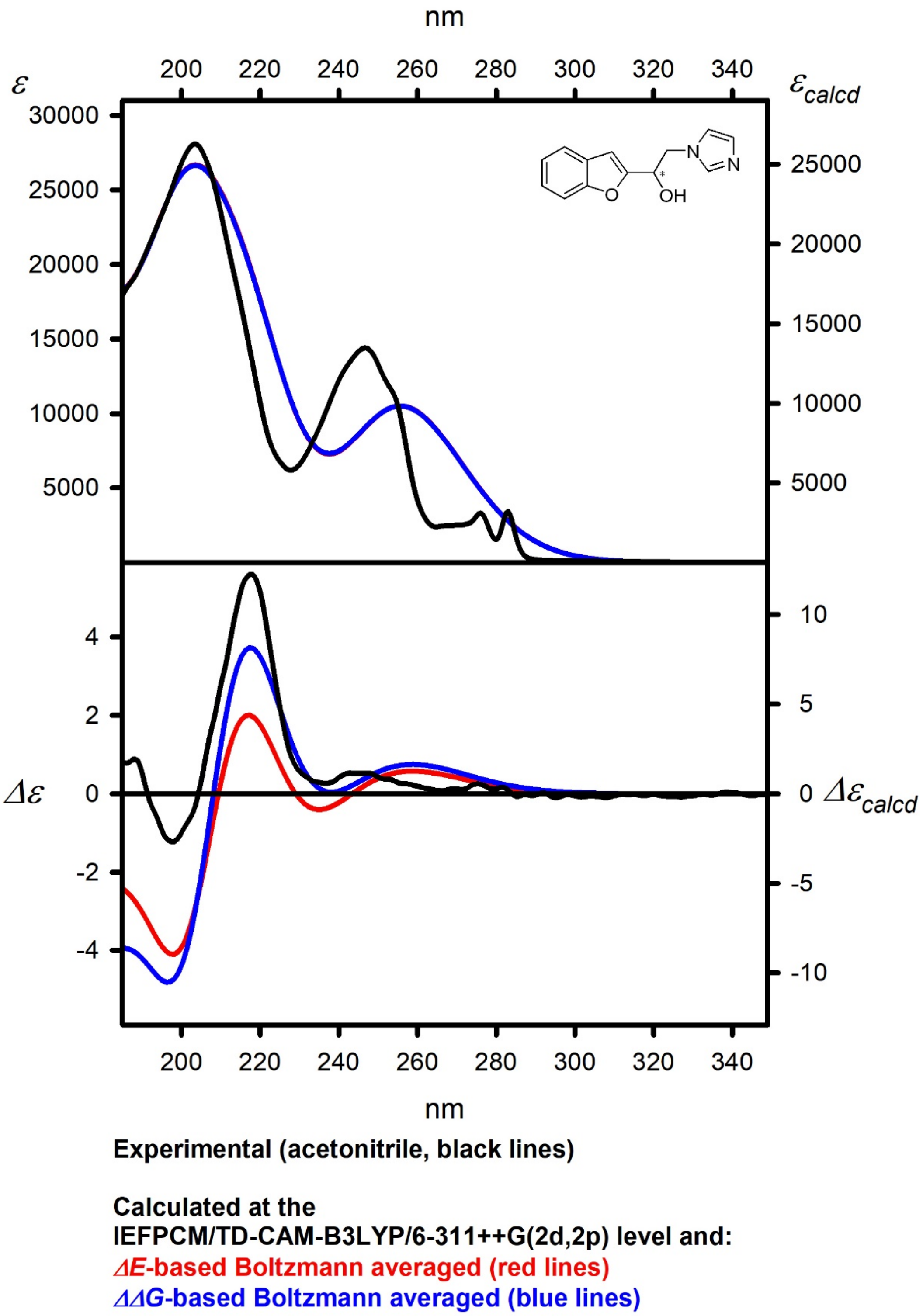

Figure 25. UV (upper panel) and ECD (lower panel) spectra of alcohol **13**, measured in acetonitrile (black lines), and calculated at the IEFPCM/TD-CAM-B3LYP/6-311++G(2p,2d) level and *ΔE*- (red lines) and *ΔΔG*-based Boltzmann averaged (blue lines). Wavelengths were corrected to match experimental UV maximum.

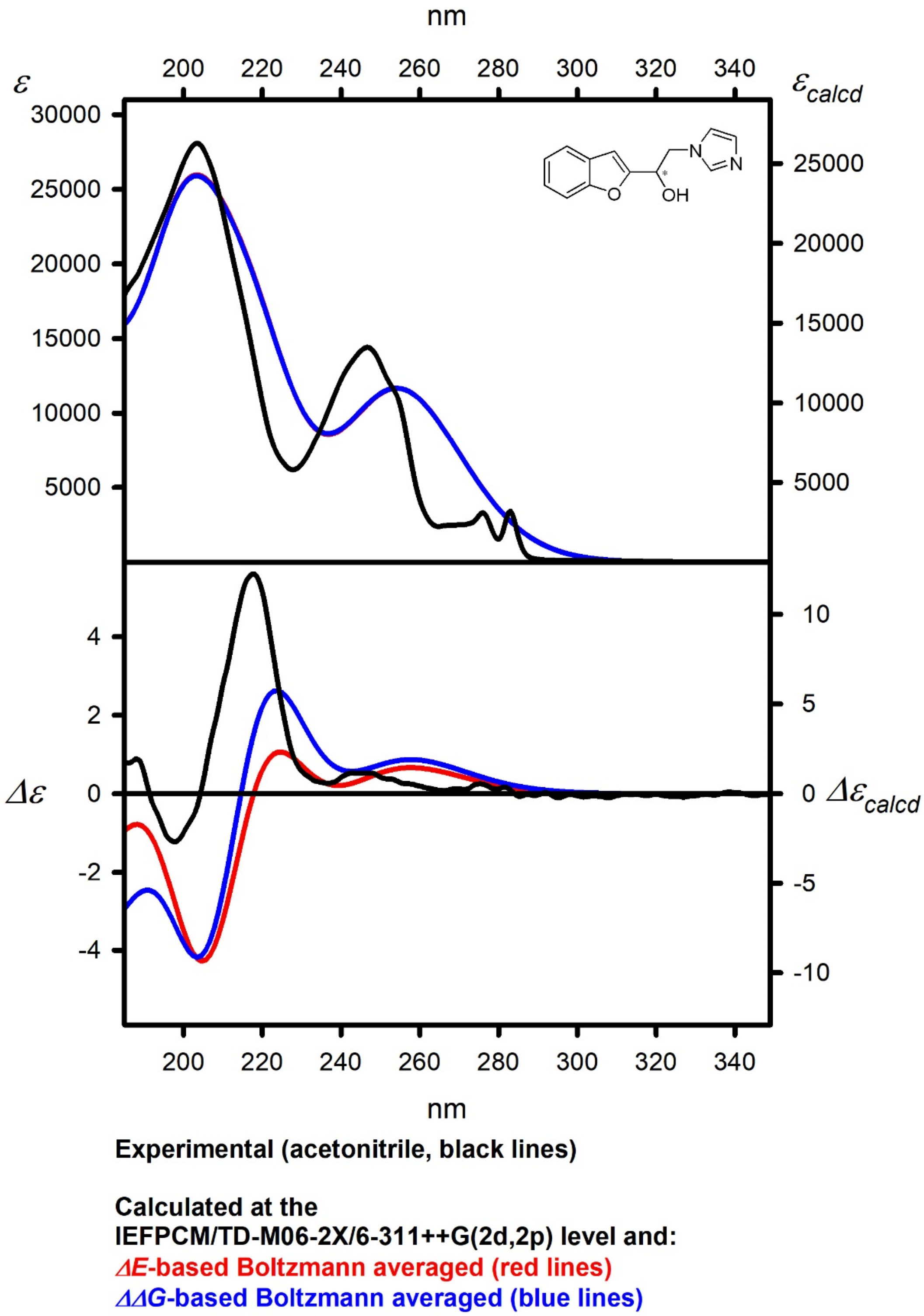

Figure S26. UV (upper panel) and ECD (lower panel) spectra of alcohol **13**, measured in acetonitrile (black lines), and calculated at the IEFPCM/TD-M06-2X/6-311++G(2p,2d) level and *ΔE*- (red lines) and *ΔΔG*-based Boltzmann averaged (blue lines). Wavelengths were corrected to match experimental UV maximum.

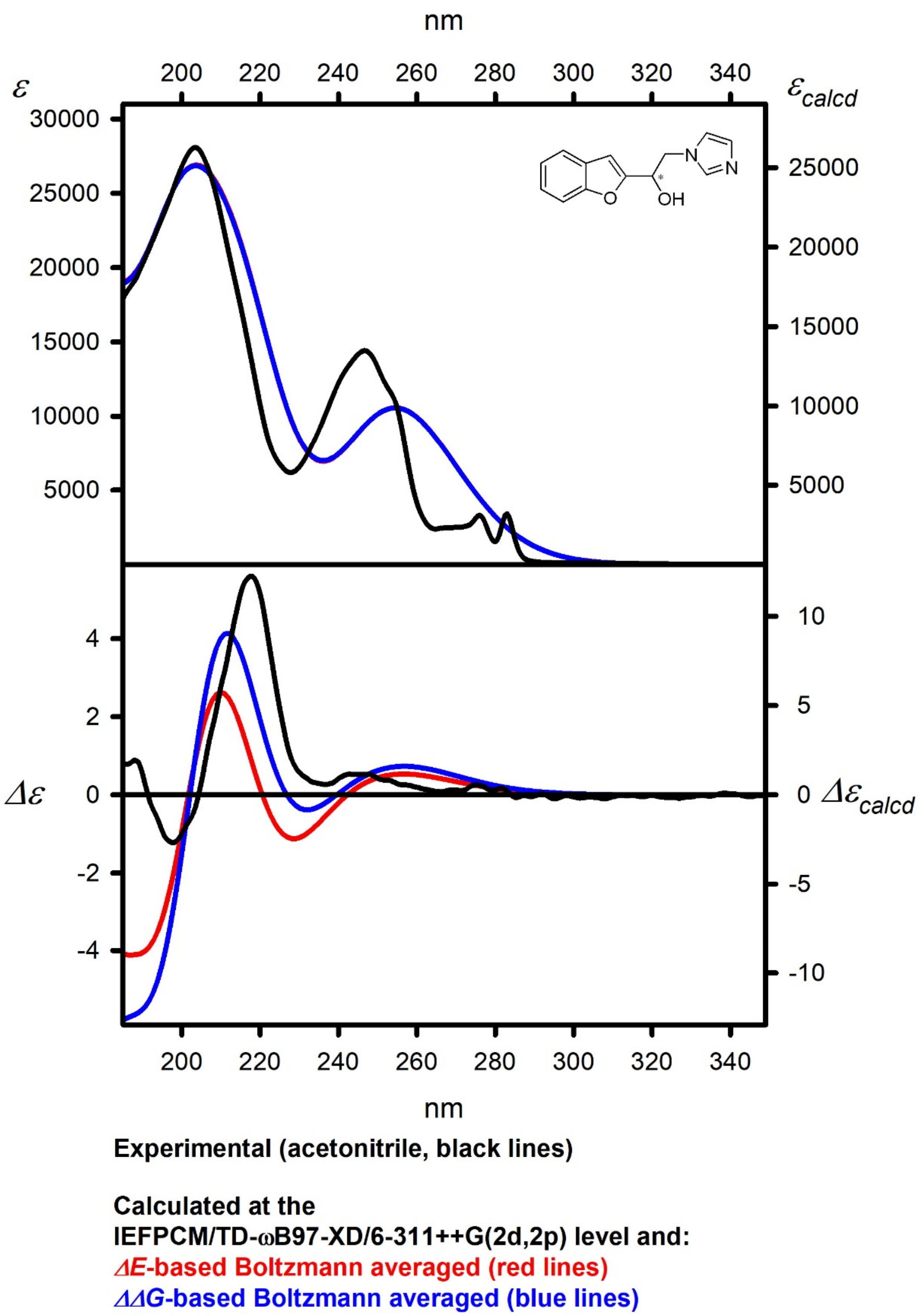

Figure S27. UV (upper panel) and ECD (lower panel) spectra of alcohol **13**, measured in acetonitrile (black lines), and calculated at the IEFPCM/TD-*ω*B97-XD/6-311++G(2p,2d) level and *ΔE*- (red lines) and *ΔΔG*-based Boltzmann averaged (blue lines). Wavelengths were corrected to match experimental UV maximum.

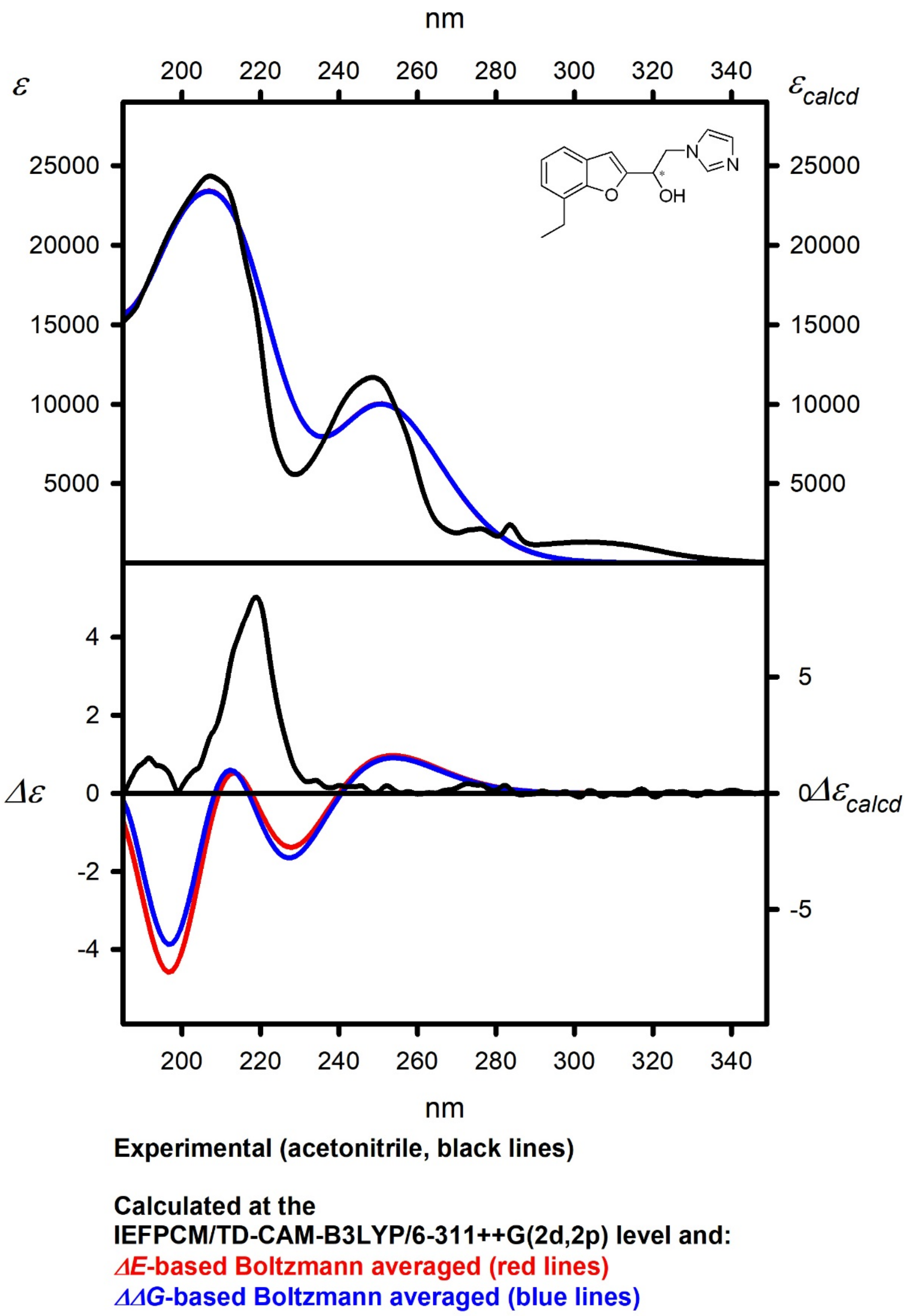

Figure S28. UV (upper panel) and ECD (lower panel) spectra of alcohol **14**, measured in acetonitrile (black lines), and calculated at the IEFPCM/TD-CAM-B3LYP/6-311++G(2p,2d) level and *ΔE*- (red lines) and *ΔΔG*-based Boltzmann averaged (blue lines). Wavelengths were corrected to match experimental UV maximum.

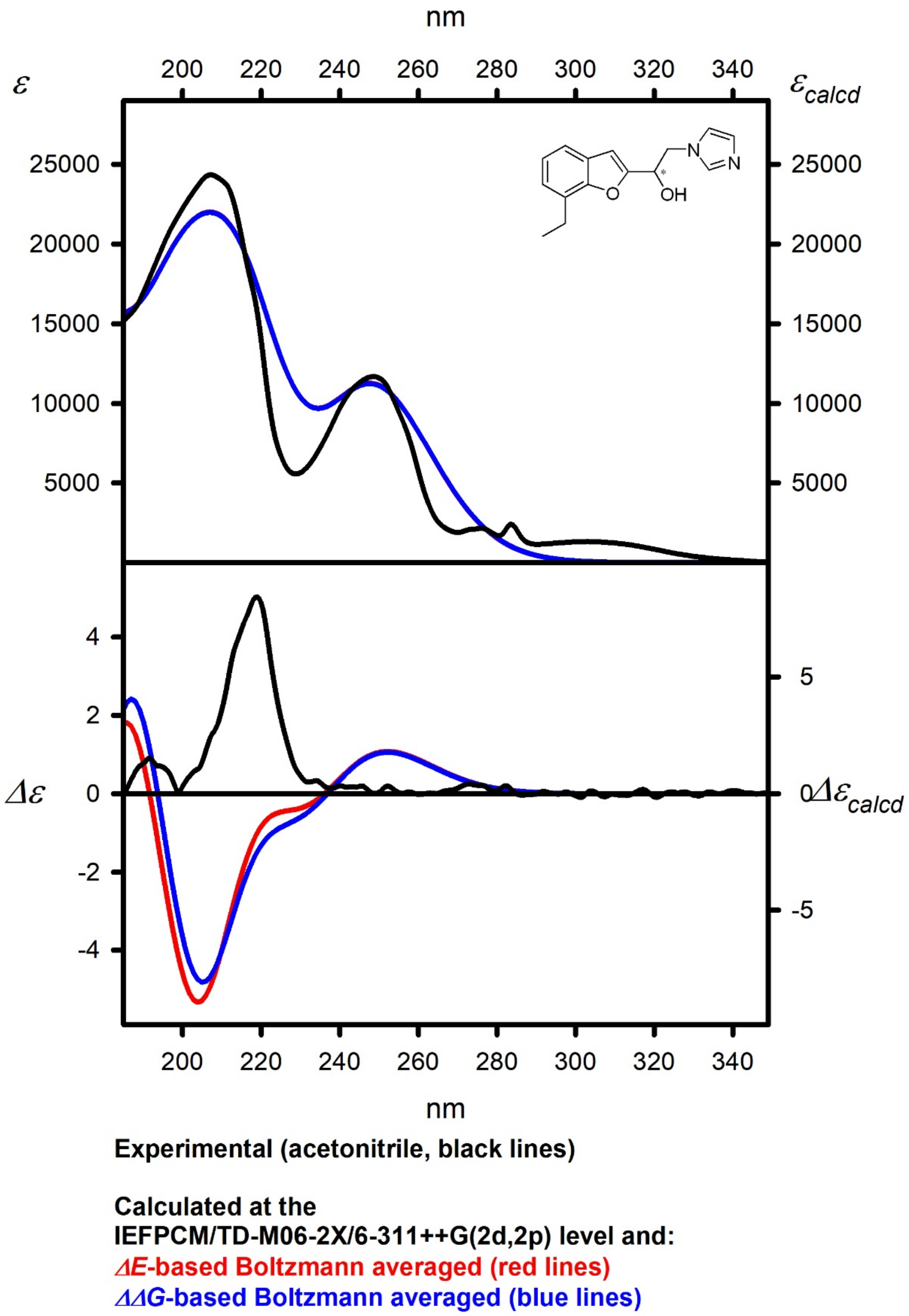

Figure S29. UV (upper panel) and ECD (lower panel) spectra of alcohol **14**, measured in acetonitrile (black lines), and calculated at the IEFPCM/TD-M06-2X/6-311++G(2p,2d) level and *ΔE*- (red lines) and *ΔΔG*-based Boltzmann averaged (blue lines). Wavelengths were corrected to match experimental UV maximum.

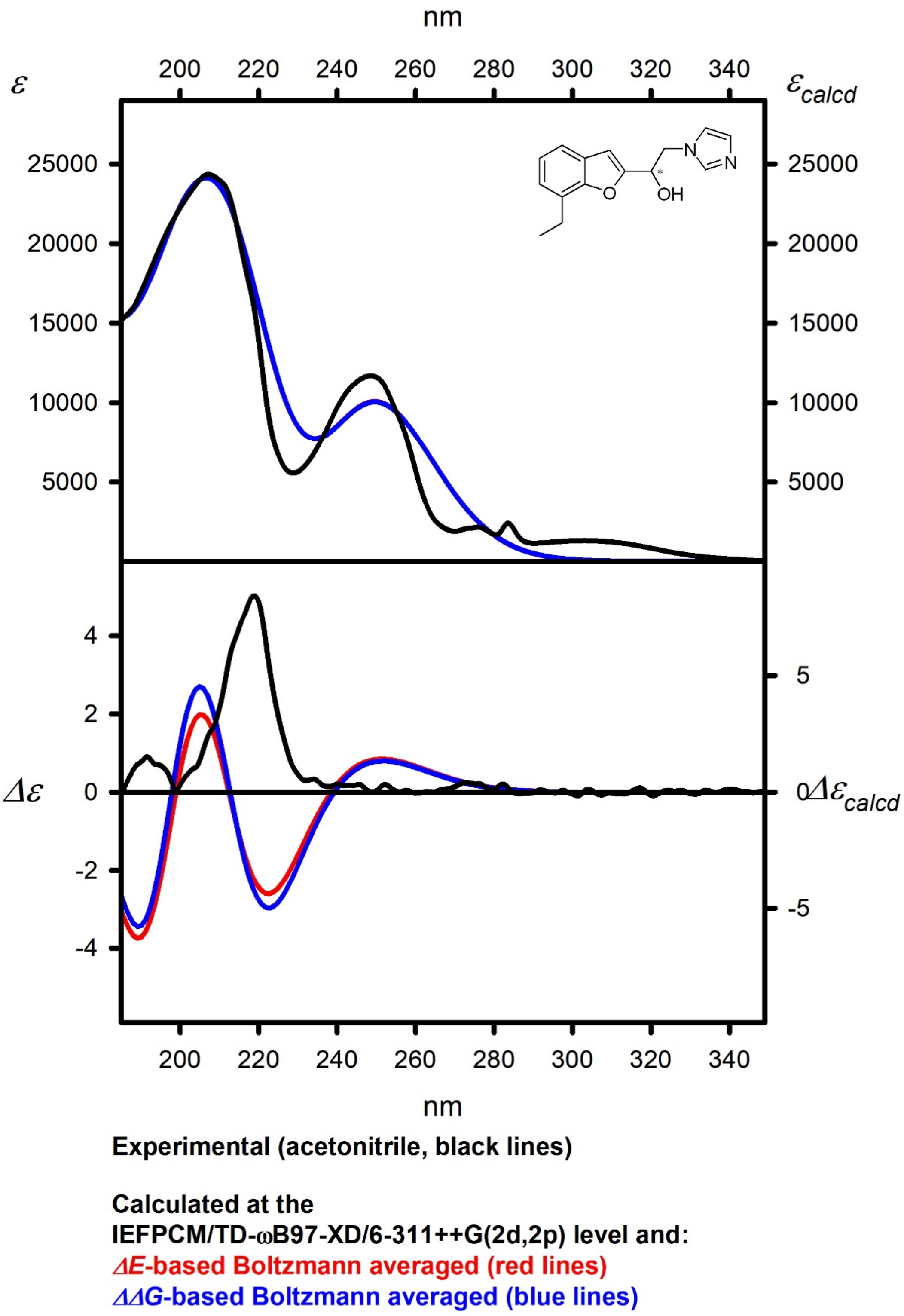

Figure S30. UV (upper panel) and ECD (lower panel) spectra of alcohol **14**, measured in acetonitrile (black lines), and calculated at the IEFPCM/TD-*ω*B97-XD/6-311++G(2p,2d) level and *ΔE*- (red lines) and *ΔΔG*-based Boltzmann averaged (blue lines). Wavelengths were corrected to match experimental UV maximum.

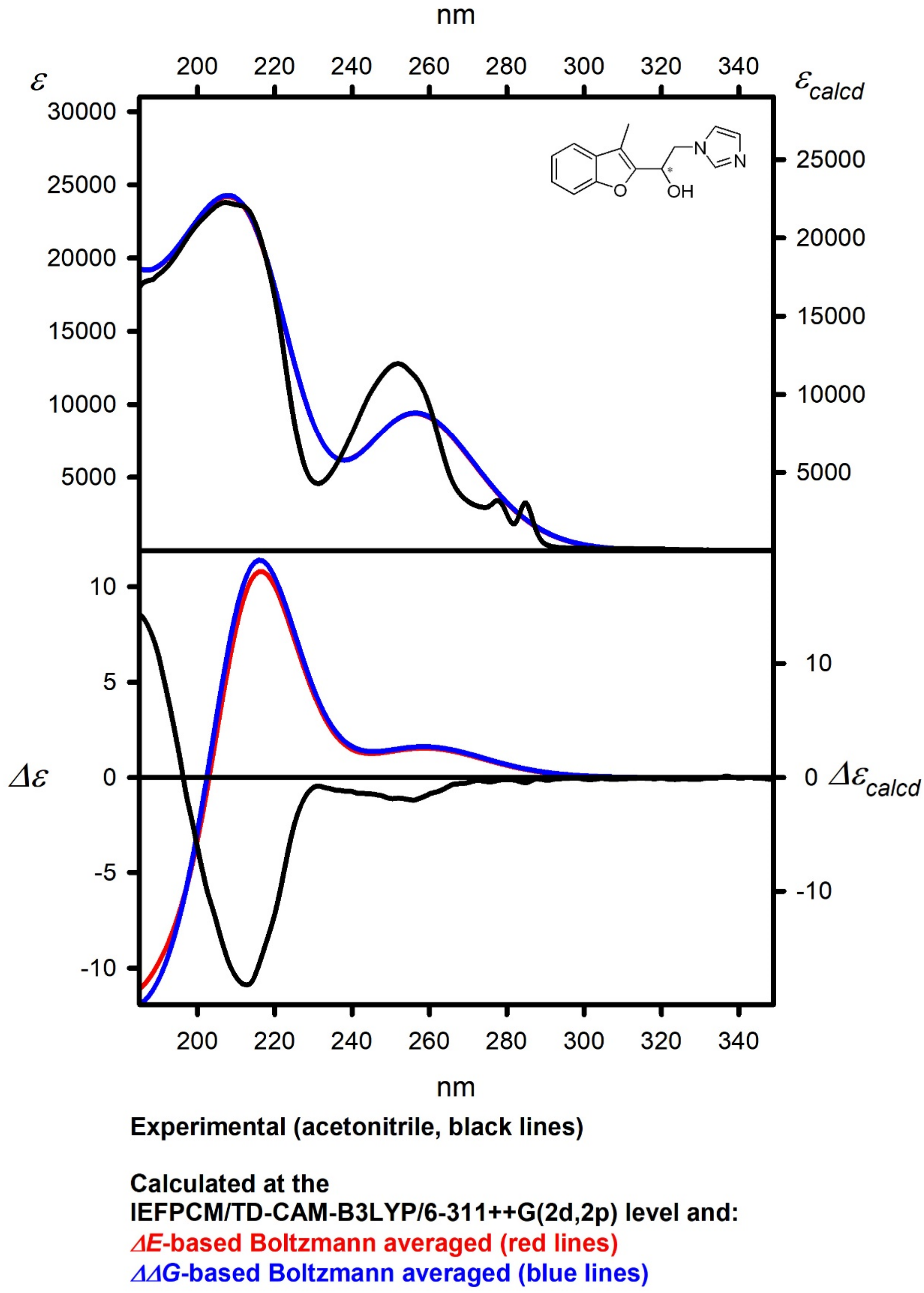

Figure S31. UV (upper panel) and ECD (lower panel) spectra of alcohol **15**, measured in acetonitrile (black lines), and calculated at the IEFPCM/TD-CAM-B3LYP/6-311++G(2p,2d) level and *ΔE*- (red lines) and *ΔΔG*-based Boltzmann averaged (blue lines). Wavelengths were corrected to match experimental UV maximum.

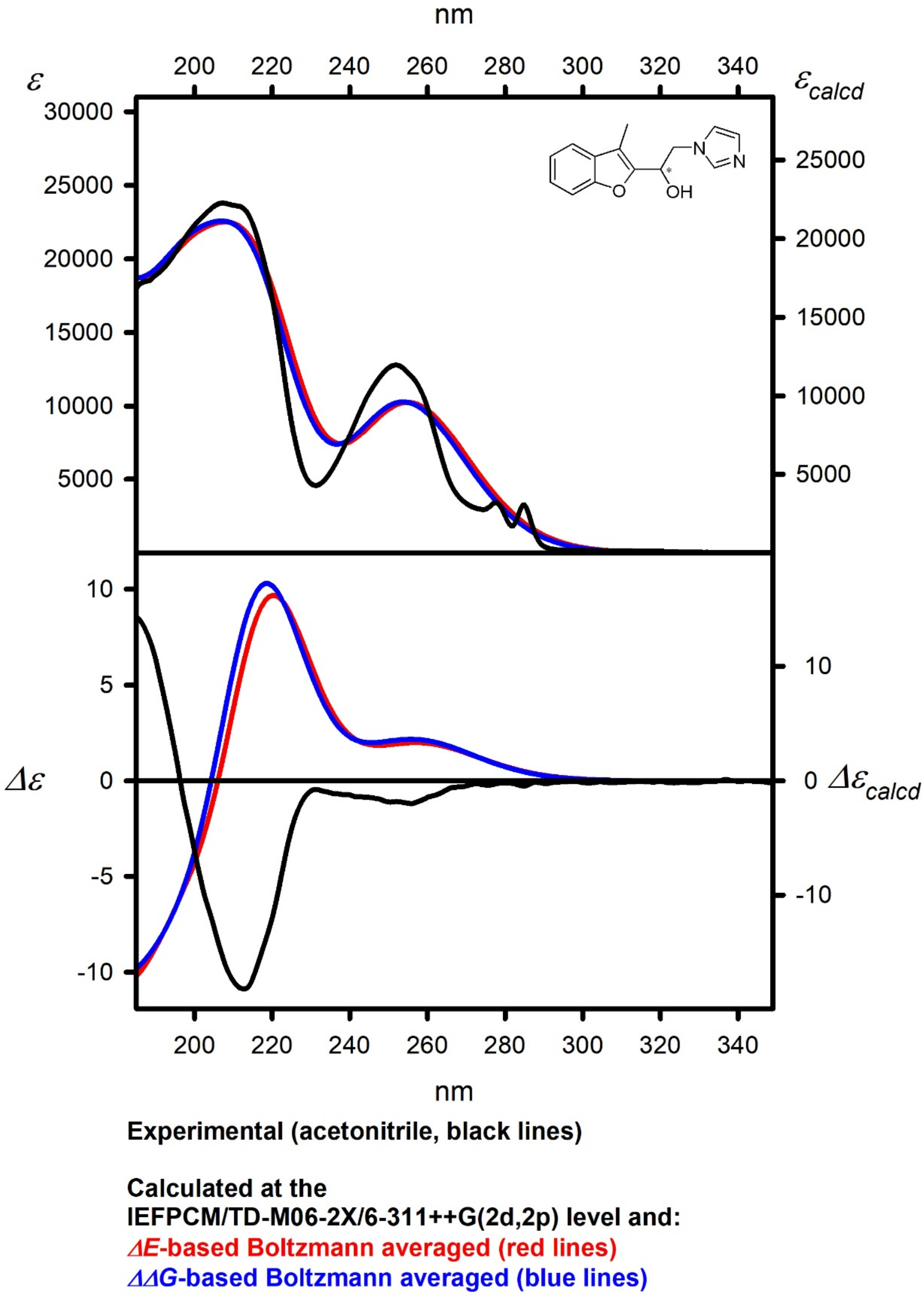

Figure S32. UV (upper panel) and ECD (lower panel) spectra of alcohol **15**, measured in acetonitrile (black lines), and calculated at the IEFPCM/TD-M06-2X/6-311++G(2p,2d) level and *ΔE*- (red lines) and *ΔΔG*-based Boltzmann averaged (blue lines). Wavelengths were corrected to match experimental UV maximum.

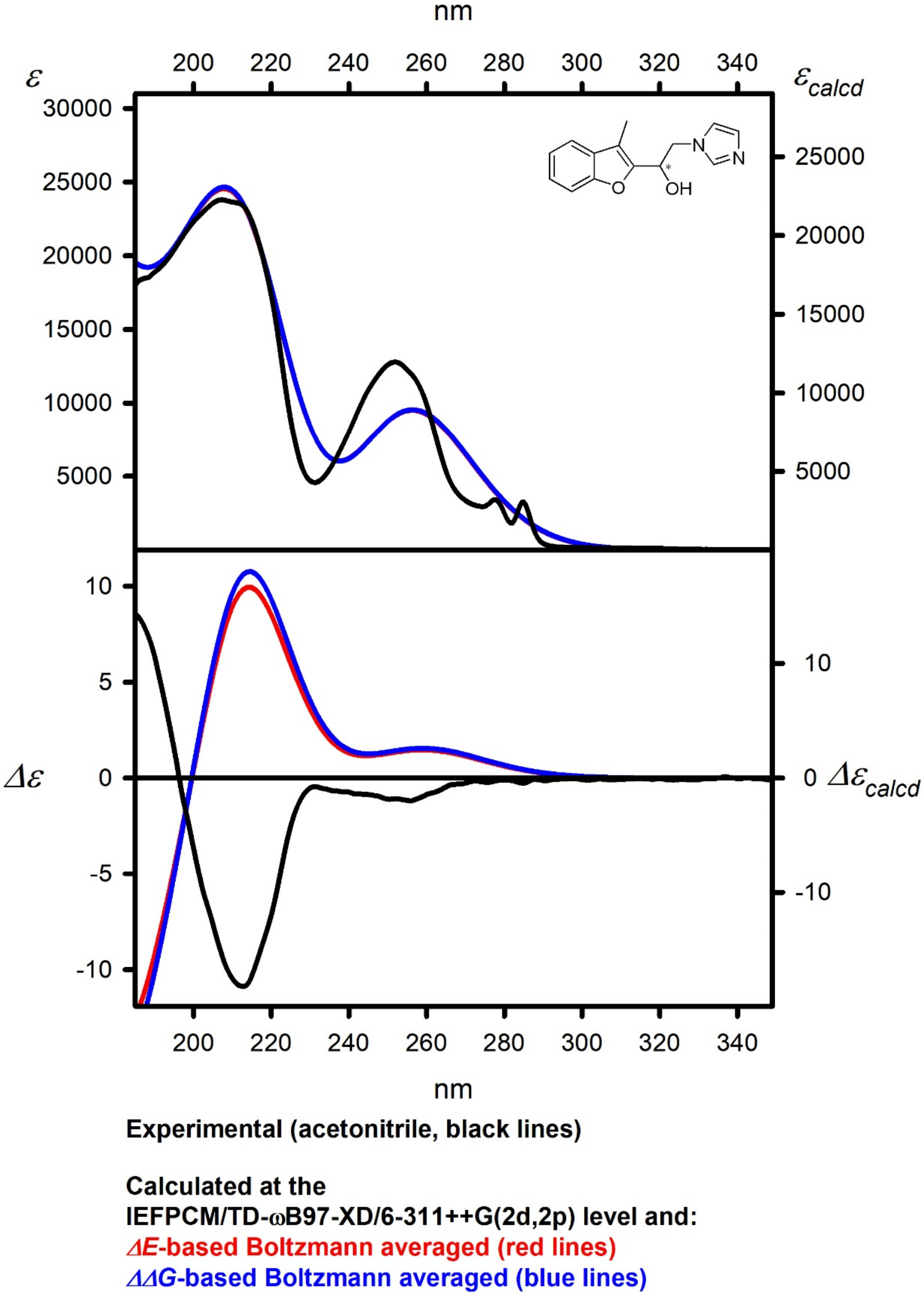

Figure S33. UV (upper panel) and ECD (lower panel) spectra of alcohol **15**, measured in acetonitrile (black lines), and calculated at the IEFPCM/TD-*ω*B97-XD/6-311++G(2p,2d) level and *ΔE*- (red lines) and *ΔΔG*-based Boltzmann averaged (blue lines). Wavelengths were corrected to match experimental UV maximum.

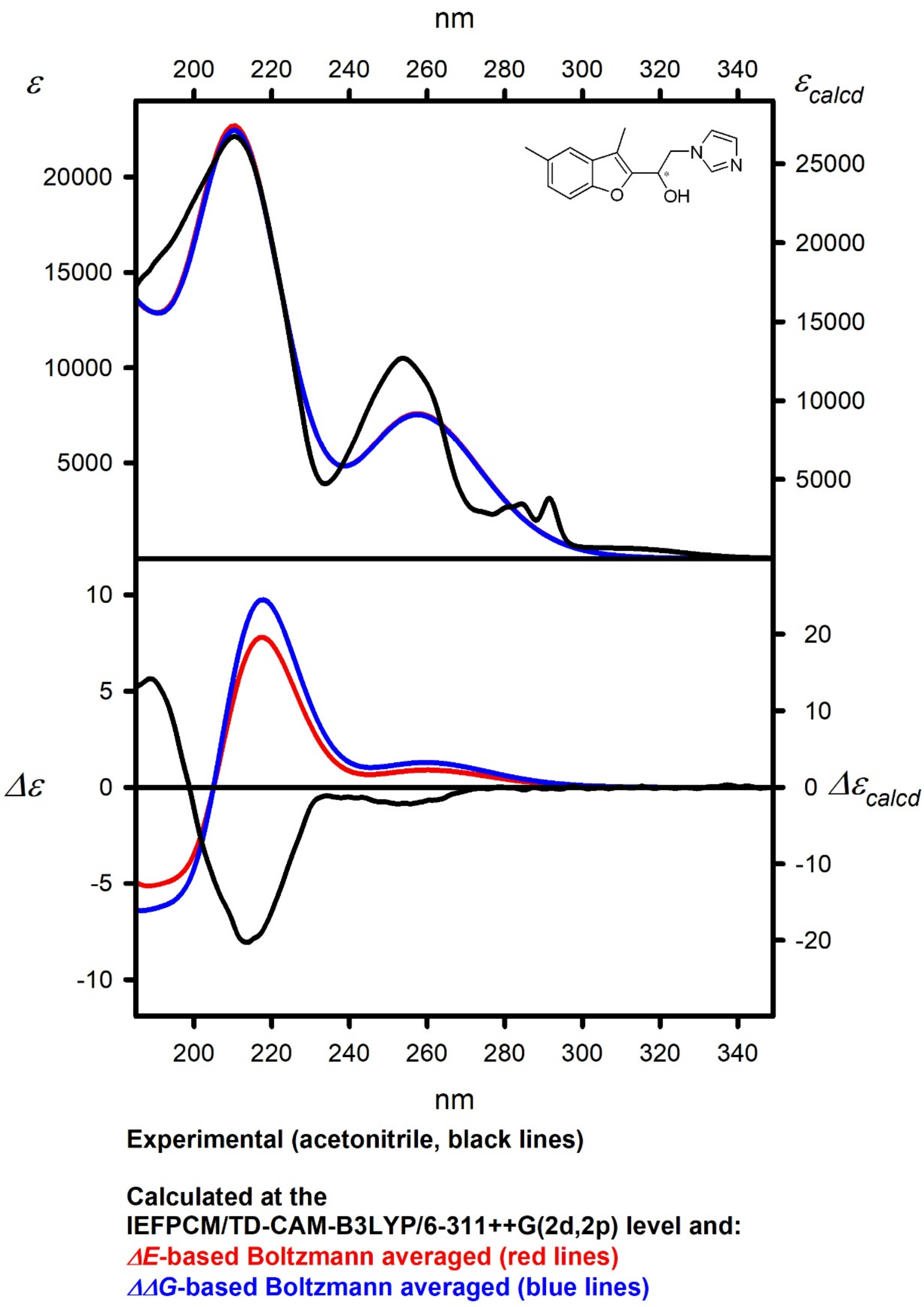

Figure S34. UV (upper panel) and ECD (lower panel) spectra of alcohol **16**, measured in acetonitrile (black lines), and calculated at the IEFPCM/TD-CAM-B3LYP/6-311++G(2p,2d) level and *ΔE*- (red lines) and *ΔΔG*-based Boltzmann averaged (blue lines). Wavelengths were corrected to match experimental UV maximum.

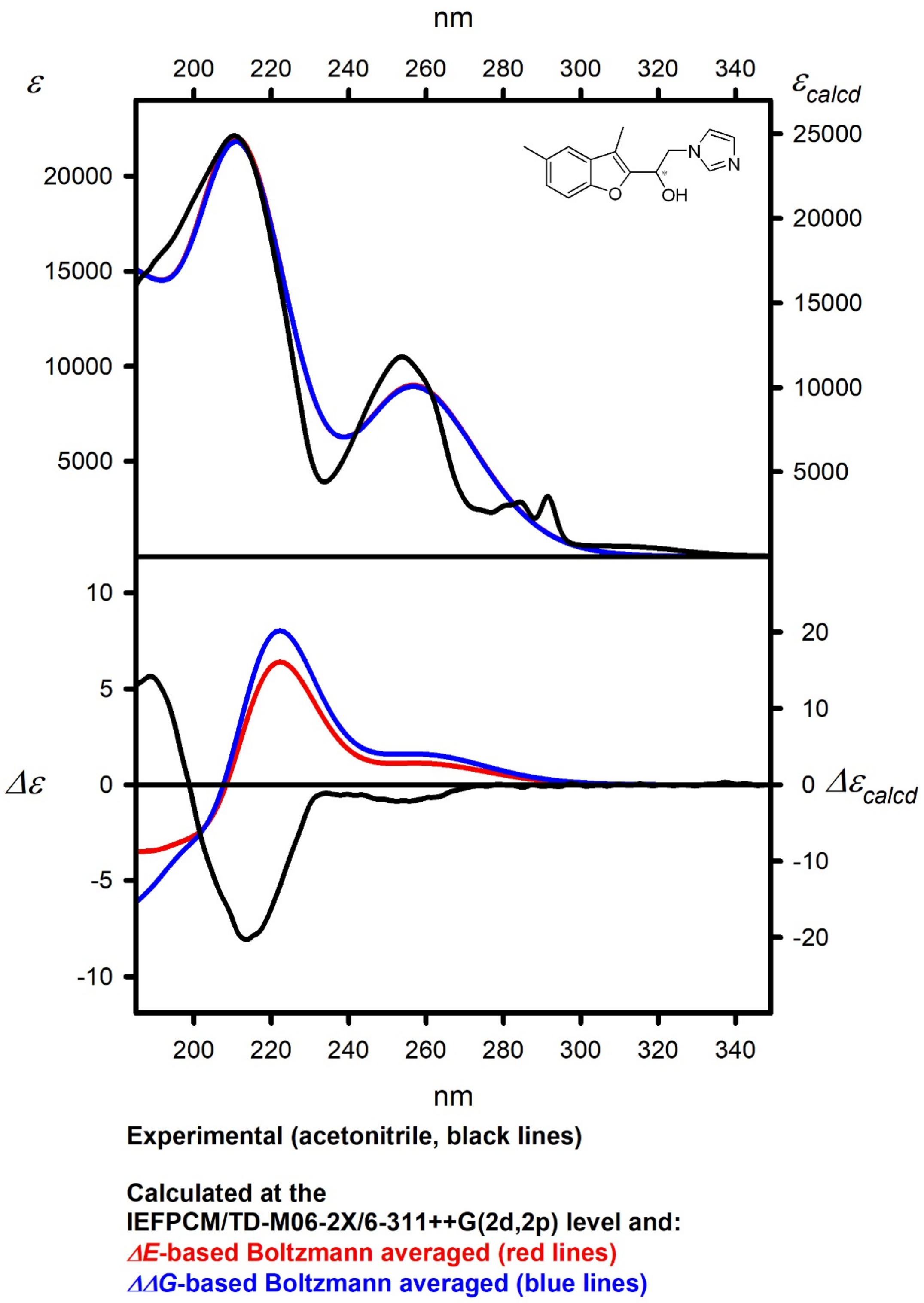

Figure S35. UV (upper panel) and ECD (lower panel) spectra of alcohol **16**, measured in acetonitrile (black lines), and calculated at the IEFPCM/TD-M06-2X/6-311++G(2p,2d) level and *ΔE*- (red lines) and *ΔΔG*-based Boltzmann averaged (blue lines). Wavelengths were corrected to match experimental UV maximum.

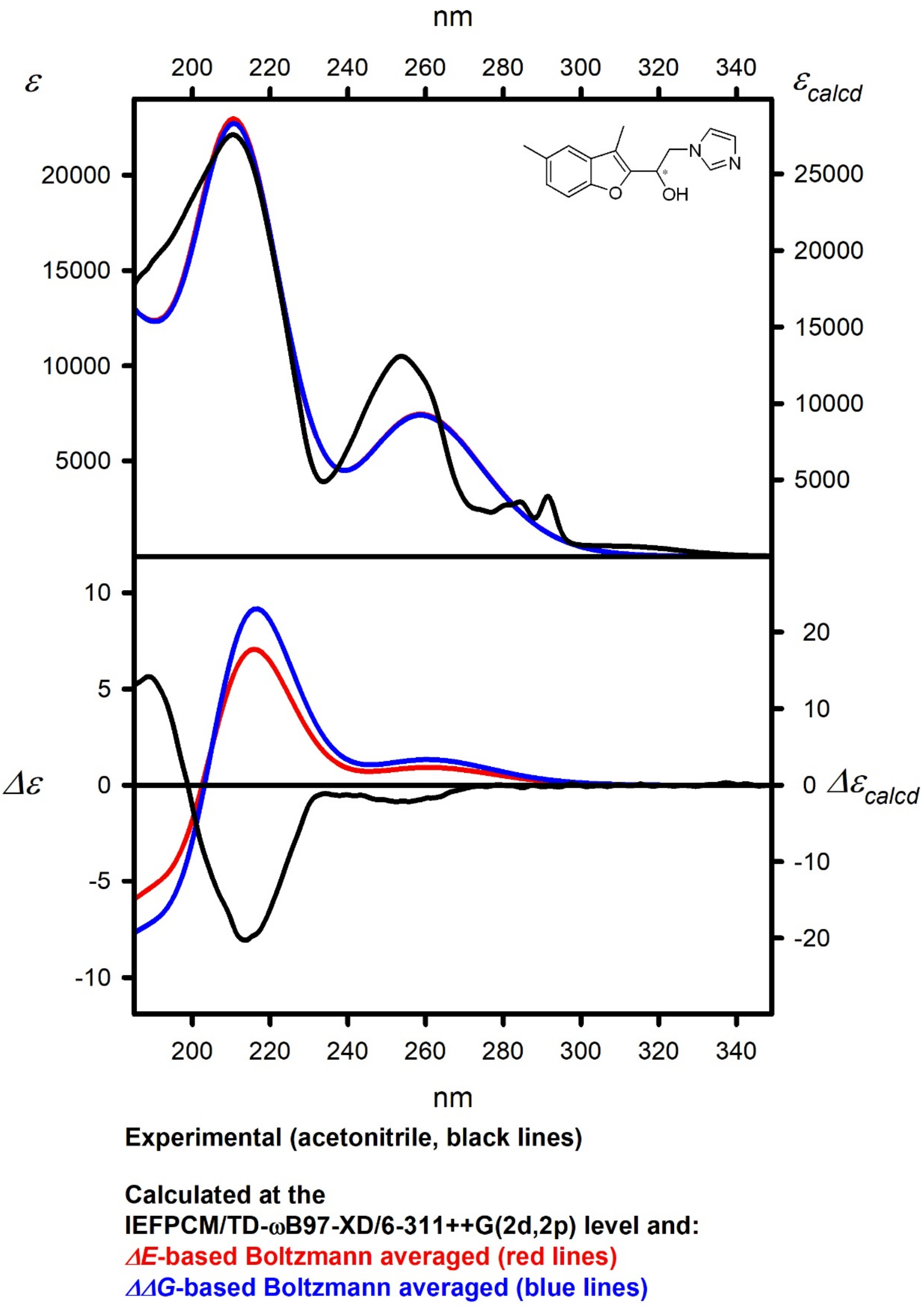

Figure S36. UV (upper panel) and ECD (lower panel) spectra of alcohol **16**, measured in acetonitrile (black lines), and calculated at the IEFPCM/TD-*ω*B97-XD/6-311++G(2p,2d) level and *ΔE*- (red lines) and *ΔΔG*-based Boltzmann averaged (blue lines). Wavelengths were corrected to match experimental UV maximum.

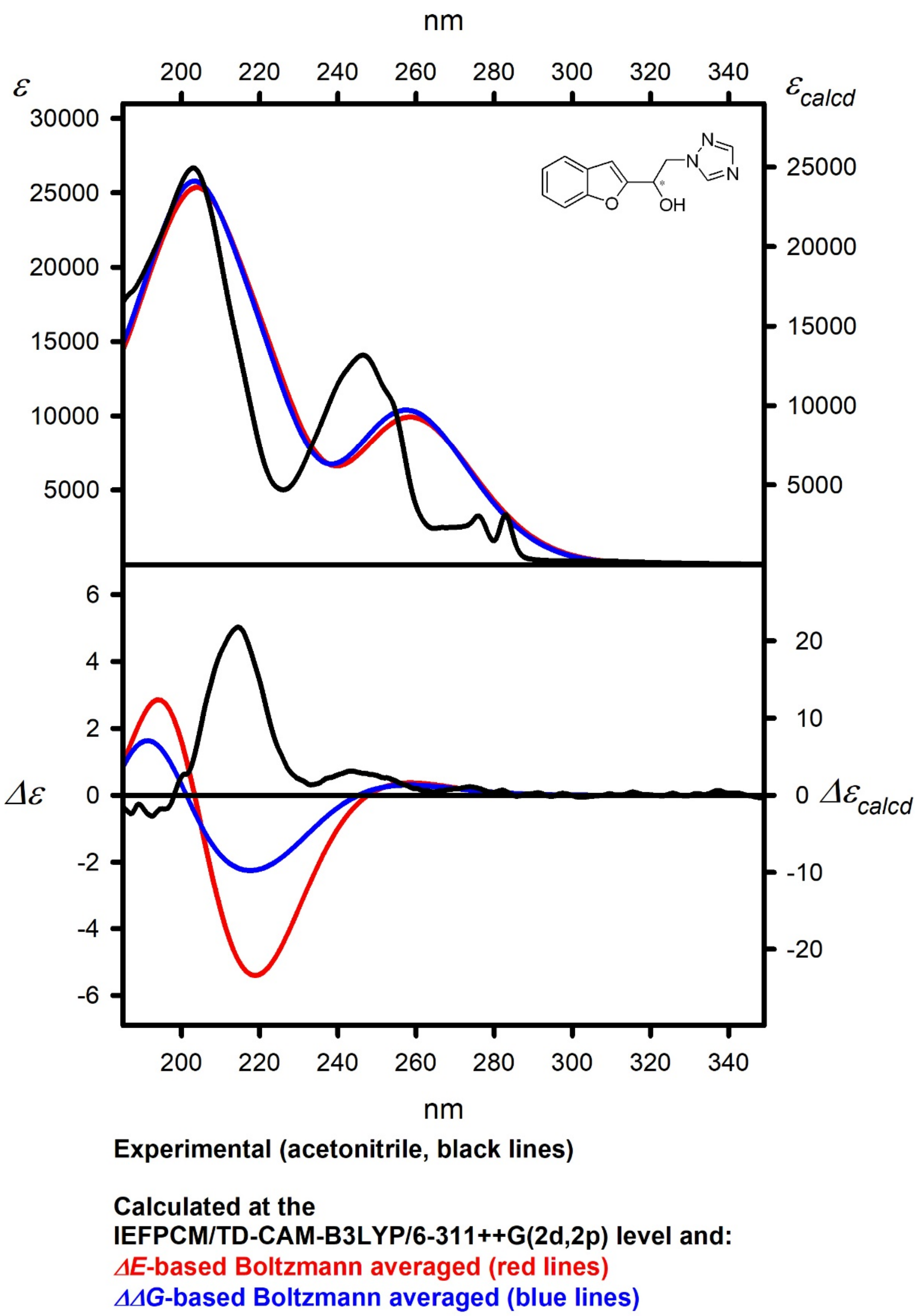

Figure S37. UV (upper panel) and ECD (lower panel) spectra of alcohol **17**, measured in acetonitrile (black lines), and calculated at the IEFPCM/TD-CAM-B3LYP/6-311++G(2p,2d) level and *ΔE*- (red lines) and *ΔΔG*-based Boltzmann averaged (blue lines). Wavelengths were corrected to match experimental UV maximum.

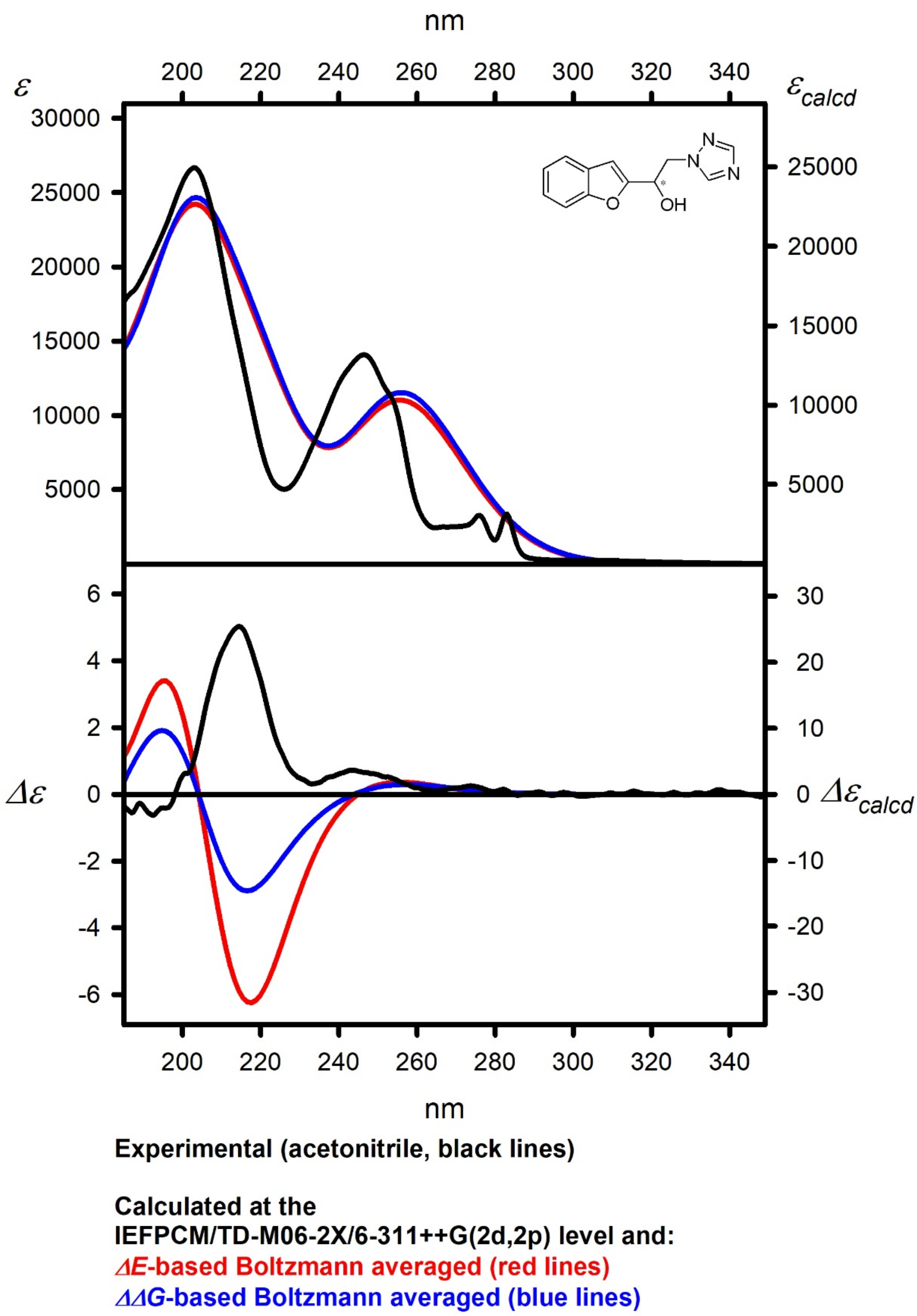

Figure S38. UV (upper panel) and ECD (lower panel) spectra of alcohol **17**, measured in acetonitrile (black lines), and calculated at the IEFPCM/TD-M06-2X/6-311++G(2p,2d) level and *ΔE*- (red lines) and *ΔΔG*-based Boltzmann averaged (blue lines). Wavelengths were corrected to match experimental UV maximum.

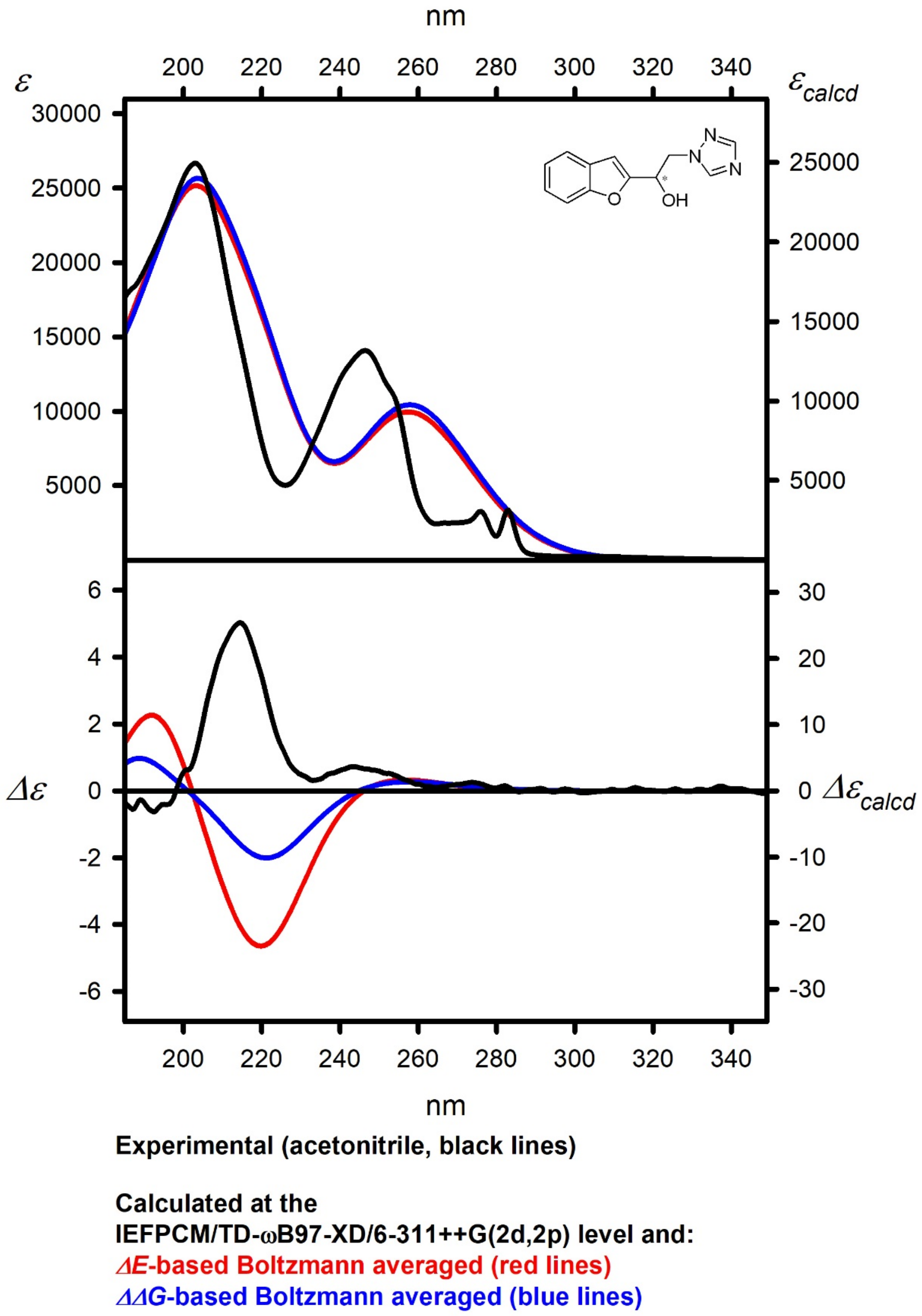

Figure S39. UV (upper panel) and ECD (lower panel) spectra of alcohol **17**, measured in acetonitrile (black lines), and calculated at the IEFPCM/TD-*ω*B97-XD/6-311++G(2p,2d) level and *ΔE*- (red lines) and *ΔΔG*-based Boltzmann averaged (blue lines). Wavelengths were corrected to match experimental UV maximum.

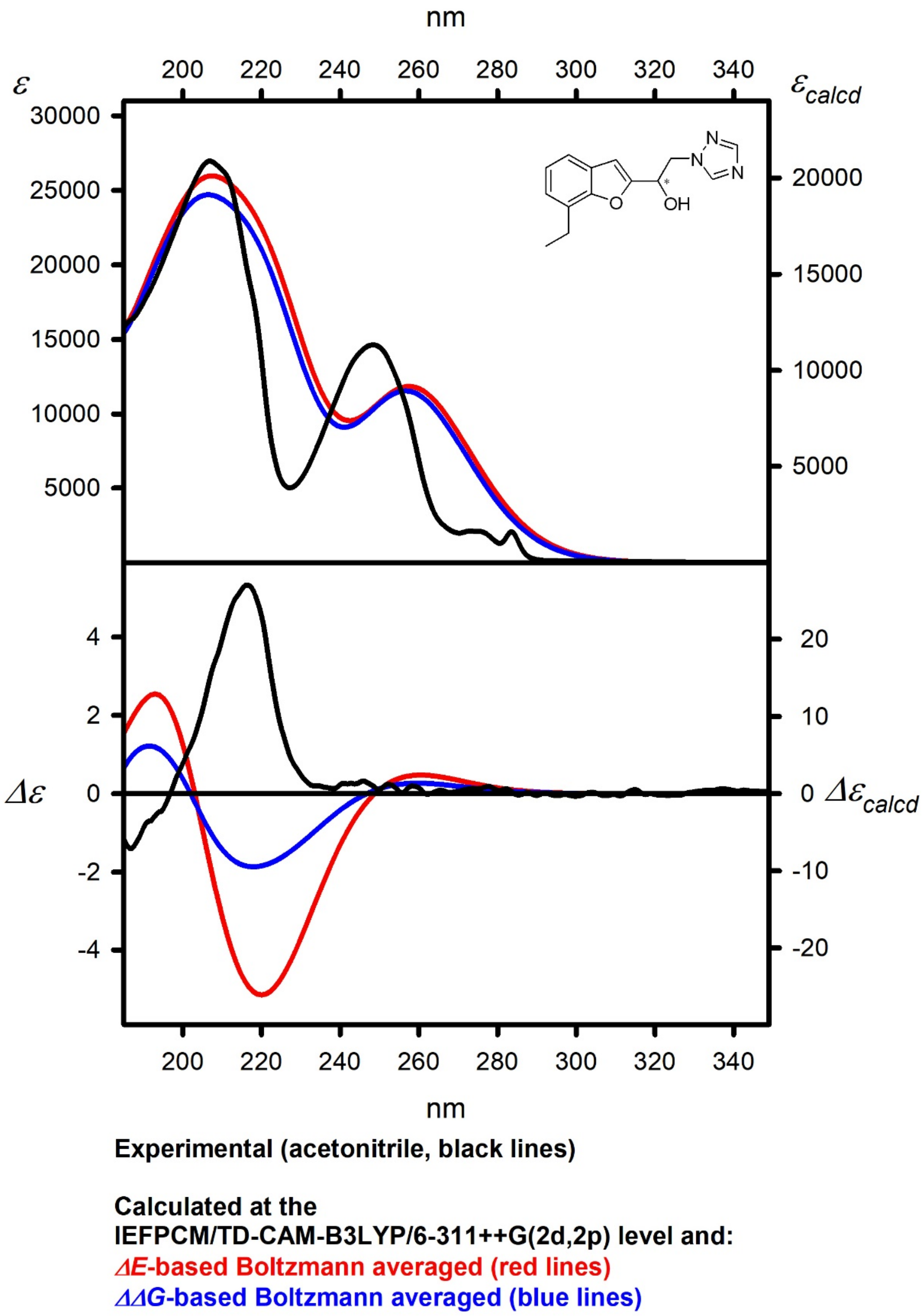

Figure S40. UV (upper panel) and ECD (lower panel) spectra of alcohol **18**, measured in acetonitrile (black lines), and calculated at the IEFPCM/TD-CAM-B3LYP/6-311++G(2p,2d) level and *ΔE*- (red lines) and *ΔΔG*-based Boltzmann averaged (blue lines). Wavelengths were corrected to match experimental UV maximum.

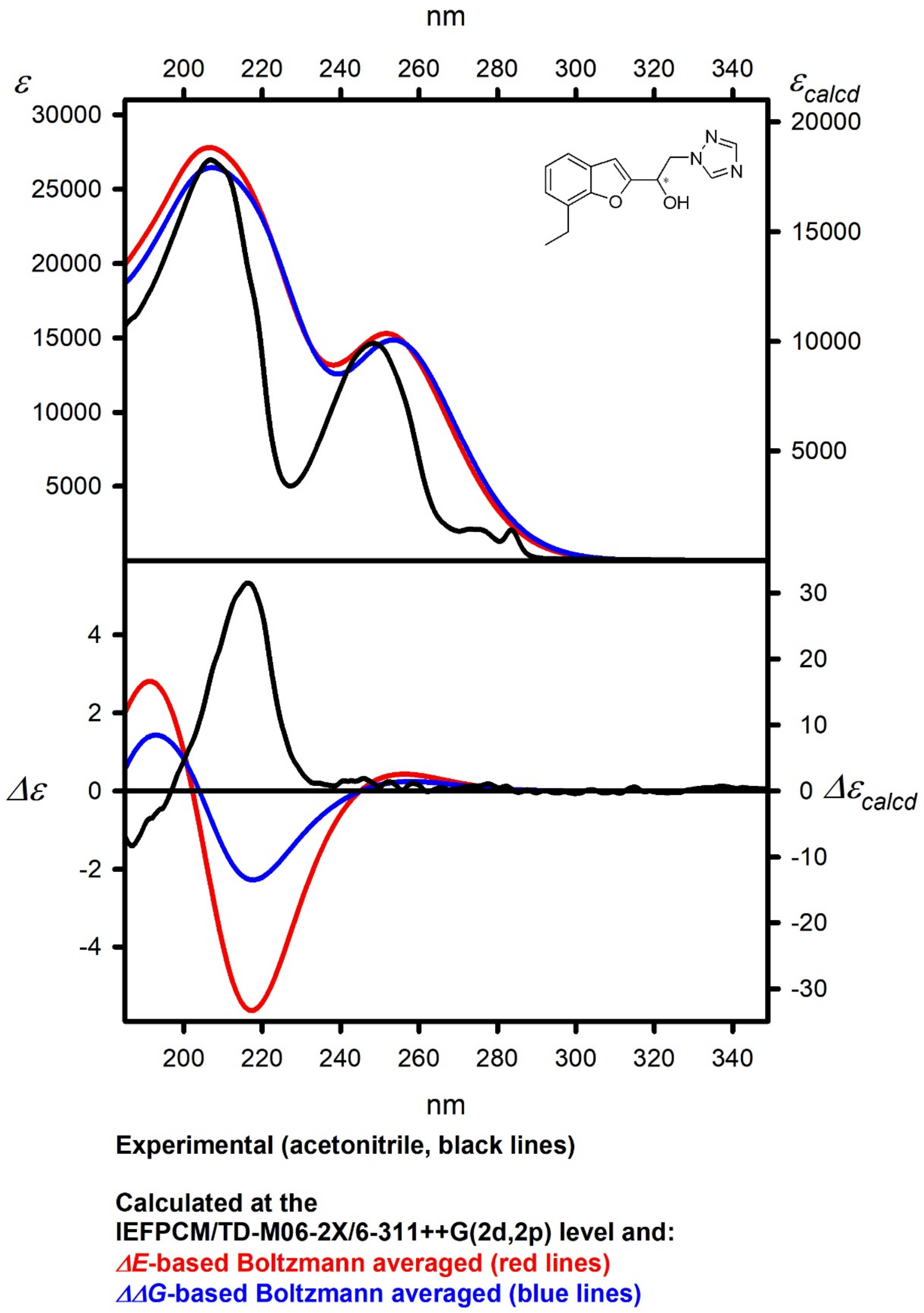

Figure S41. UV (upper panel) and ECD (lower panel) spectra of alcohol **18**, measured in acetonitrile (black lines), and calculated at the IEFPCM/TD-M06-2X/6-311++G(2p,2d) level and *ΔE*- (red lines) and *ΔΔG*-based Boltzmann averaged (blue lines). Wavelengths were corrected to match experimental UV maximum.

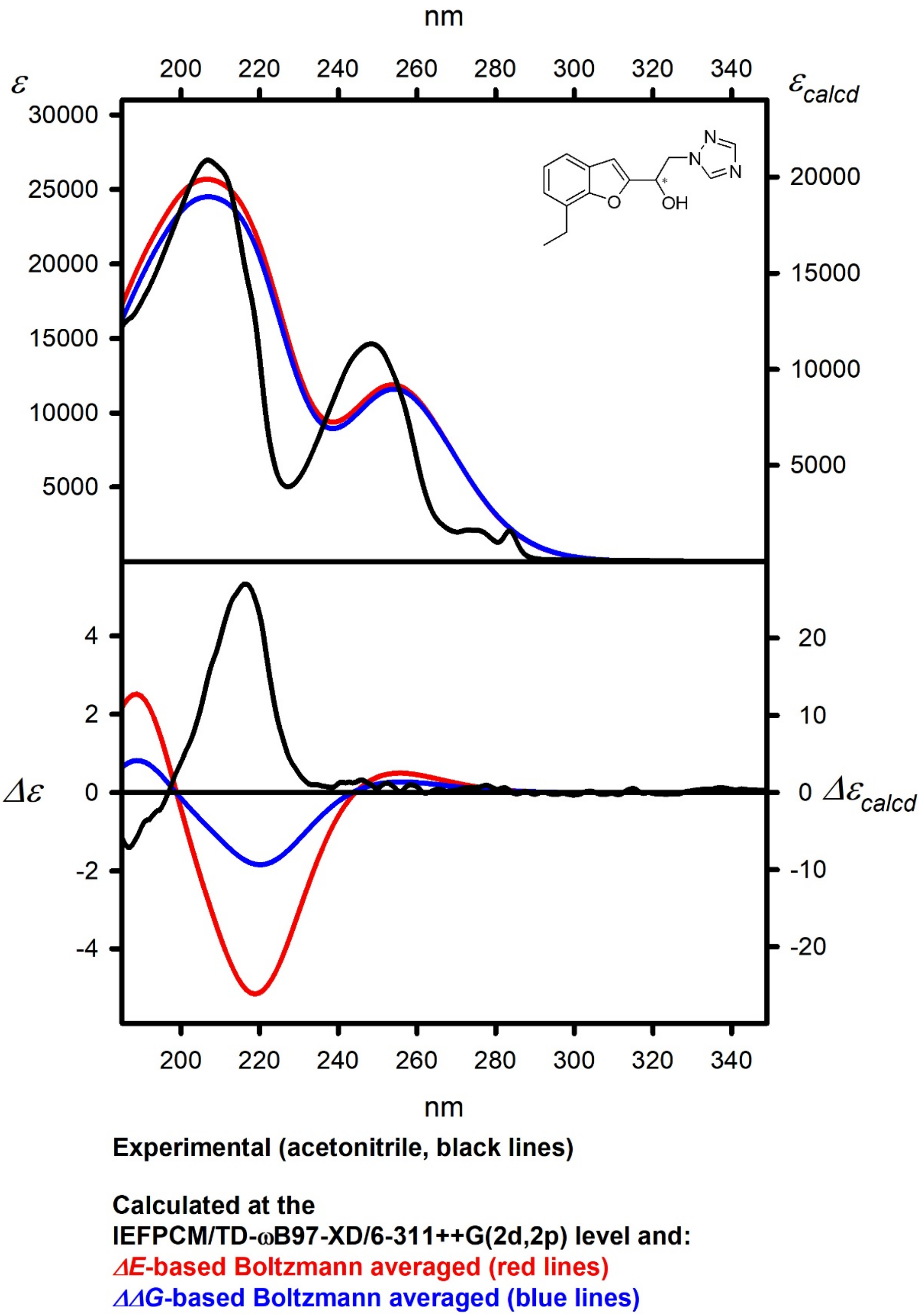

Figure S42. UV (upper panel) and ECD (lower panel) spectra of alcohol **18**, measured in acetonitrile (black lines), and calculated at the IEFPCM/TD-*ω*B97-XD/6-311++G(2p,2d) level and *ΔE*- (red lines) and *ΔΔG*-based Boltzmann averaged (blue lines). Wavelengths were corrected to match experimental UV maximum.

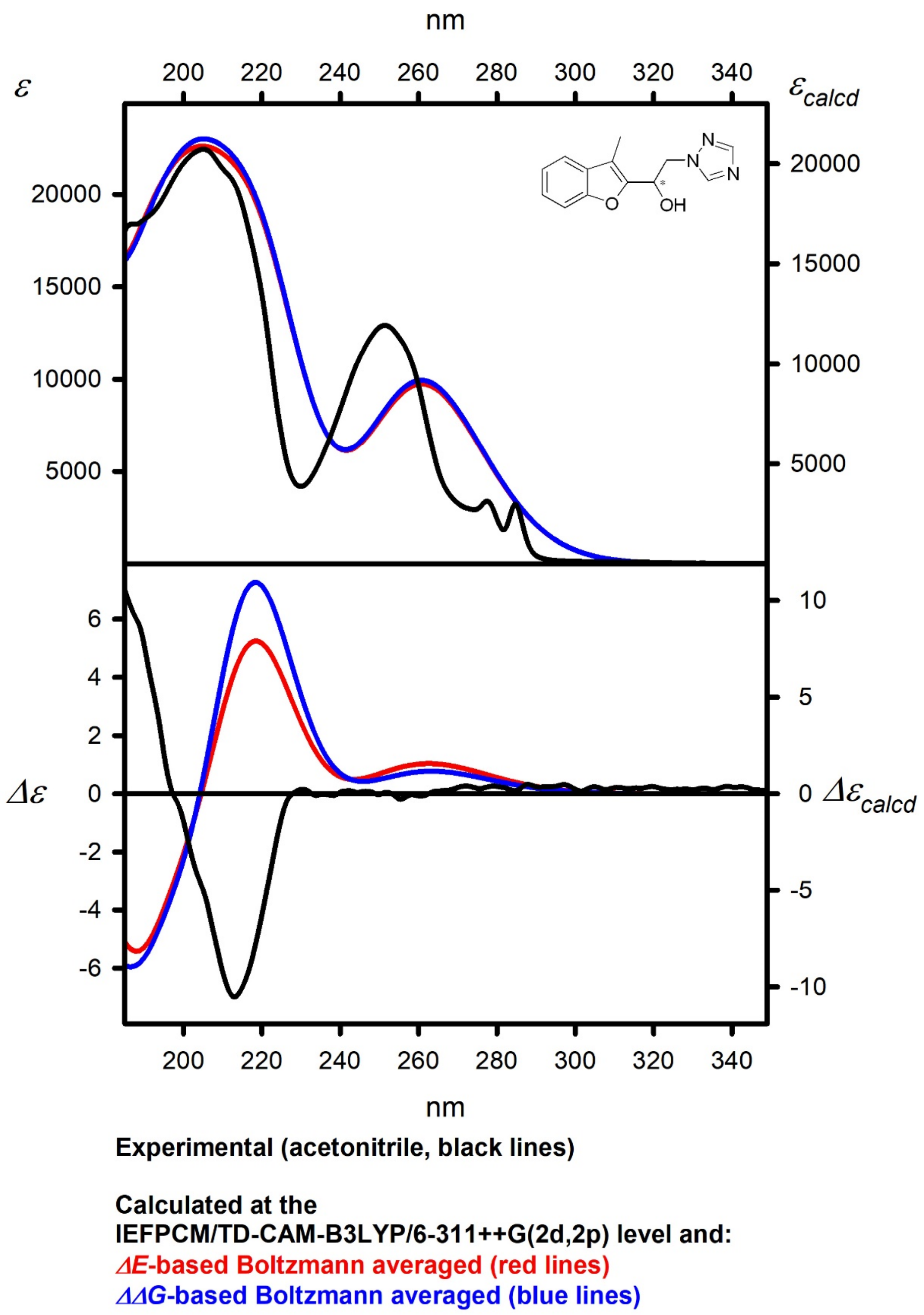

Figure S43. UV (upper panel) and ECD (lower panel) spectra of alcohol **19**, measured in acetonitrile (black lines), and calculated at the IEFPCM/TD-CAM-B3LYP/6-311++G(2p,2d) level and *ΔE*- (red lines) and *ΔΔG*-based Boltzmann averaged (blue lines). Wavelengths were corrected to match experimental UV maximum.

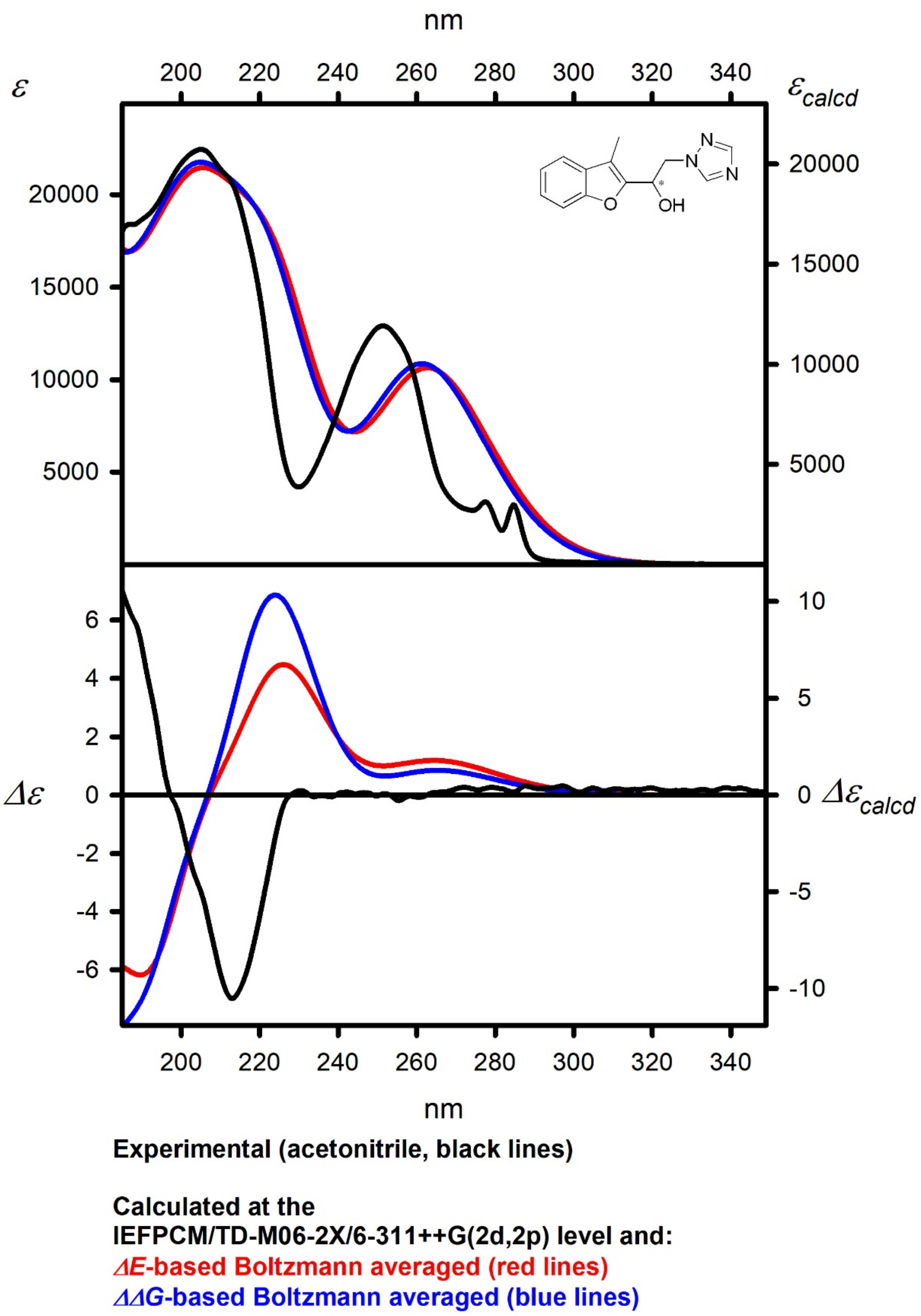

Figure S44. UV (upper panel) and ECD (lower panel) spectra of alcohol **19**, measured in acetonitrile (black lines), and calculated at the IEFPCM/TD-M06-2X/6-311++G(2p,2d) level and *ΔE*- (red lines) and *ΔΔG*-based Boltzmann averaged (blue lines). Wavelengths were corrected to match experimental UV maximum.

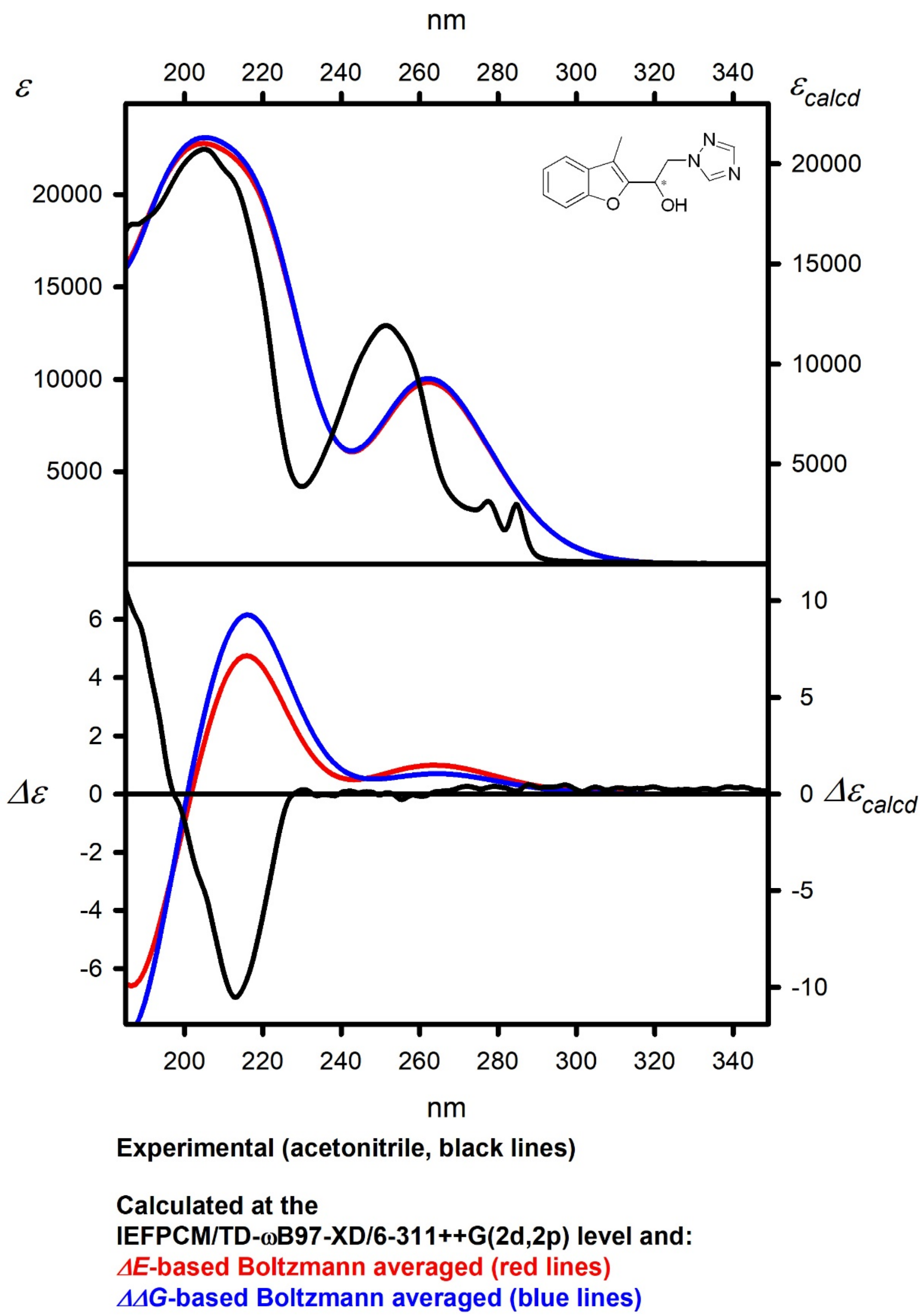

Figure S45. UV (upper panel) and ECD (lower panel) spectra of alcohol **19**, measured in acetonitrile (black lines), and calculated at the IEFPCM/TD-*ω*B97-XD/6-311++G(2p,2d) level and *ΔE*- (red lines) and *ΔΔG*-based Boltzmann averaged (blue lines). Wavelengths were corrected to match experimental UV maximum.

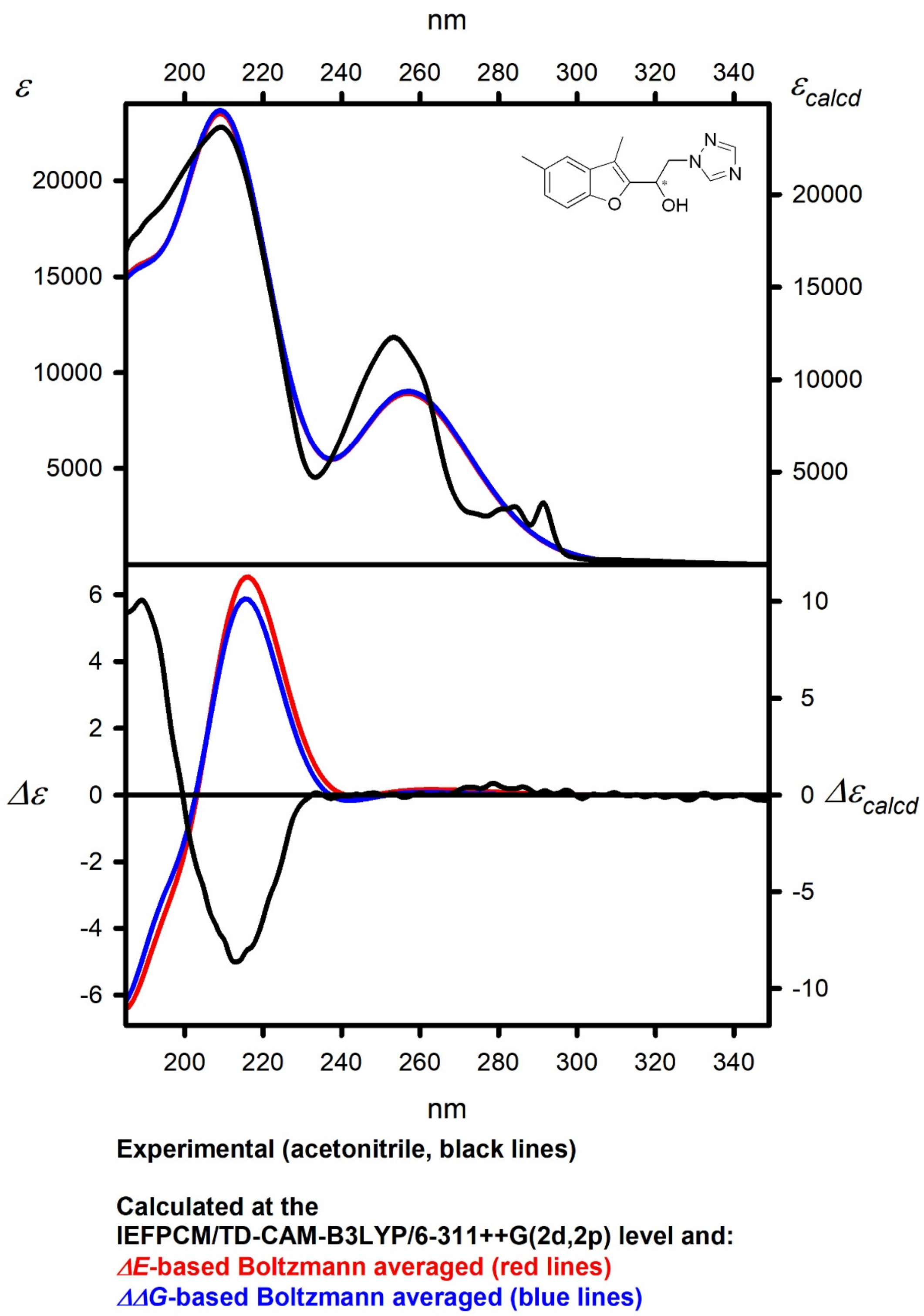

Figure S46. UV (upper panel) and ECD (lower panel) spectra of alcohol **20**, measured in acetonitrile (black lines), and calculated at the IEFPCM/TD-CAM-B3LYP/6-311++G(2p,2d) level and ΔE- (red lines) and $\Delta\Delta G$-based Boltzmann averaged (blue lines). Wavelengths were corrected to match experimental UV maximum.

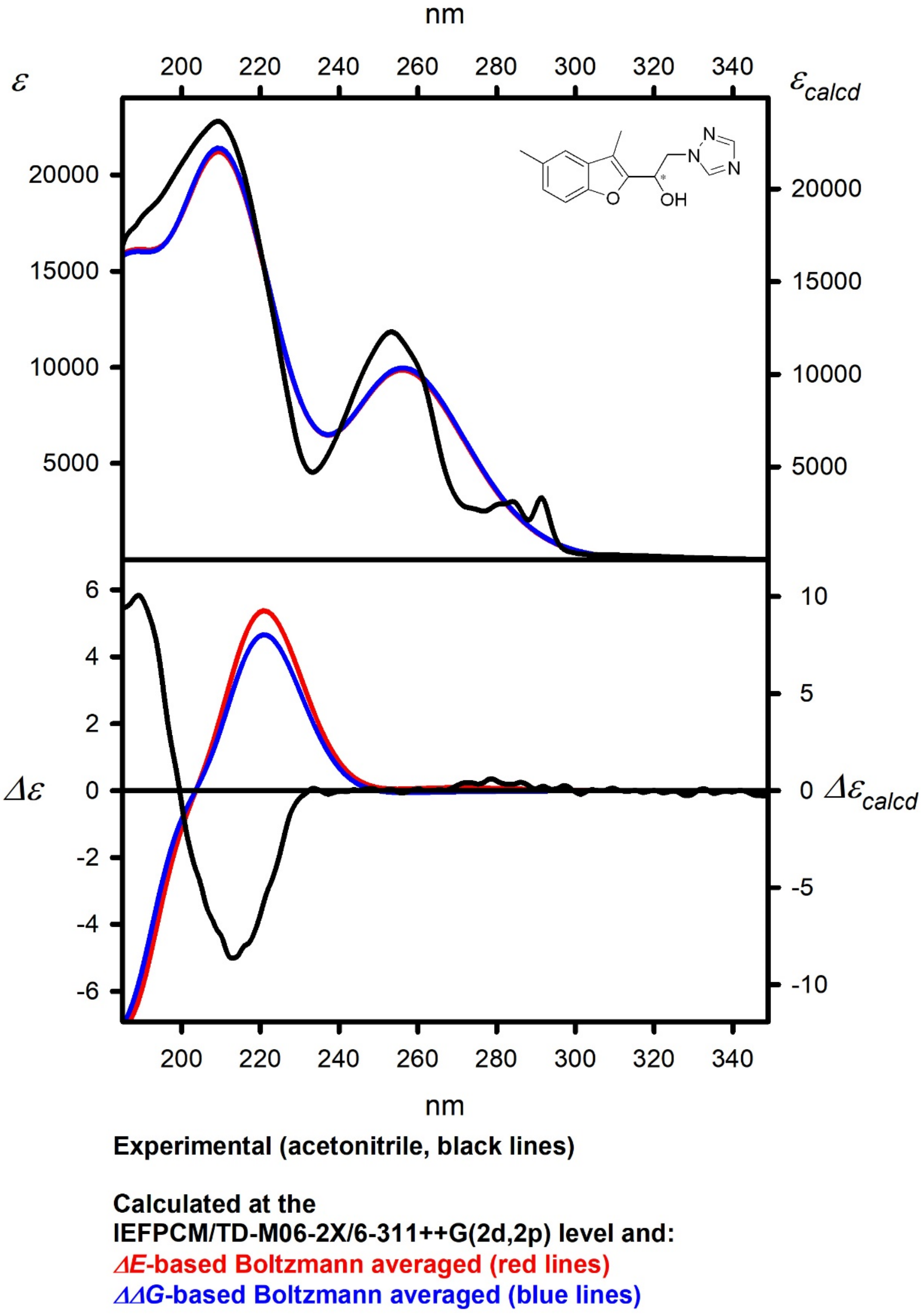

Figure S47. UV (upper panel) and ECD (lower panel) spectra of alcohol **20**, measured in acetonitrile (black lines), and calculated at the IEFPCM/TD-M06-2X/6-311++G(2p,2d) level and ΔE- (red lines) and $\Delta\Delta G$-based Boltzmann averaged (blue lines). Wavelengths were corrected to match experimental UV maximum.

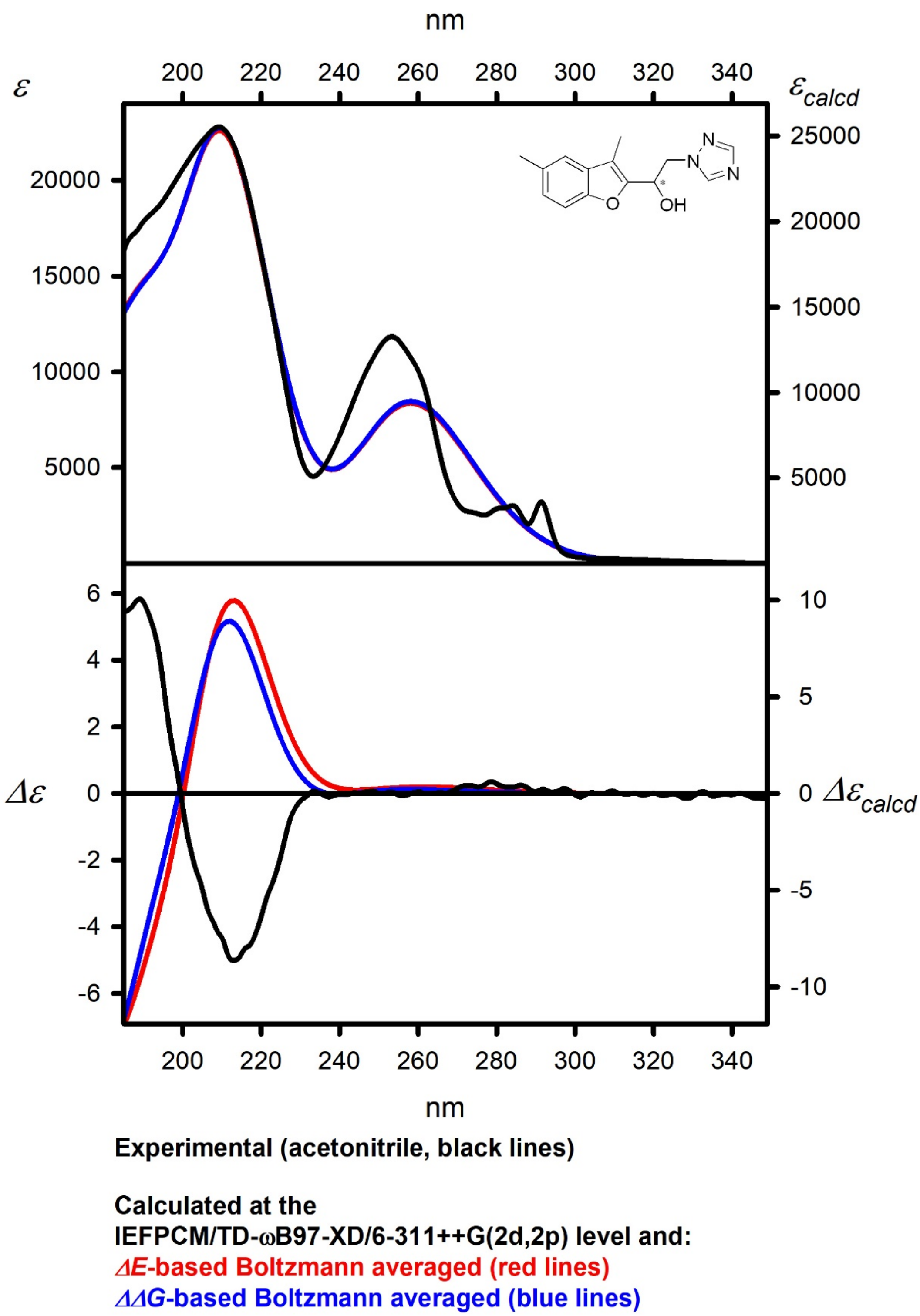

Figure S48. UV (upper panel) and ECD (lower panel) spectra of alcohol **20**, measured in acetonitrile (black lines), and calculated at the IEFPCM/TD-*ω*B97-XD/6-311++G(2p,2d) level and *ΔE*- (red lines) and *ΔΔG*-based Boltzmann averaged (blue lines). Wavelengths were corrected to match experimental UV maximum.

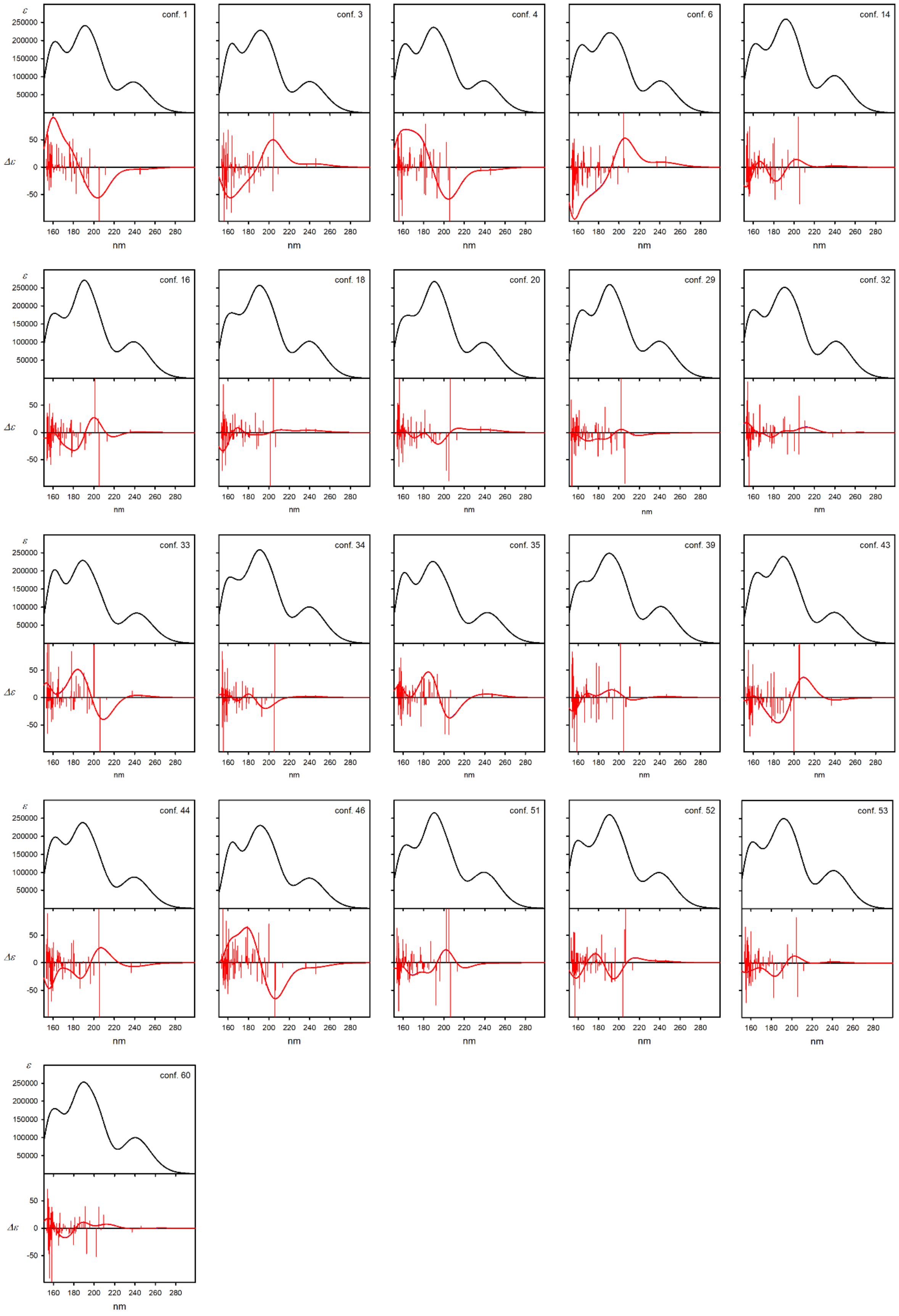

Figure S49. UV (upper panels) and ECD (lower panels) spectra calculated at the IEFPCM/TD-CAM-B3LYP/6-311++G(2d,2p) level for individual low-energy conformers of **13**. Wavelengths were not corrected.

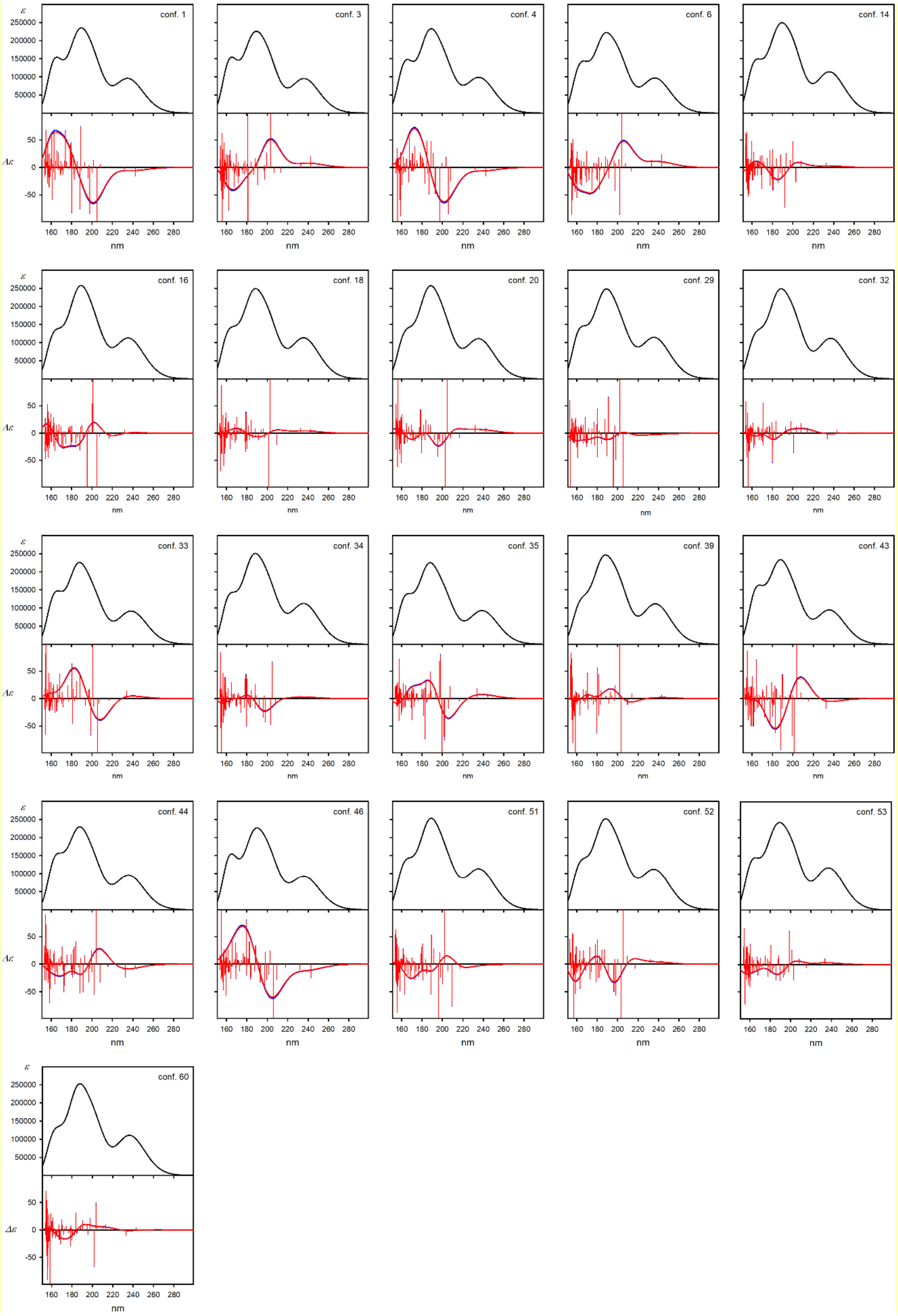

Figure S50. UV (upper panels) and ECD (lower panels) spectra calculated at the IEFPCM/TD-M06-2X/6-311++G(2d,2p) level for individual low-energy conformers of **13**. Wavelengths were not corrected.

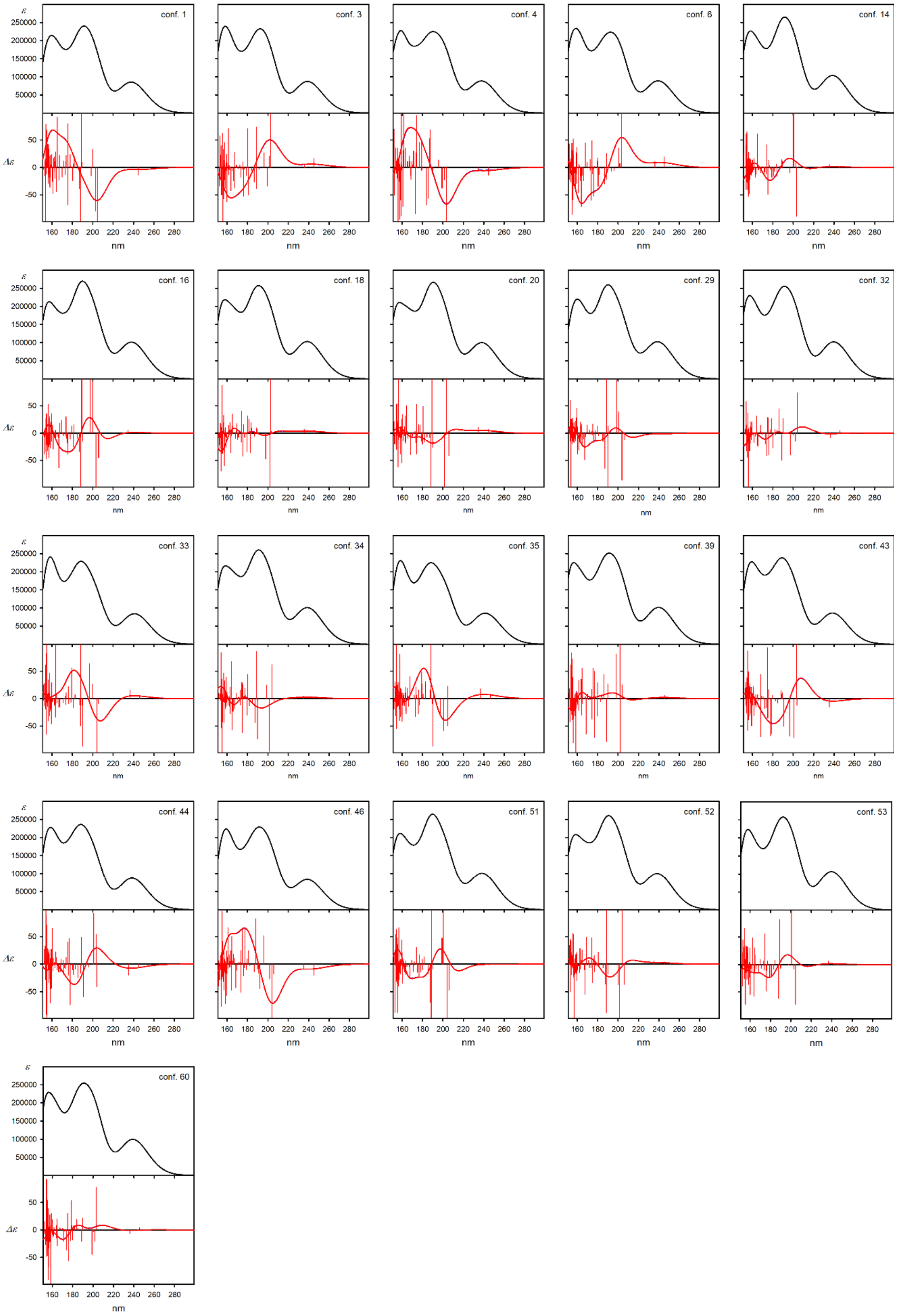

Figure S51. UV (upper panels) and ECD (lower panels) spectra calculated at the IEFPCM/TD-ωB97-XD/6-311++G(2d,2p) level for individual low-energy conformers of **13**. Wavelengths were not corrected.

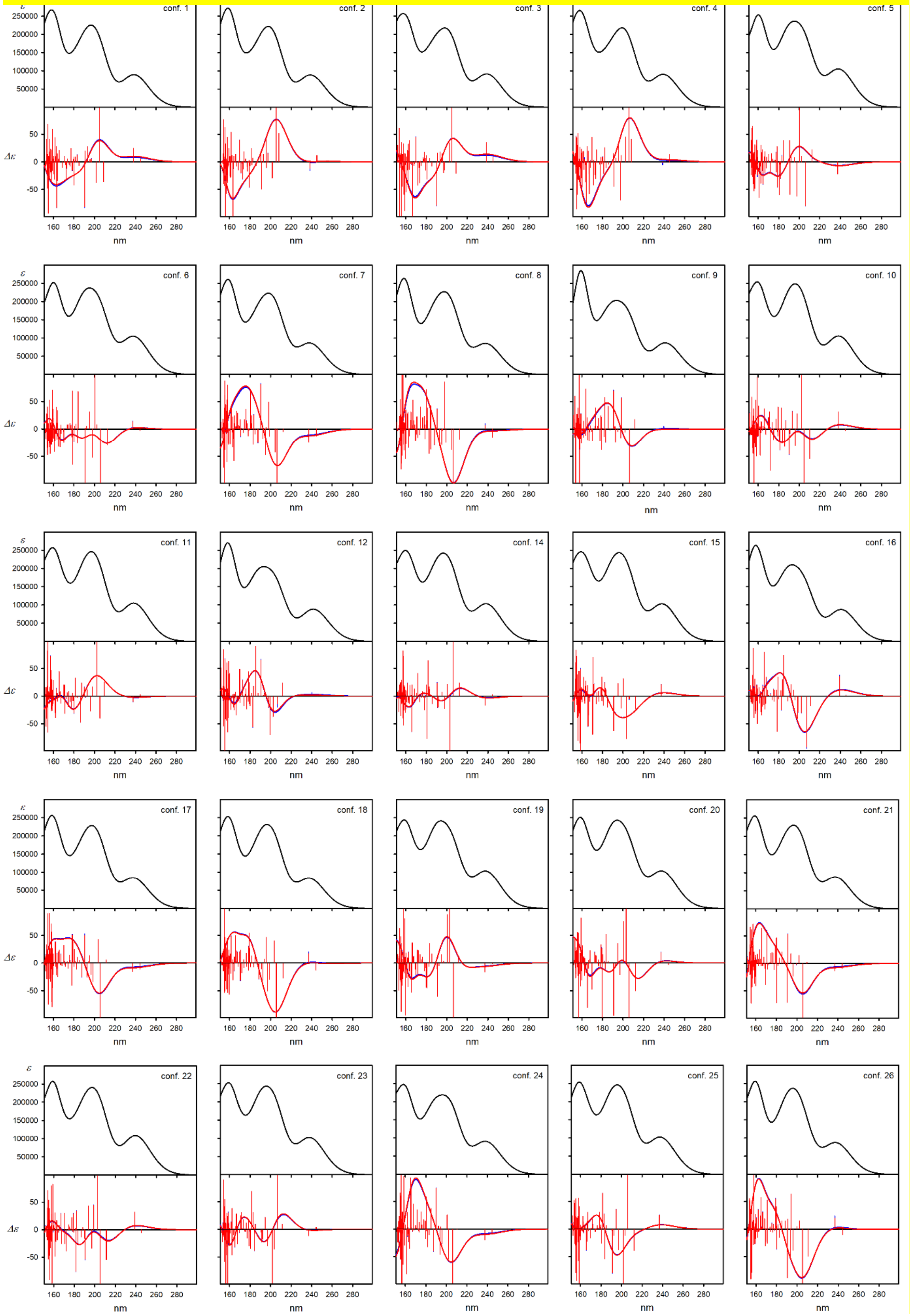

Figure S52. UV (upper panels) and ECD (lower panels) spectra calculated at the IEFPCM/TD-CAM-B3LYP/6-311++G(2d,2p) level for individual low-energy conformers of **14**. Wavelengths were not corrected.

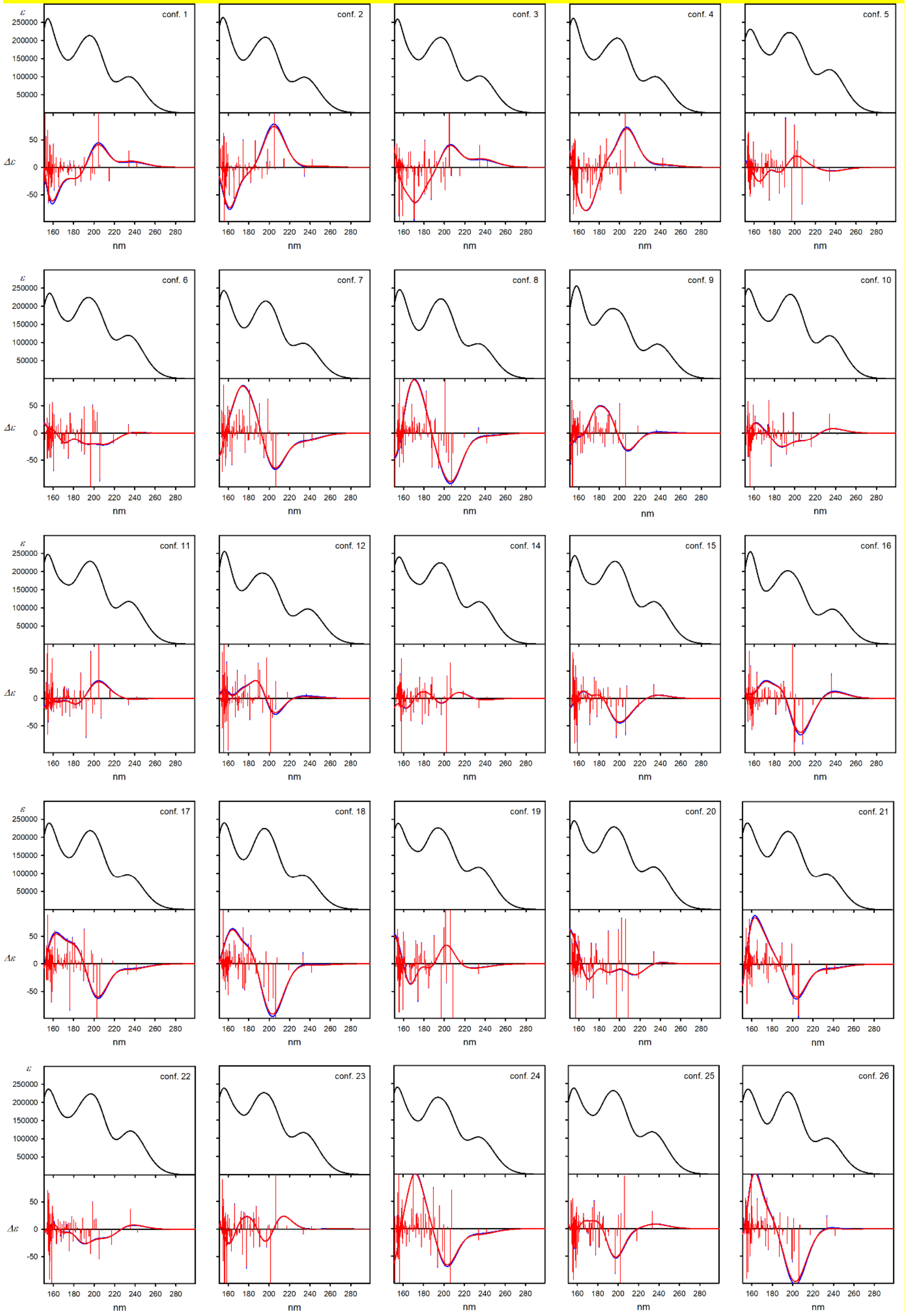
conf. 1
conf. 2
conf. 3
conf. 4
conf. 5
conf. 6
conf. 7
conf. 8
conf. 9
conf. 10
conf. 11
conf. 12
conf. 14
conf. 15
conf. 16
conf. 17
conf. 18
conf. 19
conf. 20
conf. 21
conf. 22
conf. 23
conf. 24
conf. 25
conf. 26
ε
Δε
250000
200000
150000
100000
50000
50
0
-50
160 180 200 220 240 260 280
nm

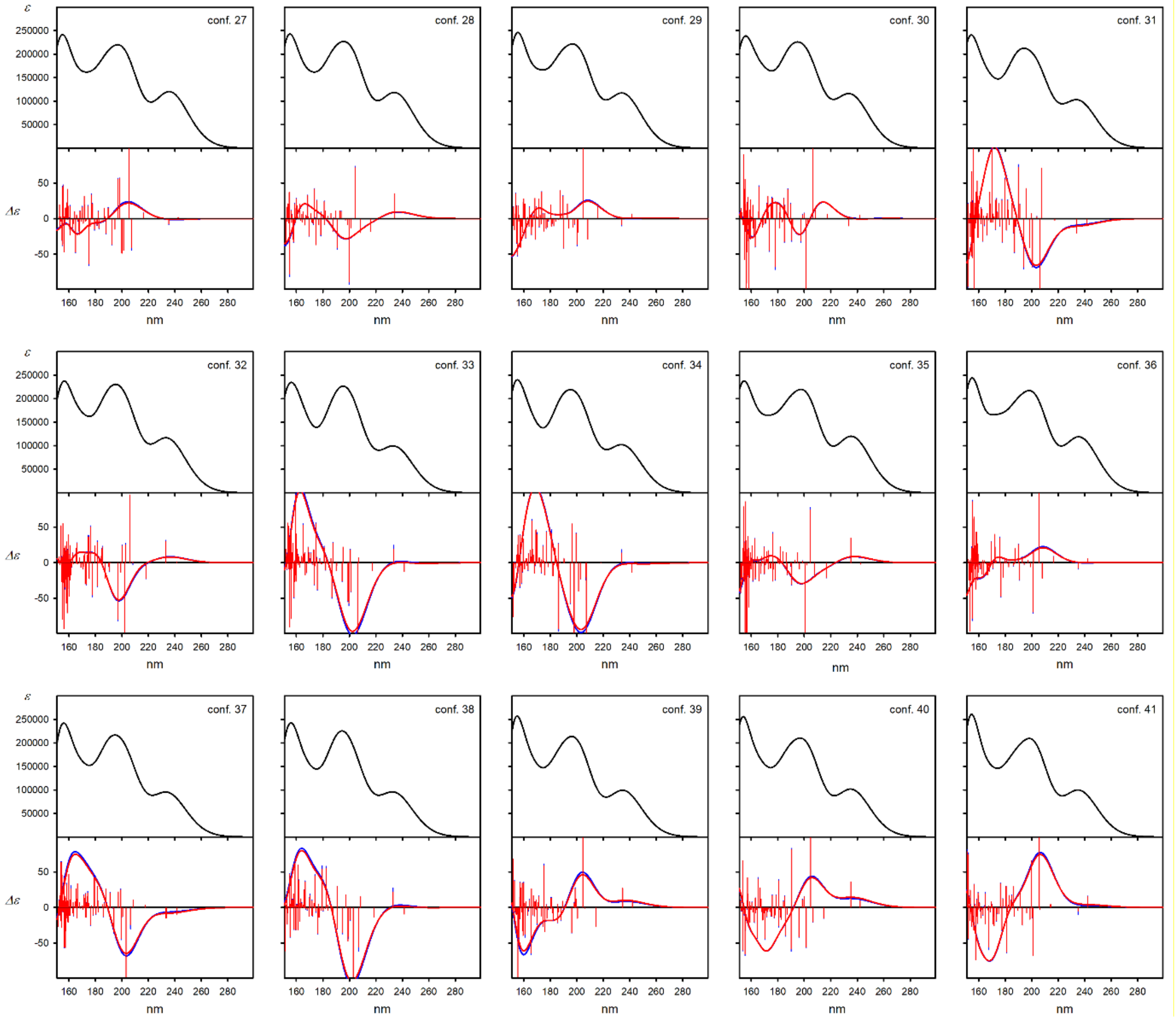

Figure S53. UV (upper panels) and ECD (lower panels) spectra calculated at the IEFPCM/TD-M06-2X/6-311++G(2d,2p) level for individual low-energy conformers of **14**. Wavelengths were not corrected.

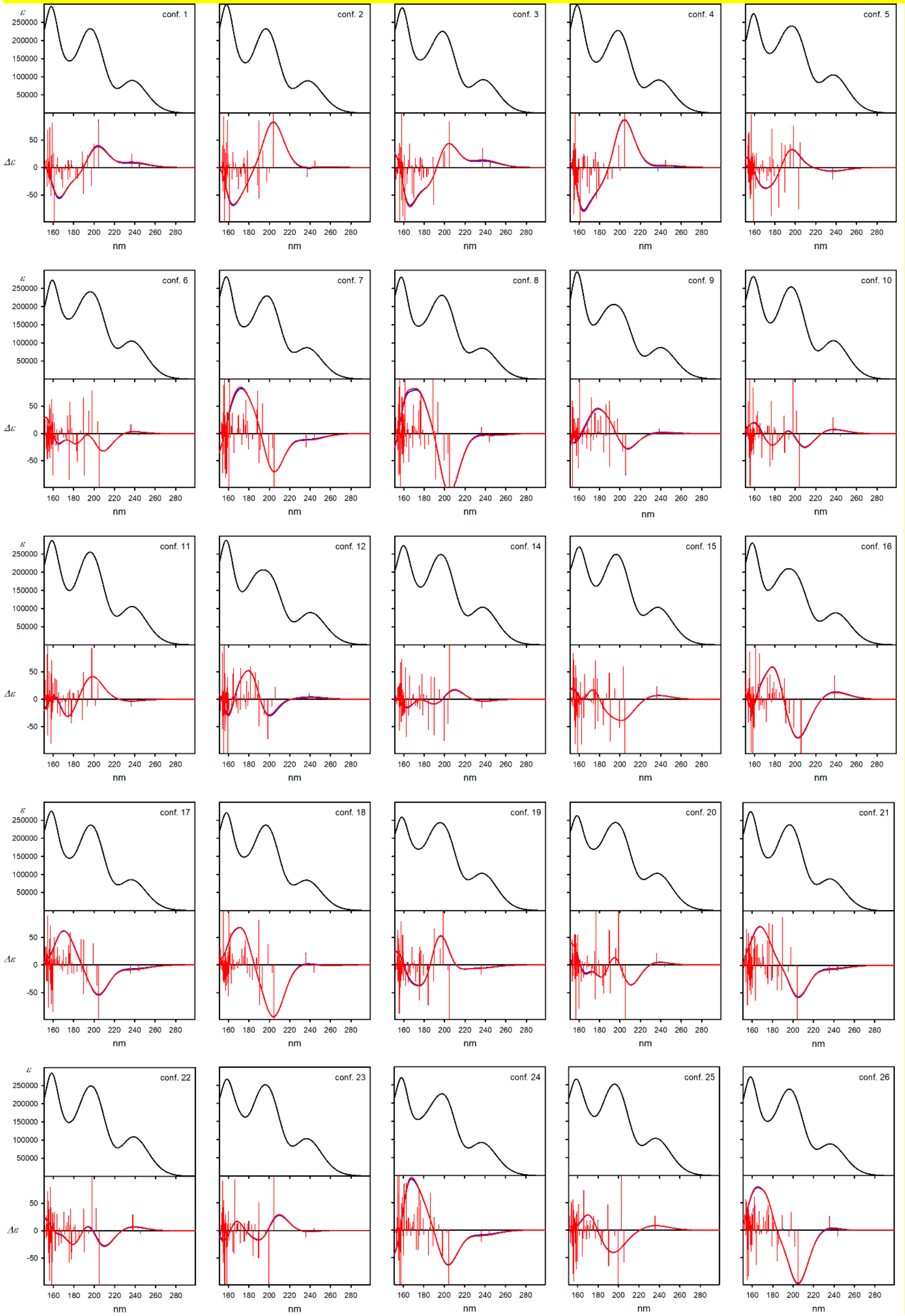
conf. 1
conf. 2
conf. 3
conf. 4
conf. 5
conf. 6
conf. 7
conf. 8
conf. 9
conf. 10
conf. 11
conf. 12
conf. 14
conf. 15
conf. 16
conf. 17
conf. 18
conf. 19
conf. 20
conf. 21
conf. 22
conf. 23
conf. 24
conf. 25
conf. 26
ε
Δε
nm

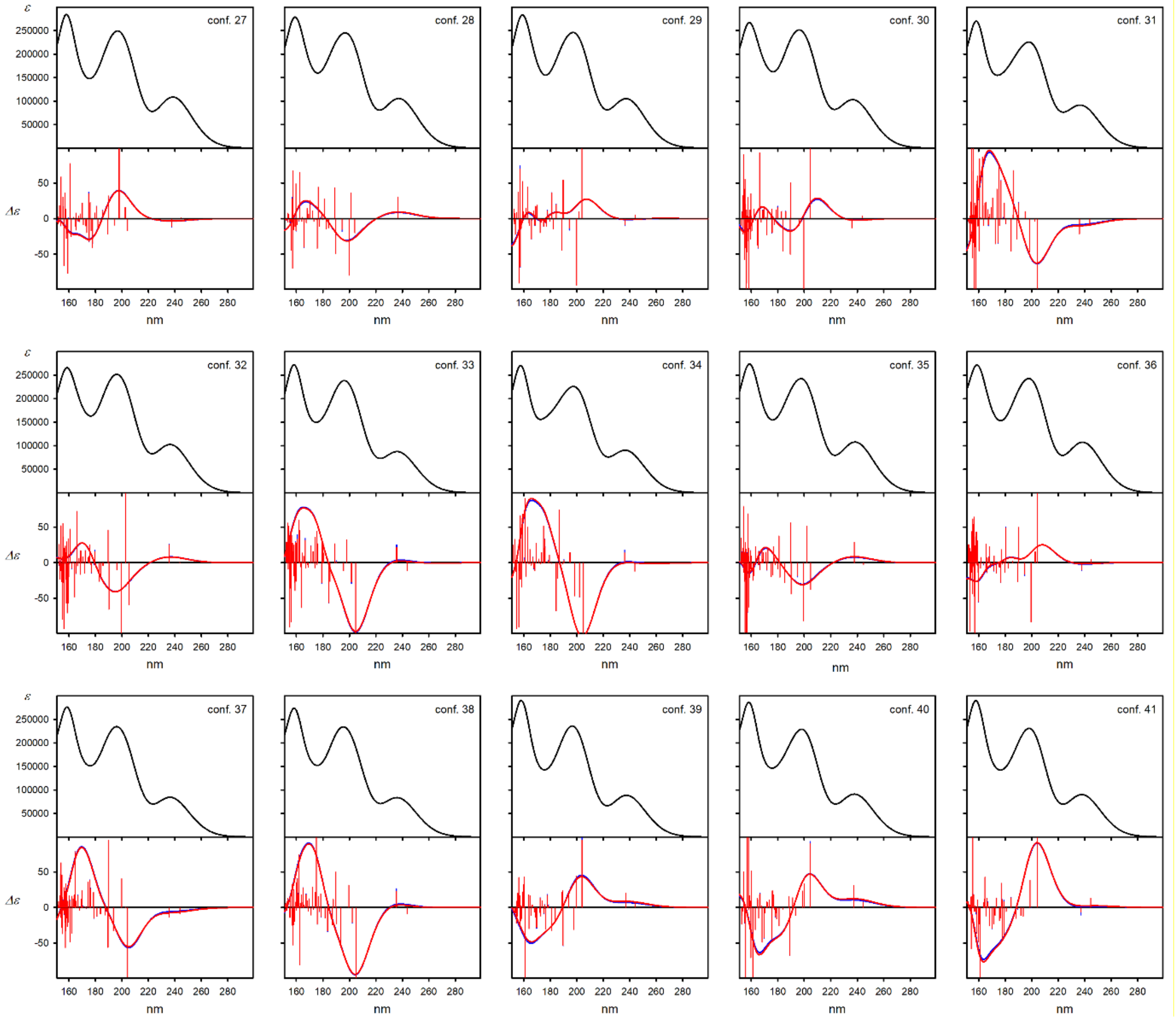

Figure S54. UV (upper panels) and ECD (lower panels) spectra calculated at the IEFPCM/TD-*ω*B97-XD/6-311++G(2d,2p) level for individual low-energy conformers of **14**. Wavelengths were not corrected.

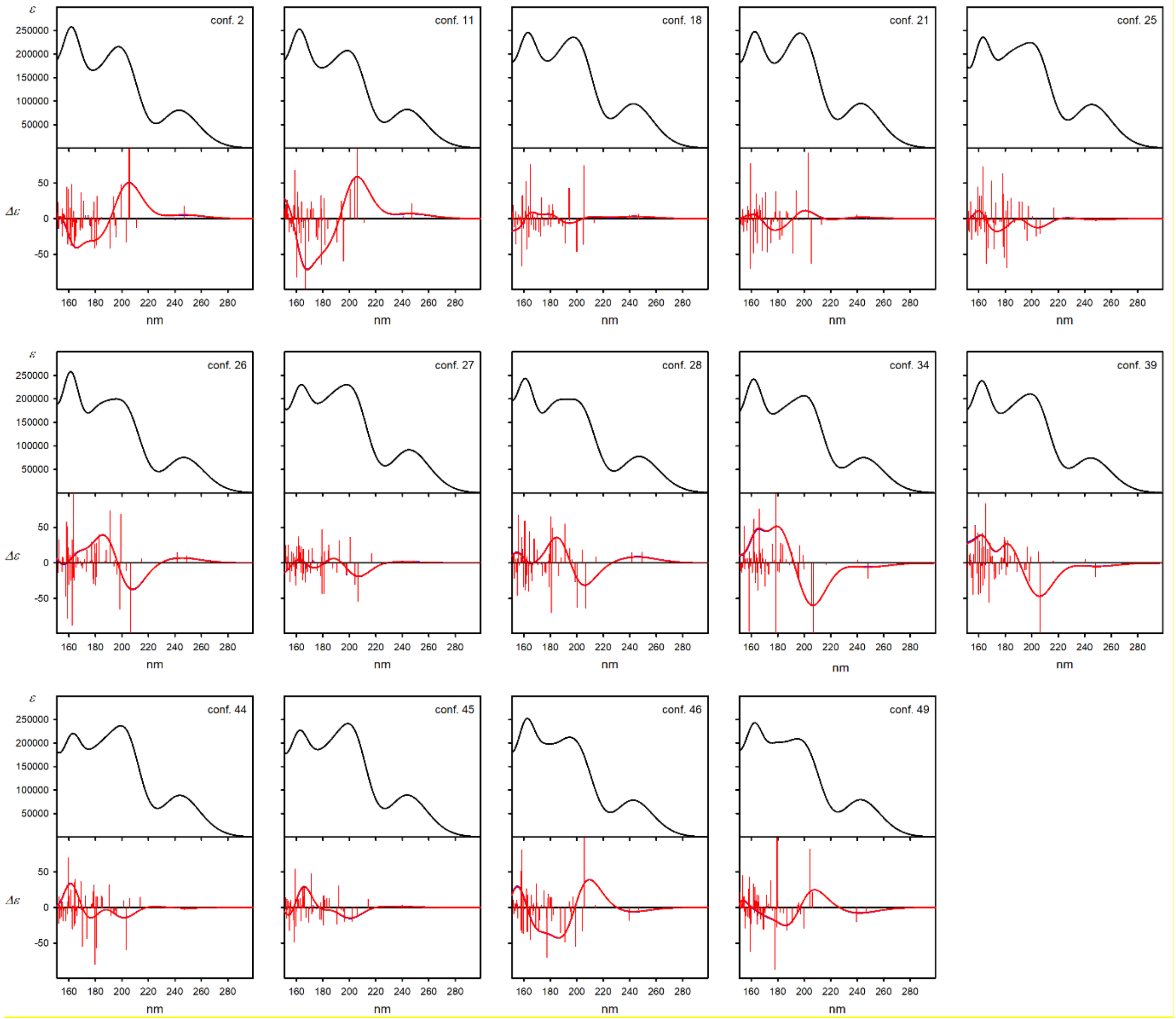

Figure S55. UV (upper panels) and ECD (lower panels) spectra calculated at the IEFPCM/TD-CAM-B3LYP/6-311++G(2d,2p) level for individual low-energy conformers of **15**. Wavelengths were not corrected.

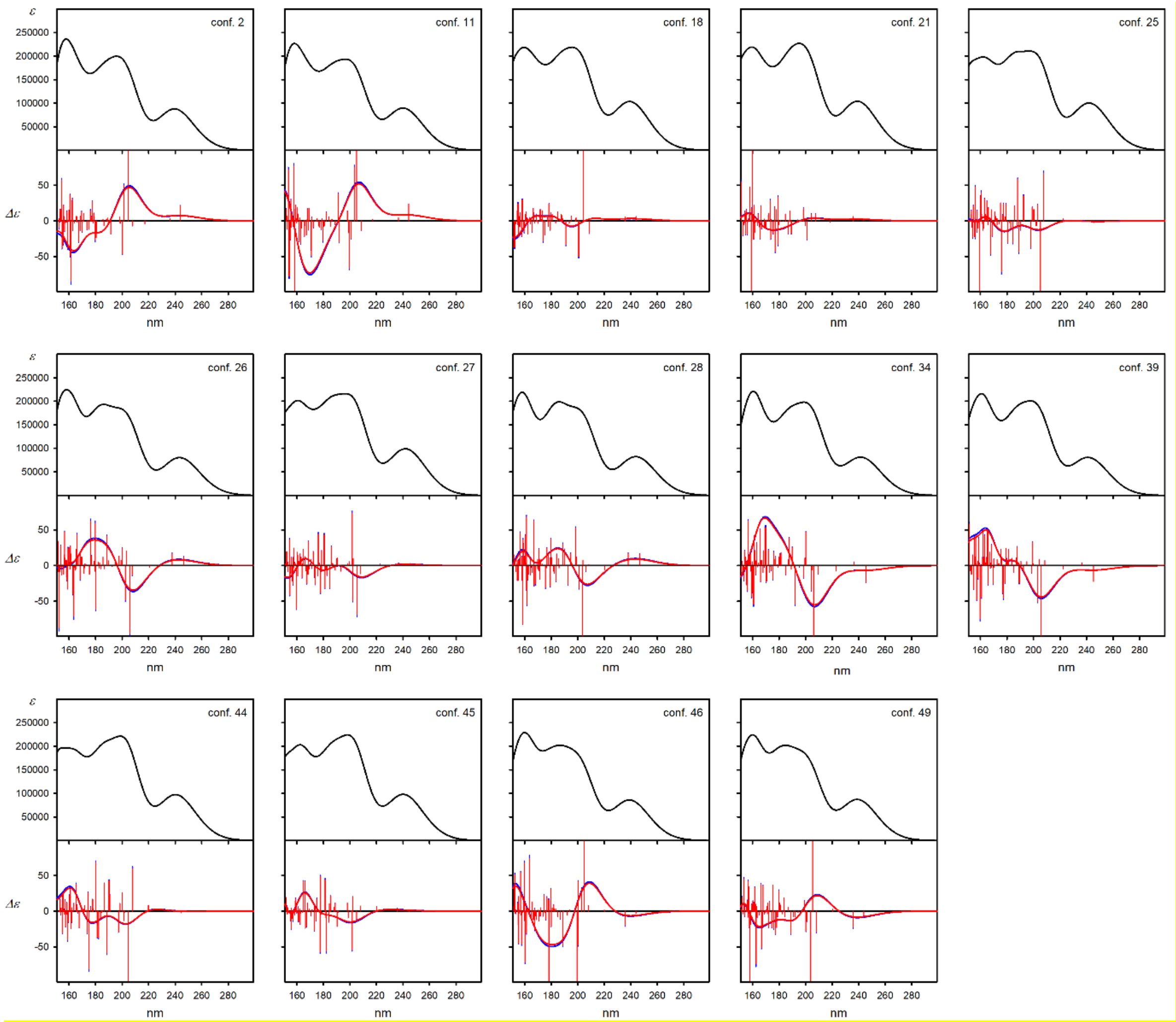

Figure S56. UV (upper panels) and ECD (lower panels) spectra calculated at the IEFPCM/TD-M06-2X/6-311++G(2d,2p) level for individual low-energy conformers of **15**. Wavelengths were not corrected.

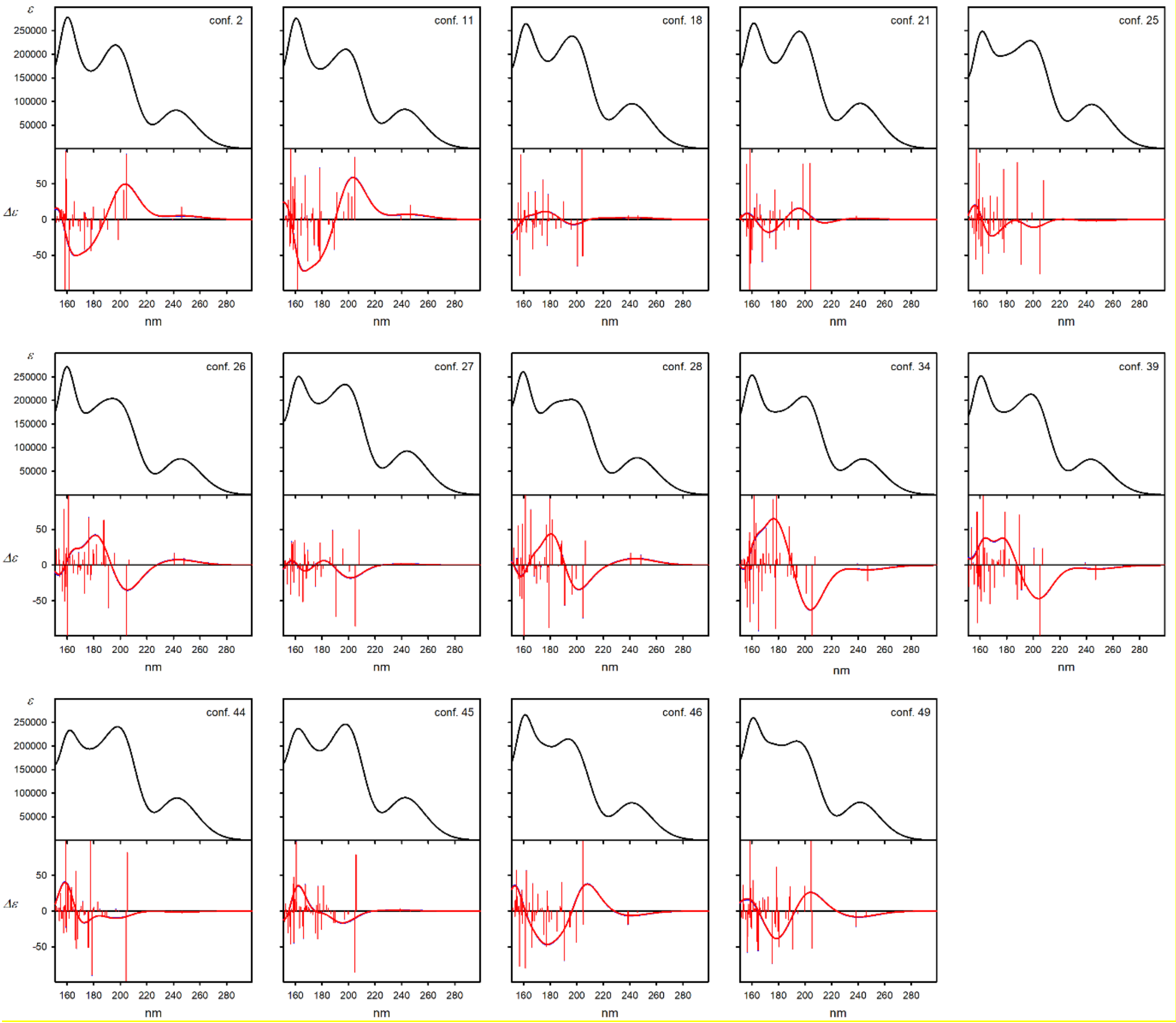

Figure S57. UV (upper panels) and ECD (lower panels) spectra calculated at the IEFPCM/TD-*ω*B97-XD/6-311++G(2d,2p) level for individual low-energy conformers of **15**. Wavelengths were not corrected.

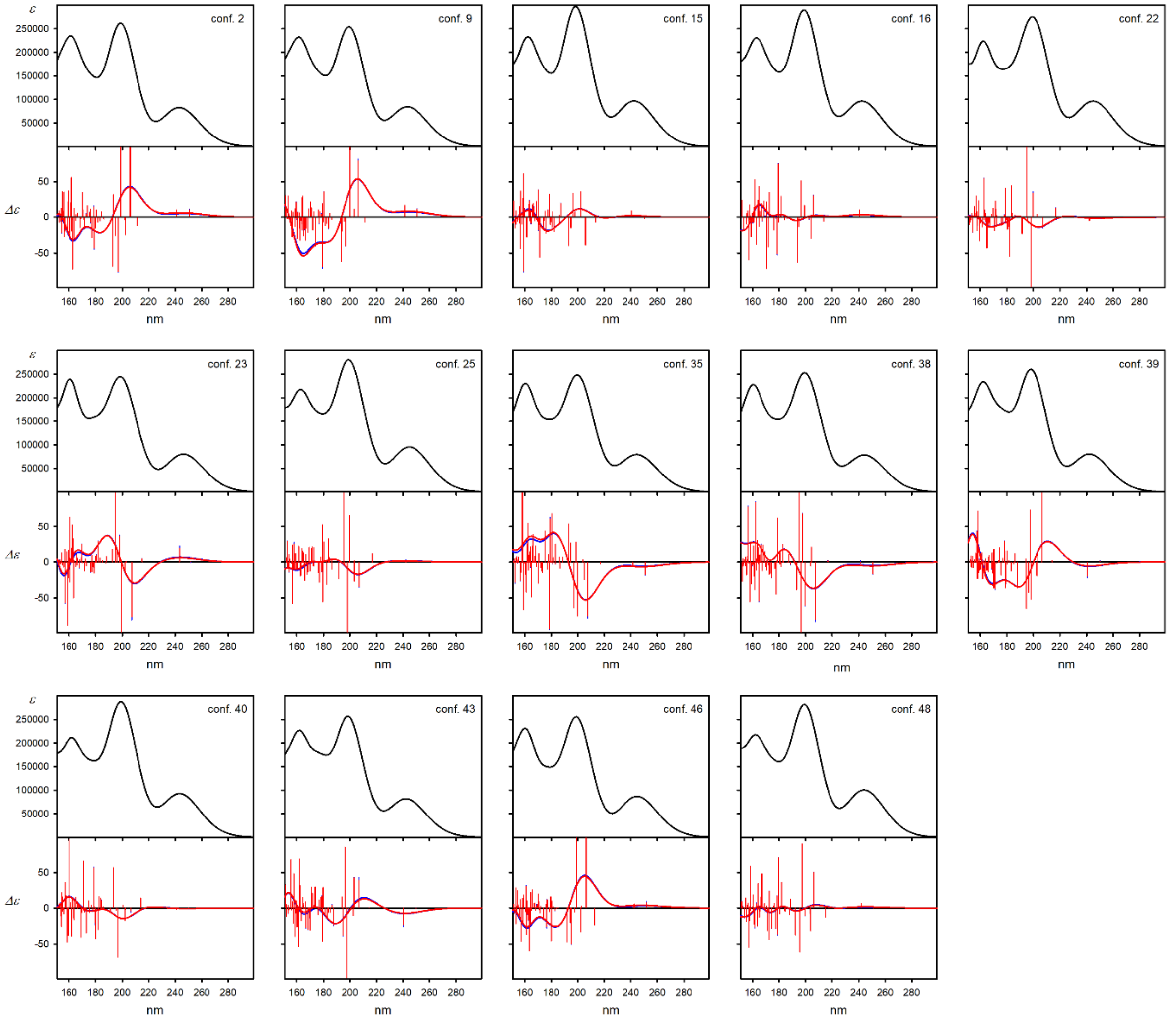

Figure S58. UV (upper panels) and ECD (lower panels) spectra calculated at the IEFPCM/TD-CAM-B3LYP/6-311++G(2d,2p) level for individual low-energy conformers of **16**. Wavelengths were not corrected.

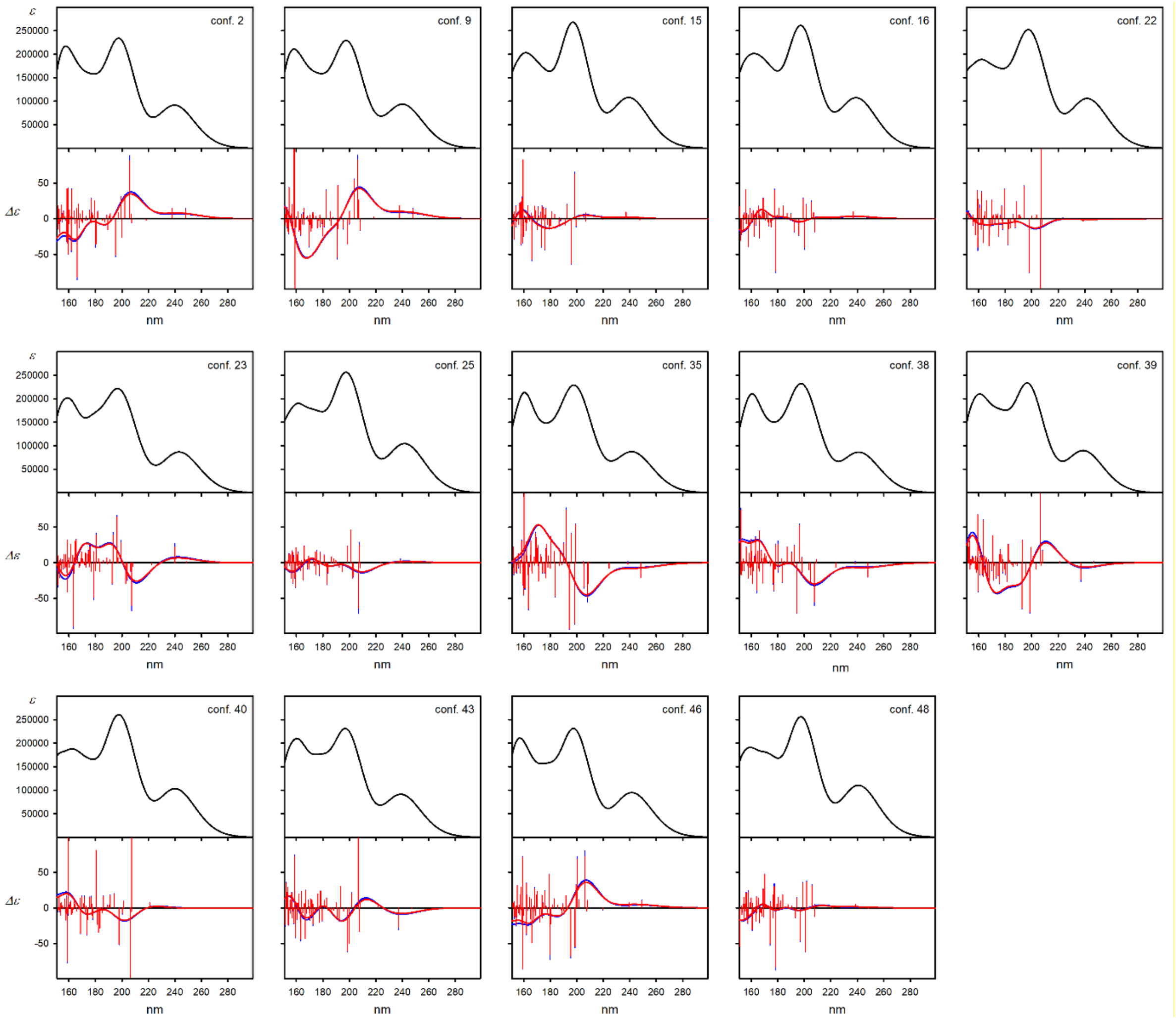

Figure S59. UV (upper panels) and ECD (lower panels) spectra calculated at the IEFPCM/TD-M06-2X/6-311++G(2d,2p) level for individual low-energy conformers of **16**. Wavelengths were not corrected.

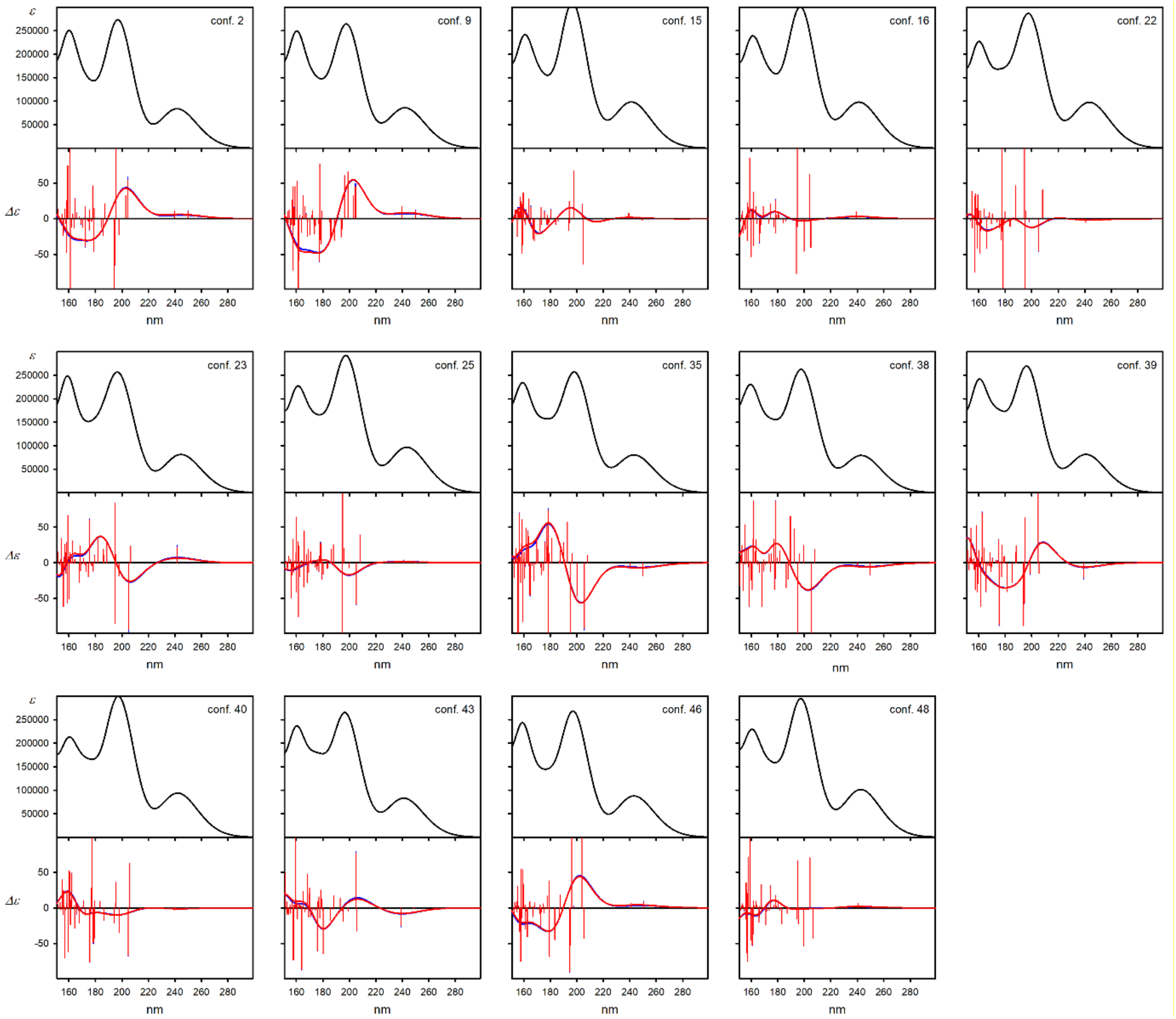

Figure S60. UV (upper panels) and ECD (lower panels) spectra calculated at the IEFPCM/TD-*ω*B97-XD/6-311++G(2d,2p) level for individual low-energy conformers of **16**. Wavelengths were not corrected.

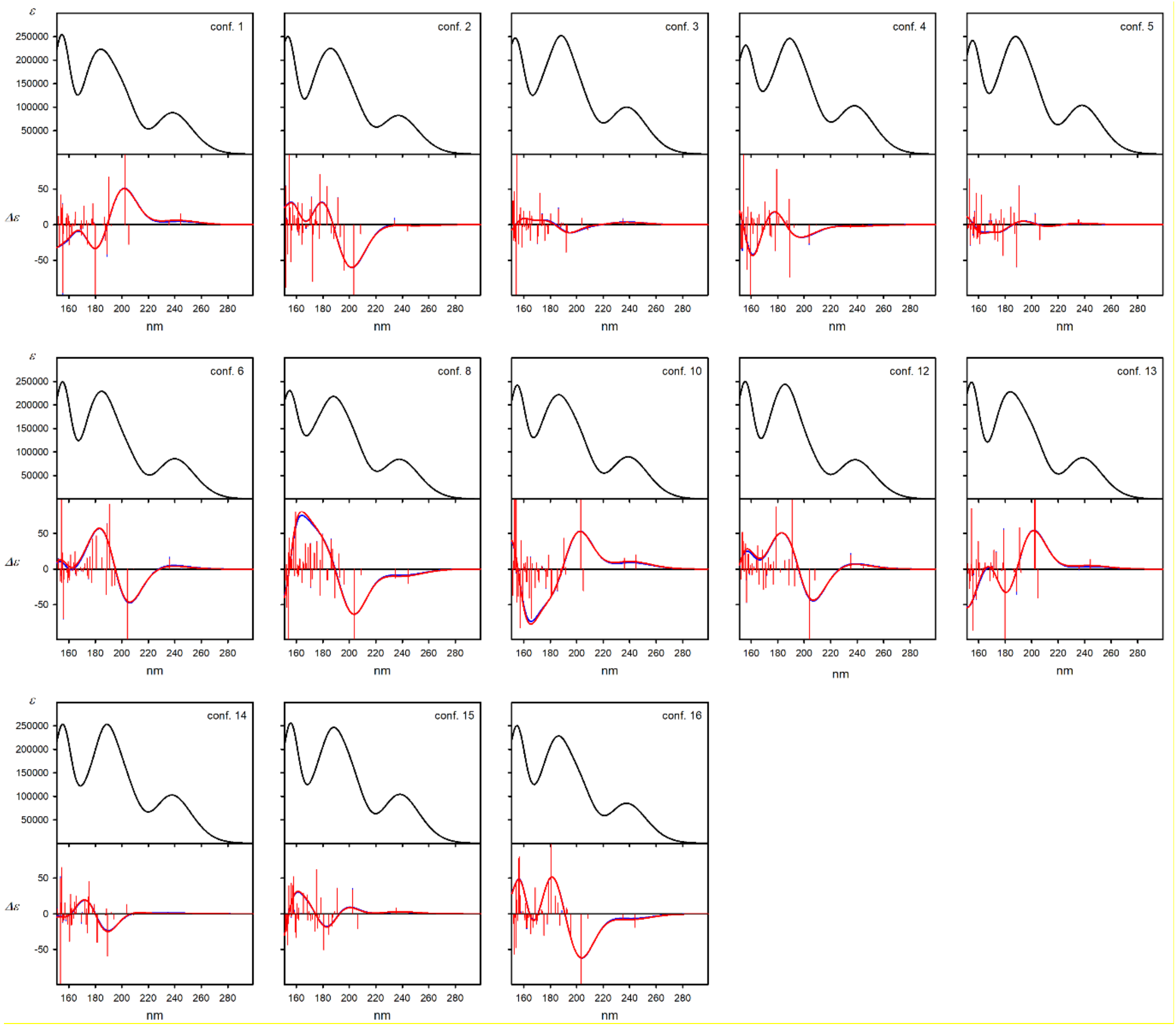

Figure S61. UV (upper panels) and ECD (lower panels) spectra calculated at the IEFPCM/TD-CAM-B3LYP/6-311++G(2d,2p) level for individual low-energy conformers of **17**. Wavelengths were not corrected.

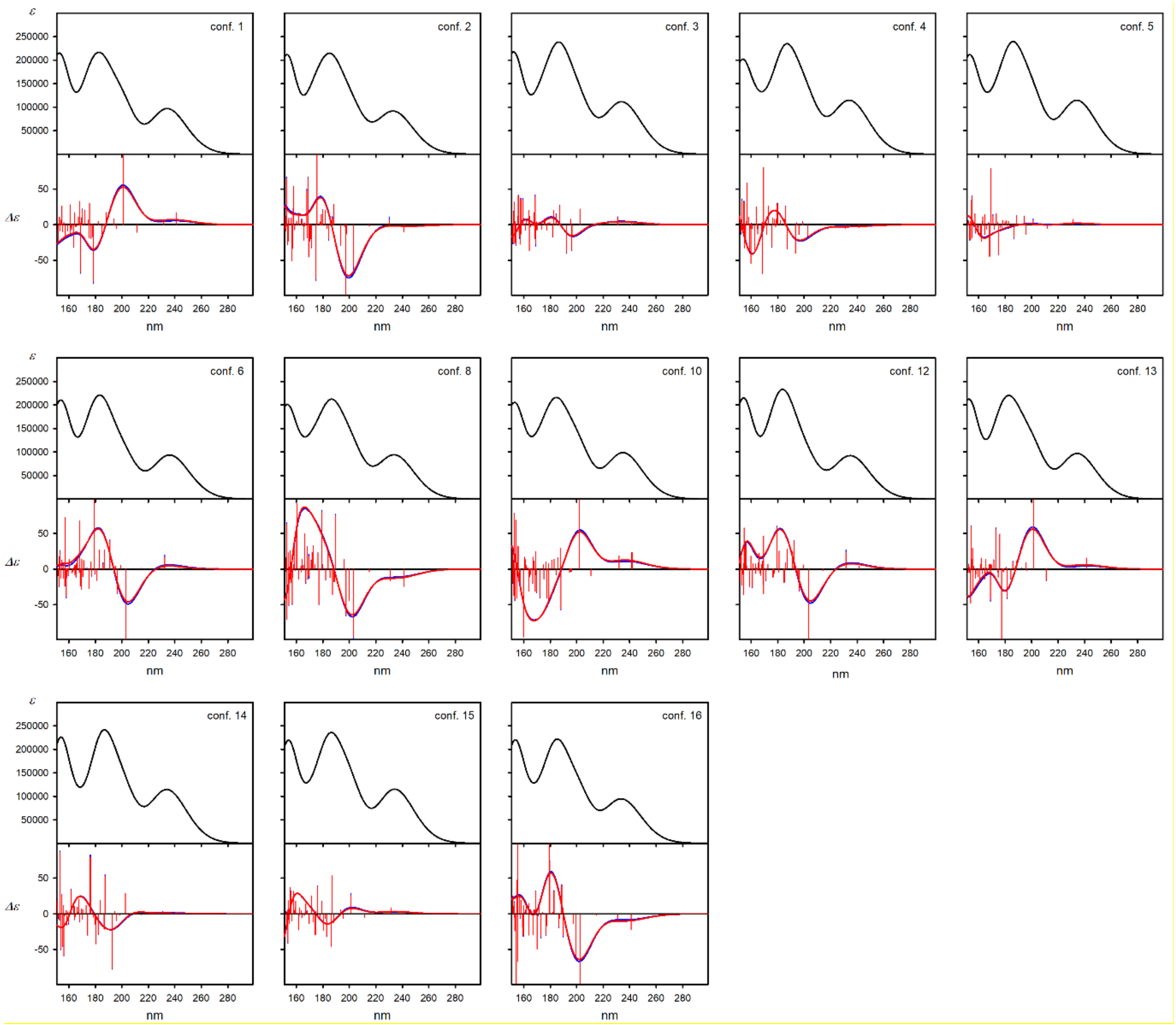

Figure S62. UV (upper panels) and ECD (lower panels) spectra calculated at the IEFPCM/TD-M06-2X/6-311++G(2d,2p) level for individual low-energy conformers of **17**. Wavelengths were not corrected.

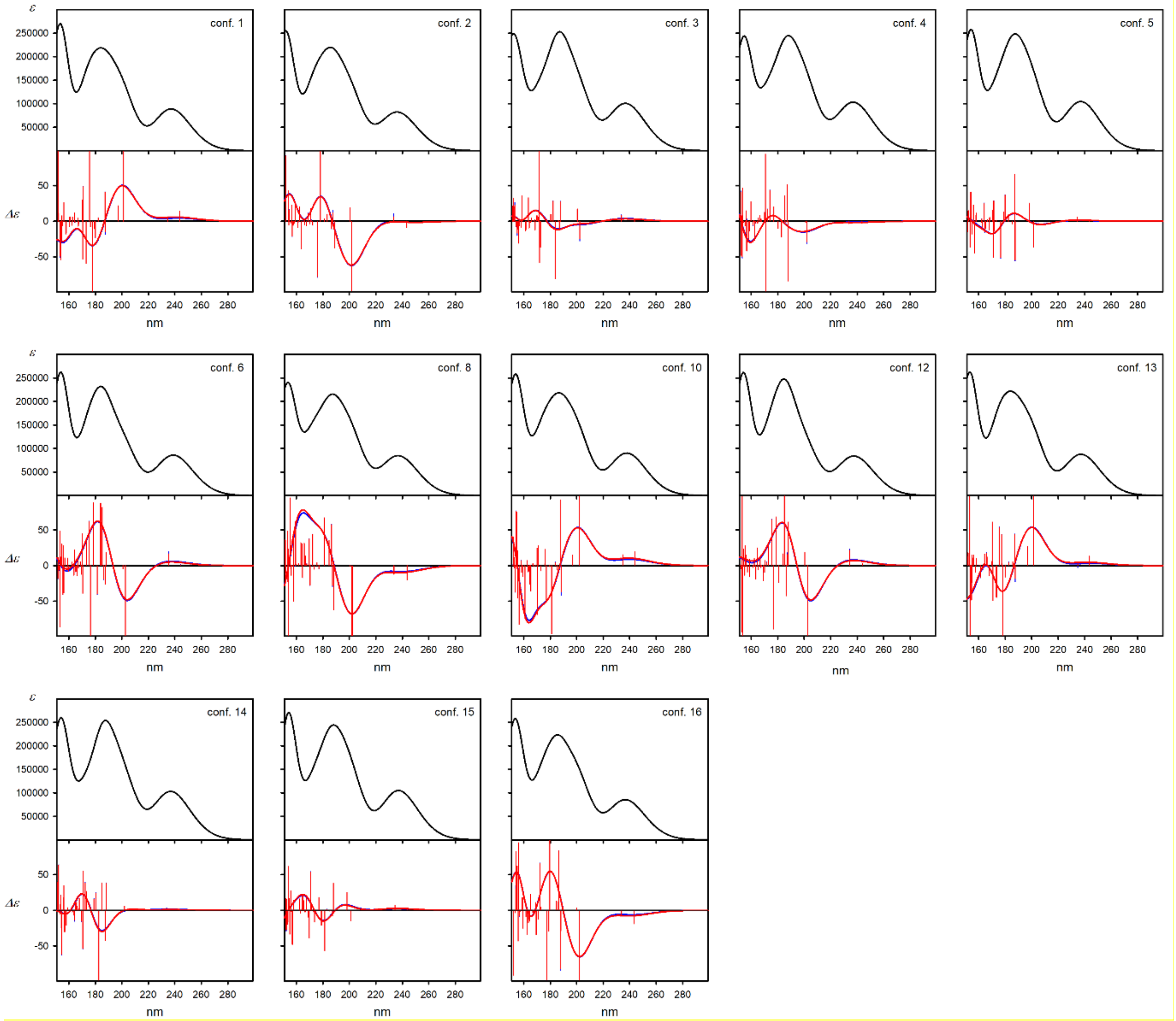

Figure S63. UV (upper panels) and ECD (lower panels) spectra calculated at the IEFPCM/TD-ωB97-XD/6-311++G(2d,2p) level for individual low-energy conformers of **17**. Wavelengths were not corrected.

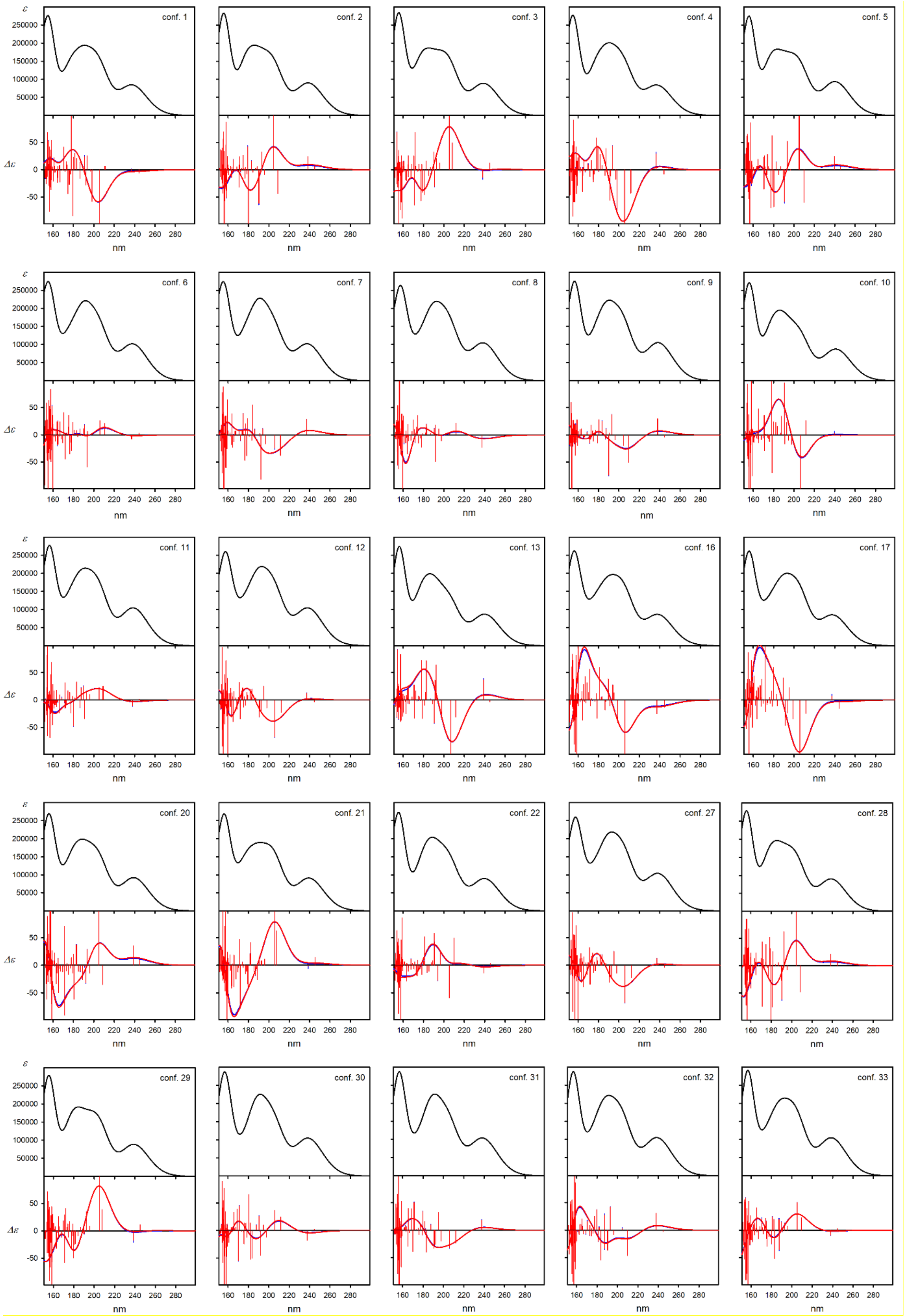

Figure S64. UV (upper panels) and ECD (lower panels) spectra calculated at the IEFPCM/TD-CAM-B3LYP/6-311++G(2d,2p) level for individual low-energy conformers of **18**. Wavelengths were not corrected.

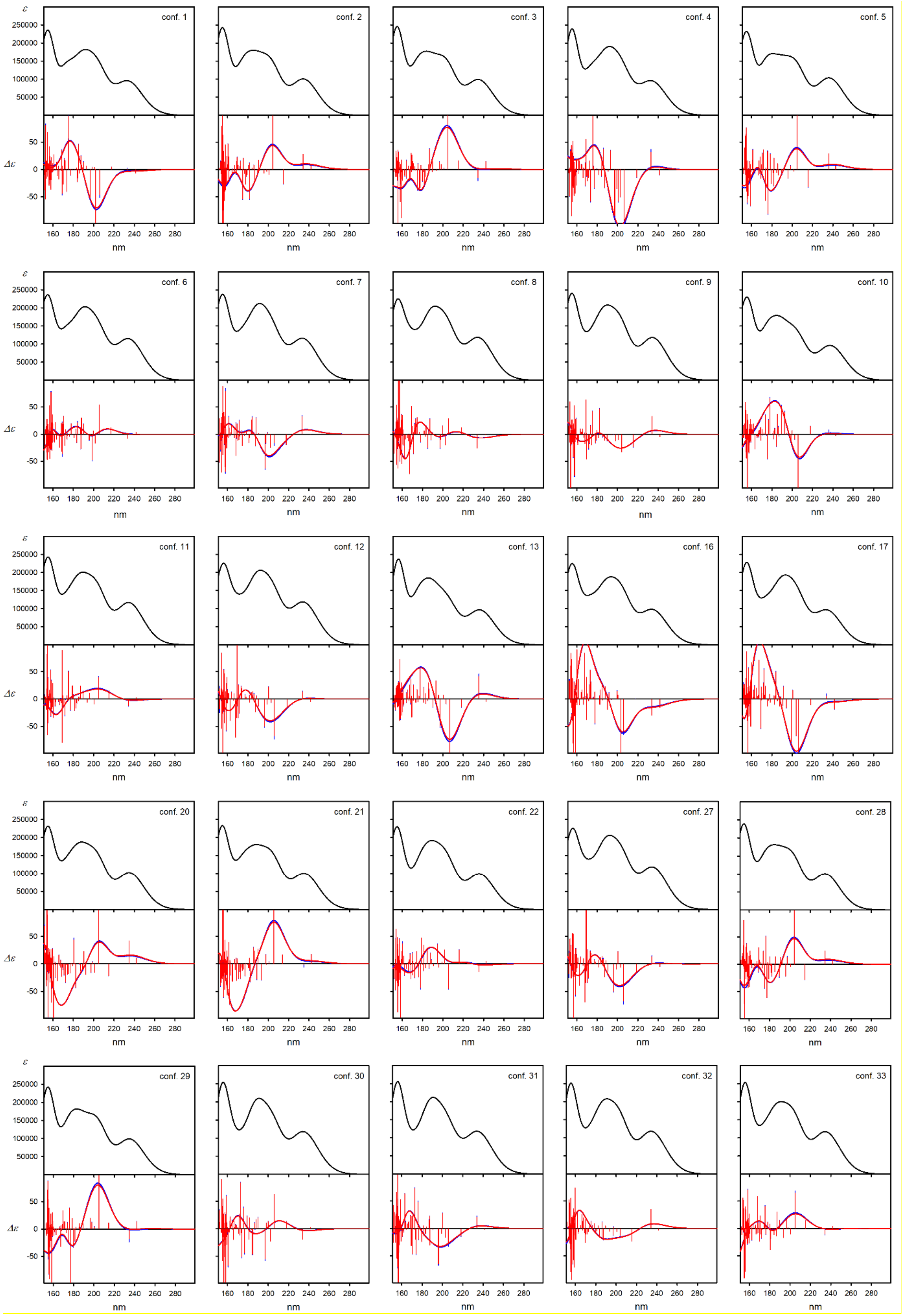

Figure S65. UV (upper panels) and ECD (lower panels) spectra calculated at the IEFPCM/TD-M06-2X/6-311++G(2d,2p) level for individual low-energy conformers of **18**. Wavelengths were not corrected.

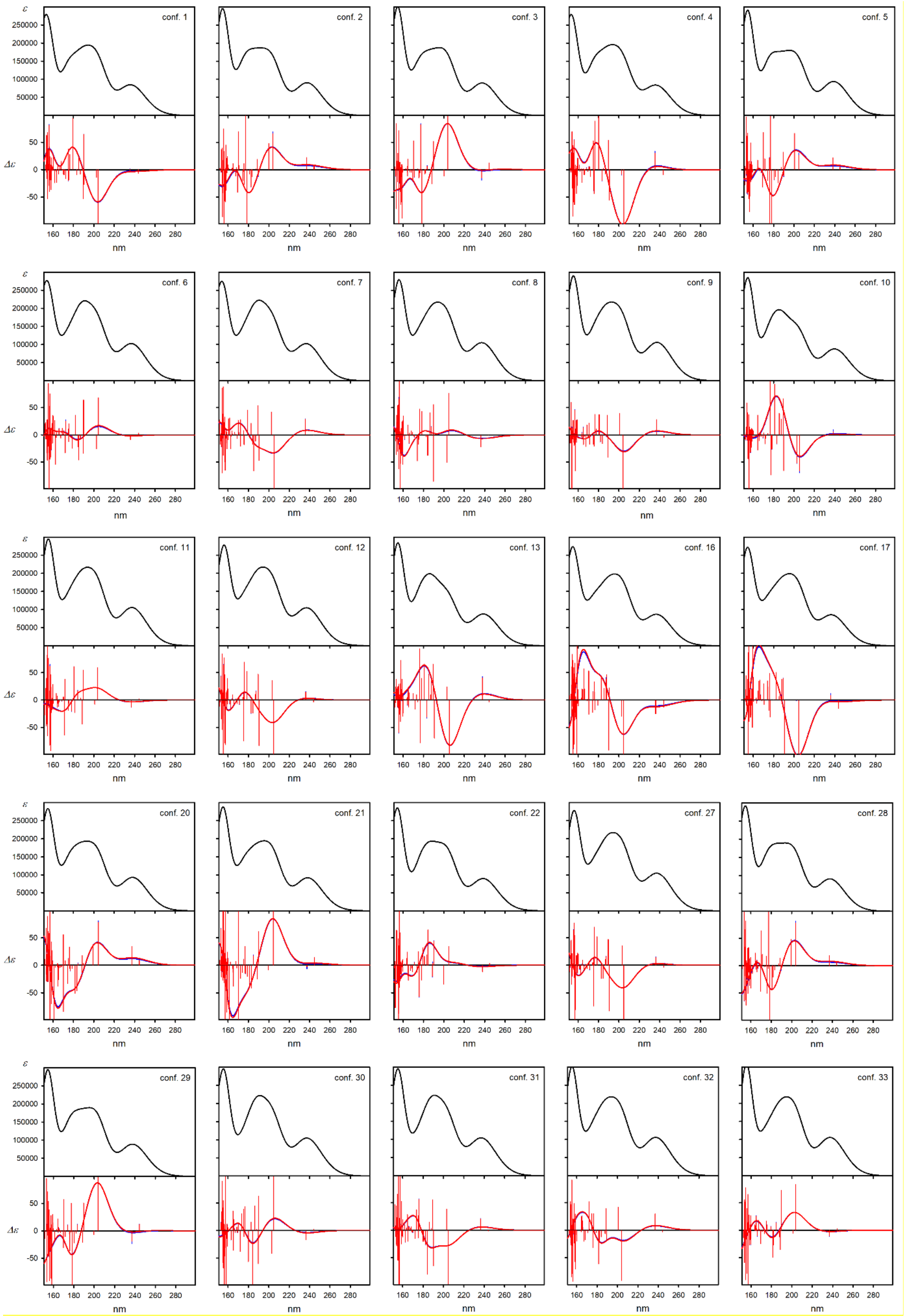

Figure S66. UV (upper panels) and ECD (lower panels) spectra calculated at the IEFPCM/TD-*ω*B97-XD/6-311++G(2d,2p) level for individual low-energy conformers of **18**. Wavelengths were not corrected.

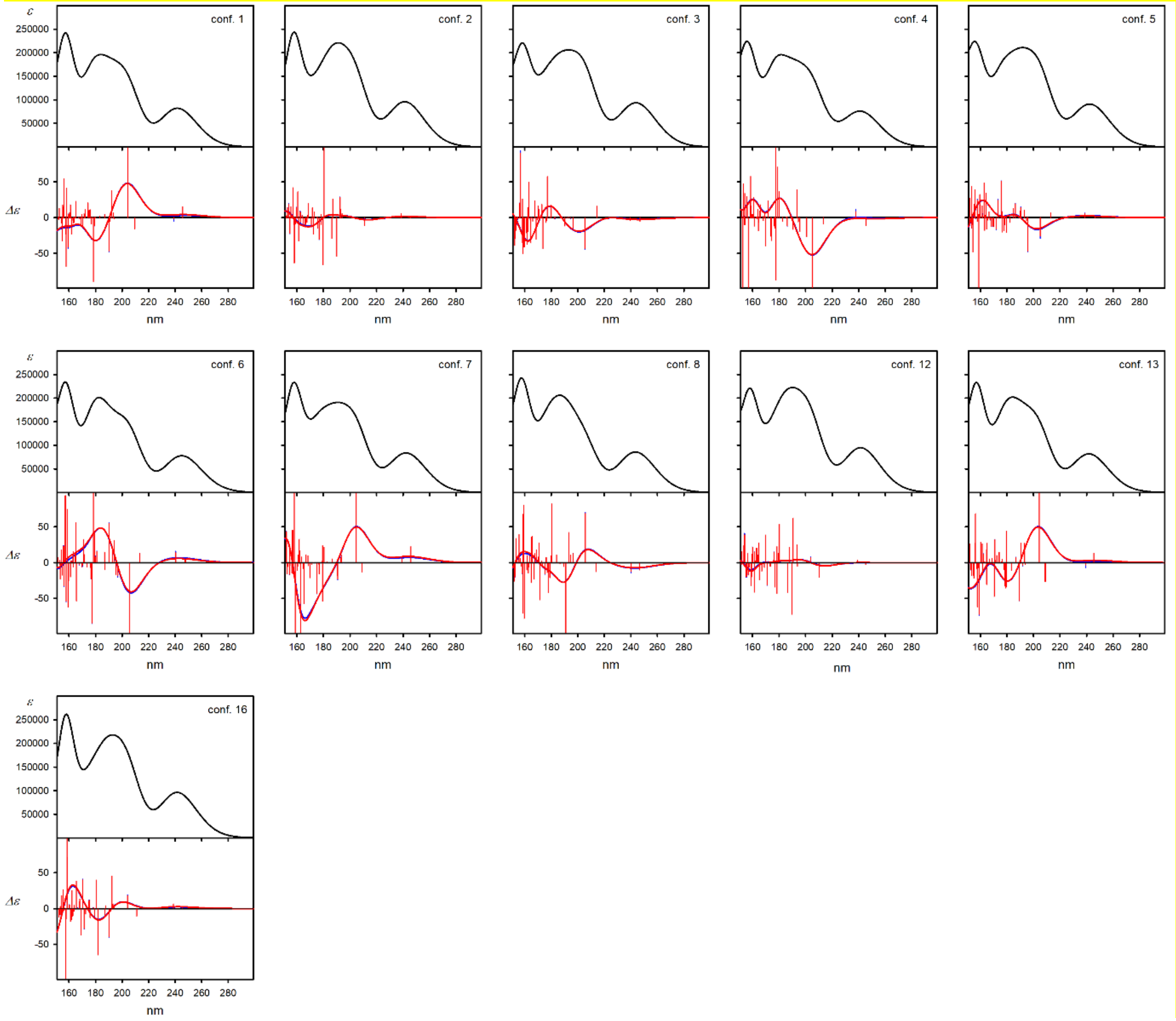

Figure S67. UV (upper panels) and ECD (lower panels) spectra calculated at the IEFPCM/TD-CAM-B3LYP/6-311++G(2d,2p) level for individual low-energy conformers of **19**. Wavelengths were not corrected.

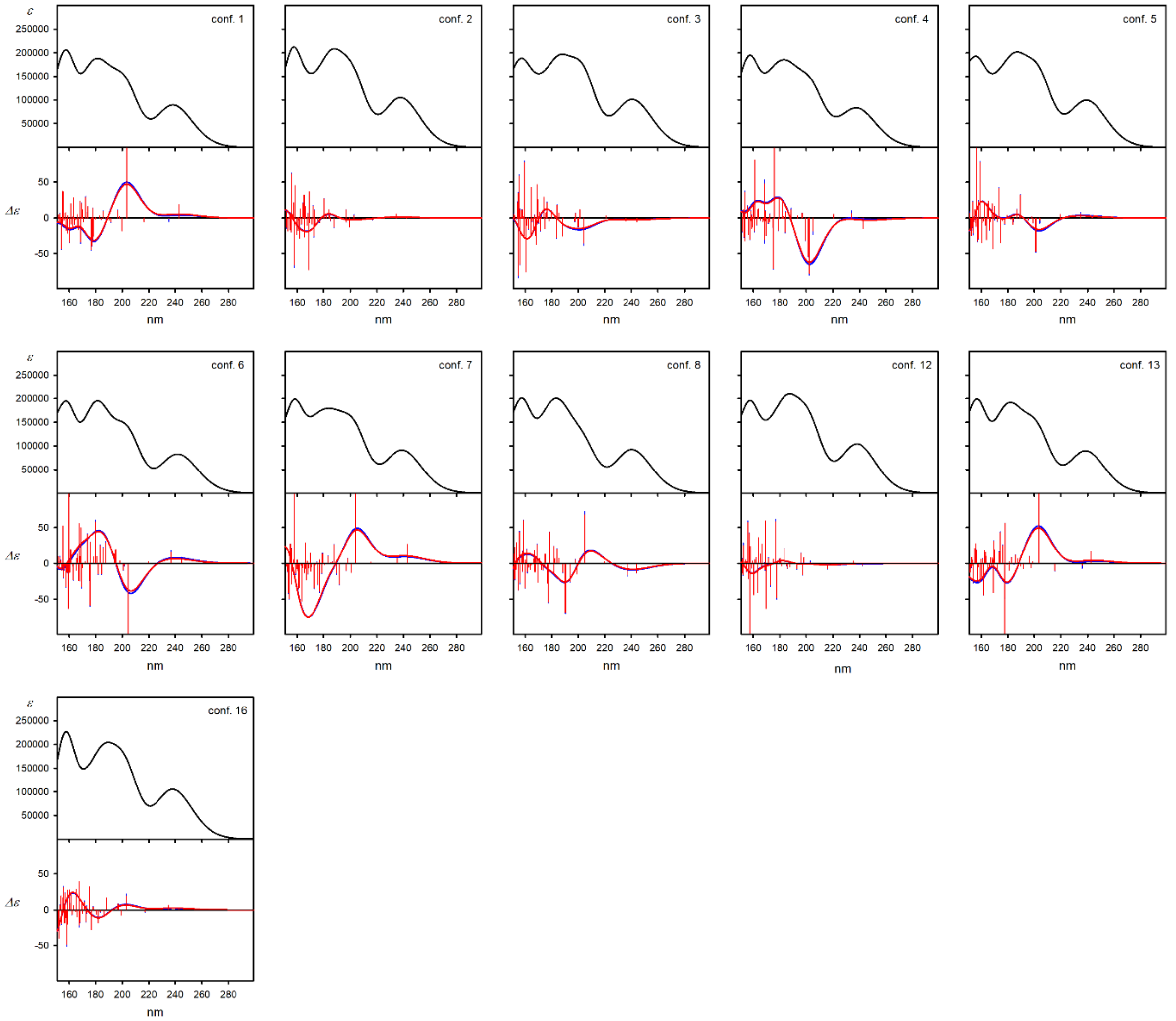

Figure S68. UV (upper panels) and ECD (lower panels) spectra calculated at the IEFPCM/TD-M06-2X/6-311++G(2d,2p) level for individual low-energy conformers of **19**. Wavelengths were not corrected.

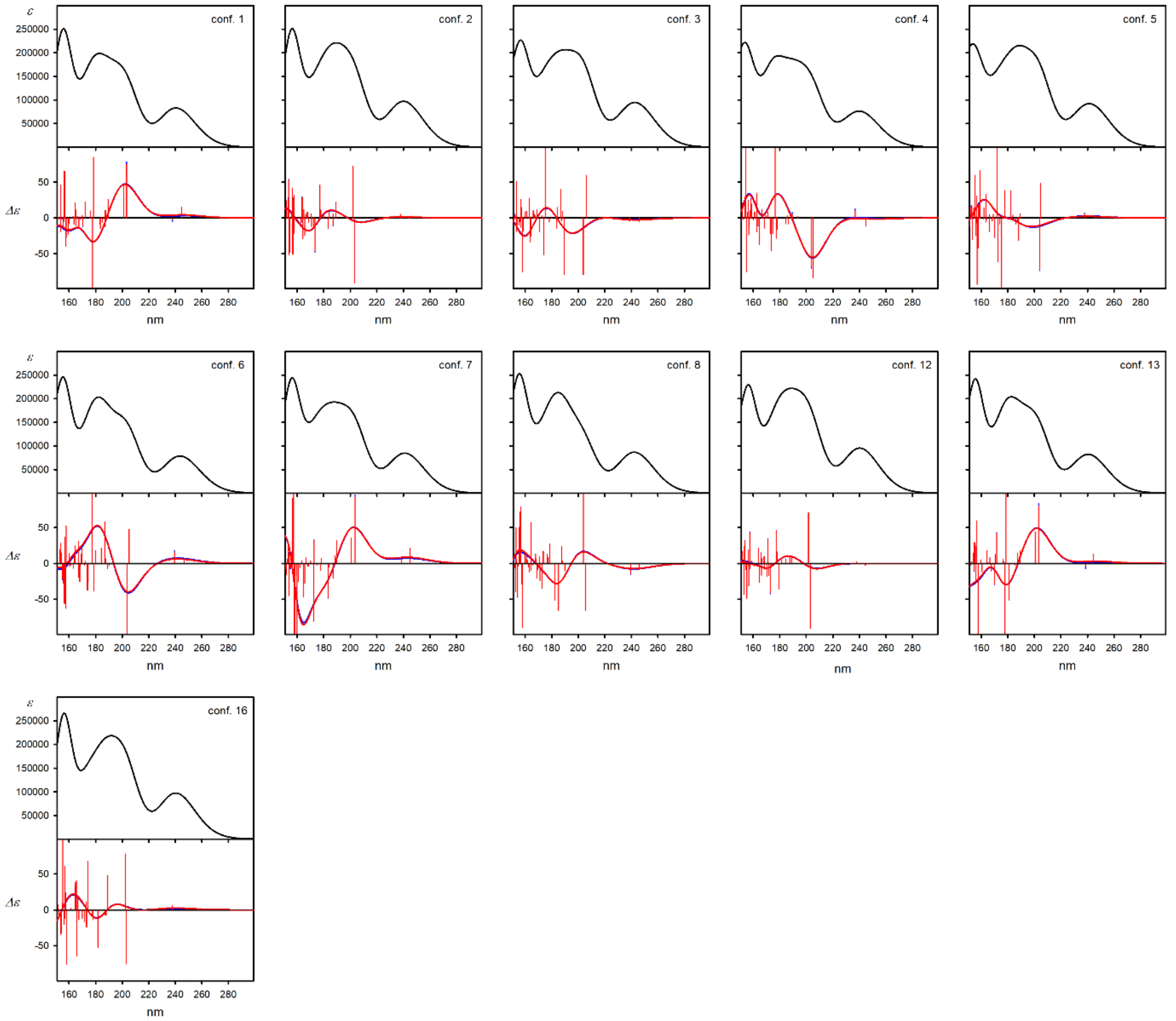

Figure S69. UV (upper panels) and ECD (lower panels) spectra calculated at the IEFPCM/TD-*ω*B97-XD/6-311++G(2d,2p) level for individual low-energy conformers of **19**. Wavelengths were not corrected.

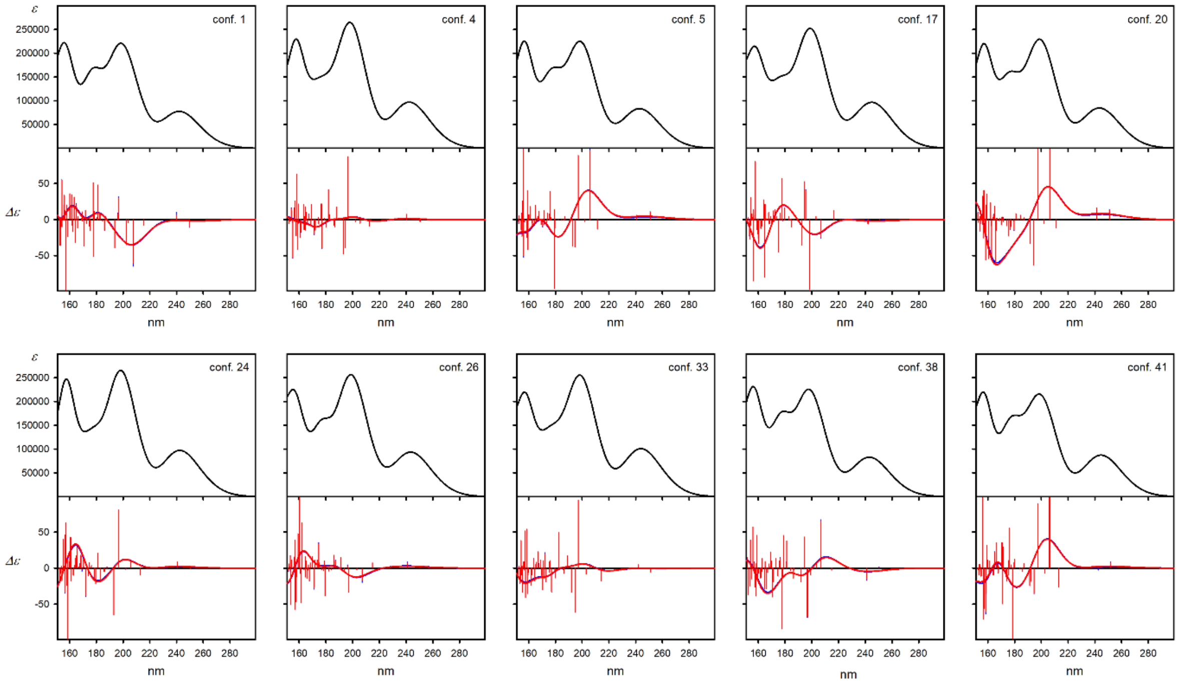

Figure S70. UV (upper panels) and ECD (lower panels) spectra calculated at the IEFPCM/TD-CAM-B3LYP/6-311++G(2d,2p) level for individual low-energy conformers of **20**. Wavelengths were not corrected.

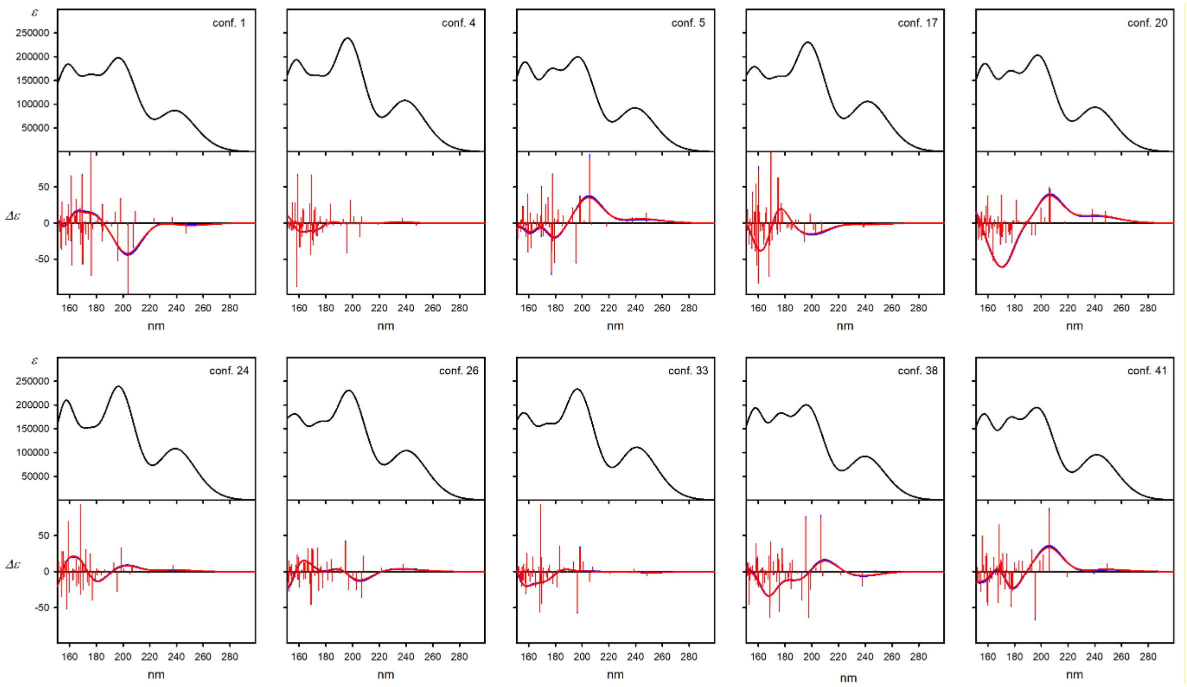

Figure S71. UV (upper panels) and ECD (lower panels) spectra calculated at the IEFPCM/TD-M06-2X/6-311++G(2d,2p) level for individual low-energy conformers of **20**. Wavelengths were not corrected.

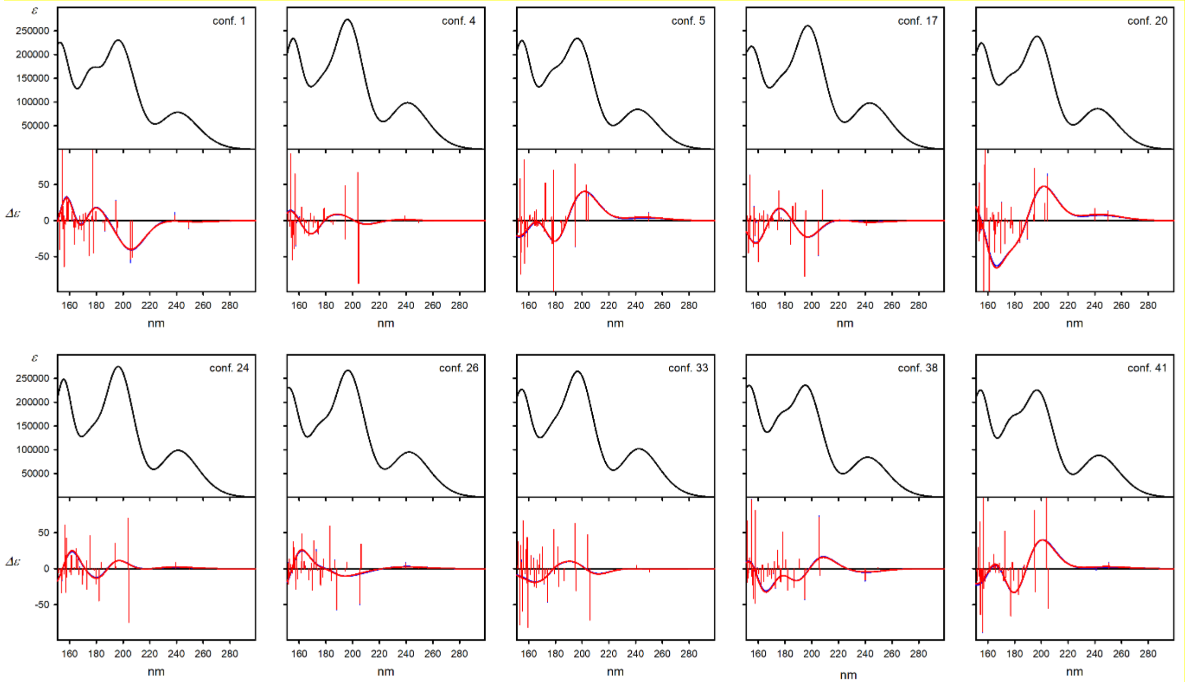

Figure S72. UV (upper panels) and ECD (lower panels) spectra calculated at the IEFPCM/TD-ωB97-XD/6-311++G(2d,2p) level for individual low-energy conformers of **20**. Wavelengths were not corrected.

References

1. Scigress 2.5, Fujitsu Ltd. FUJITSU Technical Computing Solution SCIGRESS. Available Online: https://www.fujitsu.com/global/solutions/business-technology/tc/sol/scigress/ (accessed on 11 September 2020)
2. Gaussian 09. Available Online: https://gaussian.com/glossary/g09/ (accessed on 9 September 2020)
3. Tomasi, J.; Mennucci, B.; Cammi, R. Quantum mechanical continuum solvation models. *Chem. Rev.* **2005**, *105*, 2999–3093.
4. Kwit, M.; Rozwadowska, M.D.; Gawroński, J.; Grajewska, A. Density Functional Theory Calculations of the Optical Rotation and Electronic Circular Dichroism: The Absolute Configuration of the Highly Flexible *trans*-Isocytoxazone Revised. *J. Org. Chem.* **2009**, *74*, 8051–8063 and references therein.
5. Yanai, T.; Tew, D.; Handy, N. A new hybrid exchange-correlation functional using the Coulomb-attenuating method (CAM-B3LYP). *Chem. Phys. Lett.* **2004**, *393*, 51–57.
6. Zhao, Y.; Truhlar, D.G. The M06 suite of density functionals for main group thermochemistry, thermochemical kinetics, noncovalent interactions, excited states, and transition elements: two new functionals and systematic testing of four M06-class functionals and 12 other functionals. *Theor. Chem. Acc.* **2008**, *120*, 215–241.
7. Harada, N.; Stephens, P.J. ECD Cotton effect approximated by the Gaussian curve and other methods. *Chirality* **2010**, *22*, 229–233.
8. Chai J.-D.; Head-Gordon, M. Long-range corrected hybrid density functionals with damped atom-atom dispersion corrections. *Phys. Chem. Chem. Phys.* **2008**, *10*, 6615–6620.

© 2020 by the authors. Submitted for possible open access publication under the terms and conditions of the Creative Commons Attribution (CC BY) license (http://creativecommons.org/licenses/by/4.0/).
